# PRION 2019 emerging concepts

**DOI:** 10.1080/19336896.2019.1615197

**Published:** 2019-05-18

## 

**Co- Editors:**

David Westaway

Centre for Prions and Protein misfolding Diseases, University of Alberta

Hermann Schaetzl

Calgary Prion Research Unit, University of Calgary

Kevin Keough

Alberta Prion Research Institute, Alberta Innovates

Dear attendees,

Welcome to Prion 2019 in downtown Edmonton.

With over 320 participants from 25 countries, we hope you are greatly stimulated by this conference. Over the coming days, 47 oral presentations, over 200 posters, two workshops and three special sessions will deliver cutting-edge concepts to understand and control debilitating prion and related neurodegenerative diseases. We hope you make the acquaintance of new theories, new technologies and - most importantly - new colleagues and collaborators.

We thank all our co-organizers and we wish you a warm welcome from the province nd people of Alberta.

David, Hermann and Kevin

## 1. Interspecies transmission of the chronic wasting disease agent

Justin Greenlee

Virus and Prion Research Unit, National Animal Disease Center, USDA Agriculture Research Service

**ABSTRACT**

The presentation will summarize the results of various studies conducted at our research center that assess the transmissibility of the chronic wasting disease (CWD) agent to cattle, pigs, raccoons, goats, and sheep. This will include specifics of the relative attack rates, clinical signs, and microscopic lesions with emphasis on how to differentiate cross-species transmission of the CWD agent from the prion diseases that naturally occur in hosts such as cattle or sheep. Briefly, the relative difficulty of transmitting the CWD agent to sheep and goats will be contrasted with the relative ease of transmitting the scrapie agent to white-tailed deer.

## 2. RT-QuIC seed amplification assays in the diagnosis of prion diseases, synucleinopathies and tauopathies

Byron Caughey

Senior Investigator at Rocky Mountain Laboratories, NIAID, NIH

**ABSTRACT**

Antemortem diagnoses of many proteopathies can be difficult, due in part to an inability to detect specific causative misfolded protein aggregates with sufficient sensitivity, specificity and practicality. To address this issue we and others have developed RT-QuIC assays that exploit the ability of such aggregates to seed the polymerization of protein monomers into amyloid fibrils. PrP-based RT-QuIC assays for are available for virtually all prion diseases of mammals and have been adapted to multiple specimens including, most recently, the skin and eyes of CJD cases. Analyses of CSF and nasal brushings can give nearly 100% accurate *antemortem* diagnosis of sCJD. We and others have adapted the seed amplification approach to the detection of synucleinopathies such as Parkinson’s disease. αSyn RT-QuIC analyses of patients’ CSF can detect α-synuclein seeds early in the clinical phase of disease and has provided unprecedented diagnostic accuracy. We have also developed three types of ultrasensitive tau RT-QuIC assays optimized for (*i*) Alzheimer disease and chronic traumatic encephalopathy (i.e., 3R/4R tauopathies), (*ii*) Pick disease (a 3R tauopathy), or (*iii*) progressive supranuclear palsy and other 4R tauopathies. Each assay provides strong selectivity for the type of tauopathy for which it was optimized. Such selectivities may underpin the propagation of specific tau aggregate conformers *in vivo*. In summary, these assays can facilitate diagnosis and allow measurements of proteopathic biomarkers. This, in turn, may promote the therapeutic trials by improving patient cohort selection and longitudinal assessments of drug efficacy.

## 3. A perspective on prion strains and their significance

John Collinge

Department of Neurodegenerative Disease, Institute of Neurology, University College London (UCL)

**ABSTRACT**

Mammalian prions are thought to comprise multimeric assemblies of misfolded cellular prion protein (PrP^C^) which propagate by recruitment of PrP monomers and subsequent fission. Although devoid of a nucleic acid genome, mammalian prions exist as multiple strains which can be serially propagated in laboratory animals and produce distinct patterns of disease. Since multiple strains can be maintained in the same inbred mouse line with identical PrP genes, strains cannot be encoded by differences in the primary structure of PrP and it is thought they represent structurally distinct seeds that are able to recruit host PrP^C^ into their distinct polymeric forms. These strain-specific biological and structural properties can be maintained even following passage through an intermediate mammalian species with a different PrP primary structure. In addition to the fundamental biological interest in such protein-based inheritance, it is clear that understanding the prion strain phenomenon is fundamental to understanding prion disease pathogenesis and transmission properties. Critically, prion strains, rather than being a biological clone, appear to constitute a cloud of diverse molecular assemblies or quasispecies which can adapt and mutate under host and other selection pressures. This has an important bearing on therapeutic strategies which need to avoid the development of drug resistance. A proof of principle of targeting neuronal PrP^C^, rather than disease-associated assemblies, was established and our therapeutic approach using an anti-human PrP^C^ monoclonal antibody in patients with CJD is underway. Prion strains may be considered as subtypes of misfolded PrP assemblies with the necessary properties and replication kinetics to evade cellular and other host defences, propagate exponentially and be able to act as an efficient pathogen, while other polymeric PrP forms are degraded or form highly stable aggregates that do not propagate and/or are relatively inert. The very diversity of prion quasispecies, the degree of which may itself vary between strains, may be fundamental to adaptation to a new host (and to particular tissues within it) to allow its survival and spread. This may also account for why some amyloids are infectious pathogens and others not and understanding the structural basis of these different pathobiological properties will be of major importance.

## 4. A unified model of prion infectivity and strain maintenance by cofactor selection

Surachai Supattapone

Departments of Biochemistry and Medicine, Dartmouth College

**ABSTRACT**

The protein-only hypothesis predicts that infectious mammalian prions are composed solely of PrP^Sc^, a misfolded conformer of the normal prion protein, PrP^C^. However, to date, all wild-type protein-only PrP^Sc^ preparations lack significant levels of prion infectivity. Using a systemic biochemical approach, our laboratory isolated and identified two different endogenous cofactor molecules, RNA and phosphatidylethanolamine, which facilitate the formation of prions with high levels of specific infectivity (>10^6^-fold greater than protein-only PrP^Sc^), leading us to propose to the alternative hypothesis that cofactor molecules are required to form wild-type infectious prions. In addition, we found that purified cofactor molecules restrict the strain properties of chemically-defined infectious prions[4], suggesting a “cofactor selection” model in which natural variation in the distribution of strain-specific cofactor molecules in different parts of the brain may be responsible for strain-dependent patterns of neurotropism. Recently, we have discovered that prions with parental strain properties and full specific infectivity can be restored from non-infectious protein-only PrP^Sc^ *in vitro* (Burke, *et al*. PLoS Pathogens, in press). The restoration reaction is rapid, potent, and requires bank vole PrP^C^ substrate, post-translational modifications, and cofactor molecules. These findings provide evidence for a unified model of prion infectivity in which the global structure of protein-only PrP^Sc^ can accurately store latent infectious and strain information, but cofactor molecules control a reversible switch that can unmask full biological infectivity. The results are also consistent with the idea that prion strains select compatible cofactor molecules on the basis of their ability to maintain strain-specific PrP^Sc^ conformations.

## 5. Structural insights into the mechanism of mammalian prion propagation

Witold K. Surewicz

Department of Physiology and Biophysics, Case Western Reserve University

**ABSTRACT**

Despite recent progress in structural studies of ordered protein aggregates such as amyloid fibrils in general, the structure of infectious mammalian prions is poorly understood and controversial. This presentation will discuss structural aspects of prion propagation, with a special focus on a model system of PrP23-144 amyloid that can reproduce in vitro the phenomena of strain multiplicity and transmissibility (seeding) barriers and is amenable to high-resolution structural characterization. Furthermore, we will discuss mass spectrometry-based approaches that can be used for structural characterization of brain-derived prions and the insights provided by these studies regarding the structural basis of prion infectivity.

## 6. Seed-induced Aβ deposition impairs adult neurogenesis in mouse models of Alzheimer’s disease

Meyer-Luehmann

Department of Neurology, Medical Center-University of Freiburg, Germany and Faculty of Medicine, University if Freiburg, Germany

**ABSTRACT**

Alzheimer´s disease (AD) is characterized by severe neuronal loss as well as the accumulation of amyloid-β (Aβ) which ultimately leads to plaque formation. These Aβ deposits can be induced in young pre-depositing APP transgenic mice by intracerebral injection of Aβ-containing brain homogenate. Although a decline of neurogenic capacity in the brain of AD patients and AD mouse models has been reported, our understanding of whether this impairment is specifically altered by Aβ plaques is limited. Here, we find that induced Aβ deposition (Aβ seeding), representing early stages of plaque formation, leads to a dramatic decrease in adult neurogenesis in the hippocampus and olfactory bulb. We further report that the generation and maturation of newborn neurons is affected by the induction of Aβ deposition in both neurogenic niches. Notably, cell death and apoptosis occur in conjunction with Aβ seeding and the exposure to enriched environment and voluntary running reduces Aβ seeding via activated microglia and vivifies neurogenesis and proliferation.

## 7. Modifying prion spread through the CNS

Christina Sigurdson

Department of Pathology, University of California, San Diego

**ABSTRACT**

Prion aggregates increase exponentially and rapidly transit through the central nervous system during prion disease, yet the driving factors from the host and the prion that ultimately govern spread are unclear. Using a series of mouse models with alterations in the extracellular matrix or in the prion conformation, we investigated the determinants of prion propagation through the brain. By targeting heparan sulfate biosynthesis, we find that prion deposition sites shift and the survival time is prolonged in a strain dependent manner. These findings suggest that manipulating endogenous co-factors may be exploited to enhance prion clearance.

## 8. Drivers of neurotoxicity in prion diseases

Adriano Aguzzi

Director, Institute of Neuropathology, University of Zurich

**ABSTRACT**

While the mechanics of prion replication have been subjected to intense studies and are becoming increasingly clear, little is known about what ultimately causes brain damage. It cannot just be mechanical damage from the accumulation of PrPSc, because the latter is present only in trace amounts – in contrast to systemic amyloidoses with enormous amyloid loads in e.g. spleen, liver or peripheral nerves. This suggests the existence of specific pathways of brain damage. If such pathways exist, their components may be exploitable as targets of therapeutic intervention. My lab has focused on the latter question using many different approaches. For example, we have studied the mechanism of action of neurotoxic antibodies against PrP. One such antibody (denominated POM1) was found to be a prion-mimetic, which elicits the same cellular programmes as bona fide prion infections, and can be antagonized by compounds which protect against prion toxicity. Currently, we are exploring the biogenesis of spongiform changes, arguably the most typical consequence of prion disease. It turns out that neuronal vacuolation, the basis of spongiform changes, is controlled by cellular checkpoint molecules that are subverted by prion infection and by prion-mimetic antibodies. The nature of said checkpoints will be discussed.

## 9. Targeting proteopathic seeds in Alzheimer´s disease

Mathias Jucker

Department of Cellular Neurology, Hertie Institute for Clinical Brain Research, University of Tübingen, and German Center for Neurodegenerative Diseases (DZNE), D-72076 Tübingen, Germany

## 10. Prion accumulation in the bone marrow: the origin of prionemia?

Olivier Andreoletti^a^, Jean-Yves Douet^b^, Alvina Huor^b^, Séverine Lugan^b^, Naima Aron^b^, Cécile Tillier^b^, Hervé Cassard^b^ and Olivier Andreoletti^b^

^a^INRA Research Director, UMR INRA ENVT 1225; ^b^UMR INRA ENVT 1225, Interactions Hôtes Agents Pathogènes, 23 chemin des capelles 31076 Toulouse, FRANCE

**ABSTRACT**

The presence of significant amount of Prion infectivity in the bone marrow (BM) of both sCJD and vCJD affected patients has been demonstrated. This raised questions about the role of BM in the occurrence of prionemia and the risk of disease transmission associated with this tissue. To address these questions, we developed a bone marrow transplantation model in PrP^+/+^ and PrP^0/0^ C57Bl_6_ mice. Despite the presence of significant amount of infectivity in the bone marrow of RML infected PrP ^+/+^ C57Bl6 mice, the reconstitution of sub-lethally γ-irradiated mice (8.5 Gy) with this tissue failed to transmit Prion disease or to cause a detectable prionemia. The RML peripheral inoculation of irradiated and BM reconstituted mice clearly demonstrated that the prion replication capacity of the radiosensitive BM cell populations has no detectable impact on the occurrence and the level of Prionemia. It also indicated that the radio resistant cells compartment is associated with the vast majority of the infectivity in the BM. In RML inoculated LT^−/-^ / TNFα ^−/-^ C57Bl_6_, prion accumulated in Bone Marrow tissue but mice displayed a very low/undetectable prionemia. Together these results support the contention that conversely to BM, germinal lymphoid centers play a central role in the occurrence and the level of prionemia.

## 11. Arguments for Alzheimer’s and Parkinson’s diseases caused by prions

Stanley B. Prusiner

Institute of Neurodegenerative Diseases, and Professor of Neurology and Biochemistry, University of California San Francisco

**ABSTRACT**

Arguments for Alzheimer’s (AD) and Parkinson’s diseases (PD) being caused by prions continue to advance with new evidence. Findings in the brains of deceased AD patients argue that both Aβ and tau prions can be demonstrated by bioassays in cultured cells as well as in transgenic (Tg) mice. Likewise, studies of the brains of deceased MSA patients have been found to contain α-synuclein prions by bioassays in cultured cells and Tg mice. Conversely, the brains of AD patients do not contain α-synuclein prions, and the brains of MSA patients do not contain Aβ or tau prions. Additionally, while the brains of patients who died of either progressive supranuclear palsy (PSP) or corticobasal degeneration (CBD) contained tau prions, neither Aβ nor α-synuclein prions were detectable. Merely measuring the levels of Aβ, tau, and α-synuclein appears to give misleading information about the etiology and pathogenesis of neurodegenerative diseases (NDs). From a large array of bioassays, we conclude that AD, PD, MSA, and the frontotemporal dementias, including PSP and CBD, are all prion diseases. Our findings argue that changes in the conformations of Aβ, tau, and α-synuclein underlie the acquisition of prion infectivity in all of these NDs.

## 12. Genetic risk factors for sporadic CJD: replication, expression, function

Simon Mead

UCL Institute of Prion Diseases and NIHR Senior Investigator

**ABSTRACT**

By definition, sporadic Creutzfeldt-Jakob disease is not caused by genetic mutation of the prion protein gene, and the patient has no identifiable environmental exposure. Like common neurodegenerative dementias, age is a powerful risk factor, but the molecular mechanisms of increasing risk with advanced age are unclear. We research both genomic and epigenomic factors in human prion disease by genome-wide association study. Last year we completed initial analysis of genome wide association study with contributions of patient samples from UK, USA, Germany, France, Italy, Netherlands, Austria, Spain, Switzerland and Australia. We have now analysed over 5,000 case samples and a large number of controls. Additional to PRNP, we identified other genetic loci at which multiple SNPs surpass statistical thresholds for genome-wide significance. A SNP at one of these loci, near to STX6, is already known to be a genetic risk factor for a tauopathy, progressive supranuclear palsy. We have followed up these findings by replication, investigation of correlations with clinical phenotypes, allelic expression of genes in blood and brain and in experimental models. Genetic risk factors point to critical aspects of the pathobiology of prion disease and should be assessed for their potential as therapeutic targets.

## 13. A solid-state of conceptualization of information transfer from gene to message protein

Steve McKnight

Department of Biochemistry UT Southwestern

**ABSTRACT**

My presentation will describe speculative ideas and early stage research concerning the flow of genetic information from the nuclear residence of genes to the disparate, cytoplasmic sites of protein synthesis. This process is particularly important for neurons, in which the nuclear location of genes may be far-removed from localized sites of mRNA translation at active synapses. I propose that this process of information transfer is meticulously guided by transient structures formed from protein segments of low sequence complexity or intrinsic disorder. These low complexity domains are ubiquitously associated with regulatory proteins that control gene expression and RNA biogenesis, but they are also found in the central channel of nuclear pores, the nexus points of intermediate filament assembly, and the locations of action of other well-studied cellular proteins and pathways. Upon being organized into localized cellular positions via mechanisms utilizing properly folded protein domains, thereby facilitating elevated local concentration, certain low complexity domains adopt cross-β interactions as described 60-70 years ago by Linus Pauling. Derivation of the atomic structure of labile polymers formed from the low complexity domain of the fused in sarcoma (FUS) RNA binding protein has demonstrated how these cross-β interactions can be both structurally specific and labile to disassembly. These weakly tethered assemblies, I propose, are built to relay the passage of genetic information from one site to another within a cell, ensuring that the process is of extreme fidelity.

## 14. Beyond neurofibrillary tangles in tauopathy

Karen Hsiao Ashe and N. Bud Grossman

Center for Memory Research and Care, Edmund Wallace and Anne Marie Tulloch Chairs in Neurology and Neuroscience at the University of Minnesota

**ABSTRACT**

Soluble forms of tau, unrelated to neurofibrillary tangles, play pathogenic roles in tauopathies.  A soluble tau fragment, Δtau314 that resists forming fibrils forms when caspase-2 (CASP2) cleaves human tau at Asp314.  Δtau314 is elevated in forebrain tissue of humans with mild cognitive impairment, Alzheimer's disease, Huntington's disease and Lewy body dementia.  This cleavage event, along with tau phosphorylation, leads to tau mislocalization and reduced AMPA receptor levels in dendritic spines and abnormal post-synaptic function (reduced mEPSCs).  Rendering endogenous mouse tau (mTau) non-cleavable by CASP2 by introducing a homologous mutation in mTau, Asp303-to-Glu reduces mTau in dendritic spines, increases excitatory synaptic transmission and resistance to excitotoxin-induced seizures, and ameliorates premature mortality in transgenic mice expressing human amyloid precursor protein.  These data support a role for non-amyloid forms of proteins present in the neuropathological amyloid-containing structures (e.g., tangles) that characterize their respective diseases, and suggest a strategy for repairing synaptic transmission in tauopathies by inhibiting CASP2.

## 15. Cellular prion infection: from traffic jams to new drug targets

Sabine Gilch

Canada Research Chair in Prion Disease Research, University of Calgary

**ABSTRACT**

Prion infection on a cellular level is characterized by the presence of PrP^Sc^ aggregates at the plasma membrane, in vesicles along the endo-lysosomal pathway and in recycling endosomes, as well as an increased level of unesterified cholesterol. High cholesterol content can affect membrane properties and vesicle trafficking. Our goal is to understand the impact of prion infection on endo-lysosomal vesicle trafficking, to define a link with aberrant cholesterol metabolism and to validate new therapeutic compounds that target those pathways. We show that prion infection induces a ‘traffic jam’ by impairing with retrograde cargo transport and lysosomal degradation capacity, processes that require functions of the retromer complex and rab7, respectively. We show that stimulating retrograde transport, lysosomal degradation capacity and enhancing cholesterol efflux in neuronal cells reduces prion propagation. Overall, our studies provide new insights into prion-induced cellular dysfunction. We observed alterations similar to those described in Alzheimer’s disease, indicating that the endo-lysosomal system might be an intersection in the pathogenesis of those neurodegenerative diseases.

## 16. Design and optimization of nucleic acid derivatives for inhibition of prion-like propagation at W32 site of SOD1 enzyme in Amyotrophic Lateral Sclerosis

Vijaya Kumar Hinge^a,b^, Nikolay Blinov^a,b^, Dipankar Roy^a,b^, W. Ted Allison^a^, David S. Wishart^a^ and Andriy Kovalenko^a,b^

^a^University of Alberta, Edmonton, Canada; ^b^Nanotechnology Research Centre, Edmonton, Canada

**CONTACT** Vijaya Kumar Hinge hinge@ualberta.ca

**ABSTRACT**

Misfolding of human Cu/Zn superoxide dismutase (SOD1) cytosolic metalloenzyme results in the formation of neurotoxic oligomers in familial Amyotrophic Lateral Sclerosis (fALS). Over 180 mutations have been identified and linked to the gene encoding of SOD1 in fALS. Currently, there is no cure for ALS and only two Food and Drug Administration (FDA)-approved drugs, Riluzole and Edaravone, are available for treatment of ALS patients. The human cell culture studies demonstrated that misfolded SOD1 propagates from cell to cell in a prion-like fashion and W32 residue is important for templated misfolding of wild type SOD1 (wtSOD1) into toxic aggregates [1]. The recent animal studies on ALS Zebrafish embryo model suggest that mutation of W32S decreases the toxicity to motor neuron functions [2]. The inhibition of pathological misfolding of SOD1 via drug-like molecules binding at W32 site is a novel and promising therapeutic strategy for the development of ALS therapy.

*In silico de novo* drug design and pharmacophore-restrained high-throughput virtual screening (HTVS) methods were used to identify the anti-ALS lead candidates at W32 site. A library of commercially available lead-like compounds (650,000) and Food and Drug Administration (FDA) approved small molecules were screened at W32 site of SOD1 using HTVS and were filtered through a uracil-based pharmacophore model. Based on the analysis of physico-chemical descriptors and pharmacophore features of the experimental binding mode of 5-FUrd at W32, a group of new Anti-ALS lead compounds were rationally designed. Anti-ALS lead candidates were ranked with in-house quantitative structure–activity relationship (QSAR) models based on the 3D-RISM-KH molecular solvation theory derived molecular descriptors which accurately predicted the blood brain barrier (BBB) permeability [3]. A new protocol was developed to account for structural solvation effects and was successfully validated via predicting experimental binding modes of 5-FUrd at W32 site of SOD1. The protocol combines the water placement algorithm based on the 3D-RISM-KH molecular theory of solvation and molecular docking simulations implemented in the Molecular Operating Environment (MOE) integrated drug discovery package. One of the anti-ALS lead compound, Telbivudine, tested in ALS-Zebrafish embryo model significantly rescued axonopathy in a dose-dependent manner [2,4]. The newly designed lead-like compounds were selected by virtual screening, and *de novo* drug design methods show higher affinity compared to the Telbivudine and can potentially inhibit prion-like propagation of misfolding of SOD1.

### 

References[1]GradL, GuestWC, Yanai A, et al.
Intermolecular transmission of superoxide dismutase 1 misfolding in living cells. Proc Natl Acad Sci USA. 2011;108:16,398–141.10.1073/pnas.1102645108PMC318270521930926[2]Duval et al. Neurobiology of disease
2019;124:297–310.10.1016/j.nbd.2018.11.02530528257[3]Roy et al. *ACS Omega*. 2019
(accepted).[4]Treatment of amyotrophic lateral sclerosis (ALS). Patent application no
62/637,013. USPTO (pending); 2018.

## 17. Large-scale production of phospholipid cofactor recombinant prions with high specific infectivity

Daniel J. Walsh^a^, Marcus D. Tuttle^b^, Cassandra M. Burke^a^, Kurt W. Zilm^b^, and Surachai Supattapone^a^^,^^d^

^a^Departments of Biochemistry and Cell Biology, Geisel School of Medicine at Dartmouth, Hanover, New Hampshire, USA; ^b^Department of Chemistry, Yale University, New Haven, Connecticut, USA; ^c^Medicine, Geisel School of Medicine at Dartmouth, Hanover, New Hampshire, USA

**CONTACT** Daniel J. Walsh daniel.j.walsh.GR@dartmouth.edu

**ABSTRACT**

The fundamental event underlying mammalian prion infectivity is the induced conformational change of PrP^C^ into PrP^Sc^. A major goal in the field is to determine the structural mechanism of this conversion process, and a critical step towards this goal would be to determine the protein structure of fully infectious PrP^Sc^. One technique that could be used to determine the structure of infectious PrP^Sc^ with high resolution is solid-state (ss)NMR, but application of this method requires the production of milligram quantities of a homogeneous, isotopically-labelled PrP^Sc^ sample whose specific infectivity is equivalent to that of authentic brain-derived PrP27-30 (~10^6^ LD_50_/µg PrP) [1].

We previously used sPMCA to produce two chemically defined recombinant (rec)PrP^Sc^ conformers originally derived from the same seed. Whereas cofactor recPrP^Sc^ produced with phospholipids displayed high levels of specific infectivity, protein-only recPrP^Sc^ produced from PrP alone was completely non-infectious [2]. A limitation of PMCA technology is that it can only be used to produce microgram quantities of PrP^Sc^, and therefore is not a feasible option for generating ssNMR samples. To overcome this limitation, we produced large quantities of cofactor recPrP^Sc^ and protein-only recPrP^Sc^ by shaking rather than PMCA. Shaking-produced cofactor recPrP^Sc^ caused scrapie in both wild-type mice and bank voles (homozygous M109), while protein-only recPrP^Sc^ failed to infect either species. End-point titration bioassays indicated that the specific infectivity of shaking-produced cofactor recPrP^Sc^ was ~10^6^ LD_50_/µg PrP, while that of protein-only recPrP^Sc^ was <5 LD_50_/µg PrP. These results confirm that shaking-produced phospholipid cofactor recPrP^Sc^ is a good candidate for structural studies of bona fide mammalian prion infectivity. The morphologies of cofactor recPrP^Sc^ and protein-only recPrP^Sc^ molecules could not be easily distinguished by negative-stain electron microscopy, but differences in the secondary structure between the two conformers can be detected by ssNMR.

### 

References[1]DeleaultNR, HarrisBT, ReesJR, et al.
Formation of native prions from minimal componenets
*in vitro. Proc Natl Acad Sci U S A*.
2007;104(23):9741–9746.1753591310.1073/pnas.0702662104PMC1887554[2]DeleaultNR, WalshDJ, PiroJR, et al.
Cofactor molecules maintain infectious conformation and restrict strain properties in purified prions. *Proc Natl Acad Sci U S A*. 2012;109(28):E1938–E1946.10.1073/pnas.1206999109PMC339648122711839

## 18. Targeting protein aggregation through nanoparticle-based inhibitors

Bibin G. Anand^a,b,c^, Kailash Prasad Prajapathi^b^, Kriti Dubey^b^ and karunakar Kar^b^

^a^Department of Bioscience and Bioengineering, Indian Institute of Technology Jodhpur, India; ^b^School of Life science Jawaharlal Nehru University, New Delhi, India; ^c^Center for Prions and Protein folding diseases, University of Alberta, Edmonton, Canada

Abstract not available.

## 19. Amplification of prions derived from single cells isolated from different brain regions of RML infected animals by laser capture microdissection

D. Gorski^a^, A. Lyon^a^, A. Mukherjee^a^, S. Medina^b^, S. Booth^b^, S. Pritzkow^a^ and C. Soto^a^

^a^Mitchell Center for Alzheimer’s disease and related Brain disorders, University of Texas Medical School at Houston; ^b^Zoonotic Diseases and Special Pathogens, National Microbiology Laboratory, Public Health Agency of Canada

**CONTACT** D. Gorski Damian.A.Gorski@uth.tmc.edu

**ABSTRACT**

Prion diseases are uniformly fatal neurodegenerative disorders characterized by the misfolding and aggregation of host-encoded prion protein (PrP^C^) into a β-sheet rich, insoluble, and protease-resistant conformation termed PrP^Sc^, which serves as the sole infectious agent. Depending on the prion disease, PrP^Sc^ can adopt different isoforms, which preferentially deposit in distinct brain regions and differentially accumulate in the neurons, astroglia, and microglia of these areas. Currently, the cellular factors and processes governing the differential cellular vulnerability to PrP^Sc^ deposition and damage amongst different cell types and brain regions are not known. The putative differences in biochemical, biological, and structural characteristics of PrP^Sc^ associated with different cells and regions is also yet to be described. Indeed, several previous reports have shown that different prion strains can co-exist in the same brain and may be masked by a single dominant pathological phenotype. In this study, we optimized the protein misfolding cyclic amplification (PMCA) technology to amplify PrP^Sc^ from a single cell. For these studies we used a cell line chronically infected with RML prions and dilute it to add individual cells per sample. Serial dilutions of infected cell culture showed that PMCA enabled amplification of prions from a single cell. This was further confirmed by plating cell dilutions. The presence of a single cell was confirmed via microscopy. We then applied this technique to amplify PrP^Sc^ from individual cells extracted from the brain of prion infected animals via laser capture dissection. We show successful amplification of prions from individual neurons from some areas of the brain and are currently analysing the presence of PrPSc in neurons and glial cells extracted via LCM from the cerebellum, thalamus, cortex, and hippocampus of RML infected mice. Partial fixation and staining of the tissue prior to LCM did not interfere with PMCA amplification. This technology will enable to study the characteristics of prions associated with different cell types isolated from distinct areas of the brain of animals and humans infected with different prion strains. Experiments can be done comparing PrP^Sc^ in neuronal versus glial cell types originating from distinct brain regions as well distinct time points of disease progression. These findings can then be coupled with single cell genomics and proteomics analysis. These findings will shed light on the molecular basis of differential cellular vulnerability to prion infection and the nerve tropism of distinct prion strains.

## 20. New insights into the structure of bovine spongiform encephalopathy (BSE) prions

Razieh Kamali-Jamil^a,b^, Sara Amidian^a,b^, Brian Tancowny^a,b^, Xiongyao Wang^a,b^, Ester Vázquez-Fernández^a,b^, Xinli Tang^a^, Howard Young^b^ and Holger Wille^a,b^

^a^Centre for Prions and Protein Folding Diseases, University of Alberta, Edmonton, Canada; ^b^Department of Biochemistry, Faculty of Medicine & Dentistry, University of Alberta, Edmonton, Canada

**CONTACT** Razieh Kamali-Jamil Kamalija@ualberta.ca

Present address: Xiongyao Wang, School of Materials Science and Engineering, Harbin Institute of Technology, Weihai, Shandong, China

**ABSTRACT**

**Background**: Bovine spongiform encephalopathy (BSE) is caused by the conversion of the cellular prion protein (PrP^C^) to the infectious form (PrP^Sc^). Prion diseases in cattle include classical BSE (C-type BSE), and atypical strains known as H-type and C-type BSE. Consumption of BSE-infected meat products may have caused variant Creutzfeldt-Jakob disease in humans. Previously, the structure of the recombinant prion protein has been analysed using X-ray crystallography and NMR spectroscopy methods. However, the PrP^Sc^ has hampered all attempts at structural determination due to its insolubility and propensity to aggregate. A recent cryo-EM study on glycosylphosphatidylinositol-anchorless PrP^Sc^ proposed a four-rung ß-solenoid architecture for individual PrP^Sc^ molecules. Currently, no three-dimensional structure is available for BSE prions. The aim of this project is the characterization and detailed analysis of the structure of BSE prions.

**Materials and Methods**: L-type BSE prions were purified from the brains of prion-infected transgenic mice expressing bovine PrP^C^, using phosphotungstate anions followed by sucrose gradient centrifugation. The purification quality was assessed using SDS PAGE followed by silver staining or Western blotting. The purified samples were analysed via negative stain electron microscopy to determine the quality of the sample preparations. We also developed an immunogold labelling protocol for the specific detection of BSE fibrils on electron microscopy grids. Next, electron micrographs of individual BSE fibrils were analysed through a variety of image processing techniques in order to obtain three-dimensional reconstructions. For this purpose, fibril images with a clear helical twist were selected, segmented, aligned, classified, and oriented. Subsequently, a three-dimensional volume could be generated through back projection.

**Results and Conclusion**: The successful purification of BSE prion fibrils was confirmed by Western blotting and negative stain EM. Image analyses revealed a heterogeneous population of BSE fibrils consisting of two intertwined protofibrils, in agreement with the results of previous studies. Additionally, fibrils with only one protofilament were observed, which is a novel finding. 2D class averages on more than 500 fibrils further confirmed the two distinct morphologies of the L-type BSE fibrils. We postulate that the two-protofilament BSE fibrils result from the interaction of two individual fibrils. Thus, our findings reveal differences on the quaternary structure level. Lastly, these observations will be helpful for future efforts to obtain high-resolution information on the structure of BSE prions.

## 21. Proximity-dependent biotinylation identifies protein interactors of pathological tau and synuclein in human ESC-derived neurons

Tagan A. Griffin, Elisa M. Cleveland, Robert W. Newberry, George A. Carlson, Paul D. Schnier and Stanley B. Prusiner

Institute for Neurodegenerative Diseases, Weill Institute for Neurosciences, University of California, San Francisco, CA, USA

**CONTACT** Tagan A. Griffin tagan.griffin@ucsf.edu

**ABSTRACT**

Tau and synuclein can act as prions, undergoing templated conformational changes resulting in the accumulation of aggregated proteins, which drives the spread of neurodegeneration throughout the brain. It is unclear which other cellular proteins contribute to this process. To investigate this question, we utilized a promiscuous biotin ligase (BioID2) fused to tau or synuclein to biotinylate interacting proteins in human embryonic stem cell (ESC)–derived neurons following treatment with disease-causing recombinant fibrils (tau K18 or synuclein preformed fibrils [PFFs]). To identify interactors in a relevant cell type, we first generated a stable human ESC line (H1 ESCs) expressing neurogenin 2 (NGN2) under the control of an inducible promoter using PiggyBac transposon vectors. Upon doxycycline induction, this ESC line differentiated into a nearly pure population of glutamatergic neurons in under 3 weeks. Additional vectors were used to generate ESC lines that differentiated into neurons expressing tau-BioID2 (WT or P301L), synuclein-BioID2 (WT or A53T), and a control line expressing BioID2 alone. Following neuronal differentiation, recombinant fibrils were added to the culture media to induce aggregation, and cells were harvested 24 h or 3 weeks later. Following denaturing lysis and streptavidin purification, the biotinylated proteins were subjected to quantitative mass spectrometry using tandem-mass tag (TMT 10-plex) labels, enabling direct comparisons between multiple conditions (WT vs. mutant protein, ± fibrils, and early vs. late time points). We identified a number of known tau and synuclein interactors as well as previously unreported candidate interactors, many of which were less abundantly biotinylated in neurons treated with fibrils relative to neurons not treated with fibrils. This was accompanied by a striking increase in the abundance of biotinylated tau or synuclein, consistent with aggregate formation and a concomitant loss of physiological interactions. We also found several other biotinylated proteins whose abundances increase following the addition of fibrils. Tau-BioID2 and synuclein-BioID2 shared few interactors in common, consistent with a model of prion replication driven by tau or synuclein alone. These results identify novel candidate interactors of tau and synuclein, and shed light on the mechanisms of prion infection and replication in human neurons.

## 22. Incapability of mutant PrP^Sc^ to recruit wild-type PrP^C^ in a familial prion disease linked to PrP^Q227X^ mutation implies a role of GPI anchor in prion propagation

Pingping Shen^a,b^, Johnny Dang^a^, Zerui Wang^a,b^, Jue Yuan^a^, Yue Lang^a,b^, Jiachun Feng^b^, Qingzhong Kong^a,b,c^, Annemiek J. M. Rozemuller^d^, Li Cui^b^, Robert B. Petersen^a,b^, Wen-Quan Zou^a,b,e^

^a^Department of Pathology and Neurology, Case Western Reserve University, Cleveland, OH, USA; ^b^Department of Neurology, First Hospital of Jilin University, Changchun, Jilin Province, China; ^c^National Prion Disease Pathology Surveillance Center, Case Western Reserve University, Cleveland, OH, USA; ^d^Dutch Surveillance Center for Prion Diseases, University Medical Center Utrecht Heidelberglaan 100, Utrecht, and Netherlands Brain Bank, Amsterdam, The Netherlands; ^e^Foundation of Science, Central Michigan University College of Medicine, Mount Pleasant, MI, USA

**CONTACT** Wen-Quan Zou wxz6@case.edu

**ABSTRACT**

Cellular prion protein (PrP^C^) is a glycoprotein anchored to the cell surface by a glycosylphosphatidylinositol (GPI) anchor. The key molecular event in the pathogenesis of prion disease is the conversion of PrP^C^ into its pathological isoform PrP^Sc^. Several lines of evidence have indicated that this event takes place on the cell surface and alternation of PrP^C^ localization on the cell surface by mediating the GPI anchor dramatically affects the formation and infectivity of PrP^Sc^. Notably, anchorless PrP in transgenic (Tg) mice produced deposition of PrP amyloid in the brain, but caused no neurological signs in animals. A patient with an anchorless PrP^Q227X^ stop codon mutation was reported to exhibit a GSS-like prion disease phenotype. Here we examined the anchorless PrP from the brain of the patient with PrP^Q227X^ mutation and compared the physicochemical properties of secreted and un-secreted PrP isoforms from a transfected human neuron line expressing PrP^Q227X^. Epitope mapping revealed for the first time that the PrP^Sc^ from the patient with Q227X was derived from the mutant allele only. Fractionation of cultured human neuroblastoma cells expressing human wild-type or Q227X mutant PrP demonstrated most wild-type PrP (PrP^WT^) being recovered in the cell lysate fraction, and most mutant PrP^Q227X^ recovered in the medium fraction, suggesting that PrP^WT^ was attached to the cell surface, while PrP^Q227X^ was secreted into the media. In addition, the mutant PrP lacked the N-linked glycans found in PrP^WT^. Two small fragments migrating at approximately ~6–8 kDa were identified in the culture media, but not in cell lysate fraction. Finally, typical PrP^Sc^ three bands were amplified with normal humanized transgenic mouse brain as the PrP^C^ substrate by serial protein misfolding cyclic amplification. Our finding that PrP^Sc^ derived from the mutant GPI-anchorless PrP failed to recruit cell surface-attached PrP^WT^ suggests that the GPI anchor plays an important role in prion propagation when mutant and wild-type alleles are present *in vivo*.

**Funding**

Supported in part by the CJD Foundation and the National Institutes of Health (NIH) NS062787 and NS087588 to W.Q.Z., NS062787 and NS109532 to W.Q.Z., and Q.K., NS088604 to Q.K. as well as the National Natural Science Foundation of China [No. 81,671,186] to LC.

## 23. Inferring the effects of deer population and landscape structure on spread of the chronic wasting disease in Alberta

Peter Smolko^a^, Dana Seidel^b^, Evelyn Merrill^a^, Margo Pybus^a,c^, Anne Hubbs^c^ and Mark Ball^c^

^a^Department of Biological Sciences, University of Alberta, Edmonton, *AB*, Canada; ^b^Department of Environmental Science, Policy & Management, University of California Berkeley, CA, USA; ^c^Alberta Fish & Wildlife Division, Government of Alberta, Edmonton, *AB*, Canada

**CONTACT** Peter Smolko smolko@ualberta.ca

**ABSTRACT**

Chronic wasting disease (CWD) is a 100% fatal prion disease of cervids that has been detected in wild deer and elk and moose in two Canadian provinces. Alberta has had a surveillance programme for CWD since 1996. CWD in wild deer in Canada was detected in adjacent Saskatchewan (2000) and later in Alberta (2005). Based on data collected during the Alberta surveillance programme we assessed the risk of a harvested mule deer and white-tailed deer having CWD over a 15-year period (2000–2016) in eastern Alberta. We hypothesized that the probability of a hunter harvested deer being CWD positive was a function of time since the disease was first detected in the area (2000), sex and age of the deer and characteristics of the area where the deer was harvested (agricultural areas, extent of woody cover, distance to rivers and streams, topography, road density or distance to human settlements, and proximity to other positive CWD cases). We used previously developed risk model using surveillance data from 2000–2012 (*n* = 162 CWD cases) to predict risk in deer harvested in 2013–2016. We then redeveloped the risk model using data from 2000–2016 (*n* = 591) and tested the predictions of this model using positives from 2017 (*n* = 327). We also assessed how species, sex, importance of site characteristics, and distance to known CWD cases changed over time in influencing predicted risk of a deer having CWD in the core area and on the edge of the CWD surveillance zone. The top ranked model supported a pattern of increasing prevalence of CWD in hunter harvested deer over time. The risk among mule deer was higher compared to white-tailed deer and among males compared to females; however, the difference between species decreased and between sexes increased compared to early period. The CWD risk was greater in areas close to rivers with abundant woody cover where surveillance and control efforts should be focused.

**KEYWORDS:** Mule deer; white-tailed deer; transmissible spongiform encephalopathy; prion-protein; prevalence; disease management

## 24. Effect of the exosomal prion protein on the amyloid-β aggregation kinetics

Mohsin Shafiq^a*^, Alexander Hartmann^a*^, Stefano Da Vela^b*^, Christiane Muth^a^, Behnam Mohammadi^a^, Luise Linsenmeier^a^, Hermann Altmeppen^a^, Susanne Krasemann^a^, Dimitri Svergun^b^ and Markus Glatzel^a^

^a^Institute of Neuropathology, University Medical Center Hamburg-Eppendorf (UKE), Hamburg, Germany; ^b^EMBL European Molecular Biology Laboratory, Hamburg, Germany

**CONTACT** Mohsin Shafiq m.shafiq@uke.de

*These authors contributed equally to this work.

**ABSTRACT**

The cellular prion protein (PrP^C^), a neuronally enriched membrane protein, is shown to impart a dual role in the pathological progression of Alzheimer’s disease. PrP^C^ molecules present on the cell surface serve as receptors for Aβ oligomers (Aβo) and initiate neurotoxic signalling events; in contrast, extracellularly shed and cleaved forms of PrP^C^ along with exosomally expressed PrP^C^ are suggested to accelerate the Aβ_42_ fibril formation by sequestering the Aβo thereby playing a neuroprotective role.

Aim of the current study is to investigate the influence of PrP^C^ expressing (WT) and PrP^C^ deficient (KO) exosomes on the Aβ_42_ oligomerization kinetics, utilizing small angle X-ray scattering (SAXS).

Experiments were designed for measuring the SAXS scattering profiles of various structural intermediates of Aβ_42_ along the different time points in aggregation cascade, in the presence of PrP^C^-WT and PrP^C^-KO exosomes.

Preliminary modelling of our data shows that the presence of both exosome variants (PrP^C^-KO and WT) leads to accelerated Aβ_42_ fibrillation. However, relatively higher levels of unbound in-solution Aβo were noticed in the presence of PrP-KO exosomes, indicating a decline in Aβ_42_ binding ability of PrP^C^-KO exosomes compared to that of PrP^C^-WT exosomes. We ascribe from our data that the presence of the PrP^C^ is crucial for the Aβ-sequestering activity in exosomes.

## 25. Aqueous extraction of formalin-fixed paraffin-embedded tissue and detection of prion disease using real-time quaking-induced conversion

Eric M. Nicholson, Trudy Tatum and Soyoun Hwang

United States Department of Agriculture, Agricultural Research Service, National Animal Disease Center, Ames, Iowa, USA

**CONTACT** Eric M. Nicholson eric.nicholson@ars.usda.gov

**ABSTRACT**

Formalin-fixed paraffin-embedded tissue (FFPET) is mainstay in the diagnosis of prion disease by immunohistochemistry (IHC), the standard by which all other TSE diagnostic protocols are judged. IHC offers advantages over diagnostic approaches that typically utilize fresh or frozen tissue with regard to sample preservation. However, IHC and the formalin-fixed tissues utilized for IHC do not typically couple well to amplification based approaches of prion disease detection. This limitation has the potential to hamper sensitivity of techniques compatible with FFPET as advances in amplification based techniques such as real-time quaking induced conversion (RT-QuIC) continue to enhance sensitivity. In this study, we apply an approach based on previous work utilizing FFPET and an aqueous extraction step for western blot and ELISA based detection of prion disease. FFPET from prion disease transmission studies of scrapie in sheep, chronic wasting disease in white-tailed deer or transmissible mink encephalopathy in cattle were cut at 5 µm thickness. Samples containing the tissue equivalent of as little as one 5 µm section can be used to readily discriminate positive from negative samples using RT-QuIC. The detection approach is avoiding the potential of a mixed chemical/biological waste stream that would occur if organic solvents were used in the removal of paraffin from the same, and offers the diagnostic and research community an addition in the diagnosis of TSEs.

## 26. Exploring the structure of mouse prion fibrils by site-directed spin-labelling and ESR spectroscopy

Kuan Yu Chu^a,b^, Chien Lun Hung^c^, Yun-Wei Chiang^d^, Sunney I. Chan^a,e^ and Rita P.-Y. Chen^b,d^

^a^Department of Chemistry, College of Science, Nation Taiwan University, Taipei, Taiwan; ^b^Institute of Biological Chemistry, Academia Sinica, Taipei, Taiwan; ^c^Department of Chemistry, Nation Tsing Hua University, Taipei, Taiwan; ^d^Institute of Biochemical Sciences, National Taiwan University, Taipei, Taiwan; ^e^Institute of Chemistry, Academia Sinica, Taipei, Taiwan

**CONTACT** Kuan Yu Chu brettchu007@gmail.com

**ABSTRACT**

**Introduction:** Recombinant prion protein (PrP) is a 23 kDa soluble protein without GPI anchor and N-glycosylation. The structure of the N-terminal domain is disordered and the structure of the C-terminal domain contains three α-helices, two β-sheets and one disulphide bond connecting helix 2 and 3. To study the structural conversion mechanism of full-length mouse prion protein (**mPrP**), we explore which segments of the protein are involved in this conformational transition thus form the cross-β structure.

**Methods**: Structure conversion of mPrP was studied using Electron Spin Resonance Spectroscopy (ESR) and Site-Directed Spin Labelling (SDSL). Two intrinsic Cys of mPrP were mutated to Ala. At the selected positions, the residue(s) were replaced by Cys and labelled with MTSSL. The spin-labelled PrPs were seeded by WT PrP fibrils to form various spin-labelled fibrils. The spin-diluted fibrils were prepared by mixing the spin-labelled PrP with WT PrP.

**Results**: ESR results of N174R1/N181R1, N181R1/Q186R1, and T193R1 fibrils showed that the whole helix 2 was converted into several β-strands and acted as part of the amyloid core by forming cross-β structure. For helix 3, not the whole helix 3 is transformed into cross-β structure during fibril formation. The local region around residue 212 might maintain its helicity and connect two intermolecular β-sheets. The 217–224 region formed a β-strand and are involved in the cross-β structure

**Conclusions**: Part of helix 1 of mPrP was unfolded during fibril formation. However, helix 2 and part of helix 3 of mPrP were involved in conformational conversion and formed the cross-β structure. The N-terminal part of helix 3 remained its helical structure.

## 27. Biodegradation of BSE prions in compost

Tim A. McAllister^a^, Shanwei Xu^b^, Sujeema Abeysekara^a,c^, Sandor Dudas^c^, Gordon Mitchell^d^ and Stefanie Czub^c^

^a^Agriculture and Agri-Food Canada, Lethbridge Research and Development Centre, Lethbridge, Alberta, Canada; ^b^Alberta Agriculture and Forestry, Lethbridge, Alberta, Canada; ^c^Canadian and OIE Reference Laboratories for BSE, Canadian Food Inspection Agency Lethbridge Laboratory, Lethbridge, Alberta, Canada; ^d^National and OIE Reference Laboratory for Scrapie and CWD, Canadian Food Inspection Agency, Ottawa, Ontario, Canada

**CONTACT** Tim A. McAllister tim.mcallister@canada.ca

**ABSTRACT**

To reduce the transmission of PrP^BSE^, specified risk material (SRM) that potentially contain PrP^BSE^ is required to be removed from the food and feed chains and disposed of via rendering and landfilling or incineration in North America. However, composting could be a more cost effective means of disposing of SRM and be more suitable in areas where SRM pickup services or suitable landfills are unavailable. Our study investigated the degradation of PrP^BSE^ (classical BSE) in lab-scale composters over 28 days and in stimulated field-scale composters over 106 days using protein misfolding cyclic amplification (PMCA). Laboratory-scale composting was conducted using 45 kg of feedlot manure with and without feathers. Compost was mixed at day 14 to generate a second heating cycle, with temperatures ≥55°C occurred for 2 days in the first cycle and less than 1 day in the second cycle. Based on PMCA, PrP^BSE^ was reduced by 1 log in compost with and without feathers after 14 days. After 28 days, PrP^BSE^ was reduced by 3 logs and between 3–4 logs in compost with and without feathers, respectively. For stimulated field-scale composting, compost piles were constructed using 2,200 kg feedlot manure and mixed at days 44 and 78 to generate three heating cycles. Temperatures ≥55°C were achieved for 67 out of 106 days of composting. However, after 106 days composting reduced PrP^BSE^ by only 1–2 logs. Our findings showed that enrichment for proteolytic microorganisms through the addition of feather keratin to compost enhances the degradation of PrP^BSE^. However, other factors such as varying concentrations and strains of PrP^BSE^ used and nature of proteolytic microbial populations in compost may cause differential results in the degradation of PrP^BSE^ in lab and field-scale composting systems.

## 28. Optimization of RT-QuIC for detection of seeding activity in preclinical blood samples from prion-infected sheep

Charlotte M. Thomas, Sandra McCutcheon, Richard A. Blanco, Boon Chin Tan and Fiona Houston

The Roslin Institute, R(D)SVS University of Edinburgh, Easter Bush, Edinburgh, Scotland, UK

**CONTACT** Charlotte M. Thomas Charlotte.Thomas@ed.ac.uk

**ABSTRACT**

Recent large-scale surveys of appendix samples estimate that as many as 1 in 2000 people in the UK have abnormal accumulation of disease-associated PrP, suggesting that they may be subclinically infected with variant Creutzfeldt-Jakob disease (vCJD) [1]. If these individuals are infectious, they pose a potential risk to others through blood and organ donation, or contamination of surgical instruments. Indeed, several cases of vCJD have been attributed to receiving blood products from individuals who donated blood during the preclinical stage of the disease [2]. Thus, a robust diagnostic test for vCJD is urgently required to detect these ‘silent’ (preclinical/subclinical) infections, in order to protect the UK blood supply.

Efforts to develop such a test are complicated by the extremely low concentrations of PrP^Sc^ present in readily accessible biological samples, such as blood, and this value is likely to be even lower in preclinical samples. Recently, several platforms have been developed which may be sensitive enough to overcome this challenge. One such technique, an ultrasensitive *in vitro* prion amplification assay, termed ‘real-time quaking induced conversion’ (RT-QuIC), has already proven to be a highly effective tool in the diagnosis of sporadic Creutzfeldt-Jakob disease (sCJD), when applied to samples of cerebrospinal fluid. [3] However, attempts to develop RT-QuIC into a blood-based diagnostic test for vCJD have been less successful.

Here we present a novel version of the RT-QuIC reaction, using a previously untested recombinant prion protein (recPrP) substrate. Due to a paucity of suitable human samples (which are necessary to optimize the platform and evaluate its performance in detecting preclinical infection), we have optimized our assay using an animal model of vCJD infection – sheep that have been experimentally infected with BSE. We show that our version of the RT-QuIC assay gives positive amplification results when applied to whole blood samples spiked with BSE-infected sheep brain homogenate. Positive samples are amplified within 20–40 h, with a high degree of analytical sensitivity, equivalent to a 10^−6^ dilution of BSE-infected brain homogenate. We then assess the performance of the assay on ‘endogenously’ infected whole blood samples, collected from BSE-infected sheep at regular time points, from the time of infection to the onset of clinical signs. If this version of the RT-QuIC reaction demonstrates high levels of sensitivity and specificity in identifying blood samples from pre-clinically infected individuals, it could then be adapted for use in humans.

### 

References[1]GillO, SpencerY, Richard-LoendtA, et al.
Prevalent abnormal prion protein in human appendixes after bovine spongiform encephalopathy epizootic: large scale survey. BMJ. 2013;347:f5675.2412905910.1136/bmj.f5675PMC3805509[2]PedenAH, HeadMW, RitchieDL, et al.
Preclinical vCJD after blood transfusion in a PRNP codon 129 heterozygous patient. Lancet
2004;364:527–529.1530219610.1016/S0140-6736(04)16811-6[3]McGuireLI, PedenAH, OrrúCD, et al.
Real time quaking-induced conversion analysis of cerebrospinal fluid in sporadic Creutzfeldt-Jakob disease. Ann Neurol. 2012;72(2):278–285.2292685810.1002/ana.23589PMC3458796

## 29. High efficiency detection of all prion subtypes of sporadic Creutzfeldt-Jakob disease by PMCA

Adam Lyon^a^, Sandra Pritzkow^b^, Silvio Notari^b^, Brian Appleby^b^, Pierluigi Gambetti^b^ and Claudio Soto^a^

^a^From the Mitchell Center for Alzheimer’s Disease and Related Brain Disorders, Department of Neurology, University of Texas Houston Medical School, Houston, Texas, USA; ^b^National Prion Disease Pathology Surveillance Center, Case Western University School of Medicine, Cleveland, OH, USA

**ABSTRACT**

Sporadic Creutzfeldt-Jakob disease (sCJD) is a fatal neurodegenerative disorder representing 85% of human prion diseases. It occurs at a rate of approximately one case per 1,000,000 people per year and its etiology is unknown. There are at least five subtypes of sCJD classified by the methionine (M)/valine (V) polymorphism at codon 129 and by the electrophoretic mobility of the protease-resistant core fragment of PrP^Sc^. The unglycosylated band of PrP^Sc^ type 1 is ~21 kDa, and that of PrP^Sc^ type 2 is ~19 kDa. The molecular classification aligns with distinct clinical and pathological features. While all subtypes remain invariably fatal, early and accurate diagnosis of sCJD and other human prion diseases is important for patients and their families, as well as for health care providers. Detection by real-time quaking induced conversion (RT-QuIC) of sCJD prions in CSF and from nasal brushings has demonstrated high sensitivity and specificity, but the challenge remains to detect sCJD prions in fluids and tissues whose collection is less invasive. Previously, we have shown successful detection of variant CJD prions in blood and urine by protein misfolding cyclic amplification (PMCA). The main goal of this study was to adapt the PMCA assay for high sensitive detection of all forms of sCJD to allow type identification of sCJD PrPSc in patients’ blood and urine. Our results demonstrate efficient amplification of all sCJD subtypes by PMCA in brain homogenate, with a variable rate depending on the specific strain. Indeed, we can reproducibly detect at least a 10^−7^–10^−11^ dilution of brain homogenate from all samples analysed. Currently, we are analysing blood and urine samples from patients with sCJD with diverse genotypes. This is a crucial step towards implementing PMCA as a general tool for routine diagnosis of sCJD including sCJD subtype, and to screen blood to increase the safety of the blood supply.

**Funding**

SB1NS079060, R01 NS083687, P01 AI106705 and Charles S. Britton Fund (PG).

## 30. A blue light optogenetic and CRISPR/Cas9 system for inducible PrP^C^ expression

Luis A. Arce^a,b^, Serene Wohlgemuth^a^ and David Westaway^a,b^

^a^Centre for Prions and Protein Folding Diseases; ^b^Department of Biochemistry, University of Alberta, Edmonton, Alberta, Canada

**CONTACT** Luis A. Arce larce@ualberta.ca

**ABSTRACT**

An inducible system for expression of wild type PrP^C^ can offer advantages for investigating the chemical biology of this GPI-linked cell-surface protein. Previous inducible PrP^C^ systems have utilized non-physiological xenobiotic compounds such as tetracycline to control gene expression [1,2]. However, wishing to avoid off-target chemical effects of xenobiotic additives, we are developing an inducible system controlled by light. CRISPR/Cas9 systems that activate transcription (CRISPRa) utilize transcriptional activation domains (TAD), as found in VP64 or p65 proteins, attached to a modified Cas9 (called dCas9) [3]. dCas9 has inactivated endonuclease function but can still be directed to a specific genomic locus by a ‘guide RNA’ (gRNA); previous studies have exploited this interaction by the development of dCas9/TAD fusion proteins that allow for the activation and overexpression of targeted genes [4]. Inducible CRISPR-based systems have been developed typically using a split-Cas9 method and small effector molecules, such as rapamycin. However, these systems can also exhibit leakiness [5]. An alternative system which utilizes the light inducible protein interactions between cryptochrome 2 (CRY2) and CIB1 from *Arabidopsis thaliana* has proven to be effective in both gene induction and mitigation of residual transcriptional activity [6,7]. The system contains two fusion proteins; the ‘anchor’ (dCas9/CIB1), and the ‘activator’ (CRY2/TAD); targeting of dCas9 is achieved by the gRNA and then heterodimerization of CRY2 and CIB1 is based upon blue light irradiation. Here we describe adaptation of this optogenetic system to modify transcription initiated from the human *Prnp* promoter region. Following a gRNA design phase, corresponding sequences were inserted into an expression vector. Serial transfections were used to deliver the two fusion proteins plasmids and designed gRNA plasmids into the human HAP1 cell line. Transfected cells are subjected to blue light irradiation and then assessed for PrP^C^ levels using quantitative capillary westerns. This inducible system may offer advantages for drug screening to identify novel chemicals that modify PrP^C^ biology.

**KEYWORDS:** CRISPR Cas9; optogenetics; PrP^C^; drug screen

### 

References[1]HuangS., et al.
PNAS
2007;104(16):6800–6805.10.1073/pnas.0608885104PMC187186517420473[2]SabuncuE., et al.
Biochem Biophys Res Commun
2005;337(3):791–798.10.1016/j.bbrc.2005.09.11416214113[3]GilbertL. A., et al. Cell
2013;154(2):442–451.10.1016/j.cell.2013.06.044PMC377014523849981[4]TanenbaumM. E., et al. Cell
2014; 159(3):635–64610.1016/j.cell.2014.09.039PMC425260825307933[5]ZetscheB., et al.
Nat Biotechnol
2015;33(2):139–141.10.1038/nbt.3149PMC450346825643054[6]KennedyM. J., et al.
Nat Methods
2010;7(12):973–975.10.1038/nmeth.1524PMC305913321037589[7]NihongakiY., et al.
Chem Biol
2015;22(2):169–174.10.1016/j.chembiol.2014.12.01125619936

## 31. Exploring the role of PrP in axonal tract formation during zebrafish nervous system development

Fabrizio Cuevas^a,b^, Ignacio Gutierrez^a,b^, Carmen G. Lemus^a,b^ and Miguel L. Concha^a,b,c^

^a^Anatomy and Developmental Biology, Institute of Biomedical Sciences, Faculty of Medicine, Universidad de Chile, Santiago, Chile; ^b^Biomedical Neuroscience Institute, Santiago, Chile; ^c^Center for Geroscience, Brain Health and Metabolism, Santiago, Chile

**CONTACT** Fabrizio Cuevas fabriziocuevas@gmail.com

**ABSTRACT**

Although the role of PrPc in neurite outgrowth, neuronal differentiation and synapse formation has been described *in vitro*, little is known of this phenomenon *in vivo* and especially during development. Zebrafish (Danio rerio) has emerged as a promising model to study PrP physiological function because of its external development, embryo transparency and amenability to genetic manipulation. The developmental expression of genes encoding for the zebrafish PrP paralogs prp-1 and prp-2 is still controversial thus we first revisited in detail the spatiotemporal expression of both genes by *in situ* hybridization. prp2 is expressed from 18 h post fertilization (hpf) in the encephalon and the first segments of the spinal cord, and also in structures outside the nervous system. In contrast, prp1 is detectable in the central nervous system only from 48 hpf, and in cranial ganglia from 60 hpf. These results are consistent with a previous report [1], but differ in the lack of expression seen at earlier stages. To begin exploring the function of PrP in axonal tract formation we perfomed acetylated tubulin by indirect immunofluorescence in mutant embryos and larvae lacking *prp-1* and *prp-2* function. We found that prp-1 and prp-2 mutants both show weaker intensity of anti-acetylated tubulin staining and less axonal arborization in some specific brain regions, like the olfactory bulb and cranial ganglia, and thinner tracts at the level of the cerebellar commissure. The presence of abnormal tracts in prp-1 and prp-2 mutants is intriguing given the normal appearance of embryos and larvae. We are currently dissecting the axonal tract deficiencies using fluorescent reporters in prp-1 and prp-2 mutants.

**Funding**

CONICYT PFCHA/DOCTORADO BECAS CHILE/2017–21,171,130, ICM P09-015-F and Fondap 15,150,012.

### 

Reference[1]CottoE, AndreM, ForgueJ, et al.
Molecular characterization, phylogenetic relationships, and developmental expression patterns of prion genes in zebrafish (Danio rerio). Febs J. 2005;272(2):500–13.1565488810.1111/j.1742-4658.2004.04492.x

## 32. The EV-export pathway for misfolded proteins

Desmond Pink^a^, John Lewis^a,b^ and Janice E.A. Braun^c^

^a^Nanostics Precision Health, Edmonton, Alberta, Canada; ^b^Hotchkiss Brain Institute, Department of Biochemistry and Molecular Biology, Cumming School of Medicine, University of Calgary, Calgary, Alberta, Canada; ^c^Department of Oncology, University of Alberta, Edmonton, Alberta, Canada

**CONTACT** Janice E.A. Braun braunj@ucalgary.ca

**ABSTRACT**

Extracellular vesicles (EVs) are a collection of secreted vesicles of diverse size and cargo that are implicated in the physiological removal of nonfunctional proteins as well as the cell-to-cell transmission of disease-causing-proteins in several neurodegenerative diseases. We have shown that the molecular chaperone, cysteine string protein (CSPα; DnaJC5), is responsible for the export of disease-causing-misfolded proteins from neurons via EVs. We report here that CSPα-EVs efficiently deliver GFP-tagged 72Q huntingtin^exon1^ to naive neurons. When we analysed the heterogeneous EV pool, we found that the misfolded GFP-tagged 72Q huntingtin^exon1^ cargo was primarily found in EVs between 180–240nm. We further determined that cargo-loading of GFP-tagged 72Q huntingtin^exon1^ into EVs was impaired by resveratrol. In addition to CSPα, we identified two other J protein co-chaperones, DnaJB2 and DnaJB6 that facilitate EV export of GFP-tagged 72Q huntingtin^exon1^. While human mutations in CSPα cause the neurodegenerative disorder, adult neuronal ceroid lipofuscinosis, mutations in DnaJB6 cause limb-girdle muscular dystrophy and mutations in DnaJB2 are linked to the neurodegenerative disorders, Charcot Marie Tooth disease, distal hereditary motor neuropathy, spinal muscular atrophy and juvenile Parkinsonism. Our data provides new insights into the parallels between proteostasis and EV export, as three J proteins linked to disease in humans are the same as those that mediate EV genesis and export of misfolded proteins.

## 33. Detection of CWD prions in third eyelids of deer and elk

Sarah K. Cooper^a^, Clare E. Hoover^a,b^, Davin M. Henderson^a^, Nathaniel D. Denkers^a^, Candace K. Mathiason^a^ and Edward A. Hoover^a^

^a^Prion Research Center, Department of Microbiology, Immunology, and Pathology, College of Veterinary Medicine and Biomedical Sciences, Colorado State University, Fort Collins, CO, USA; ^b^AstraZeneca, Waltham, NJ, USA

**CONTACT** Sarah K. Cooper scooper2@rams.colostate.edu

**ABSTRACT**

**Background**: The increasing prevalence of CWD globally, makes critical the development of fast, cost-effective methods to detect the disease in hunter-harvested deer and assist wildlife population disease management. Based in part on the demonstration of PrPSc in the third eyelid lymphoid follicle in sheep with scrapie, we explored the third eyelid for its potential as a non-invasive and easily accessible lymphoid tissue that could be sampled without special anatomical training antemortem and postmortem for PrP^CWD^ detection by real-time quaking induced conversion (RT-QuIC) and immunohistochemistry (IHC).

**Methods**: We compared RT-QuIC detection sensitivity in the third eyelid to the retropharyngeal lymph node and obex region of the brain from experimentally CWD inoculated white-tailed deer and naturally occurring subclinical elk using IHC. We examined symptomatic and asymptomatic white-tailed deer inoculated with CWD(+) and CWD(-) brain homogenate or saliva via the *per os* or aerosol route. In addition, we examined samples from asymptomatic, naturally exposed elk which were culled due to being RAMALT biopsy positive or suspect by RT-QuIC.

**Results**: We identified prion seeding activity in third eyelids in 24 out of 25 (96%) samples from terminal, experimentally CWD-infected deer and found RT-QuIC positivity in the third eyelid as early as 1 month after experimental exposure to CWD. In addition, we identified prion seeding activity in third eyelids in 17 out of 25 (68%) samples asymptomatic, naturally exposed elk. By contrast, IHC detected prion deposition in third eyelid lymphoid follicles in 5 of 10 deer (50%). We show that RT-QuIC can be more sensitive for CWD prion detection in the third eyelid compared to IHC.

**Conclusions**: RT-QuIC testing on the third eyelid is a rapid and sensitive means for post-mortem detection of CWD with potential to augment CWD diagnostics and hunter testing compliance.

**Funding**

Supported by NIH R01-NS-061902, P01-AI-077774, F30-ODO-118,143, T32-OD0-10,437

## 34. microRNAs profiling in patient bloods identifies a novel signature associated with sCJD disease progression

Penny Norsworthy, John Collinge, Simon Mead and Emmanuelle Viré

MRC Prion Unit at UCL, Institute of Prion Diseases, Queen Square House, Queen Square, University College London, London, UK

**CONTACT** Emmanuelle Viré e.vire@ucl.ac.uk

**ABSTRACT**

Prion diseases are fatal neurodegenerative diseases of humans and animals caused by the misfolding and aggregation of prion protein (PrP). Misfolded proteins play an important role in many forms of dementia but despite intensive efforts to better understand the pathophysiology of these diseases, the mechanisms involved are still poorly understood. Although neurodegeneration is affecting millions of people worldwide there is a huge unmet need for novel diagnostic and treatment approaches in neurodegenerative disorders to counter therapeutic nihilism in this area. Recent studies have implicated a role for genetic and epigenetic variation in neurodegenerative disorders. To date, however, no study has systematically explored the contribution of epigenetic changes in human prion diseases.

microRNAs (miRNAs) play crucial role in human diseases, and the discovery of the presence of cell-free miRNAs in the blood stream probed researchers to investigate the role of miRNAs as potential non-invasive biomarkers.

We performed genome-wide microRNA deep sequencing on 107 blood samples from patients with sporadic CJD and healthy controls and identified five differentially expressed microRNAs. We confirmed that this miRNA signature is specific to sporadic CJD since the level of those 5 miRNA remained unchanged in other neurodegenerative disorders (CJD and Alzheimer’s diseases). Investigating longitudinal samples from more than 20 patients with sCJD, we found that the miRNA signature strongly correlates with disease severity. These results have been replicated using both an independent cohort and qPCR technology.

Integrating our data, we have established a sCJD specific signature, with genes between the two groups being enriched in functions related to neuroinflammation and immunity; and have correlated it with age of onset, disease progression, severity, decline, and genotype.

In light of our findings, we will discuss the potential implications for this novel gene signature to complement existing non-invasive biomarkers and its relevance for disease management.

## 35. Process of structural diversification and synergy between prion subassemblies during host adaptation

Angélique Igel-Egalon^a^, Mohammed Moudjou^a^, Florent Laferrière^a^, Philippe Tixador^a^, Jan Bohl^a^, Mathieu Mezache^a,b^, Tina Knäpple^a^, Laetitia Herzog^a^, Fabienne Reine^a^, Marie Doumic^b^, Vincent Béringue^a^ and Human Rezaei^a^

^a^VIM, INRA, Université Paris-Saclay, Jouy-en-Josas, France; ^b^INRIA, MAMBA, Université Paris VI, Paris, France

**CONTACT** Human Rezaei human.rezaei@inra.fr

**ABSTRACT**

In prion diseases, the cellular prion protein PrPC can misfold into PrPSc and auto-organize into conformationally distinct assemblies or strains. A plethora of observations reports the existence of PrPSc structural heterogeneity within prion strains, suggesting the emergence and coevolution of structurally distinct PrPSc subassemblies during prion replication in controlled environment. However, the nature of and the underlying processes of the structural diversification remain poorly explored and mainly based on conjectural observations. Conceptually, such structural heterogeneity appears central to prion host adaptation since the actual paradigm consists mostly on the best replicator selection, i.e. selection of the best-fit subset of PrPSc assemblies within the PrPSc structural cloud.

Here, we first characterized the evolution of the PrPSc quaternary structure in early versus late stage of prion replication (in a homotypic PrP context) *in vivo* and in bona fide cell-free amplification assays. Regardless of the strain studied, the early replication stage conduced to the preferential formation of small PrPSc oligomers, thus highlighting a quaternary structural convergence phenomenon. The evolution of these small oligomeric assemblies led to the formation of structurally different multimers through a secondary templating pathway in an autocatalytic and PrPC-dependent manner. Second, we addressed the role of such structural PrPSc diversification in prion adaptation on cross-species transmission. We isolated by size-fractionation or by dilution the formed PrPSc assemblies and measured their relative propensity to cross an existing species barrier, as modelled by transgenic mice expressing heterotypic PrPC. We showed impaired and aberrant fitness of the isolated PrPSc subassemblies as compared to the unfractionated and undiluted populations, respectively.

Altogether our observations uncover the existence of two mechanistic processes during prion replication: first, prion structural diversification occurs through a secondary templating pathway. Second, overcoming species barriers requires complementation between the PrPSc subassemblies. These processes explain how structurally diverse assemblies can coexist despite competition for the same PrPC substrate and truly challenge the best- replicator selection models.

## 36. Structural characterization of amyloidogenic proteins implicated in neurodegeneration under conditions of intracellular crowding and in non-polar environments

Nikolay Blinov^a,b^, David S. Wishart^c^, Neil R. Cashman^d^, and Andriy Kovalenko^a,b^

^a^University of Alberta, Department of Mechanical Engineering, Edmonton, Canada; ^b^NRC Nanotechnology Research Centre, Edmonton, Canada; ^c^University of Alberta, Departments of Biological Sciences and Computing Science, Edmonton, Canada; ^d^University of British Columbia, Department of Medicine, Vancouver, Canada

**CONTACT** Nikolay Blinov nblinov@ualberta.ca

**ABSTRACT**

Misfolding and aggregation of proteins is a key pathological event in many neurodegenerative disorders, including the prion diseases, Alzheimer’s disease (AD), and Amyotrophic Lateral Sclerosis. Structural properties of proteins and their aggregates (oligomers and amyloid fibrils) are essentially affected by their solvation environments, the presence of metabolites and salt ions. Structural characterization of oligomers which may be the most toxic agents in neurodegeneration is a very important task because the inhibition of formation of these aggregates and their pathological action has been proposed as a therapeutic target to fight neurodegeneration.

Experimental structural studies are complicated by the transient nature of oligomers. In the case of AD, amyloid (A)β peptides (constituent units of the neurotoxic oligomers responsible for neurotoxicity in AD) are intrinsically disordered, which introduces another level of complexity to the problem. Despite the significant efforts, there is no consensus on structure and aggregation pathways of Aβ peptides in cellular environments. The situation is further complicated by the fact that the peptides can aggregate and perform their neurotoxic activity intracellularly. The intracellular Aβ species can also be secreted into the extracellular space where they may trigger prion-like pathological misfolding.

In this poster, we provide an overview of our recent efforts towards structural characterization of the amyloidogenic proteins and their aggregates under different solvent environments. The new methodology has been developed based on the 3D-RISM-KH molecular theory of solvation to account for the effects of specific conditions of intra and extracellular environments on the conformational equlibrium of intrinsically disordered peptides. Applied to AD, it shows the similarities and differences between conformational properties of Aβ peptides under conditions of intracellular crowding and in non-polar environments (such as imposed by the neuronal lipid membrane). Specifically, both the crowding and the non-polar solvation make the peptides less disordered, which may result in the increase of their propensity for aggregation. Among the differences are the opposite trends in bend and turn secondary structure contents under different conditions. We also show that the 3D-RISM-KH based methodology could be used to predict effects of synaptic metabolites and other small molecules (e.g. therapeutic agents) on the structure of the peptides. This feature of the methodology can be used to screen potential inhibitors of pathological misfolding of proteins in neurodegeneration.

## 37. Spontaneous neurodegeneration in transgenic mice caused by histidine to tyrosine substitution at non-OR copper-binding site

Alba Marín-Moreno^a*^, Thanh Hoa Tran^b*^, Juan Carlos Espinosa^a^, Edoardo Bistaffa^c^, Fabio Moda^c^, Juan María Torres^a^ and Giuseppe Legname^b^

^a^Centro de Investigación en Sanidad Animal (CISA-INIA), Madrid, Spain; ^b^Laboratory of Prion Biology, Department of Neuroscience, Scuola Internazionale Superiore di Studi Avanzati, Trieste, Italy; ^c^Fondazione IRCCS Istituto Neurologico Carlo Besta, Milan, Italy

**CONTACT** Alba Marín-Moreno marin.alba@inia.es

*These authors contributed equally to the work

**ABSTRACT**

Prion protein has long been known as a copper binding protein via histidine residues in the octapeptide repeats (OR) and the non-OR region located in the disordered N-terminal tail of the protein. The functional implication of copper binding to PrP is not yet clear, but copper seems to play an important factor in prion disease.

Transgenic mice expressing the mouse prion protein replacing histidine (H) by tyrosine (Y) at codon 95 to disrupt the non-OR copper-binding site have been generated.

Mice overexpressing approximately two fold PrP H95Y showed clinical signs and died at about 100 days with spongiform degeneration and atypical PK-resistant PrP in the brain. Inoculation of brain homogenate from terminally ill mice overexpressing PrP H95Y to mice expressing a low level of PrP H95Y (1 fold) or Tga20 mice also causes lethal, spongiform encephalopathy.

H95Y substitution at non-OR region could promote PrP^C^-PrP^Sc^ conversion and induce spontaneous neurodegeneration disease in transgenic mice, implying a critical role for the non-OR copper binding site in prion disease.

**KEYWORDS**

Scrapie; prion strain; transmission barrier; mouse bioassay

**Funding**

Spanish Ministerio de Ciencia, Innovación y Universidades (AGL2016-78,054R).

## 38. Signalling pathways underlying prion neurotoxicity

Nhat T. Le, Robert C. Mercer and David A. Harris

Department of Biochemistry, Boston University School of Medicine, Boston MA, United States of America

**ABSTRACT**

The process by which prions propagate is now well understood; however, the molecular mechanism responsible for their neurotoxicity remains unclear. Synaptic loss is one of the earliest events in both *in vivo* and *in vitro* models of prion disease. Recently, we have developed sophisticated neuronal cell culture models to analyse the mechanisms of prion-induced synaptic degeneration in a more physiologically relevant setting. Using this system to dissect the underlying cellular and molecular mechanisms, we show that exposure of hippocampal neurons to PrP^Sc^ results in rapid, PrP^C^-dependent retraction of dendritic spines. In this process, PrP^Sc^ activation of AMPA and NMDA receptors induces calcium influx, which subsequently activates p38 MAPK and its downstream MK2/3 kinases. Pharmacologically and genetically reducing p38 MAPK activity not only blocks PrP^Sc^-induced loss of dendritic spines, but also reverses dendritic spine loss that has already occurred. In order to identify additional components of prion-induced synaptotoxic pathways, we measured changes in the phosphoproteome and transcriptome of cultured neurons exposed to PrP^Sc^ while they are undergoing well-defined alterations in synaptic structure and function. This work identifies a new, druggable target that could be used to block synaptotoxic sequelae in prion diseases: p38 MAPK. We are in the process of testing whether reducing p38 MAPK function, either genetically or pharmacologically, ameliorates the symptoms of prion disease in mice.

## 39. Generation of bona fide prions by large-scale Protein Misfolding Cyclic Amplification

Fei Wang, Luis Concha-Marambio, Enrique Armijo and Claudio Soto

Mitchell Center for Alzheimer’s Disease and Related Brain Disorders, Department of Neurology, McGovern Medical School, University of Texas Health Science Center at Houston, Houston, Texas, USA

**CONTACT** Fei Wang Fei.Wang.1@uth.tmc.edu

**ABSTRACT**

The misfolding of normal prion protein (PrP^C^) into pathogenic PrP^Sc^ is the key event causing prion diseases. However, the molecular mechanism underlying such conformational conversion remains elusive, largely due to the absence of high-resolution structural information of PrP^Sc^, hence impeding the developments of efficacious therapeutic strategies against these currently incurable diseases. To facilitate the structural studies of PrP^Sc^, here we present our latest results of large-scale production of bona fide prions by Protein Misfolding Cyclic Amplification (PMCA), which not only provides the platform for ultra-sensitive detection of prions in biological samples, but can also faithfully reproduce the key properties of prions, including infectivity, species barrier, and strain variability. In a pilot study, normal mouse brain homogenates were seeded with RML prion in large containers to produce bona fide prions by PMCA in reaction volumes ranging from 30 mL to 120 mL. The prion yielded in this large-scale production maintained the RML prion strain characteristics in terms of infectivity in wild type animals, incubation time, PrP^Sc^ biochemical profile and pathological changes. In the follow-up study, recombinant prion generated from bacterially expressed full-length recombinant mouse PrP seeded with RML prion was used to seed the large-scale production of recombinant PrP^Sc^ by PMCA. The large-scale PMCA (LS-PMCA) platform is capable of producing at least 2 mg of PK-resistant recombinant PrP^Sc^ in a 200-mL reaction in 24 h and has the option to scale up to as much as 1000 mL per round of LS-PMCA. The infectivity assessment of the recombinant PrP^Sc^ from LS-PMCA is currently ongoing. Availability of a procedure to produce massive amounts of PrP^Sc^ using recombinant protein as substrate which maintain the typical biological and biochemical properties of bona fide prions, will facilitate structural and biochemical studies of infectious prions.

## 40. Impact of different recombinant PrP substrates on the signalling response in the RT-QuIC

Susana Correia, Matthias Schmitz and Inga Zerr

Department of Neurology, Medical University of Goettingen, Goettingen, Germany

**CONTACT** Susana Correia Susana.correia@med.uni-goettingen.de

**ABSTRACT**

Transmissible spongiform encephalopathies (TSE) are a group of neurodegenerative disorders, such as Creutzfeldt-Jakob disease (CJD), which are based on the conformational change of the prion protein (PrP) from the cellular form, PrPC, to the infectious scrapie form, PrPSc.

Based on the mechanism that PrPSc acts as a template for the conversion of PrPC, protein misfolding amplification assays, such as the real-time quaking-induced conversion (RT-QuIC) has been applied for the detection of misfolded PrPSc in the brain and cerebrospinal fluid (CSF) for the pre-mortem diagnosis of human prion diseases.

The technique bears a huge diagnostic and analytical potential. However the clinical variability of different sCJD subtypes (MM1, MM2, MV1, MV2, VV1, and VV2) has a relevant impact on the accuracy of diagnostic assays. An additional important factor is the kind of recombinant PrP substrate (e.g. hamster, hamster-sheep chimera, bank vole, etc.) used in the RT-QuIC. The diagnostic accuracy for different sCJD subtypes, familial forms – or the variant form of CJD is variable, e.g. only a very few recombinant PrP substrates are able to detect variant CJD prions, such as bank vole PrP.

In our study, we aim to increase the diagnostic accuracy and the application spectrum of the RT-QuIC for all types of prion diseases, in particular for the atypical and rare forms like sCJD (MM2T, MM2C, and VV1), FFI or GSS. We will produce and purify a panel of different recombinant PrP substrates and compared the sensitivity and specificity among them across the wide spectrum of prion diseases to reach an optimized diagnostic accuracy.

## 41. A screen for prion and α -synuclein aggregate decontaminants to enhance safety of surgical instrument reprocessing

Daniel Heinzer^a^, Merve Avar^a^, Manuela Pfammatter^a^, Rita Moos^a^, Linda Irpinio^a^, Dezirae Schneider^a^, Benjamin Kuhn^b^, Stefan Mauerhofer^b^, Urs Rosenberg^b^, Adriano Aguzzi^a^, Simone Hornemann^a^

^a^Institute of Neuropathology, University of Zurich, Zurich, Switzerland; ^b^Borer Chemie AG, Zuchwil, Switzerland

**CONTACT** Simone Hornemann simone.hornemann@usz.ch

**ABSTRACT**

Prions, the infectious agents causing prion diseases such as the Creutzfeldt-Jakob disease, show extreme robustness to common decontamination procedures. Therefore, there is a risk of iatrogenic transmission of these lethal diseases by inefficiently decontaminated medical instruments. So far, improvement of effective decontaminating agents applicable to routine sterilization procedures has not been feasible as the traditional assays for measuring prion infectivity are time-consuming and constrained by a limited throughput. The development of the real-time quaking induced conversion (RT-QuIC) assay [1], a fast and ultra-sensitive method, based on the self-propagation of proteinaceous agents in misfolding diseases, provides a new approach for the detection of infectious prions.

The adaption of this assay to a carrier mimicking surgical steel allows evaluating the efficiency of novel potential decontaminants in a fast and proficient way. In addition, with the emergence of evidence of α-synucleinopathies exhibiting prion-like infectious properties, this method was further adapted and applied to assess inactivation procedures for potentially hazardous α-synuclein fibrils. We demonstrated that the prion RT-QuIC assay is suitable to detect propagating prions adsorbed to 316L steel type metal beads, and that the treatment with the standard prion decontamination procedure (1 M NaOH for 2 h) completely abolished a positive signal in the assay. Around 120 different formulations were tested for their efficiency to inactivate or remove prions from the steel beads. We identified one potential candidate formulation with good material compatibility that strongly and reliably reduced prion propagation activity in the RT-QuIC assay. The candidate formulation and two further alkaline cleaner are currently validated in a cell and mouse bioassay to confirm their decontamination properties. In addition, we developed an α-synuclein RT-QuIC assay that we used to test the inactivation efficiencies of known prion decontaminants on α-synuclein preformed fibrils. Interestingly, some of the commonly used prion decontamination procedures were less effective to deactivate the propagation efficiency of α -synuclein preformed fibrils. This implies that the resistance of α -synuclein fibrils might differ or even exceed the one of bona fide prions, and that other decontamination procedures need to be applied for the effective deactivation of α-synuclein fibrils.

### 

Reference[1]WilhamJM, OrrúCD, BessenRA, et al.
Rapid end-point quantitation of prion seeding activity with sensitivity comparable to bioassays. PLoS Pathog. 2 December
2010;6(12):e1001217.10.1371/journal.ppat.1001217PMC299632521152012

## 42. Multiple sclerosis brain transmits pathology to humanized transgenic mice

Shigeki Tsutsui^a^, Shoraf Dadakhujaev^a^, Francisca Cavalcante Melo^a^, Geert Schenk^b^, Roel Klaver^b^, Hugo Tedford^a^, Najwa E Kadi^a^, Karen Cummins^a^, Gui Fang Guo^a^, Andrew V Caprariello^a^, Julianne Proft^a^, Shyamosree Roychoudhury^a^, Kyoichi Tsutsui^a^, V. Wee Yong^a^, Jeffrey T Joseph^a^, Gerald W. Zamponi^a^, Jeroen JG Geurts^b^ and Peter K Stys^a^

^a^University of Calgary, Calgary, *AB*, Canada; ^b^VU University Medical Center, Amsterdam, Netherlands

**ABSTRACT**

The fundamental aetiology of multiple sclerosis (MS) remains unknown. Two competing theories propose that on the one hand, MS is driven by a dysregulation of the peripheral immune system promoting inflammatory attacks on the CNS, while an alternative model posits that an initial primary degenerative process may secondarily trigger autoimmunity. Our hypothesis is that MS, like many neurodegenerative diseases, might be a protein misfolding disorder driven by accumulation of pathological cytotoxic aggregates that can spread through the CSF and CNS. One defining property of such diseases is the transmission of pathology to susceptible hosts.

We have previously shown that intracerebral inoculation (i.c.) of MS brain homogenates into human prion protein (PrP) over-expressor mice induced significant MS-like pathology, together with cognitive disability. We have expanded our dataset using i.c. transmission of MS pathologies in larger numbers of animals (53 mice from 10 primary or secondary-progressive MS patients, 65 mice from 9 control donors, 30 mice from 3 chronic encephalitis brains, and 10 mice from other neurological diseases, such as AD and Lewy body disease), using additional immunohistochemical injury markers, which further supports the underlying hypothesis. Passaging (inoculation of naïve mice with brain homogenate of MS-inoculated mice) also continued to transmit pathology. MS-injected mouse brains exhibited significant degeneration of myelin and axonal damage with microglial and astroglial activation in the corpus callosum, leukocortical junction, thalamus and peri-ventricular region, assessed by SMI-94, QD9, SMI-32, Iba-1 and GFAP immunohistochemistry, whereas mice injected with control human brain did not develop pathology. Importantly, PrP-immunodepleted MS brain homogenates, blunted transmission of pathology, further supporting a possible role of pathological PrP. Formic acid-resistant prion protein aggregates were detected in both white and grey matter regions. Protease resistance, often seen in more conventional prionopathies, was not detected in either human MS brain or MS-inoculated mice.

Conclusion: Our results are consistent with the hypothesis that MS might be a primary degenerative disorder caused by accumulation and propagation of atypical pathogenic PrP, that can transmit pathology to humanized murine hosts. We speculate that these toxic pathological conformers could circulate in the CSF and spread to various brain regions in contact with CSF, as is characteristic of human MS lesion distribution. The resulting degeneration of myelin and release of antigenic debris could secondarily trigger an autoimmune inflammatory response in immune-predisposed hosts. Together, such a convolution of a primary prion-dependent degeneration, with secondary inflammation could explain the broad spectrum of human MS phenotypes.

## 43. Molecular characterization of prion diseases in Denmark: the national reference center experience from 2002 to 2018

Aušrinė Areškevičiūtė^a^, Helle Broholm^a^, Linea C. Melchior^a^, Piero Parchi^b^, Anna Bartoletti-Stella^b^, Eva L. Lund^a^

^a^Danish Reference Center for Prion Diseases, Department of Pathology, Copenhagen University Hospital - Rigshospitalet, Copenhagen, Denmark; ^b^IRCCS Istituto delle Scienze Neurologiche di Bologna, Ospedale Bellaria, Bologna, Italy

**CONTACT** Aušrinė Areškevičiūtė ausrine.areskeviciute@regionh.dk

**ABSTRACT**

We present a Danish cohort of prion disease cases with well-characterized fresh frozen brain samples that provides a great foundation for future research.

The aim of this study was an up-to-date molecular characterization and classification of all fresh frozen prion disease samples delivered to the Danish Reference Center for Prion Diseases in the period of 2002 and 2018.

Molecular characterization and classification of these samples was based on an improved immunoblotting method and sequencing of the whole prion protein gene coding region. Immuno- and histopathological findings in the brain tissues were re-evaluates in several cases. Available medical records were gathered for estimation of patients’ age at disease onset and death, disease duration, and disease clinical presentation.

Thirty-seven of forty-one samples were classified as sporadic prion diseases with the following subtypes: MM/MV1 (*n* = 23), VV2 (*n* = 7), MM/MV1+2 (*n* = 3), MV2 (*n* = 2), MM2 (*n* = 2). A case of CJD with sporadic phenotype that occurred to a mother and wife of P102L mutation carriers was also a part of the Danish prion diseases cohort [1].

Four of forty-one cohort samples were found to harbor mutations in the prion protein gene. Two of those mutations were unique 5 and 8 octapeptide repeat insertions [2]. The other two mutations found in the cohort were a well-known P102L, and a novel T201S point mutations. The pathogenicity of T201S was recently investigated and described [3].

Re-evaluation of the cohort samples revealed that initial disease classification was inaccurate or missing in nearly every 5th case, which is a strong argument for consideration to re-evaluate older samples as techniques with higher sensitivity and specificity are being developed, and the classification system updated.

### 

References[1]Areškevičiūtė, et al.
JNEN
2018;77:673–684.10.1093/jnen/nly04329889261[2]Areškevičiūtė, et al. A novel eight octapeptide repeat insertion in
*PRNP* causing prion disease in a Danish family. JNEN;2019:In Press.10.1093/jnen/nlz03731107536[3]Mok, et al. Neurobiol Aging
2018;71:265e1–265.e7.

## 44. Understanding prion-like mechanisms in traumatic brain injury using a novel *in vivo* model

Hadeel Alyenbaawi^a,b^ and W. Ted Allison^a,b,c^

^a^Center for Prion & Protein Folding Diseases, University of Alberta, Edmonton AB, Canada; ^b^Department of Medical Genetics, University of Alberta, Edmonton AB, Canada; ^c^Department of Biological Sciences, University of Alberta, Edmonton *AB*, Canada

**CONTACT** Hadeel Alyenbaawi alyenbaa@ualberta.ca

**ABSTRACT**

**Background/Introduction**: Traumatic brain injury (TBI) is known as one of the risk factors leading to neurodegeneration and dementia, in particular chronic traumatic encephalopathy (CTE). CTE is one of the tauopathies characterized by a distinct accumulation of hyperphosphorylated tau. Similar to Alzheimer Disease (AD) and other tauopathies, pathology seems to progress to other regions during later stages of the disease, mimicking the spreading mechanisms of prion disease. The prion-like seeding and transmission of tau in CTE and TBI cases has begun to be illuminated by a limited number of *in vitro* and *in vivo* studies. However, there is still a lack of information regarding the mechanisms of prion-like spreading and cell uptake of tau seeds when it comes to TBI. We aimed to establish a novel TBI model using our novel transparent tau biosensor zebrafish that we engineered to detect and visualize tau seeds. There is much promise in establishing *in vivo* models to gain greater understanding of the mechanisms controlling the spreading of tau pathology in TBI patients.

**Material and Methods**: We successfully engineered a stable transgenic zebrafish line that expresses a tauopathy biosensor reporter protein that detects various forms of tau from two to four days post-transduction. We also optimized a simplified system to induce blast injury on the tauopathy biosensor zebrafish. Zebrafish subjected to blast injury were analysed for the formation of GFP+ puncta indicative of tau seeds in the brains and spinal cord at various time points. Additionally, we evaluated the impact of administering convulsant drugs as stress agents on the spreading and cell uptake of tau inclusions in both a dose- and time-dependent manner.

**Results**: The zebrafish subjected to blast injury exhibited seizure-like movement immediately following the injury. We could reliably detect GFP+ tau inclusions three to four days after subjecting the larvae to blast injury. The number of these tau inclusions was significantly higher in the blast injury group compared to the control group. Treatment with various concentrations of convulsant drugs affected the formation of those inclusions in a dose-dependent manner.

**Conclusions**: The engineering of an *in vivo* tau biosensor and TBI model in larvae zebrafish will uncover information surrounding prion-like mechanisms of tau pathology and will also serve as a valuable model for drug screening and intervention.

## 45. Two prion protein paralogs of zebrafish have opposing impacts on seizure susceptibility

Laszlo F. Locskai, Richard Kanyo, Patricia L.A. Leighton and W. Ted Allison

Centre for Prions & Protein Folding Disease, University of Alberta, Edmonton AB, Canada; Department of Biological Sciences, University of Alberta, Edmonton *AB*, Canada

**ABSTRACT**

**Background**: Cellular prion protein (PrP^C^) is famous from its misfolded counterpart, PrP^Sc^, which is involved in neurodegenerative diseases such as Creutzfeldt Jakob’s disease. PrP^C^ has also been linked to Alzheimer’s disease, the most prevalent form of dementia, by direct interactions with amyloid-β oligomers and amyloid-β precursor protein (APP). Our focus here is on understanding PrP and APP physiological interactions and functions. Reduced protein functions likely impact disease progression, hampering the engineering of potential therapeutics. Using the mutant zebrafish we have engineered, we seek to explore the mechanism of neural regulation attributable to zebrafish homologs of PrP^C^.

**Materials**: We have engineered mutant zebrafish with disruptions in homologs of *PRNP* (encoding PrP^C^) and *APP*, being *prp1*^−/-^, *prp2*^−/-^ or compound *prp1*^−/-^; *prp*2^−/-^, and *appa*^−/-^. Zebrafish larvae were treated with convulsants pentylenetetrazol (PTZ) or kainic acid (KA) via bath treatment to induce seizures. Seizure responses were measured by EthoVision behavioural tracking software. The neural activity of free-swimming larvae was also quantified during induced seizures by genetically encoded Calcium Modulated Photoactivatable Ratiometric Integrator (CaMPARI).

**Results**: When induced with the convulsant PTZ, *prp2^−/-^* mutants have a large increase in seizure response compared to wildtype whereas *prp1*^−/-^ exhibit a more modest increase. Neural activity in the hindbrain measured by CaMPARI presented parallel findings insomuch that PTZ induced increased activity in *prp2^−/-^* zebrafish compared to wildtype. Initial results with KA suggest zebrafish exhibit a decrease in locomotor activity in *prp1* mutants compared to wildtype. This contrasts compound mutant disruption, which appears to be similar to locomotor activity versus wildtype. PTZ induction of *appa^−/-^* larvae caused an increased seizure response of a similar PTZ dose-response profile, but higher in magnitude to *prp1^−/-^.*

**Conclusions**: These results suggest that PrP^C^ homologs have taken on non-additive or perhaps opposing functions in zebrafish neurophysiology, and that *appa* functions in neuroprotection. It appears each paralog may regulate ion channels in opposing ways to mediate their neuroprotective function, though further research is warranted. Understanding which specific channels are regulated by each protein paralog, and in what fashion, has potential to illuminate the mechanism of PrP^C^ and APP function at the synapse.

## 46. Characterization of Syrian hamster adapted H-BSE prion

Kohtaro Miyazawa^a^, Kentaro Masujin^b^, Yuichi Matsuura^a^, Yoshifumi Iwamaru^a^

^a^Prion disease unit, National Institute of Animal Health (NIAH), National Agriculture and Food Research Organization (NARO), Tsukuba, Ibaraki, Japan; ^b^Exotic Disease Research Unit, Division of Transboundary Animal Diseases, NIAH, NARO, Kodaira, Tokyo, Japan

**CONTACT** Kohtaro Miyazawa miyazawak@affrc.go.jp

**ABSTRACT**

**Introduction**: Bovine spongiform encephalopathy (BSE) is a prion disease in cattle, which is characterized by spongiform changes and accumulation of a disease-associated isoform of prion protein (PrP^d^). Nowadays, BSE is classified into three types (classical type: C-BSE, high-type: H-BSE, and low-type: L-BSE) according to the electrophoretic migration of their proteinase K resistant core of PrP^d^ (PrPres). Although Syrian hamster models have already proved to be useful for studying prion diseases, transmission of C- and H-BSE prions from cattle to Syrian hamsters has been inefficient. We previously reported that mouse-passaged C-BSE prion was transmissible to Syrian hamsters. Recently, we also achieved successful transmission of H-BSE prion from cattle to transgenic mice overexpressing hamster PrP (TgHaNSE). The distribution of immunolabeled PrP^d^ in the brain was different between TgHaNSE mice infected with cattle H-BSE prion and those infected with mouse-passaged C-BSE prion even after the third passage in TgHaNSE mice. However, the molecular mass of the unglycosylated PrPres and glycoform profiles were indistinguishable between them. Because these results were obtained under artificial condition in TgHaNSE mice, we performed a subsequent transmission of TgHaNSE mouse-passaged H-BSE prion to Syrian hamsters and compared the biological and biochemical properties of Syrian hamster-adapted H-BSE prion to those of Syrian hamster-adapted C-BSE and L-BSE prions.

**Results**: Syrian hamsters inoculated with 10% brain homogenates of H-BSE prion-infected TgHaNSE mouse developed the neurological signs of the disease after long incubation periods of approximately 450 days. Even after the second passage, hamsters survived for ~390 days. The PrPres banding patterns and the glycoform profiles observed in H-BSE prion-infected TgHaNSE mice were maintained after the transmission into Syrian hamsters. Moreover, PrPres banding patterns of hamsters infected with H-BSE prion were identical with those of hamsters infected with C-BSE prion even after the second passage. On the other hand, the molecular mass of the unglycosylated PrPres of hamsters infected with L-BSE prion was slightly lower than those of hamsters infected with H- and C-BSE prions. Glycoform profiles were quite similar between hamsters infected with three different BSE prions. But the distribution of immunolabeled PrP^d^ in the brain and the lesion profiles were different between them.

**Conclusion**: Our data suggest that the PrPres banding patters and the glycoform profiles may be influenced by the properties of host PrP^C^ rather than by prion strain. On the other hand, biological and pathological features including survival periods, PrP^d^ distribution in the brain and the lesion profiles may be determined by prion strain itself. It is interesting that the ratio of di-glycosylated PrPres is dominant in Syrian hamsters infected with all three BSE prions. These models might be useful for investigations of the mechanisms underlying the conversion from PrP^C^ to PrP^Sc^.

## 47. Sugar analogues and their mechanism of action as prion disease therapy

Garrett J.B. Molner^a^, Manjeet Kumar^a,b^ and Valerie L. Sim^a,b^

^a^Centre for Prions and Protein Folding Diseases, University of Alberta; ^b^Department of Medicine, Division of Neurology, University of Alberta

**CONTACT** Garrett J.B. Molner gmolner@ualberta.ca

**ABSTRACT**

A central hallmark of prion disease is the conversion of normal cellular prion protein (PrPC) to a pathogenic, protease resistant form (PrPSc) that ultimately leads to neurodegeneration. Species devoid of prion protein are incapable of propagating prions and therefore are incapable of prion infection.1 Interestingly, pathogenic prions are hypothesized to exist in strains which may induce distinct strain-specific pathologies and phenotypes. One phenotypic strain characteristic is the relative amounts of un, mono, and di-glycosylated PrP.2 While strain information is largely thought to be encoded in the protein core conformation, there is evidence to suggest a role for glycosylation in PrP conversion and trafficking.3–5 From this concept, both a potential therapeutic strategy and an opportunity to examine a role for glycosylation in strain-restricted conversion is possible.

We manipulated PrPC glycosylation in cell culture using naturally occurring sugar analogues and their alky-derivatives that target α- and β-glucosidases. Treatment led to the elimination of PrPSc in CAD5 cells chronically infected with RML strain prions. However, there were concomittent changes in PrPC levels, leading us to question whether the mechanism of action was indeed by alteration of conversion susceptibility of PrPC (with modified sugars), or whether this treatment led to a sequestration or degradation of PrPC, reducing its likelihood of conversion. To fully understand the mechanism of inhibition, we are using immunoblot, glycoform profiling, and confocal microscopy to determine the effect these compounds have on PrP glycosylation and cellular localization. Results will be discussed in the context of using sugar analogues as a prion therapeutic.

### 

References[1]BüelerH, et al. Cell
1993;73(7):1339–47.10.1016/0092-8674(93)90360-38100741[2]Khalili-ShiraziA, et al.
J Gen Virol. 2005;86(9):2635–44.10.1099/vir.0.80375-016099923[3]MakaravaN, et al.
Acta Neuropathol Com. 2018;6(1):92. 4.10.1186/s40478-018-0597-yPMC613479230208966[4]LiJ, et al.
Science. 2010;327(5967):869–72.10.1126/science.1183218PMC284807020044542[5]KatorchaE, et al. RepSci
2015;5:16,912.

## 48. Transcriptomic analysis reveals conserved cross species functions of the cellular prion protein

Niall M Pollock^a,b^, Patricia L. A Leighton^a,b^, Gavin J. Neil^a^ and W. Ted Allison^a,b^

^a^Department of Biological Sciences, University of Alberta, Edmonton *AB*, Canada; ^b^Centre for Prion and Protein Folding Diseases, University of Alberta, Edmonton *AB*, Canada

**CONTACT** Niall M Pollock nmpolloc@ualberta.ca

**ABSTRACT**

**Introduction**: Cellular Prion Protein (PrP^C^) is well-studied due to being able to misfold into Scrapie Prion Protein (PrP^SC^) causing progressive and fatal neurodegenerative diseases both in humans and other animals. Normally folded PrP^C^ is implicated in the pathology of Alzheimer’s disease, the leading cause of dementia worldwide, through high affinity binding with soluble amyloid-β oligomers. However, the normal function(s) of PrP^C^ are not well understood. We have used our *prp1; prp2* compound homozygous knockout mutant zebrafish to identify potentially conserved, cross-species functions of PrP^C^ using RNA-sequencing analysis in addition to exploring the increasing evidence for PrP^C^ involvement in cell adhesion.

**Methods**: Three-day post fertilization wild-type and *prp1^ua5003/ua5003^; prp2^ua5001/ua5001^* zebrafish larvae underwent PE100-125 HiDeq2500 RNA-sequencing. Three biological replicates each consisting of 50 pooled larvae were used for each genotype. Analysis of sequencing results was processed using Geneious R9/10 and R packages cufflinks and cummeRbund. Alterations in transcript abundance were confirmed using RT-qPCR for selected genes of interest. Further cell adhesion studies were carried out through *in situ* hybridization analysis and immunohistochemistry.

**Results**: RNA-sequencing analysis of mutant fish show large changes in transcript abundance (log_2_ fold change of 0.5 or greater) of 1,249 genes compared to wild-type fish. Of interest are genes involved in cell adhesion, including *st8sia2, ncam1a* and members of the protocadherin family, which are thought to be involved during early development and continued maintenance of the central nervous system (CNS). Through search and comparison with published transcriptomes from embryonic PrP^C^ knockout mice, we have identified many common affected biological processes in our mutant zebrafish including apoptosis, proteolysis, oxidative stress and cell proliferation, and adhesion. Further analysis on cell adhesion through RT-qPCR of *ncam1a* and *st8sia2* shows a significant reduction in transcript abundance between wild-type and mutant fish, with *in situ* hybridization of *ncam1a* confirming a decreased distribution of mRNA across the CNS.

**Conclusions**: Our transcriptomic sequencing analysis supports a role for PrP^C^ during early development and sustainment of the CNS, seen through significant changes in transcript abundance in members of the protocadherin family and other genes responsible for cell adhesion and proliferation. We propose cell adhesion is an evolutionary conserved function of PrP^C^ which will be of important consideration regarding how PrP^C^ function may be affected during disease.

**KEYWORDS** Cellular prion protein; cell adhesion; RNA-sequencing; zebrafish

## 49. The agent of transmissible mink encephalopathy passaged in sheep is similar to BSE-L

E. D. Cassmann^a,b^, S. J. Moore^a,b^, R. D. Kokemuller^a^, A. Balkema-Buschmann^c^, M. H. Groschup^c^ and J. J. Greenlee^a^

^a^Virus and Prion Research Unit, National Animal Disease Center, ARS, United States Department of Agriculture, Ames, IA, USA (EDC, SJM, RDK, JJG); ^b^Oak Ridge Institute for Science and Education (ORISE) through an interagency agreement between the U.S. Department of Energy (DOE) and the U.S. Department of Agriculture (USDA). ORISE is managed by ORAU under DOE contract number DE-SC0014664. (EDC, SJM), Department of Veterinary Pathology, Iowa State University, Ames, IA, USA (JDS); ^c^Institute of Novel and Emerging Infectious Diseases, Friedrich-Loeffler-Institut, Federal Research Institute for Animal Health, Greifswald – Isle of Riems, Germany (ABB, MHG)

**CONTACT** E. D. Cassmann eric.cassmann@usda.gov

**ABSTRACT**

**Introduction**: Transmissible mink encephalopathy (TME) is a fatal neurologic prion disease of farmed mink. Epidemiologic and experimental evidence following a Wisconsin outbreak in 1985 has linked TME to low-type bovine spongiform encephalopathy (BSE-L). Evidence suggests that farmed mink were likely exposed through feeding of BSE-L infected downer cattle. The interspecies transmission of TME to cattle has been documented. Recently, we demonstrated the susceptibility of sheep to cattle passaged TME by intracranial inoculation. The aim of the present study was to compare ovine passaged cattle TME to other prion diseases of food-producing animals. Using a bovine transgenic mouse model, we compared the disease phenotype of sheep TME to BSE-C and BSE-L.

**Materials and Methods**: Separate inoculants of sheep passaged TME were derived from animals with the VRQ/VRQ (VV_136_) and ARQ/VRQ (AV_136_) prion protein genotype. Transgenic bovinized mice (TgBovXV) were intracranially inoculated with 20 µl of 1% w/v brain homogenate. The disease phenotypes were characterized by comparing the attack rates, incubation periods, and vacuolation profiles in TgBovXV mice.

**Results**: The attack rate for BSE-C (13/13), BSE-L (18/18), and TME_VV_ (21/21) was 100%; whereas, the TME_AV_ group (15/19) had an incomplete attack rate. The average incubation periods were 299, 280, 310, and 541 days, respectively. The vacuolation profiles of BSE-L and TME_VV_ were most similar with mild differences observed in the thalamus and medulla. Vacuolation profiles from the BSE-C and TME_AV_ experimental groups were different than TME_VV_ and BSE-L.

**Conclusion**: Overall the phenotype of disease in TME inoculated transgenic mice was dependent on the sheep donor genotype (VV vs AV). The results of the present study indicate that TME isolated from VRQ/VRQ sheep is similar to BSE-L with regards to incubation period, attack rate, and vacuolation profile. Our findings are in agreement with previous research that found phenotypic similarities between BSE-L and cattle passaged TME in an ovine transgenic rodent model. In this study, the similarities between ovine TME and BSE-L are maintained after multiple interspecies passages.

## 50. Cellular Prion protein on human leucocytes is associated with iron metabolism

Ewald Lindner^a^, Nora Woltsche^a^, Domagoj Ivastinovic^a^, Verena Zrim^b^, Rita Moos^c^, Andreas Wedrich^a^ and Adriano Aguzzi^c^

^a^Department of Ophthalmology, Medical University Graz; ^b^Core Facility Imaging, Center for Medical Research Graz; ^c^Institute of Neuropathology, University Hospital Zurich

**CONTACT** Ewald Lindner Ewald.lindner@medunigraz.at

**ABSTRACT**

**Introduction**: The function of the prion protein remains elusive. Several findings indicate a role in iron metabolism. In this study, we wanted to investigate an association between indicators of iron storage and the expression of prion protein on human leucocytes in glaucoma patients.

**Materials and Methods**: Patients were recruited at the Department of Ophthalmology at the Medical University Graz. Twenty patients with glaucoma were included. The expression of prion protein on CD3+ CD4+, CD3+ CD8+ and CD14+ leukocytes was investigated by FACS with POM1 antibody.

**Results**: The expression of Prion Protein correlated significantly with soluble transferrin receptor, haemoglobin, and serum iron (Pearson *r* > 0.6; *p*-value < 0.01). No association was found between expression of prion protein and glaucoma progression.

**Conclusions**: We found a strong correlation between iron metabolism and the expression of prion protein on human leucocytes. This presents add to our understanding of the function and regulation of the cellular prion protein.

**KEYWORDS:** Prion protein; human leucocytes; soluble transferrin receptor; haemoglobin

## 51. Cryptic prion strains/variants appear when anti-prion systems are disabled

Reed B. Wickner, Moonil Son, Evgeny Bezsonov, Songsong Wu, Morgan Dewilde and Herman K. Edskes

Laboratory of Biochemistry and Genetics, National Institute of Diabetes and Digestive and Kidney Diseases, National Institutes of Health, Bethesda, MD, USA

**CONTACT** Reed B. Wickner wickner@helix.nih.gov

**ABSTRACT**

The yeast prions [PSI+] and [URE3] are in-register parallel amyloids of Sup35p and Ure2p, respectively. Ssb1,2p are ribosome-associated Hsp70s that ~10-fold repress [PSI+] generation. Upf1,2,3p are factors essential for mRNA nonsense-mediated decay that, at normal levels, cure >90% of [PSI+] variants arising in any *upf* mutant. The formation of the Upf1,2,3-Sup35 complex is critical for the curing process. The *upf* mutants show 10–15 fold elevation in frequency of spontaneous or induced [PSI+] generation. By controlling cellular levels of 5-pyrophospho-inositol pentaphosphate, Siw14p prevents the propagation of about half of the [PSI+] variants arising in its absence. Normal levels of the disaggregating chaperone Hsp104 cures about half of the [PSI+] variants that arise in an *hsp104^T160M^* mutant defective in the Hsp104 overproduction curing activity. Normal levels of Btn2p and Cur1p are paralogs that cure ~90% of [URE3] prion strains/variants arising in a *btn2*Δ *cur1*Δ strain, preferentially those with low propagon numbers. Btn2p (but not Cur1p) cures by collecting Ure2p amyloid filaments at a single site in the cell co-inciding with Btn2p itself. Cell division results in frequent curing of one of the daughter cells.

The common thread of most of these systems is that prion strains that would not survive in a wild type strain are detected in mutants defective in one or more of these anti-prion systems. We infer that the normal cell experiences a large load of nascent prions, most of which are quickly eliminated by one or more of these systems. The mechanistic details, insofar as we know them, argue that each of these systems are distinct. The excess prion strains/variants detected in cells defective in one anti-prion system are evidently immune to curing by the other anti-prion systems, indicating that each anti-prion system defines a new class of prion strains/variants. It is likely that some prion/prion-like diseases are a consequence of breakdown of homologous/analogous human anti-prion systems.

Future work will address the following questions:

- What other components are involved in each of these processes? Is there any overlap?
- Do cells defective in multiple anti-prion systems show even higher prion generation frequencies?
- What other anti-prion systems are there?

## 52. Microglial-specific exosome isolation from prion-infected fluids

Stephanie Booth, Lise Lamoureux and Sarah Medina

Public Health Agency of Canada, NML

**ABSTRACT**

Prion disease is caused by the conversion of PrP^c^ (cellular prion protein) to PrP^sc^ (abnormal prion protein) which aggregates in the brain causing spongiform neurodegeneration. In response, astrocytes and microglia are activated. Both PrP^c^ and PrP^sc^ have been shown to be associated with exosomes, which are thought to be involved in prion pathogenesis. Exosomes are small 30–150nm vesicles that are released from cells via exocytosis into biological fluids. They are involved in cell to cell communication as they can cross biological barriers easily. Exosomes contain protein, lipids, and nucleic acids that are representative of the cell types from which they originate. Therefore, they could be specifically isolated and used as the basis for cell-type specific biomarker discovery. Few studies have looked at prion-specific changes in the expression of biomolecules in exosomes from cells that are involved in the disease process. Exosomes originating from the brain (specifically microglia) could be an ideal source for disease-specific molecular signatures from early stages of disease. We hypothesized that TMEM119, a transmembrane protein that is expressed highly specific on microglial cells, could be used as a marker to specifically select microglial exosomes. The goal of this preliminary study was to develop methods to isolate microglial-specific exosomes from cells infected with prions. We used western blots and nanoparticle tracking analysis (NTA) to look at the composition of microglial-specific exosomes. Exosomes were isolated in media from BV2 cells (a microglial cell line). A TMEM119 antibody was labelled with a fluorophore and binding to exosomes was visualized via NTA. Exosomes were also isolated from prion-infected and uninfected primary cerebellum slice cultures. TMEM119 antibody was bound to beads which could then successfully ‘pull-out’ microglial specific exosomes. This methodology will be tested for its utility in prion disease-specific biomarker discovery from microglial exosomes isolated from biofluids from infected animals.

## 53. Evaluation of the inter-species transmission potential of different CWD isolates

Rodrigo Morales**^a^**, Carlos Kramm**^a^**^,b^, Paulina Soto**^a^**, Adam Lyon**^a^**, Sandra Pritzkow**^a^**, Claudio Soto**^a^**

^a^Mitchell Center for Alzheimer’s disease and Related Brain Disorders, Dept. of Neurology, McGovern School of Medicine University of Texas Health Science Center at Houston, TX, USA; ^b^Facultad de Medicina, Universidad de los Andes, Santiago, Chile

**ABSTRACT**

Chronic Wasting Disease (CWD) has reached epidemic proportions in North America and has been identified in South Korea and Northern Europe. CWD-susceptible cervid species are known to share habitats with humans and other animals entering the human food chain. At present, the potential of CWD to infect humans and other animal species is not completely clear. The exploration of this issue acquires further complexity considering the differences in the prion protein sequence due to species-specific variations and polymorphic changes within species. While several species of cervids are naturally affected by CWD, white-tailed deer (WTD) is perhaps the most relevant due to its extensive use in hunting and as a source of food. Evaluation of inter-species prion infections using animals or mouse models is costly and time consuming. We and others have shown that the Protein Misfolding Cyclic Amplification (PMCA) technology reproduces, in an accelerated and inexpensive manner, the inter-species transmission of prions while preserving the strain features of the input PrP^Sc^. In this work, we tested the potential of different WTD-derived CWD isolates to transmit to humans and other animal species relevant for human consumption using PMCA. For these experiments, CWD isolates homozygous for the most common WTD-PrP polymorphic changes (G96S) were used (96SS variant obtained from a pre-symptomatic prion infected WTD). Briefly, 96GG and 96SS CWD prions were adapted in homologous or heterologous substrate by PMCA through several (15) rounds. End products, as well as intermediates across the process, were tested for their inter-species transmission potentials. A similar process was followed to assess seed-templated misfolding of ovine, porcine, and bovine PrP^C^. Our results show differences on the inter-species transmission potentials of the four adapted materials generated (PrP^C^/PrP^Sc^ polymorphic combinations), being the homologous combinations of seed/substrate the ones with the greater apparent zoonotic potential. Surprisingly, 96SS prions adapted in homologous substrate were the ones showing the easiest potential to template PrP^C^ misfolding from other animal species. In summary, our results show that a plethora of different CWD isolates, each comprising different potentials for inter-species transmission, may exist in the environment. These experiments may help to clarify an uncertain and potentially worrisome public health issue. Additional research in this area may be useful to advise on the design of regulations intended to stop the spread of CWD and predict unwanted zoonotic events.

## 54. Analysis of amyloid properties of MBP protein in the brain of rat R. norvegicus

A. A. Shenfeld^a,b*^, M. J. Velizhanina^a^, J. V. Sopova^a,b^, T. A. Belashova^b^ and A. P. Galkin^a,b^

^a^St-Petersburg State University, dept of Genetics and Biotechnology, St-Petersburg, Russia; ^b^St-Petersburg branch of Vavilov Institute of General Genetics, Lab. of Genetic Modeling of Human Diseases, St-Petersburg, Russia

**CONTACT** A. A. Shenfeld shenaleksandr@mail.ru

## 55. PAD-beads enhances detection of PrP^Sc^ in scrapie infected sheep brain samples using real-time quaking-induced conversion

Soyoun Hwang, Rohana P. Dassanayake and Eric M. Nicholson

United States Department of Agriculture, Agricultural Research Service, National Animal Disease Center, Ames, Iowa, USA

**CONTACT** Soyoun Hwang Soyoun.Hwang@ars.usda.gov

**ABSTRACT**

Scrapie, a transmissible spongiform encephalopathy (TSE), naturally occurs in sheep and goats. This fatal neurodegenerative disease results from misfolding of the cellular prion protein (PrP^C^) to a pathogenic prion protein form (PrP^Sc^). This pathogenic form accumulates in the brain and other tissues.The presence of PrP^Sc^ can been detected by an *in vitro* conversion assay known as real-time quaking induced conversion (RT-QuIC). RT-QuIC has been used to detect PrP^Sc^ in a variety of biological tissues from brains to fluids. While this technique is both rapid and sensitive enhancing the detection of prions would be valuable in the diagnostic laboratories. In this study, we evaluated if RT-QuIC can be coupled to PrP^Sc^ binding to a PrP^Sc^ specific ligand found on commercially available PAD-Beads for clean up of PrP^Sc^ containing samples enhanced prion detection. Coupling the RT-QuIC amplification to PAD-Bead based cleanup allowed detection of scrapie prion rapidly and without dilutions sheep brain homogenates prior to RT-QuIC. The PAD-Bead sample pretreatment step prior to RT-QuIC is a useful enhancement in the diagnosis of TSEs.

**KEYWORDS:** Scrapie; prion diseases; RT-QuIC; transmissible spongiform encephalopathy; PAD-Beads; magnetic particle extraction

## 56. Understanding chronic wasting disease spread potential for at-risk species

Catherine I. Cullingham, Anh Dao, Debbie McKenzie and David W. Coltman

Department of Biological Sciences, University of Alberta, Edmonton *AB*, Canada

**CONTACT** Catherine I. Cullingham cathy.cullingham@ualberta.ca

**ABSTRACT**

Genetic variation can be linked to susceptibility or resistance to a disease, and this information can help to better understand spread-risk in a population. Wildlife disease incidence is increasing, and this is resulting in negative impacts on the economy, biodiversity, and in some instances, human health. If we can find genetic variation that helps to inform which individuals are susceptible, then we can use this information on at-risk populations to better manage negative consequences. Chronic wasting disease, a fatal, transmissible spongiform encephalopathy of cervids (both wild and captive), continues to spread geographically, which has resulted in an increasing host-range. The disease agent (PrP^CWD^) is a misfolded conformer of native cellular protein (PrP^C^). In Canada, the disease is endemic in Alberta and Saskatchewan, infecting primarily mule deer and white-tail deer, with a smaller impact on elk and moose populations. As the extent of the endemic area continues to expand, additional species will be exposed to this disease, including bison, bighorn sheep, mountain goat, and pronghorn antelope. To better understand the potential spread-risk among these species, we reviewed the current literature on species that have been orally exposed to CWD to identify susceptible and resistant species. We then compared the amino acid polymorphisms of PrP^C^ among these species to determine whether any sites were linked to susceptibility or resistance to CWD infection. We sequenced the entire PrP coding region in 578 individuals across at-risk populations to evaluate their potential susceptibility. Three amino acid sites (97, 170, and 174; human numbering) were significantly associated with susceptibility, but these were not fully discriminating. All but one species among the resistant group shared the same haplotype, and the same for the susceptible species. For the at-risk species, bison had the resistant haplotype, while bighorn sheep and mountain goats were closely associated with the resistant type. Pronghorn antelope and a newly identified haplotype in moose differed from the susceptible haplotype, but were still closely associated with it. These data suggest pronghorn antelope will be susceptible to CWD while bison are likely to be resistant. Based on this data, recommendations can be made regarding species to be monitored for possible CWD infection.

**KEYWORDS:** Chronic wasting disease; Prnp; wildlife disease; population genetics; ungulates

## 57. Oligomerization of Aβ(1–40/42) in the presence of somatostatin-14 investigated *in silico*

Min Wu^a^, Lyudmyla Dorosh^a,b^, Gerold Schmitt-Ulms^c^, Holger Wille^d,e^, Maria Stepanova^a,b^

^a^Department of Electrical and Computer Engineering, University of Alberta, Edmonton, Canada; ^b^National Research Council of Canada, Edmonton, Alberta, Canada; ^c^Laboratory Medicine and Pathobiology, University of Toronto, Toronto, Canada; ^d^Department of Biochemistry, University of Alberta, Edmonton, Canada; ^d^Centre for Prions and Protein Folding Diseases, University of Alberta, Edmonton, Canada

**CONTACT** Min Wu wu2@ualberta.ca; Lyudmyla Dorosh dorosh@ualberta.ca

**ABSTRACT**

Alzheimer’s disease (AD) is associated with pathological oligomeric aggregates and fibrils formed by amyloid beta (Aβ) peptides. Experiments indicated that somatostatin-14 (SST_14_), a small cyclic peptide found in the brain, may influence the formation of toxic oligomers of Aβ_1–42_, but not Aβ_1-40_ [1]. However, the specific role of SST_14_ and the reason for the different responses of Aβ_42_ and Aβ_40_ remain unclear. We have investigated the effects of SST_14_ on aggregation of Aβ_1-40_ and Aβ_1–42_ peptides using 100 ns long all-atom molecular dynamics simulations. Starting from randomly positioned Aβ_40/42_ and SST_14_ chains in water, we observed the formation of oligomers, and investigated their structure and dynamics. For comparison, we have investigated a similar system containing Aβ_1–42_ and the arginine vasopressin (AVP) peptide as a negative control [1]. We have employed well-established structural biology tools, as well as a novel essential collective dynamics (ECD) method [2], which allows analysing pair correlations, main-chain flexibilities, and domains of correlated motion within the same framework. We observed formation of stable co-oligomers containing Aβ chains and SST_14_ or AVP molecules in all the three systems, Aβ_42_-SST_14_, Aβ_40_-SST_14_, and Aβ_42_-AVP. During the simulations, the number of hydrogen bonds in the systems gradually increased. This was accompanied by a buildup of salt bridges, hydrophobic contacts, and other interactions responsible for the oligomerization process. Interestingly, C-terminal residues of both Aβ_42_ and Aβ_40_ are often located at the surface of the oligomers, whereas their central parts stay preferentially in the interior regions. The ECD analysis indicated that in most Aβ oligomers, C-termini exhibited greater main-chain flexibility in comparison to central regions, also indicating a stronger solvent exposure of the C-termini. The solvent accessible surface area (SASA) of hydrophobic side groups is the greatest in Aβ_42_-SST_14_ oligomers, in part due to the larger hydrophobic SASA of SST_14_ in comparison to the AVP control. Aβ_42_-AVP oligomers are relatively small in size, and their hydrophobic SASA is less than in the other systems. We conclude that differences in aggregation of Aβ_42_-SST_14_ and the other two systems may result from a greater hydrophobic SASA of both Aβ_42_ and SST_14_, producing a stronger ‘sticky surface’ effect. In Aβ_42_-AVP the effect appears lessened by a lower hydrophobicity of AVP, whereas in Aβ_40_-SST_14_, the reduced number of solvent-exposed hydrophobic C-terminal residues of Aβ may play a role.

### 

References[1]WangH, MuiznieksLD, GhoshP, et al.
Somatostatin binds to the human amyloid β peptide and favors the formation of distinct oligomers. eLife. 2017;6:e28401.2865031910.7554/eLife.28401PMC5505701[2]DoroshL, StepanovaM.
Probing oligomerization of amyloid beta peptide in silico. Mol.BioSyst.
2017;13:165–182.10.1039/c6mb00441e27844078

## 58. Understanding the mechanisms and effects of impaired Rab7 function in prion infection

Pearl Cherry, Su Yeon Shim and Sabine Gilch

Department of Ecosystem and Public Health, Calgary Prion Research Unit, Faculty of Veterinary Medicine; Hotchkiss Brain Institute; University of Calgary, Calgary, Canada

**CONTACT** Pearl Cherry pearl.cherry@ucalgary.ca

**ABSTRACT**

**Background**: In persistently prion-infected cell cultures, reduced membrane association of Rab7 and, as a consequence, reduced lysosomal maturation and impaired lysosomal degradation is observed. Other hallmarks of prion infections are increased cholesterol levels and impaired axonal retrograde trafficking. Targeted cargo trafficking is regulated mainly by rab proteins and requires them to shuttle between an active GTP and membrane bound and an inactive cytosolic state. Rab7 is a protein essential for intracellular transport of cargos like low density lipoprotein (LDL) and neurotrophins. The aim of this study is to elucidate the causes and effects of reduced membrane bound Rab7, specifically on how it influences PrPSc propagation, trafficking of low density lipoprotein (LDL) and neurotrophins and hence its effect on cholesterol metabolism and neurodegeneration.

**Methods**: We used prion infected (strain 22L) N2a cells for transient transfection with different functional mutants of Rab7 including the wild type (WT), the constitutively active (Q67L) and the transdominant negative (T22N) mutants for 48 h and analysed PrPSc levels. The differential effect of overexpression of these mutants on cholesterol metabolism in 22LN2a cells is analysed through Amplex cholesterol assay. Immunofluorescence experiments are conducted using an antibody against Rab7-GTP in prion infected and non-infected cell lines. Trafficking of LDL is analysed using by pulse-chase experiments with fluorescently labelled LDL and co-localization with endosomal markers.

**Results**: We demonstrate that in the prion infected cell lines, transient over-expression of the constitutively active mutant Rab7 reduces PrPSc propagation. Since prion infected cell cultures exhibit an increase in the cholesterol levels, we analysed how different functional mutants of Rab7 regulate cholesterol levels and observed that overexpression of the constitutively active Q67L mutant reduces the cholesterol level with respect to Rab7-WT mutant in 22LN2a cells. Overexpression of Rab7-WT did neither affect PrPSc levels, nor cholesterol content in those cells. This indicates that activation of Rab7 by GDP/GTP exchange might be impaired in 22LN2a cells. Preliminary results of a comparative analysis of Rab7-GTP in N2a and 22LN2a point at a reduction of Rab7-GTP upon prion infection. Since Rab7 is required for LDL transport, LDL trafficking is analysed to define a link between aberrant vesicle trafficking and enhanced cholesterol synthesis.

**Conclusion**: Through this study, we shed light on the underlying mechanisms and effects of impaired membrane association of Rab7 in prion infection. These effects include deterioration of cellular processes such as vesicle trafficking and cholesterol metabolism, hence favouring PrPSc propagation.

## 59. The role of glial cells in BSE pathology: a histomorphological and immunohistochemical investigation

Christine Fast^a^, Kristina Santiago-Mateo^b^, Rakhi Katoch^b^, Martin H. Groschup^a^, Ute Ziegler^a^, Stefanie Czub^b^

^a^Friedrich-Loeffler Institut, Isle of Riems, Germany; ^b^Canadian Food Inspection Agency, Lethbridge, Alberta, Canada

**CONTACT** Christine Fast Christine.Fast@fli.de

**ABSTRACT**

**Background/Introduction**: Bovine spongiform encephalopathy (BSE) is a fatal neurological disease of cattle associated with the accumulation of an altered form of the cellular prion protein, termed pathological prion protein (PrP^BSE^) The pathomechanisms linking abnormal protein accumulation with the typical severe neurological alterations in BSE are less than clear. Here we investigated the role of glia cells in the pathology of BSE infected cattle, specifically if and to which degree the histopathological changes are glia mediated and therefore due to the host response and not to the BSE agent itself.

**Material and Methods**: We examined 29 experimentally BSE infected cattle at preclinical (no PrP^BSE^ in the brain stem), late preclinical (no clinical signs, first traces of PrP^BSE^ in the brain stem), and clinical (clinical signs and distinct accumulation of PrP^BSE^ in the brain stem) stages of the disease. The following defined regions of the CNS were examined: Obex, Red Nucleus, Cerebellum, Parietal Cerebrum, Septal Nucleus, and Hippocampus and both histopathological lesions and PrP^BSE^ accumulation were determined. Additionally, antibodies Iba-1, CX3CR1, and GFAP were used to visualize and identify glial involvement within the context of the disease. In addition, four unchallenged animals of the same age groups were included as negative controls.

**Results**: PrP^BSE^ is found only in neurons and microglia. Different analytic approaches showed that the most consistent changes in glial numbers and phenotypes were detected in the Obex, Red Nucleus, and Hippocampus. However, only in clinical affected cattle, we observed with all antibodies a mild diffuse increase in glial reactivity, indicating a gliosis, as compared to controls, preclinical, and late preclinical animals. Additionally, different glial reaction patterns were almost exclusively detected in clinical animals. This includes a hyperplastic and in parts irregular appearance of the glial network, an accumulation of clumsy cells with shortened processes and rounded cell bodies, a diffusely distributed coarse granular staining and occasionally perineuronal cluster of glial cells.

**Conclusion**: Apart from neurons, we identified microglia as the only other PrP^BSE^ accumulating cell population; computerized assessment of IBA1, CX3CR1, and GFAP antibodies are ongoing. The preliminary results presented here indicate that the activation of a glial response is a rather late event, most probably associated with the accumulation of PrP^BSE^.

## 60. Caribou *Prnp* polymorphism distribution in Canada and its impact on CWD pathogenesis

Maria I. Arifin^a^, Samia Hannaoui^a^, Yuan-Hung Huang^a^, Gordon Mitchell^b^, Antanas Staskevicius^b^, Lech Kaczmarczyk^c,d^, Walker Jackson^c,d^ and Sabine Gilch^a^

^a^Department of Ecosystem and Public Health, University of Calgary; ^b^National and OIE Reference Laboratory for Scrapie and CWD, Ottawa Laboratory Fallowfield, Canadian Food Inspection Agency; ^c^Wallenberg Center for Molecular Medicine, Department of Clinical and Experimental Medicine, Linköping University; ^d^German Center for Neurodegenerative Disease (DZNE), Bonn

**CONTACT** Maria I. Arifin maria.arifin@ucalgary.ca

**ABSTRACT**

Wild reindeer in Norway were recently diagnosed with chronic wasting disease (CWD) [1]. Prions from CWD-infected deer were also transmissible to other reindeer in experimental settings [2,3]. Although CWD has not yet been reported in wild reindeer (caribou) in Canada, these studies show that they are at risk of infection. The wild-type reindeer, homozygous for serine at prion protein (PrP) residue 138 (138SS), developed clinical disease upon oral inoculation. Animals carrying at least one asparagine allele (138SN, 138NN) accumulated prions only in the periphery. However, both genotypes were susceptible to intracerebral prion inoculation [2,3]. Fallow deer are wild-type 138NN and are resistant to peripheral but not intracerebral prion infection [4]. Thus, we hypothesize that the138N allele, present in caribou herds in Alberta [5], alters CWD pathogenesis by limiting prion transport from the periphery to the CNS. Our aim is to elucidate the mechanisms involved in this process.

We extracted DNA from ±800 caribou, sequenced the *Prnp* open reading frame and determined the 138N allele prevalence in Canadian caribou populations. Results show that the 138N allele was highly prevalent in the Chinchaga woodland caribou population in BC (64%). It was also higher in barren-ground (37%) than in other woodland caribou populations (26%). To analyse how the 138N allele affects CWD pathogenesis, we generated knock-in (KI) mice where the mouse PrP is replaced by wild-type 138SS or 138NN cervid PrP. The KIs were obtained by injecting CRISPR/Cas9-edited C57BL6 embryonic stem cells (Bruce4) into wild-type blastocysts, generating chimeras, and breeding progeny to homozygosity in a C57BL6 background. PrP expression was determined using western blotting and qPCR. Correct PrP post-translational modifications were confirmed by PNGase-F and Endo-H digestion. We will inoculate our KIs with CWD-positive material from the corresponding reindeer genotypes [1,2]. The CWD-infected reindeer material was characterized by real-time quaking-induced conversion (RT-QuIC) and its transmissibility to transgenic mice overexpressing elk PrP (TgElk). Attack rate in the TgElks were almost 100%, except for those inoculated with 138SN lymph node material. Western blotting confirmed the presence of proteinase-K resistant PrP in all terminally ill mice. Our goal is to analyse the susceptibility of KIs carrying the 138N allele to intracerebral and peripheral prion infection. We will also assess the efficiency of prion transport from the periphery to the CNS in intraperitoneally inoculated KIs. Prion strain propagation within a host is highly dependent on its PrP genotype. This study will provide insight into how the 138N PrP specifically influences CWD propagation in caribou.

**KEYWORDS:** Chronic wasting disease; polymorphism; caribou; reindeer; cervid; transgenic mice; knock-in mice

### 

References[1]Benestad, et al.
Vet Res.
2016;47:1.10.1186/s13567-016-0375-4PMC502446227641251[2]Mitchell, et al. PLoS ONE
2012;7(6):e39055.10.1371/journal.pone.0039055PMC337759322723928[3]Moore, et al. Emerg Infect Dis
2016;22(12):2142–5.10.3201/eid2212.160635PMC518914627869594[4]Rhyan, et al.
J Wildl Dis.
2011;47(3):739–4.10.7589/0090-3558-47.3.73921719844[5]Cheng, et al.
Prion. 2017;11(2):136–42.10.1080/19336896.2017.1300741PMC539990428350512

## 61. Prion propagation in cultured astrocytes: a model for prion infection in astroglia

Waqas Tahir^a^, Basant A Abdulrahman^a^, Dalia H Abdelaziz^a^, Simrika Thapa^a^, Rupali Walia^a^ and Hermann M Schatzl^a^

^a^Department of Comparative Biology & Experimental Medicine, Faculty of Veterinary Medicine, University of Calgary, Calgary, Alberta, Canada; ^b^Calgary Prion Research Unit, and Hotchkiss Brain Institute, University of Calgary, Calgary, Alberta, Canada

**CONTACT** Waqas Tahir waqas.tahir1@ucalgary.ca

**ABSTRACT**

**Background**: Neuronal cell lines permissive to prion infection are useful experimental tools for studying the molecular mechanisms underlying prion infection. Such cell models are widely used to find and characterize chemical compounds which have anti-prion potential. Unfortunately, findings from cell culture are not very predictive for *in vivo* efficacy of such compounds. One reason might be that other cell types in the brain propagate prions, and that their processing of prions might differ from that of neurons. Exactly how astrocytes propagate, clear and disseminate prions is not well studied, explained by the lack of astrocyte cell models permissive of stable prion infection.

**Objective**: We investigated the permissiveness of an immortalized and uncloned astrocyte cell line to prion propagation, in order to develop a persistent prion infection model of astrocytes.

**Material and Methods**: We quantified mRNA and cell surface levels of PrP^C^ in astrocytes with qPCR and FACS analysis, respectively, and compared them to those of other permissive neuronal (N2a and CAD5) and non-neuronal (MEF) cell lines. Next, we inoculated immortalized astrocytes with three mouse-adapted scrapie prion strains (22L, RML and ME7), and cultured them continuously for multiple passages. Prion propagation was examined with immunoblotting, RT-QuIC, and immunofluorescence methods at every passage.

**Results**: Surface expression of PrP^C^ was comparable between astrocytes and N2a and CAD5 cells, and was higher than in MEF cells. Astrocytes infected with 22L prions showed PrP^Sc^ in immunoblot analysis throughout all tested passages. Similarly, immunofluorescence results showed that astrocytes were able to propagate 22L prions stably. Infection with Me7 prions was negative in immunoblot, with transient weak signals in immunofluorescence and RT-QuIC analysis. Although RML infection was negative in immunoblot until passage 6 p.i., RML-infected astrocytes had the strongest signal in RT-QuIC in all passages (p1-p6).

**Conclusions**: Our data show that this uncloned astrocyte cell line is permissive to stable prion infection, depending on the prion strain. Whereas infection with 22L prions resulted in the expected strong signals in immunoblot, RT-QuIC, and immunofluorescence analysis, infection with RML prions yielded a stable infection which was positive in RT-QuIC and mostly negative in immunoblot analysis. Obviously, RML and 22L prions were handled very differently by this cell line, with regard to prion conversion and PK resistance. This new cell model will be helpful for better understanding of the role of astrocytes in prion infection.

## 62. A novel *ex vivo* model for characterization of Alzheimer’s disease strains

Hailey E. Pineau^a,b^, Grant Norman^a,b^, David Westaway^a,b^ and Valerie L. Sim^a,b^

^a^Centre for Prions and Protein Folding Diseases, University of Alberta; ^b^Department of Medicine, Faculty of Medicine & Dentistry, University of Alberta

**CONTACT** Hailey E. Pineau pineau@ualberta.ca

**ABSTRACT**

**Background**: Affecting >48 million people worldwide, Alzheimer’s disease (AD) is the leading cause of dementia. Evidence suggests that both amyloid β (Aβ) and tau, the proteins associated with neurodegeneration in AD, exist in many different conformations (strains), which may explain variation in pathology and phenotype between AD patients. Specifically, we are interested in whether strain differences underlie differences in rate of disease progression. Using biophysical techniques and prion-based slice culture assays, we hope to elucidate Aβ properties and plaque morphology characteristic of typical and rapidly progressive AD, and to develop a novel *ex vivo* model that can replicate strain features.

**Methods**: The organotypic slice culture assay (OSCA) is a mouse brain slice culture that can be infected with different strains of prions and undergoes pathology as seen *in vivo*. Because Aβ and tau possess prion-like infectivity, we are adapting this technique to AD using coronal brain slice cultures from 5xFAD and CRND8 mice. These mice express human mutant amyloid precursor protein and develop plaques and other neuropathology by 2 and 3 months of age, respectively. Typical or rapidly progressive AD brain homogenate will be applied to these slice cultures to induce strain-specific aggregates. Using confocal microscopy, we will quantify plaque burden, morphology, localization, and adjacent pathology. ELISA will be done to quantify levels of Aβ40 and Aβ42 in the AD brain samples and slice culture. Guanidinium denaturation curves will allow for comparison of Aβ conformational stability between typical and rapidly progressive AD brain samples. Additionally, real-time quaking induced conversion (RT-QuIC) will be performed to assess seeding ability and aggregation kinetics of the Aβ in all of the AD samples.

**Results**: Confocal images of Aβ plaques stained with thioflavin S and/or 6E10 in fresh and cultured slices will be compared, and the effects of exposure to rapidly progressive AD brain homogenate will be shown. ELISA results will demonstrate a positive correlation between disease duration and hippocampal levels of Aβ42 and RT-QuIC data on aggregation kinetics may also be presented.

**Conclusions**: An *ex-vivo* model of AD that can replicate strain features will facilitate investigation into AD pathogenesis and strain-specific treatments.

## 63. Alteration of prion strain emergence by non-host factors

Sara A. M. Holec and Jason C. Bartz

Department of Medical Microbiology and Immunology, Creighton University School of Medicine, Creighton University, Omaha, Nebraska, USA

**CONTACT** Sara A. M. Holec SaraHolec@creighton.edu

**ABSTRACT**

Prions can persist in the environment for extended periods of time after adsorption to surfaces including soils, feeding troughs, or fences. The adsorption of PrP^Sc^ to surfaces is influenced by a several factors including the prion strain, species of origin, and surface type. Attachment to surfaces can alter PrP^Sc^ properties. The cumulative effect of these factors can result in strain- and soil-specific differences in prion adsorption, infectivity, and response to inactivation. Environmental processes such as wetting and drying or freezing and thawing can decrease PrP^Sc^ conversion efficiency and enhance PrP^Sc^ degradation. Little is known about how strain-specific incomplete inactivation of surface bound PrP^Sc^ affects transmission and strain emergence. To examine this biologically relevant mode of prion transmission, we investigated the effect of surface binding and environmental forces on the emergence of a strain from a mixture.

To investigate the effects of environmental forces on strain emergence, we tested multiple parameters including proteolytic digestion, surface adsorption, and repeated cycles of wetting and drying. Extensive proteinase K (PK) digestion of HY and DY PrP^Sc^ resulted in earlier emergence of HY PrP^Sc^ in protein misfolding cyclic amplification strain interference (PMCAsi) compared to undigested controls. This result confirmed the proof of principle that selective alteration of the starting ratios of conversion competent HY and DY PrP^Sc^ can alter strain emergence. We then further investigated if environmentally relevant factors such as surface binding and weathering could have a similar effect. Adsorption of HY and DY PrP^Sc^ to silty clay loam (SCL) reduces conversion efficiency that can be strain-dependent; however, adsorption of HY and DY PrP^Sc^ to SCL, both individually and together, did not alter strain emergence compared to unbound control PMCAsi reactions. Similarly, repeated cycles of wetting and drying of HY and DY PrP^Sc^ bound to SCL did not alter the emergence of HY PrP^Sc^. This data suggests that prion strain interference can occur when bound to surfaces. Since it is likely that surface bound PrP^Sc^ is responsible for a significant portion of prion transmission under natural conditions, this is a highly significant finding. Under the limited conditions tested, we found that surface binding and weathering did not alter the emergence of a HY PrPSc.

## 64. Investigation of the role of lamin A-induced molecular ageing in tauopathy

Zhuang (George) Han AND David Westaway

Centre for Prions and Protein Folding Diseases, Department of Biochemistry, Faculty of Medicine and Dentistry, University of Alberta

**CONTACT** Zhuang (George) Han zzhan@ualberta.ca

**ABSTRACT**

Tauopathies are a diverse group of age-related neurodegenerative diseases characterised by filamentous Tau lesions in brain [1]. Alzheimer’s disease, frontotemporal dementia and progressive supranuclear palsy are examples of tauopathies that have presented a tremendous challenge to health care systems worldwide [2–4]. Tau is a microtubule-associated protein predominantly expressed in axons of neurons. In disease states, it becomes hyperphosphorylated and migrates to the cell body, forming aggregates that are toxic to neurons [1,5]. Certain tauopathies may associate with impaired lamin-enriched nuclear lamina and thus are also considered laminopathies, at least in the experimental context of *Drosophila melanogaster* [6,7].

Hutchinson Gilford progeria syndrome (HGPS), an autosomal dominant disorder of premature ageing, associates with a truncated form of lamin A (termed ‘progerin’) [8,9]. Neurons in the brain mainly produce abundant lamin C but little lamin A, a consequence of prelamin A mRNA degradation by a brain-specific microRNA [10]. Although this reasonably explains the absence of neurological symptoms in lamin A-induced accelerated ageing, it remains unclear whether progerin – if it were expressed in neurons – would enhance pathological Tau-induced neurotoxicity in brain, given that overexpression of progerin can be toxic to enteric nerves and stem cell-derived neurons [11,12]. To address this question, we are using the HGPS progeric mutation of lamin A to accelerate cellular ageing in petri-dish cultures; we hypothesise that when the expression level of aggregation-prone Tau is low (near-physiological level), lamin A- induced cellular changes might amplify or alter its pathogenic potential.

Progeric and wildtype forms of lamin A were transfected into an HEK293 Tau RD-YFP reporter cell line [13] with subcellular localization of progerin and perturbation of the nuclear lamina assessed by immunohistochemistry. Following transduction of different classes of Tau fibril derived from transgenic P301L mice and human P301L patients, morphologies of Tau aggregates in HEK reporter cells with and without lamin A expression are determined by fluorescence microscopy. Aggregated Tau is then harvested, digested with trypsin and analysed with capillary westerns and folding state-dependent immunoassays. Based upon results from tauopathy cell culture model, the HGPS progeric mutation may be applied to animal studies to augment the use of chronological ageing as a parameter in studies of Tau-mediated pathogenesis.

**KEYWORDS:** Tauopathy; laminopathy; progeria syndrome; ageing; neurodegenerative disease

### 

References[1]LeeVM, GoedertM, TrojanowskiJQ.
Neurodegenerative tauopathies. Annu Rev Neurosci. 2001;24:1121–59.1152093010.1146/annurev.neuro.24.1.1121[2]NathU, Ben-ShlomoY, ThomsonRG, et al.
The prevalence of progressive supranuclear palsy (Steele-Richardson-Olszewski syndrome) in the UK. Brain
2001;124(Pt 7):1438–49.1140833810.1093/brain/124.7.1438[3]PlassmanBL, LangaKM, FisherGG, et al.
Prevalence of dementia in the United States: the aging, demographics, and memory study. Neuroepidemiology. 2007;29:125–132.1797532610.1159/000109998PMC2705925[4]OnyikeCU, Diehl-SchmidJ.
The epidemiology of frontotemporal dementia. Int Rev Psychiatry. 2013;25(2):130–137.2361134310.3109/09540261.2013.776523PMC3932112[5]Mietelska-PorowskaA, WasikU, GorasM, et al.
Tau protein modifications and interactions: their role in function and dysfunction. Int. J. Mol. Sci.
2014;15:4671–4713.2464691110.3390/ijms15034671PMC3975420[6]Frost, et al. Alzheimer’s disease: an acquired neurodegenerative laminopathy. Nucleus
2016;7(3):275–283.10.1080/19491034.2016.1183859PMC499124027167528[7]FrostBardai FH, FeanyMB, et al.
Lamin dysfunction mediates neurodegeneration in tauopathies. Current Biol. 2016;26:129–136.10.1016/j.cub.2015.11.039PMC471333526725200[8]PollexRL, HegeleRA.
Hutchinson-Gilford progeria syndrome. Clin Genet. 2004;66(5):375–81.1547917910.1111/j.1399-0004.2004.00315.x[9]SchreiberKH, KennedyBK.
When lamins go bad: nuclear structure and disease. Cell
2013
152(6):1365–75.2349894310.1016/j.cell.2013.02.015PMC3706202[10]JungHJ, CoffinierC, ChoeY, et al.
Regulation of prelamin A but not lamin C by miR-9, a brain-specific microRNA. Proc. Natl Acad. Sci.
2012;109:E423–E431.2230834410.1073/pnas.1111780109PMC3289373[11]YangSH, ProcacciaS, JungH-J. et al. Mice that express farnesylated versions of prelamin A in neurons develop achalasia. Human Mol. Genet.
2015;24(10):2826–2840.2565240910.1093/hmg/ddv043PMC4406294[12]MillerJD, GanatYM, KishinevskyS, et al.
Human iPSC-based modeling of late-onset disease via progerin-induced aging. Cell Stem Cell. 2013;13:691–705.2431544310.1016/j.stem.2013.11.006PMC4153390[13]SandersDW, KaufmanSK, DeVosSL, et al.
Distinct tau prion strains propagate in cells and mice and define different tauopathies. Neuron
2014;82:1271–1288.2485702010.1016/j.neuron.2014.04.047PMC4171396

## 65. vCJD strain is consistent in individuals of two *PRNP* codon 129 genotypes

Abigail B. Diack^a^, Aileen Boyle^a^, Chris Plinston^a^, Diane Ritchie^b^, Emma Hunt^a^, Matthew Bishop^b^, Robert G. Will^b^ and Jean C. Manson^c^

^a^The Roslin Institute and R(D)SVS, University of Edinburgh, Easter Bush, UK; ^b^National CJD Research & Surveillance Unit, Center for Clinical Brain Sciences, University of Edinburgh, Edinburgh, UK; ^c^University of Edinburgh, Edinburgh, UK

**CONTACT** Abigail B. Diack abigail.diack@roslin.ed.ac.uk

**ABSTRACT**

**Background/Introduction**: The identification of a clinical case of vCJD in a *PRNP* 129 heterozygous individual in 2016 raised concerns over whether 129MV individuals can transmit the vCJD infection and whether the 129MV genotype could give rise to a novel strain of vCJD; both of which have significant public health concerns. To date, of the 178 definite/probable cases of vCJD, only one has been of the 129MV genotype however evidence of abnormal PrP has been observed in two asymptomatic cases of vCJD. We have now undertaken the strain characterization of both a clinical case of vCJD and that of an asymptomatic case of vCJD in 129MV individuals.

**Methods**: CNS material from a 129MV clinical case of vCJD [1] and spleen material from an asymptomatic 129MV individual [2] were inoculated into panels of wildtype mice. Serial passage of the spleen material from the asymptomatic 129MV individual was then performed by inoculating brain homogenate from a TSE positive mouse from each mouse strain into the wildtype mouse panel. This was then repeated for a second mouse passage. A combination of clinical signs, neuropathology (TSE vacuolation and PrP deposition) and biochemical analysis were carried out for each transmission and compared against 129MM and BSE transmission data.

**Results**: Primary passage of the clinical case of 129MV vCJD (CNS) in wildtype mice gave rise to pathologically confirmed (TSE vacuolation and PrP deposition) clinical disease. Differences in transmission data were observed at the primary and first subpassage of the asymptomatic 129MV case (spleen) compared to 129MM transmissions. These differences included low attack rates with no or little TSE vacuolation at primary passage and changes in incubation periods and rankings. These differences were resolved upon further passage giving rise to strain characteristics consistent with 129MM vCJD and BSE transmission data.

**Conclusions**: These studies comprise the first strain characterization of vCJD in 129MV individuals. Upon primary passage of the clinical case of 129MV vCJD transmission characteristics resemble that of the129MM vCJD and BSE strain. While some differences were observed at primary and subpassage of the asymptomatic case, these resolved by the second subpassage and strain characteristics are totally consistent with 129MM vCJD. We demonstrate that vCJD strain properties are not affected by transmission through individuals with the 129MV genotype and thus no alteration in virulence should be associated with different host genotype.

### 

References[1]MokT, JaunmuktaneZ, JoinerS, et al.
Variant Creutzfeldt-Jakob disease in a patient with heterozygosity at PRNP codon 129. N Engl J Med. 2017;376(3):292–4.10.1056/NEJMc161000328099827[2]PedenAH, HeadMW, RitchieDL, et al.
Mok TPreclinical vCJD after blood transfusion in a PRNP codon 129 heterozygous patient. Lancet. 2004;364(9433):527–9.1530219610.1016/S0140-6736(04)16811-6

## 66. Scrapie strain typing of brain and lymph node-derived isolates in ovinized models reveals mixture of substrains with distinct pathobiological properties

Tomás Barrio^a^, Hicham Filali^a^, Alicia Otero^a^, Jessica Sheleby-Elías^a^, Belén Marín^a^, Enric Vidal^b^, Juan María Torres^c^, Martin Groschup^d^, Olivier Andréoletti^e^, Juan José Badiola^a^ and Rosa Bolea^a^

^a^Centro de Encefalopatías y Enfermedades Transmisibles Emergentes, Facultad de Veterinaria, Instituto Agroalimentario de Aragón – IA2, Universidad de Zaragoza – CITA, Zaragoza, Spain; ^b^Priocat Laboratory, Centre de Recerca en Sanitat Animal (CReSA), UAB-IRTA, Universitat Autònoma de Barcelona (UAB), Barcelona, Spain; ^c^Centro de Investigación en Sanidad Animal, CISA-INIA, Madrid, Spain; ^d^Institute of Novel and Emerging Infectious Diseases, Friedrich-Loeffler-Institute, Greifswald-Isle of Riems, Germany; ^e^UMR INRA ENVT 1225- IHAP, École Nationale Vétérinaire de Toulouse, Toulouse, France

**CONTACT** Rosa Bolea rbolea@unizar.es

**ABSTRACT**

Phenotypic variability has been observed in several prion diseases, including human TSEs and scrapie of sheep of goats. This heterogeneity has been associated with distinct prion strains. The existence of strains in prion biology can be accommodated within the protein-only hypothesis by supporting the view that several conformational variants of PrP^Sc^ exist, which encode distinct pathobiological properties. Within this framework, the conformational selection model proposes that a given amino acid sequence for PrP^C^ allows a limited portfolio of PrP^Sc^ conformations, such that the degree of overlapping between host and donor *Prnp* sequences determines the capability of the isolate to cause disease.

Transmission barrier occurs upon heterologous interaction between incompatible PrP sequences, giving rise to a variety of new prion conformers. Therefore, transgenic models expressing homologous PrP^C^ are crucial to faithfully study the actual variety of prion strains. Ovinized models show enhanced susceptibility to infection with scrapie prions and have been employed to characterize strains in natural sheep isolates.

In the present study, we used the transgenic murine lines TgShp and Tg338, which express ovine PrP^C^ ARQ and VRQ, respectively, to bioassay 22 sheep scrapie isolates from distinct outbreaks within the Spain-France-Andorra transboundary territory. Animals were intracerebrally inoculated and survival periods, lesion profiles, PrP^Sc^ distribution, and glycosylation patterns were studied.

In all cases but one, Western blot from sheep tissues disclosed glycosylation patterns compatible with a low molecular weight (Mw) scrapie strain (~19 kDa). Only Inoculum 8L showed PrP^res^ of higher Mw (~21 kDa).

On bioassay, all inocula caused on second-passage TgShp mice similar survival periods together with high attack rates. While most TgShp mice accumulated 19-kDa PrP^res^ in their spinal cords, a number of isolates (including inoculum 8L) triggered deposition of the 21-kDa isoform.

In Tg338 mice, a majority of isolates induced survival times similar to those seen in TgShp, together with high attack rates and 19-kDa PrP^res^ in the spinal cord. However, a group of isolates showed different features consisting in very long survival periods with low attack rates and presence of 21-kDa PrP^res^. Additionally, these animals showed lower vacuolization scores in all evaluated brain areas and occasional presence of amyloid plaques.

These results suggest that some scrapie isolates contain mixtures of substrains that are resolved distinctly in different transgenic lines. A number of isolates seemed to comprise a major 19-kDa component and a minor 21-kDa component that is specifically amplified by the TgShp, but not the Tg338 line. In contrast, some other isolates contained low titers of a 21-kDa, low pathogenicity isoform that seemed to interfere with the propagation of the major 19-kDa substrain exclusively in Tg338 thanks to its amyloidogenic properties, that sequester neurotoxic PrP monomers delaying the onset of clinical signs and restraining the development of neuropathology.

## 67. Diagnostic accuracy of cerebrospinal fluid RT-QuIC in cases of suspected prion disease and the potential utility of using RT-QuIC for public health surveillance

Daniel D. Rhoads^a,b^, Aleksandra Wrona^c^, Aaron Foutz^a^, Curtis Tatsuoka^a,d^, Janis Blevins^a^, Ryan A. Maddox^e^, Ermias D. Belay^e^, Lawrence B. Schonberger^e^, Mark L. Cohen^a,b,f^ and Brian S. Appleby^a,b,f,g^

^a^National Prion Disease Pathology Surveillance Center, Case Western Reserve University, Cleveland, OH, USA; ^b^Department of Pathology, Case Western Reserve University, Cleveland, OH, USA; ^c^Yale University, New Haven, CT, USA; ^d^Department of Biostatistics, Case Western Reserve University, Cleveland, OH, USA; ^e^Centers for Disease Control and Prevention, Atlanta, GA, USA; ^f^Department of Neurology, Case Western Reserve University, Cleveland, OH, USA; ^g^Department of Psychiatry, Case Western Reserve University, Cleveland, OH, USA

**CONTACT** Brian S. Appleby bsa35@case.edu

**ABSTRACT**

**Background**: The clinical heterogeneity, potential mimickers, and rapid progression make prion diseases difficult to diagnose. The only way to definitely diagnose prion disease is via neuropathologic examination, but reliable antemortem diagnoses can be made, especially with the advent of real time quaking induced conversion (RT-QuIC) to detect prions. Individuals with a neuropsychiatric disorder and positive RT-QuIC are considered to have probable sporadic Creutzfeldt-Jakob disease (sCJD) using current diagnostic criteria.

**Materials and Methods**: Demographic and laboratory data were examined for cerebrospinal fluid (CSF) specimens submitted to the National Prion Disease Pathology Surveillance Center (NPDPSC) from May 2015 to April 2018. In cases with multiple specimen submission, only the first specimen received was included in these analyses. Second generation RT-QuIC using truncated hamster prion protein substrate was performed on all specimens. Neuropathologic examination at autopsy was used as the gold standard comparator. Autopsy is biased in favour of cases where the diagnosis is uncertain at death, namely when false positive or false negative RT-QuIC results are suspected. Autopsy tissue received through October 2018 was included in the analyses.

**Results**: The NPDPSC received CSF specimens from 10,498 living patients with suspected prion disease, and 567 (5.4%) subsequently underwent neuropathologic examination by the NPDSPC. Overall, 1,103 (10.5%) CSF specimens submitted to the NPDPSC were RT-QuIC positive. At autopsy, 497 (87.7%) cases were positive for prion disease, including 437 (77.4%) with definitive sCJD. Overall CSF RT-QuIC diagnostic sensitivity was 90.3% (449/497) and specificity was 98.5% (69/70). Sensitivity was highest in sCJD (92.9%; 406/437) compared to genetic prion diseases (82%; 31/38). RT-QuIC was negative in all cases of sporadic fatal insomnia (sFI, *n* = 5), sCJD VV1 (*n* = 3), and fatal familial insomnia (FFI, *n* = 4). Younger subjects were more likely to have false negative RT-QuIC results. One false positive RT-QuIC test occurred in a case of multifactorial dementia (Alzheimer’s disease and vascular).

**Conclusions**: We present analyses of a large cohort of CSF specimens and autopsy tissue submitted to the NPDPSC that includes high variability in demographics and prion disease subtypes. RT-QuIC demonstrated its highest sensitivity when testing CSF from older individuals and individuals with common forms of sCJD. RT-QuIC demonstrated poor sensitivity in detecting some uncommon prion disease subtypes (sFI, sCJD VV1, and FFI). The high specificity of RT-QuIC suggests that counting individuals who are positive for RT-QuIC in public health surveillance case numbers of probable/definitive prion disease, and antemortem CSF testing may foster improved prion disease surveillance.

## 68. Bioassays reveal Aβ and tau prions decline with longevity in Alzheimer’s disease

Carlo Condello^a,b^, Atsushi Aoyagi^a,c^, Jan Stöhr^a,b,d^, Weizhou Yue^a^, Brianna M. Rivera^a^, Joanne C. Lee^a^, Amanda L. Woerman^a,b^, Glenda Halliday^e^, Sjoerd van Duinen^f^, Martin Ingelsson^g^, Lars Lannfelt^g^, Caroline Graff^h,i^, Thomas D. Bird^j,k^, C. Dirk Keene^l^, William W. Seeley^b,m^, William F. DeGrado^a,n^ and Stanley B. Prusiner^a,b,o^

^a^Institute for Neurodegenerative Diseases, Weill Institute for Neurosciences, University of California, San Francisco, CA, USA; ^b^Department of Neurology, Weill Institute for Neurosciences, University of California, San Francisco, CA, USA; ^c^Daiichi Sankyo Co., Ltd., Tokyo, Japan; ^d^AC Immune SA, Lausanne, Switzerland; ^e^School of Medical Science, University of New South Wales, Sydney, Australia; ^f^Leiden University Medical Center, Leiden, The Netherlands; ^g^Department of Public Health and Caring Services/Geriatrics, Uppsala University, Uppsala, Sweden; ^h^Department of Neurobiology, Care Sciences and Society, Karolinska Institute, Huddinge, Sweden; ^i^Department of Geriatric Medicine, Karolinska University Hospital, Stockholm, Sweden; ^j^Department of Medicine, Division of Medical Genetics, University of Washington, Seattle, WA, USA; ^k^Department of Neurology, University of Washington, Seattle, WA, USA; ^l^Department of Neuropathology, University of Washington School of Medicine, Seattle, WA, USA; ^m^Department of Pathology, University of California, San Francisco, CA, USA; ^n^Department of Pharmaceutical Chemistry, University of California, San Francisco, CA, USA; °Department of Biochemistry and Biophysics, University of California, San Francisco, CA, USA.

**CONTACT** Carlo Condello carlo.condello@ucsf.edu

**ABSTRACT**

We present evidence that both the Aβ and tau proteins misfold into prions leading to Alzheimer’s disease (AD), which is a sporadic or genetic dementing disorder but not communicable or contagious. Since the progression of AD correlates poorly with insoluble Aβ in the central nervous system (CNS), it was difficult to distinguish between inert amyloids and Aβ prions. To measure the progression of AD, we devised exquisitely selective and sensitive bioassays to measure the abundance of isoform-specific Aβ prions in human CNS samples from patients with AD or other neurodegenerative diseases. Although brains from all human AD patients had measurable Aβ prions at death, the oldest individuals had lower Aβ prion levels. Long-lived individuals had low tau prion levels that correlated with phosphorylated tau. Surprisingly, the longevity-dependent decrease in tau prions occurred in spite of increasing amounts of total insoluble tau. When corrected for the abundance of insoluble tau, the tau prions decreased exponentially with respect to the age of death with a half-time of approximately one decade, and this correlated with the abundance of phosphorylated tau. Our findings with tau prions were not only unexpected but also counterintuitive; thus, tau phosphorylation and tau prion activity decreased exponentially with longevity in patients with AD ranging from ages 37 to 99 years. Our findings demonstrate an inverse correlation between longevity in AD patients and the abundance of neurotoxic tau prions. Moreover, our discovery may have profound implications for the selection of phenotypically distinct patient populations and development of diagnostics and effective therapeutics for AD.

## 69. Why is there a decline in the incidence of sporadic Creutzfeldt Jakob Disease in the older adult population?

**Investigating mixed pathologies in sporadic Creutzfeldt Jakob Disease**

Jane Lumsden, Bob Will, Colin Smith and Suvankar Pal

National CJD Research and Surveillance Unit, University of Edinburgh

**ABSTRACT**

**Background**: Sporadic Creutzfeldt Jakob Disease (sCJD) is a rapidly progressive and fatal neurodegenerative disorder and the commonest occurring form of human prion disease. Age-specific mortality rates for sCJD have increased up to ages 65–79 years over the past four decades in the United Kingdom. Of considerable interest is an apparent reduced incidence at ages of 80 and over. A similar phenomenon has been observed in other European countries. This reduced incidence could be due to an under ascertainment in the most elderly. It has also been recently hypothesized that the apparent decline in incidence of sCJD in older adults could be due to the inhibitory effects of the Alzheimer’s disease associated amyloid β-protein(A β) on prion propagation. Aβ deposition is common in advanced age, even in cognitively asymptomatic individuals, and a recent study suggested that soluble aggregates of Aβ can compete with prions for binding to misfolded cellular prion protein. This study raised the possibility that presence of Alzheimer’s disease pathology may be protective against CJD. Another recent neuropathological study investigating 30 cases with confirmed sCJD reported that co-morbid presence of abnormal proteins associated with other neurodegenerative disorders including Alzheimer’s disease, amyloid angiopathy, Lewy body dementia, argyrophilic grain disease, and progressive supranuclear palsy was found to be significantly associated with prolonged survival. This led authors to suggest that presence of dual/multiple neurodegenerative pathologies may be contributing to this phenomenon. Furthermore, additional work has demonstrated that sCJD cases with abundant deposits of Aβ present at a higher median age of disease onset and have longer disease duration. This project proposal identifies aims and hypotheses for this ongoing project.

**Aims**:

1. To review UK postmortem reports of patients with confirmed sCJD to establish presence of β-amyloid, neurofibrillary tangles, neuritic plaques, α synuclein, amyloid angiopathy, and record Thal phase and Braak stage.
2. To review clinical details of these cases including clinical co-morbidities, age at onset of sCJD, presenting symptoms, disease duration, codon 129 status, MRI findings, 14–3-3, and RT-QuIC results
3. To compare clinical features and investigation results of cases with dual/multiple co-pathologies with cases with no known co-pathology.

**Hypotheses**:

1. Cases with neuropatholgical evidence of dual or multiple pathologies will present differently to cases with isolated sCJD pathology.
2. MRI scans will show focal atrophy, or vascular burden which may confound the typical cortical and basal ganglia changes seen in sCJD.
3. CSF 14–3-3 and RT-QuIC results will differ. Specifically, 14–3-3 will be negative in cases of dual/multiple pathologies.
4. Individual co-pathologies will influence the clinical features and investigation results in a differential manner.
5. Cases with significant Lewy body deposition will likely test positive for alpha-synuclein in CSF.

## 70. A histidine swap in the prion protein’s Copper Center Drives a neuroprotective cis-interaction

Kevin Schilling^a^, Lizhi Tao^b^, Bei Wu^c^, Glenn Millhauser^a^, David Britt^b^ and David Harris^c^

^a^Department of Chemistry & Biochemistry, UC Santa Cruz, Santa Cruz CA, USA; ^b^Department of Chemistry, UC Davis, Davis, CA, USA; ^c^Department of Biochemistry, Boston University School of Medicine, Boston, MA, USA

**CONTACT** Kevin Schilling kschilli@ucsc.edu

**ABSTRACT**

The cellular prion protein (PrPC) is comprised of two domains – a globular C- terminal domain and an unstructured N-terminal domain. Recently, copper has been observed to drive tertiary structure in PrPC, inducing a *cis-*interaction between the two domains of the protein.1–3 The location of this interaction on the C-terminus overlaps with the sites of PrPSc docking, toxic antibody docking, and pathogenic mutations. Combined with recent evidence that the N-terminus is a toxic effector regulated by the C-terminus,4,5 there is a significant possibility that this *cis-*interaction serves a protective role, and that the disruption of this interaction by PrPSc oligomers is the cause of toxicity in prion disease. We demonstrate here that two highly conserved histidines in the C-terminal domain of PrPC are essential for the protein’s *cis-*interaction, which helps to protect against neurotoxicity carried out by its N-terminus. We show that mutation of these histidines drastically weakens the *cis-*interaction and biases cultured cells towards toxic events – the first C-terminal mutation to do so. Mechanistically, we propose an equatorial swap – a copper bound histidine from the N-terminus of the protein is replaced by a histidine from its C-terminus, forming a tether that holds the two domains together. We also find that extra N-terminal histidines in pathological familial mutations involving octarepeat expansions inhibit this interaction by stealing copper from the C-terminus, suggesting a mechanism for the toxicity of these mutants. We believe we have found the structural basis for PrPC’s C-terminal regulation of its N-terminus, and that this mechanism is responsible for preventing neurotoxicity in the properly functioning form of the protein.

### 

References[1]Thakur, et al. JBC
2011; 286:38,533–38,545.[2]Spevacek, et al. Structure
2013; 21:236–246.[3]Evans, et al. Structure
2016; 24:1057–10674.[4]Sonati, et al. Nature
2013; 501:102–106.[5]Wu, et al.
eLife
2017. doi:10.7554/eLife.23,473.001

## 71. Challenging assumptions: an empirical analysis of hunter response to chronic wasting disease in Alberta

John K. Pattison-Williams^a^, Lusi Xie^a^, Vic (W.L.) Adamowicz^a^, Margo Pybus^b^ and Anne Hubbs^b^

^a^Department of Resource Economics and Environmental Sociology, University of Alberta, Edmonton, Alberta, Canada; ^b^Alberta Environment and Parks, Edmonton, Alberta, Canada

**CONTACT** John K. Pattison-Williams johnp@ualberta.ca

**ABSTRACT**

**Background**: Chronic Wasting Disease (CWD) has fundamentally impacted wildlife management in North America. An integral partner in CWD management strategies is the hunting community, whose membership contributes in surveillance, harvest, and policy development. A conventional assumption has been that hunters will avoid or reduce hunting in areas where CWD is identified as high prevalence due to potential human health risks [1], requiring policy incentives to promote continued or increased hunting in these areas. However, this assumption has recently been challenged [2].

**Methods**: Using mule deer hunter draw information and CWD surveillance data from the Alberta Ministry of Environment and Parks (AEP), we empirically explore the response of mule deer hunters to CWD using license application trends in wildlife management units (WMUs) where CWD has been positively detected. Using a fixed effects (FE) approach, we model the relationship between total resident draw applications and covariates of CWD head counts, quotas, draw success rates between 2005 and 2016 in 39 WMUs.

**Results**: Our model indicates that mule deer hunters in Alberta are continuing to hunt in WMUs with high CWD prevalence and no significant relationship exists between CWD count and draw applications. These results suggest that (i) hunter decisions regarding license applications are not significantly affected by CWD presence or prevalence, or (ii) hunters perceive CWD in a WMU as a factor that does not detract from, and may be attractive for hunting opportunities.

**Conclusions**: Our results have important policy implications for CWD management. First, it challenges the assumption that hunters are avoiding CWD zones. Second, it suggests that alternative hunter-based strategies – such as expanded hunting seasons, increased tags or replacement tags – may be more effective and less costly than previously thought to manage the disease. One caveat is that our analysis is based on analyses of aggregate demand for hunting licenses at the WMU level, rather than individual hunter demands over time.

**KEYWORDS:** Chronic wasting disease; recreational hunting; cervids; alberta; mule deer; wildlife management

### 

References[1]Cooney, et al. Hum Dimens Wildl
2010; 15: 194–207.[2]Haus, et al. Wildl Soc Bull
2017; 41:294–300.

## 72. miR-146a regulates cellular and disease associated prion protein isoforms during prion propagation

Wenting Zhao^a,b^, Cathryn Ugalde^a^, Steven Collins^c^, Vicki Lawson^d^, Lesley Cheng^a^, Shayne Bellingham^b^ and Andrew Hill^a^

^a^Department of Biochemistry and Genetics, La Trobe Institute for Molecular Science, La Trobe University, VIC, Australia; ^b^Department of Biochemistry and Molecular Biology, The University of Melbourne, VIC, Australia; ^c^Florey Institute of Neuroscience and Mental Health, The University of Melbourne, VIC, Australia; ^d^Department of Microbiology and Immunology, The Peter Doherty Institute for Infection and Immunity, The University of Melbourne, VIC, Australia.

**CONTACT** Wenting Zhao wenting.zhao@latrobe.edu.au

**ABSTRACT**

Prion diseases are transmissible neurodegenerative disorders characterized by the structural transformation of the cellular prion protein (PrP^C^) to the prion disease associated isoform (PrP^Sc^). MicroRNAs (miRNAs) are a class of small non-coding RNAs that regulate gene or protein expression by targeting mRNAs and triggering either translational repression or mRNA degradation. Distinct miRNA signatures have been detected in prion disease models and patients, serving as potential disease diagnostic biomarkers. In this study, increased expression levels of miR-146a, an anti-inflammatory miRNA known to be dysregulated in prion diseases, were detected in four prion disease models: in prion strain M1000 infected neuronal N2a cells; at day 42 and day 70 of M1000 infected organotypic brain slices; at week 17 and terminal stages of M1000 infected mouse brains; and in the post-mortem brain tissue samples from sporadic Creutzfeldt-Jakob disease (sCJD) patients. CRISPR/Cas9 mediated miRNA editing and prion infection were further used to examine the effects of miR-146a gain/loss-of-function on prion proteins in cell culture and organotypic brain slice culture models. miR-146a was shown to increase PrP^C^ levels when overexpressed or downregulated in N2a cells. The prion protein encoding gene (Prnp) was shown to be targeted by miR-146a in the 3’ untranslated region (3’ UTR). miR-146a overexpression decreased the amount of PrP^Sc^ in M1000 infected N2a cells, while M1000 infected organotypic brain slices from miR-146a knockout mice exhibited increased PrP^Sc^ levels compared to the wildtype controls. Overall, the protective role of miR-146a was revealed in prion diseases by regulating cellular and disease associated prion proteins, suggesting its therapeutic potential.

## 73. Transgenic overexpression of the PrP N1 fragment does not protect against prion disease or Aβ toxicity due to impaired ER translocation

Behnam Mohammadi^a^, Luise Linsenmeier^a^, Mohsin Shafiq^a^, Berta Puig^a^, Giovanna Galliciotti^a^, Jörg Tatzelt^b^, Markus Glatzel^a^* and Hermann C. Altmeppen^a^*

^a^Institute of Neuropathology, University Medical Center Hamburg-Eppendorf (UKE), Hamburg, Germany; ^b^Biochemistry of Neurodegenerative Diseases, Institute of Biochemistry and Pathobiochemistry, Ruhr University Bochum, Bochum, Germany

**CONTACT** Behnam Mohammadi b.mohammadi@uke.de

*These authors contributed equally to this work.

**ABSTRACT**

The highly conserved and constitutively active endogenous α-cleavage of the prion protein has so far mostly been linked with protective effects. As a soluble factor, the released unstructured N1 fragment acts beneficial in several ways as it, for instance, reduces hypoxia-induced neuronal damage and may be involved in myelin maintenance and other aspects of intercellular communication. With regard to neurodegenerative diseases, numerous studies have shown that N1 is able to block toxic oligomers, such as Aβ in Alzheimer`s disease, and interferes with their synaptic impairment and neurotoxicity.

For prion diseases, however, insight into a potentially protective role of N1 by similarly neutralizing PrP^Sc^ oligomers and interfering with prion conversion is lacking to date. Since the responsible protease for the α-cleavage has not been identified unequivocally, pharmacological targeting of this entity is not possible to date. We therefore decided to directly address this issue *in vivo* by generating transgenic mice (tgN1) overexpressing N1 on a wild-type background and challenging them with RML prions. Despite moderate differences in PrP^Sc^ formation and p38 activation, incubation time, and disease duration was similar between tgN1 mice and wild-type littermate controls. Biochemical and morphological assessment of brain samples, primary neurons, and cell culture models then revealed, that our direct ‘protective’ strategy failed due to cytosolic accumulation and lack of secretion of the transgenic N1. Nevertheless, our study provides the first *in vivo* proof of the recently described impaired translocation of intrinsically disordered peptides into the endoplasmic reticulum. Moreover, it demonstrates effects of cytosolic accumulation of N1 with uncleaved signal peptide, addresses proteasomal degradation, questions a general relevance of cytosolic prions in prion diseases, and highlights important aspects to be considered when investigating the α-cleavage of PrP^C^ or devising N1-based therapeutic approaches.

## 74. Characterization of gene expression profiles in brains of mice infected with typical and atypical BSEs

Takeshi Yamasaki^a^, Akio Suzuki^a^, Rie Hasebe^b^, Yuichi Matsuura^c^, Kohtaro Miyazawa^c^, Yoshifumi Iwamaru^c^ and Motohiro Horiuchi^a^

^a^Laboratory of Veterinary Hygiene, Faculty of Veterinary Medicine, Hokkaido University, Japan; ^b^Institute for Genetic Medicine, Hokkaido University, Japan; ^c^National Institute of Animal Health, National Agriculture and Food Research Organization (NARO), Japan

**CONTACT** Takeshi Yamasaki Yamasaki@vetmed.hokudai.ac.jp

**ABSTRACT**

Transgenic mice overexpressing bovine PrP (TgBoPrP) infected with typical BSE (C-BSE), atypical L-type BSE, and H-type BSE (L-BSE and H-BSE) show different clinical and neuropathological characteristics. To understand host responses that contribute to the differences in the phenotypes, we analysed gene expression profiles in brains of TgBoPrP mice infected with C-BSE, L-BSE, and H-BSE. We performed a transcriptome analysis by RNA-sequencing on brainstems of the mice at pre-clinical, clinical, and terminal stages of infection and those of age-matched un-infected mice. First, we performed hierarchical clustering of samples based on gene expression data using 16,894 genes that code proteins. The samples from mice infected with L-BSE at 198 days post intracerebral inoculation (dpi), C-BSE at 239 and 253 dpi, and H-BSE at 291, 319 and 362 dpi were clearly separated from cluster containing un-infected samples, suggesting that host responses accompanied with marked transcriptomic changes initiated by 198, 239, and 291 dpi in L-BSE, C-BSE and H-BSE infected mice, respectively. Next, we identified 3,219 differentially expressed genes (DEGs) that showed over 2-fold change in expression level between infected and un-infected mice. By applying K-means clustering to the DEGs, we found four characteristic gene clusters that contain genes of which expression was upregulated by infection. Three clusters contained 338, 517 and 294 upregulated genes unique to C-BSE, L-BSE, and H-BSE, respectively, and the other cluster contained 251 upregulated genes common to all BSEs. Gene ontology (GO) analysis based on Metascape database revealed that genes related to immune responses were highly enriched in the upregulated genes common to all BSEs. Although enrichment scores were not so high, the upregulated genes unique to each BSE showed a significant enrichment of GO terms related to regulation of immune system. These data might suggest the presence of immune responses both common and unique to C-BSE, L-BSE, and H-BSE infection.

## 75. Mortality surveillance of individuals potentially exposed to chronic wasting disease

Ryan A. Maddox^a^, Rachel F. Klos^b^, Suzanne N. Gibbons-Burgener^b^, Bobbi L. Bryant^a^, Joseph Y. Abrams^a^, Brian S. Appleby^c^, Lawrence B. Schonberger^a^ and Ermias D. Belay^a^

^a^National Center for Emerging and Zoonotic Infectious Diseases, Centers for Disease Control and Prevention (CDC), Atlanta, GA, USA; ^b^Wisconsin Department of Health Services, Division of Public Health, Madison, WI, USA; ^c^National Prion Disease Pathology Surveillance Center (NPDPSC), Case Western Reserve University, Cleveland, OH, USA

**CONTACT** Ryan A. Maddox rmaddox@cdc.gov

**ABSTRACT**

**Introduction**: Chronic wasting disease (CWD) is a prion disease of cervids. It is unknown whether CWD prions can infect people; if so, transmission would most likely occur through consumption of meat from infected animals. Since 2003, Wisconsin Department of Health Services, Division of Public Health (WDHS) personnel have maintained a database consisting of information collected from hunters who reported eating, or an intention to eat, venison from cervids positive for CWD. This data source makes it possible to evaluate causes of mortality in individuals potentially exposed to CWD.

**Methods**: The WDHS database contains the name, date of birth, when available, year of CWD-positive deer harvest, and city and state of residence for each potentially exposed individual. The database also includes information on how the deer was processed (self-processed or by a commercial operator) and when applicable, names of others with whom the venison was shared. Duplicate entries (i.e. those who consumed venison from CWD-positive deer in multiple hunt years) are determined by first name, last name, and date of birth. All names in the database are cross-checked with the National Prion Disease Pathology Surveillance Center (NPDPSC) neuropathology database. Vital status of individuals with date of birth available will be tracked through the identification of possible matches in the National Death Index (NDI) and the evaluation of corresponding cause of death codes.

**Results**: The database consists of 1561 records for hunt years 2003–2017. Of these, 613 records had accompanying date of birth; 14 entries were removed as duplicates, 1 of whom had consumed venison from a CWD-positive deer during three different hunt years, leaving 599 unique individuals for pending submission to NDI. Of these individuals, 265 of 399 (66%) who ate venison from a CWD-positive deer and provided processing information reported self-processing. No matches were found among persons in the database cross-checked with NPDPSC data.

**Conclusion**: Because of the robust data link between person and CWD-positive animal, reviewing the cause of mortality in potentially exposed persons is possible; those individuals who self-processed and consumed the meat are likely the best source of information about the potential for zoonotic transmission. The expected long incubation period, should transmission to humans occur, necessitates many years of vital status tracking.

## 76. Prion disease incidence, United States, 2003–2016

Ryan A. Maddox^a^, Marissa K. Person^a^, Janis E. Blevins^b^, Joseph Y. Abrams^a^, Bobbi L. Bryant^a^, Brian S. Appleby^b^, Lawrence B. Schonberger^a^ and Ermias D. Belay^a^

^a^National Center for Emerging and Zoonotic Infectious Diseases, Centers for Disease Control and Prevention (CDC), Atlanta, GA, USA; ^b^National Prion Disease Pathology Surveillance Center (NPDPSC), Case Western Reserve University, Cleveland, OH, USA

**CONTACT** Ryan A. Maddox rmaddox@cdc.gov

**ABSTRACT**

**Introduction**: Mortality data and the results of neuropathological and genetic testing are used to estimate the incidence of prion diseases in the United States.

**Methods**: Prion disease decedents were identified from restricted-use U.S. national multiple cause-of-death data, via a data use agreement with the National Center for Health Statistics, and from the National Prion Disease Pathology Surveillance Center (NPDPSC) database for 2003–2016. NPDPSC decedents with neuropathological or genetic test results positive for prion disease for whom no likely match was found in the multiple cause-of-death data were added as cases for incidence calculations; those with negative neuropathology results but with cause-of-death data indicating prion disease were removed. Age-adjusted average annual incidence rates for the combined data were calculated using the year 2000 as the standard population.

**Results**: A total of 5737 decedents were identified as having prion disease during 2003–2016 for an age-adjusted average annual incidence of 1.2 per million populations. The age-adjusted annual incidence ranged from 1.0 per million in 2004 and 2006 to 1.4 per million in 2013; there was an increasing trend in age-adjusted incidence over the entire period (*p* <0.0001) but no significant increase during the second half (2010–2016, *p* = 0.08). The age-adjusted incidence between males and females (1.3 and 1.1 per million, respectively) differed significantly (*p* <0.0001). Twelve cases during 2003–2016 were <30 years of age for an age-specific incidence of 6.9 per billion; only two of these very young cases were sporadic, with the rest being familial (7), variant (2), or iatrogenic (1). The age-specific average annual incidence among those <55 and ≥55 during the time period was 0.2 and 4.7 per million, respectively; incidence among those ≥65 was 5.9 per million.

**Conclusion**: The incidence of prion diseases, which are invariably fatal, can be estimated through analysis of mortality data supplemented with the results of neuropathological and genetic testing. Incidence in the United States over the last 7 years appears to be relatively stable. Cases <30 years of age continue to be very rare and usually indicate an exogenous source of infection or the presence of a genetic mutation.

## 77. Assessing chronic wasting disease strain differences in free-ranging cervids across the United States

Kaitlyn M. Wagner^a^, Caitlin Ott-Conn^b^, Kelly Straka^b^, Bob Dittmar^c^, Jasmine Batten^d^, Robyn Pierce^a^, Mercedes Hennessy^a^, Elizabeth Gordon^a^, Brett Israel^a^, Jenn Ballard^e^ and Mark D Zabel^a^

^a^Prion Research Center at Colorado State University; ^b^Michigan Department of Natural Resources; ^c^Texas Parks and Wildlife Department; ^d^Missouri Department of Conservation, 5. Arkansas Game and Fish Commission

**CONTACT** Kaitlyn M. Wagner miedkait@rams.colostate.edu

**ABSTRACT**

**Background/Introduction:** Chronic wasting disease (CWD) is an invariably fatal prion disease affecting captive and free-ranging cervids, including white-tailed deer, mule deer, moose, elk, and reindeer. Since the initial description of the disease in the 1960’s, CWD has spread to 23 states, 3 Canadian Provinces, South Korea, Norway and, most recently, Finland. While some outbreaks of CWD were caused by transport of infected animals from endemic regions, the origin of CWD in other epizootics is unclear and has not been characterized. Previous studies have shown that there are two distinct strains of CWD. However, the continuous spread and the unclear origin of several outbreaks warrant continued surveillance and further characterization of strain diversity.

**Materials and Methods:** To address these knowledge gaps, we used biochemical tests to assess strain differences between CWD outbreaks in Michigan, Texas, Missouri, and Colorado, USA. Brain or lymph node samples were homogenized and digested in 50 µg/mL proteinase K (PK). These samples were then run on a Western blot to assess glycoform ratio and electrophoretic mobility. Texas samples were digested in 100 µg/mL PK. To assess conformational stability, brain or lymph node homogenates were incubated in increasing concentrations of guanidine hydrochloride from 0 M to 4 M in 0.5 M increments. Samples were then precipitated in methanol overnight, washed and PK digested in 50 µg/mL PK before slot blotting.

**Results:** Our results have found significant differences in glycoform ratio between CWD from Michigan and Colorado, but no differences were observed in conformational stability assays. Interestingly, when testing our CWD isolates from Texas to analyse electrophoretic mobility and glycoform ratio, we found that these samples did not exhibit the characteristic band shift when treated with PK, but PK resistant material remained. Additionally, results from our conformational stability assay demonstrate a unique profile of these Texas isolates. Testing of samples from Missouri is currently underway.

**Conclusions:** Thus far, our data indicate that there are strain differences between CWD circulating in Michigan and CWD in Colorado and provide important insight into CWD strain differences between two non-contiguous outbreaks. We have also identified a unique strain of CWD in Texas with biochemical strain properties not seen in any of our other CWD isolates. These results highlight the importance of continued surveillance to better understand this devastating disease. These results have important implications for CWD emergence, evolution and our understanding of prion strain heterogeneity on the landscape.

## 78. Optogenetically stimulation of medial septum-diagonal band complex ameliorates the cognitive dysfunctions of APP^NL-G-F^, mouse model of Alzheimer’s disease

Jogender Mehla, Surjeet Singh, Robert J. McDonald and Majid H. Mohajerani

Canadian Centre for Behavioural Neuroscience, University of Lethbridge, Lethbridge, Alberta, Canada.

**CONTACT** Jogender Mehla jogender.mehla@uleth.cal; jsmehlaaiims@gmail.com

**ABSTRACT**

**Introduction**: Cholinergic neuron degeneration in the basal forebrain is known to be one of the initial pathological hallmarks of Alzheimer’s disease (AD). Additionally, currently available drug therapy is also based on the cholinergic hypothesis which provides only symptomatic treatment. Therefore, considering the importance of cholinergic system in AD, we planned this study to investigate the effect of chronic optogenetic stimulation of medial septum-diagonal band (MSDB) complex, a part of basal forebrain on the cognitive deficits and amyloid pathology in knock-in mouse model of AD.

**Materials and Methods**: In the present study, we used male APP^NL-G-F^ mice. We used male C57BL/6J mice as a normal control. We injected channelrhodopsin virus and implanted optrodes into MSDB complex of 2 months old APP^NL-G-F^ mice. After one month of surgery, these mice were divided into two groups; one, where no optogenetic stimulation was done and second, in which MSDB stimulation was performed for 3 months using blue light (473 nm, 3–4 mW). After end of 3 months stimulation, Morris water maze (MWM) and fear conditioning tests were performed for the assessment of learning and memory functions. Lastly, we perfused the mice and brains were extracted for immunostaining.

**Results**: The results show that optogenetically stimulation of MSDB improved the learning ability of APP^NL-G-F^ mice in MWM evidenced by significant decrease in escape latency on day 8 in comparison with APP^NL-G-F^ sham group. Furthermore, optogenetic stimulated mice spent significantly more time in target quadrant as compared to average of other quadrants. Nonetheless, optogenetically stimulated mice also spent significantly more time in target quadrant in comparison with APP^NL-G-F^ sham group indicating an improvement in memory retention. APP^NL-G-F^ mice also showed an improvement in memory function in tone and contextual fear conditioning tests. The chronic optogeneticallly stimulation of MSDB reduced the amyloid burden in cortex and hippocampus of APP^NL-G-F^ mice which may be responsible for improvement in cognitive functions.

**Conclusion**: The findings from the present study support the potential of cholinergic hypothesis and optogenetics for the treatment of AD.

## 79. The FXR1 protein is a functional amyloid of mammalian brain

A. V. Sergeeva^a^, M. E. Velizhanina^a^, J. V. Sopova^a^^,^^b^, E. I. Koshel^c^, T. A. Belashova^b^, S. P. Zadorsky^a^^,^^b^, V. A. Siniukova^b^ and A. P. Galkin^a^^,^^b^

^a^Department of Genetics and Biotechnology, St. Petersburg State University, Petersburg, Russian Federation; ^b^Vavilov Institute of General Genetics, St. Petersburg Branch, Russian Academy of Sciences, Petersburg, Russian Federation; ^c^Department of Cytology and Histology, St. Petersburg State University, St. Petersburg, Russian Federation

**CONTACT** A. V. Sergeeva Aleksandra.helwig@yandex.ru

Amyloids are non-branching fibrils that are composed of stacked monomers stabilized by intermolecular β-sheets. Our novel proteomic approach PSIA-LC-MALDI separates amyloids from the majority of other protein complexes of a non-amyloid nature. Being applied to *R. norvegicus* brain proteome, PSIA-LC-MALDI allowed identification of several amyloid-like proteins forming SDS-resistant aggregates. FXR1 was among identified proteins. FXR1 binds and regulates translation of target mRNAs and takes part in memory and emotions control. We demonstrated that FXR1 forms amyloid conformers that strongly co-localize with thioflavin S, bind RNA and prevent its RNAseA-mediated degradation in the cortex neurons of the rat brain. Furthermore, we showed that N-terminal region of the FXR1 protein underlies the ability of full-length FXR1 to form SDS-resistant oligomers and insoluble aggregates in the brains of young and healthy rat males and forms amyloid fibrils *in vitro*. Moreover, *in vitro* assay confirmed the ability of amyloid conformers of the N-terminal region of FXR1 to bind poly(A)-RNA extracted from the rat brain. To further evaluate the amyloidogenic properties of FXR1 N-terminal region we used bacterial-based C-DAG system. Not to our surprise, FXR1(1–380) forms extracellular fibrils in bacteria, that can be visualized by TEM and demonstrates apple-green birefringence when stained with Congo Red and viewed under crossed polarizers. Specification of FXR1’s amyloidogenic region boundaries allowed us to shorten it from (aa 1–380) to (aa 1–270), as FXR1(1–270) demonstrates same amyloidogenic properties as FXR1(1–380) in C-DAG system. To shed the light on the possibility of the FXR1 protein being a functional amyloid of human brain as well, we used a human neuroblastoma cell line, IMR32, as a human brain model. We demonstrated that in human neuroblastoma cells FXR1 forms insoluble aggregates of high molecular mass the abundance of which is controlled in serum-dependent manner. Considering the fact that serum starvation is one of the standard methods of inducing neuroblastoma cells differentiation, we speculate that FXR1 oligomers and amyloid aggregates may play a role in cell differentiation via its interaction with mRNAs of cell cycle proteins, such as p21. Interestingly, the N-terminal region of FXR1 is highly conserved across mammals and contains identically arranged potentially amyloidogenic sequences in evolutionary distinct vertebrates. These data support our hypothesis that the ability of FXR1 to function in amyloid form is conserved in evolution. The reported study was funded by RFBR according to the research project 18–34-00420 and grant of SpbSU to Laboratory of Amyloid Biology.

## 80. Cellular prion protein promotes cell metabolic orientation towards the oxidative degradation of glucose

H. Arnould^a^, A. Baudry^a^, M. Pietri^a^, A. M. Haeberlé^b^,M. Laforge^a^, G. Bertho^c^, G. Schmitt-Ulms^d^, Y. Bailly^b^,O. Kellermann^a^ and B. Schneider^a^

^a^INSERM UMR 1124, Université Paris Descartes, Paris, France; ^b^CNRS UPR 3212, Institut des Neurosciences Cellulaires et Intégratives, Strasbourg, France; ^c^CNRS UMR 8601, Université Paris Descartes, Paris, France; ^d^Tanz Centre for Research in Neurodegenerative Diseases, University of Toronto, Canada

**CONTACT** H. Arnould helene.arnould@parisdescartes.fr

While the implication of cellular prion protein PrP^C^ has been widely documented in prion diseases, the role(s) of this ubiquitous protein still remain(s) elusive. To address this issue, PrP^C^ was chronically silenced in the 1C11 neuronal cell line, a neuronal stem cell endowed with the capacity to differentiate into serotonergic or noradrenergic neurons. A global analysis was performed to compare the proteome of PrP^C^-depleted 1C11 cells (referred to as PrPnull-1C11 cells) to that of parental PrP^C^-expressing 1C11 cells with the aim to identify cell function(s) deregulated by the absence of PrP^C^. Our proteomic analysis reveals strong abnormalities in the expression level of enzymes at play in the glucose energetic metabolism in PrPnull-1C11 cells with a reduced expression of mitochondrial enzymes in favour of the enzymes involved in the glycolytic-fermentative pathway. Surprisingly, functional experiments by NMR and Seahorse approaches to probe metabolic fluxes in cells indicate reduced glycolytic and lactic fermentative fluxes as well as decreased mitochondria oxygen consumption in PrPnull-1C11 cells when grown in a medium containing glucose. Global reduction of glucose oxidative degradation in PrP^C^-depleted cells is associated with down-expression of the glucose transporter GLUT3 and down-regulation of pyruvate dehydrogenase (PDHA1) activity that normally promotes the entry of pyruvate into mitochondria and transformation into acetyl-CoA. Finally, we show a metabolic shift in PrPnull-1C11 cells with a preferred orientation of the energetic metabolism towards fatty acid degradation by the β-oxidation pathway to maintain ATP concentration at high levels. The flip side of such conversion in the energetic pathways is the onset of oxidative stress conditions in PrP^C^-depleted cells. For the first time, our work provides evidence that PrP^C^ plays a key role in the energetic metabolism by potentiating glucose oxidative degradation through the glycolysis- mitochondria pathway. As corruption of PrP^C^ function(s) by pathogenic prions is at the root of prion diseases, any perturbation of PrP^C^ control of glucose degradation in prion-infected cells may contribute to neurodegeneration.

**KEYWORDS:** Proteomics; PrP^C^ function; glucose and fatty acid energetic metabolisms; oxidative stress

## 81. Detection of neuronal dysfunction associated with genetic prion disease in human cerebral organoids

Simote T. Foliaki^a^, Bradley R. Groveman^a^, Aleksandar R. Wood^a^, Jue Yuan^b^, Leslie Cooperman^b^, Paul Tesar^b^, Wen-Quan Zou^b^ and Cathryn L. Haigh^a^

^a^National Institute of Allergy and Infectious Diseases, NIH; ^b^Case Western Reserve University School of Medicine

**CONTACT** Simote T. Foliaki simote.foliaki@nih.gov

**ABSTRACT**

**Introduction**: One of the fundamental problems of researching human prion diseases is access to live human brain tissue. Utilizing stem cell technology to produce human cerebral organoids (COs), which currently represent the nearest model to structured human brain tissue, offers a new avenue of investigation [1]. These organoids are self-organizing, structured regions analogous to human brain tissue that mature to contain multiple neuronal populations as well as astrocytes and oligodendrocytes [1, 2]. Importantly, these COs could be generated from donors who are carriers of mutations associated with familial prion diseases such as the E200K mutation. Herein we aimed to investigate the influence of E200K mutation that pre-dispose individuals to prion disease on neuronal electrical signalling.

**Methods**: COs have been generated from donors with E200K mutations (pre-disposed) and without (non-predisposed) mutations within the prion gene using the protocol described by Lancaster and Knoblick [1]. To measure the ability of these organoids to evoke spontaneous neuronal electrical signalling, they were sliced into 300 µm thick slices and mounted onto multi-electrode arrays (MEAs). MEAs are embedded with micro-electrodes to measure extracellular neuronal electrical spike (action potentials) and network communication throughout individual organoid. During the recording, the organoid slices were provided with adequate nutrients and oxygen through continuous superfusion with oxygenated artificial cerebrospinal fluid. To induce electrical signalling associated with cognition and behaviour, we exposed the slices to known stimulants and depressants of neuronal activity to determine their ability to express functions associated with memory formation and those related to memory loss [3, 4].

**Results and Conclusion**: We found that both non-predisposed and predisposed organoids exhibited active spontaneous neuronal function and network activity. Exposure of these organoids to glycine induced a persistently enhanced rate of neuronal spikes, a physiological correlate of memory formation called Long-Term Potentiation (LTP). Exposure of these organoids to glutamate/glycine induced LTP as well as Long-Term Depression (LTD), a physiological correlate of memory loss. Overall, we observed differences between the predisposed and non-predisposed organoids in neuronal excitability and response to depressants which may influence excitotoxicity events and level of fatigue in neuronal activity in these organoids.

### 

References[1]Lancaster, et al.
Nat Protoc. 2014;9(10):2329–2340.10.1038/nprot.2014.158PMC416065325188634[2]Renner, et al. Embo J
2017;36(10):1316–1329.10.15252/embj.201694700PMC543022528283582[3]Igartua, et al. Neuropharmacology
2007;52(8):1586–1595.10.1016/j.neuropharm.2007.03.00317462677[4]Hasegawa, et al.
Sci Rep. 2015;5:7707.10.1038/srep07707PMC464834925573377

## 82. The celecoxib derivative AR-12 induces autophagy and controls prion infection *in vitro* and *in vivo*

Basant A. Abdulrahman^a^^,^^b^, Dalia Abdelaziz^a^^,^^c^, Simrika Thapa^a^^,^^c^, Li Lu^a^^,^^c^^,^ Sabine Gilch^b^^,^^c^ and Hermann M. Schatzl^a^^,^^c^

^a^Department of Comparative Biology & Experimental Medicine, Faculty of Veterinary Medicine, University of Calgary, Calgary, Canada; ^b^Department of Ecosystem & Public Health, Faculty of Veterinary Medicine, University of Calgary, Calgary, Canada; ^c^Calgary Prion Research Unit, and Hotchkiss Brain Institute, University of Calgary, Calgary, Canada

**CONATCT** Basant A. Abdulrahman baabdulr@ucalgary.ca

**ABSTRACT**

Prion diseases are fatal infectious neurodegenerative disorders that affect both humans and animals. The autocatalytic conversion of the cellular prion protein (PrPC) into the pathologic isoform PrPSc is a key feature in prion pathogenesis. AR-12 is an IND-approved derivative of celecoxib that demonstrated preclinical activity against several microbial diseases. Loss of neurons, astrogliosis and mild microglia activation are the main pathological features of prion diseases. This results in a progressive spongiform degeneration of the central nervous system (CNS), leading to ataxia, behavioural changes and, in humans, highly progressive loss of intellectual abilities. In the last two decades, great efforts have been made to establish treatment options for prion diseases. These included testing existing drugs for anti-prion activity in experimental models with only a few agents progressing to human studies of patients with prion diseases. Investigations to date have not resulted in a recognized/proven treatment for prion diseases. Recently, AR-12 (a.k.a. OSU-03012), a potent inhibitor of PDK-1 (phosphoinositide-dependent kinase 1) and autophagy stimulator, has been shown to facilitate clearance of misfolded proteins. The latter proposes AR-12 to be a potential therapeutic agent for neurodegenerative disorders. In our study, we investigated the role of AR-12 in controlling prion infection. We tested AR-12 in prion infected neuronal cells (ScN2a, ScCAD5) and non-neuronal cells (ScMEF). Immunoblotting and confocal microscopy results showed that AR-12 significantly reduced PrPSc levels after only 72 h of treatment. Furthermore, infected cells were cured of PrPSc after exposure of AR-12 for only 2 weeks. We partially attribute the influence of the AR-12 on prion propagation to autophagy stimulation. Furthermore, we tested the therapeutic effect of AR-12 *in vivo* in RML-infected mice. Interestingly, treatment with AR-12 (intraperitoneally and orally) significantly extended the survival of the treated animals compared to the control group, in line with our previous findings that drug-induced stimulation of autophagy has anti-prion effects *in vitro*. Taken together, this study demonstrates that AR-12 represents a potential new therapeutic agent for prion diseases and possibly protein misfolding disorders involving prion-like mechanisms.

**KEYWORDS:** Prions; PrPSc; Autophagy; AR-12

## 83. Rationally designed, structure-based vaccine candidates targeting prion diseases

Andrew Fang^a^, Xinli Tang^a^, Brian Tancowny^a^, Xiongyao Wang^a^* and Holger Wille^a^

^a^Centre for Prions and Protein Folding Diseases & Department of Biochemistry, University of Alberta, Edmonton, Alberta, Canada

**CONTACT** Andrew Fang jfang5@ualberta.ca

*Present address: School of Materials Science and Engineering, Harbin Institute of Technology, Weihai, Shandong, China

**ABSTRACT**

Prion diseases are caused by the misfolding of PrP^C^ to PrP^Sc^, resulting in radical changes in secondary and tertiary structure. The lack of prophylactic or therapeutic vaccines means uncontrolled spread and an invariably fatal outcome for the host. No high-resolution structure for PrP^Sc^ exists despite it having been studied extensively, but there is good evidence to suggest a four-rung β-solenoid structure. HET-s is a fungal prion protein that shares the β-solenoid fold with PrP^Sc^, albeit with only two-rungs. A four-rung β- solenoid version of HET-s, termed HET-2s, was engineered via a linker connecting the prion-forming domain of HET-s twice. Strategic placement of seven PrP-based amino acids into HET-2s allowed us to mimic the surface structure of PrP^Sc^ and marks a new, structure-based approach in prion vaccine design. Constructs were expressed in E. coli, purified, and assembled into amyloid fibrils morphologically indistinguishable from native HET-s fibrils. Vaccine candidates showing good fibrillization, and therefore adopting the proper β-solenoid fold, were used for immunization experiments. We recently showed that one of our vaccine candidates resulted in a specific immune response towards native PrP^Sc^.

Efficacy testing using an oral prion infection model in Syrian hamsters is ongoing to evaluate whether the vaccine candidate is effective against peripheral infection routes. Monoclonal antibodies (mAbs) that originated from the immunized mice were shown to recognize native PrP^Sc^ in brain homogenates from all prion isolates that were tested. A competition ELISA that uses these mAbs is showing great potential as a diagnostic tool (See Tang *et al*. poster). Moreover, the ability of these mAbs to recognize native PrP^Sc^ from various strains also reveals shared structural characteristics (See Wille *et al*. poster). Furthermore, the IgM and IgG clones are being sequenced and revealed unique complementarity-determining regions, indicating they all have different epitopes (See Rathod *et al*. poster). Lastly, this approach has been used to also design vaccines against other amyloid diseases, specifically targeting misfolded Aβ, α-synuclein and tau protein (See Flores-Fernandez *et al*. poster). Taken together, our novel vaccine design moves us one step closer to develop passive and active immunotherapies for the prion diseases.

## 84. Stress granules “road to ruin’’ in neurodegenerative diseases

Neelam Younas, Saima Zafar and Inga Zerr

Department of Neurology, Clinical Dementia Centre, University Medical Centre Goettingen(UMG), Germany

**ABSTRACT**

**Introduction**: Stress granules are membrane less cytoplasmic foci of mRNA and RNA binding proteins that are formed in the cell to cope with variety of stressors. Emerging evidences have shown disturbances in the dynamics of these non-membranous foci, as a pathophysiological element of amyotrophic lateral sclerosis (ALS), frontotemporal dementia (FTD), and more recently in Alzheimer disease^1,2^. These evidences redirect research efforts towards understanding the role of stress granule biology in neurodegenerative diseases. In this study, we aim to explore the role of oxidative-stress to proteopathic disease linked proteins which are major hallmarks of neurodegenerative diseases.

**Methods**: For generation of Cell Stress Model, human cervical adenocarcinoma (HeLa) cell line was treated with 0.6mM sodium arsenite (a classical stress inducer) at 37°C for 60 min. Immunofluorescence was used for visualization of stress granules. Differential expression of selected target proteins was analysed in untreated (control) and treated cells (stressed) and in Human brain patients by Immunoblotting. Tau-Pathology Model was used to find out direct mechanistic link of target proteins to disease pathology.

**Results**: Interestingly, we found that aggregated prone proteins particularly phosphorylated form of Tau and Prion protein (Tau, PrP) were recruited to cytoplasmic stress granules after sodium arsenite treatment. Stress granules were identified by classical marker TIA-1 and PABP. To find out stress specific interactors of aggregated prone proteins interactome mapping of these proteins in untreated and stress induced cells revealed a common stress specific target, splicing factor proline, and glutamine rich (SFPQ) which is an important component of Neuronal transport granules. Here, we show that SFPQ is also a constituent of cytoplasmic stress granules. Arsenite treatment induced cytoplasmic SFPQ granules. Further to find out granule formation propensity of SFPQ, it was subjected to catGRANULE software which predicted a score of 1.66 showing a good propensity of SFPQ to form granules or liquid-liquid demixing. SFPQ was found to be significantly regulated in Alzheimer and CJD patient’s brain in subtype dependent manner.

**Conclusions**: Our results highlight novel association/localization of aggregation prone proteins with stress granules linking cellular stress response to pathobiology of these proteins. Further differential regulation of SFPQ, its LLPS property and its localization in stress granules and interaction with Tau and Prion Protein links dots for SFPQ as might be crucible of aggregation via stress granules.

Supported by Helmholtz-Alberta Initiative – Neurodegenerative Diseases Research (HAI-NDR); Alberta innovates Bio solutions, and DZNE Goettingen.

### 

References[1]ZhangP, et al.
2018;bioRxiv: 348,870.[2]Vanderweyde, et al.
Cell Reports. 2016;15:1455–1466.10.1016/j.celrep.2016.04.045PMC532570227160897

## 85. Kinetics of prion seeding activity in brains of mice infected with mouse-adopted BSE prion

Yoshifumi Iwamaru, Yuichi Matsuura and Kohtaro Miyazawa

Prion Disease unit, Division of Transboundary Animal Disease, National Institute of Animal Health (NIAH), National Agriculture and Food Research Organization (NARO), Tsukuba, Japan

**CONTACT** gan@affrc.go.jp

**ABSTRACT**

Prion diseases are fatal neurodegenerative disorders in humans and animals. The key event in the pathogenesis of these diseases is the conversion of host-encoded normal cellular prion protein (PrPC) into its pathogenic isoform (PrPSc) and its accumulation in the central nervous system. PrPSc is copurified with infectivity and thus thought to be the main constitute of the agent of prion diseases. PrPSc is partially resistant towards the proteinase K (PK) digestion and PK-resistant PrPSc (PrPres) has been used extensively as a surrogate marker for prion infection. However, several studies have revealed that prion infectivity is not always well-correlated with total PrPres levels. Real-time quaking-induced conversion (RT-QuIC) is one of newly developed *in vitro* amplification methods based on prion-seeded amyloid fibril formation by recombinant prion protein. According to a previous study, prion seeding activity appears to be correlated with prion infectivity than with total PrPres-levels. In our previous study of kinetics of mouse-adopted BSE prion propagation and PrPres accumulation, we demonstrated that the brain titer reached a plateau at 100 days post infection whereas PrPres continuously increased towards the end of the incubation period. In this study, we have proceeded to measure kinetics of prion seeding activity in the brain of these mice. We have used end-point dilution RT-QuIC on brain homogenates to produce a detailed measurement of seeding activity throughout the incubation period of the mice and evaluated the temporal relationships among seeding activity, infectivity, and PrPres levels.

## 86. Characterization of cellulose ether liposomes for the inhibition of prion formation in prion-infected cells

Keiko Nishizawa^a^, Kenta Teruya^a^, Ayumi Oguma^a^, Yuji Sakasegawa^a^, Hiroshi Kamitakahara^b^, Hermann Schatzl^c^, Sabine Gilch^d^ and Katsumi Doh-ura^a^

^a^Department of Neurochemistry, Tohoku University Graduate School of Medicine, Sendai, Japan; ^b^Division of Forest & Biomaterials Science Graduate School of Agriculture, Kyoto University, Kyoto, Japan; ^c^Department of Comparative Biology and Experimental Medicine, Faculty of Veterinary Medicine; Hotchkiss Brain Institute; University of Calgary, Canada; ^d^Department of Ecosystem and Public Health, Calgary Prion Research Unit, Faculty of Veterinary Medicine, Hotchkiss Brain Institute, University of Calgary, Calgary, Canada

**CONTACT** Katsumi Doh-ura doh-ura@med.tohoku.ac.jp

**ABSTRACT**

Our previous study demonstrated that a single peripheral administration of cellulose ethers (CE) dose-dependently prolonged the survival of prion-infected rodents when immediately administered after prion infection or even 1 year prior to infection. Additionally, CE inhibited prion formation of persistently prion-infected cells in a manner parallel to the *in vivo* efficacy. However, the mechanism of anti-prion action of CE is not fully understood. Even in the most effective CE, considerable doses, such as few mg/gm body weight in rodents or tens of mg/ml in cell culture media are needed for obtaining substantial effects. Therefore, improvement of the bioavailability is required for the practical use of CE. In this study, we prepared CE-loaded liposomes and examined the anti-prion activity in prion-infected cells to compare the effect of CE-loaded liposomes with that of unformulated CE on anti-prion activity. We also modified CE-loaded liposomes and investigated the uptake levels in prion-infected and macrophage-like cells, and then compared the uptake levels with anti-prion activity levels to help elucidate the anti-prion mechanism of CE-loaded liposomes. The liposomal formulation reduced the EC_50_ dose of CE by <1/200-fold in prion-infected cells. Compared to empty liposomes, CE-loaded liposomes were taken up much more highly by prion-infected cells and less by macrophage-like cells. Phosphatidylserine modification reduced the uptake of CE-loaded liposomes in prion-infected cells and did not change the anti-prion activity, whereas increased the uptake in macrophage-like cells. Polyethylene glycol modification reduced the uptake of CE-loaded liposomes in both types of cells and reduced the anti-prion activity in prion-infected cells. These results suggest that a liposomal formulation of CE is more practical than unformulated CE and showed that the CE-loaded liposome uptake levels in prion-infected cells were not associated with anti-prion activity. Although further improvement of the stealth function against phagocytic cells is needed, the liposomal formulation is useful to improve CE efficacy and elucidate the mechanism of CE action.

## 87. Nascent β structure in the elongated hydrophobic region of a Gerstmann-Sträussler-Scheinker PrP Allele

Ze-Lin Fu^a^^,^^b^, Peter C. Holmes^b^, David Westaway^a^^,^^b^ and Brian D. Sykes^b^

^a^Centre for Prions and Protein Folding Diseases, University of Alberta, Edmonton, AB Canada; ^b^Department of Biochemistry, University of Alberta, Edmonton, AB Canada

**ABSTRACT**

**Objective:** To understand the molecular basis of the misfolding of a novel prion protein (PrP) mutant that causes Gerstmann-Straussler-Scheinker (GSS) disease. This mutant designated ‘HRdup’ was defined in a 34-year-old GSS patient and contains an 8-amino-acid duplication (LGGLGGYV) after Val129. This allele encompasses both part of the glycine-rich portion of the hydrophobic region (HR) and all the first beta strand and is thus distinct from other GSS mutations that comprise missense mutations or insertions of extra octarepeats. Transgenic (Tg) mice expressing HRdup PrP develop a spontaneous neurological syndrome and proteinase K digestion of brain homogenates from these mice shows a pathognomonic GSS band at ~8kDa [1].

**Methods:** For structural analysis, we purified ^13^C, ^15^N double-labelled recombinant mPrP HRdup (118–231) along with two control alleles, mPrP V128 (118–231) and mPrP WT (118–231). Using 3D NMR experiments performed at pH 5.0 and in the absence of added co-factors, we assigned the NMR spectra of both HRdup and V128; therefore, determined the secondary structure of these two proteins and explored the molecular motions and dynamics of HRdup and V128. The secondary structure prediction programmes and MD simulation were also employed to predict and verify the conformation of the N-terminus of HRdup.

**Results:** The NMR experiments showed that HRdup has the same canonical globular PrP^C^ structure as V128, and that the elongated N-terminus of HRdup displayed significant β-propensity while maintaining its high flexibility. MD simulation depicted this nascent β-structure in the hydrophobic region as a β-turn. In addition, we observed that a methionine/valine polymorphism at codon 128 (equivalent to codon 129 in humans) in the presence of oligomerization facilitated by high protein concentrations affected conformational exchange dynamics at residue G130 [2].

**Conclusions:** We conclude that the novel β-structure at the elongated hydrophobic region of HRdup is the root cause of spontaneous misfolding of HRdup PrP. As the exchange dynamics of G130 also suggests the potential involvement of the canonical β-sheet in the initialization of PrP misfolding, we hypothesize that the hydrophobic region of HRdup can adopt a fully extended configuration, fold back and develop into a three-strand antiparallel β-structure that incorporates the canonical β-sheet. This proposed structure is highly consistent with the structure observed in full length hPrP when crystalized with nano-body at its N-terminal end [3].

### 

References[1]MercerRCC, et al. PathogenPLOS
2018;14(1):e1006826.[2]FuZ-F, et al.
Submitted for publication.[3]AbskharonRNN, et al.
JACS
2014;136(2):937–944.

## 88. Sporadic CJD in pregnancy: an interdisciplinary success story

Collin Luk^a^, Allison Thiele^b^, Christina Orru-Groveman^c^, Byron Caughey^c^ and Valerie L. Sim^a,d^

^a^Department of Medicine, Division of Neurology, Faculty of Medicine & Dentistry, University of Alberta; ^b^Department of Obstetrics and Gynaecology, Division of Maternal Fetal Medicine, Faculty of Medicine & Dentistry, University of Alberta; ^c^Rocky Mountain Laboratories, NIAID, National Institutes of Health, Hamilton, MT, USA; ^d^Centre for Prions and Protein Folding Diseases, University of Alberta

**CONTACT** Valerie L. Sim valerie.sim@ualberta.ca

**ABSTRACT**

Sporadic Creutzfeldt-Jakob disease (sCJD) affects 1–2 people per million per year, with a peak incidence in the 7th decade of life. Rarely, CJD can affect women of childbearing age.

There are seven previously reported cases of CJD (six sporadic, one iatrogenic) in pregnancy with five healthy deliveries and no evidence of vertical transmission to date.1–5 In these reports, immunohistochemical screens of placental tissue were negative for PrPSc, but in the one study that tested for placental infectivity, prion disease was transmitted to five of eight cerebrally inoculated Balb mice.2 The possibility that placenta may contain infectious prions has implications for vertical transmission and risk management of delivery. We present a 35 year old G2P1 woman with probable sCJD who presented in her 10th gestational week with a 7-month history of progressive memory impairment and visuospatial difficulties. Initial investigations, including CSF studies, were negative for autoimmune, inflammatory, infectious, and neoplastic conditions. A positive ds-DNA raised the possibility of lupus, for which she was started on Plaquenil, but no other clinical criteria were identified. An electroencephalogram demonstrated bilateral diffuse slowing. Brain MRI was delayed until the beginning of the 2nd trimester out of theoretical concern for foetal exposure. The MRI showed restricted diffusion of basal ganglia and cortical ribboning consistent with Creutzfeldt-Jakob disease. A repeat CSF revealed elevated 14–3-3 (>80 000 AU/mL), elevated Tau protein (>10 513 pg/mL) and positive EP-QuIC assay. There was no PRNP gene mutation. The rate of clinical progression required extensive interdisciplinary management to preserve the pregnancy and safely plan for a number of possible obstetric scenarios. A Kaofeed was required to maintain nutrition. Oxygen was provided by nasal prongs; intubation and ventilation was not required. Fevers and tachycardia developed but without evidence of infection, suggesting central autonomic dysregulation. Ultimately, a healthy baby was delivered via C- section at 36 weeks 3 days gestation, with no obstetric or post-anaesthetic complications. Cord blood, amniotic fluid and placental tissue were collected for evaluation by RT-QuIC. The patient passed away 24 days after delivery. A discussion of diagnostic challenges, management strategies and QuIC results will be presented.

### 

References[1]XiaoX, et al.
Am J Pathol. 2009;174(5):1602–160810.2353/ajpath.2009.081045PMC267124919349373[2]TamaiY, et al.
N Engl J Med. 1992;327(9):649.10.1056/NEJM1992082732709181640969[3]SperlingR, et al.
J Obstet Gynecol Neonatal Nurs. 2005;34(5):546–550.10.1177/088421750528027716227509[4]Di GangiS, et al.
J Matern Fetal Neonatal Med. 2015;28(3):254–261.10.3109/14767058.2014.91667824749800[5]DalmasAF, et al.
Ann Fr Anesth Reanim. 2010;29(11):815–817.10.1016/j.annfar.2010.07.01420934303

## 89. Exploiting their sweet tooth: sugars against prions

M. Kumar^a,b^, Garrett J.B. Molner^c^ and Valerie L. Sim^a,b^,

^a^Department of Medicine, Division of Neurology, Faculty of Medicine & Dentistry, University of Alberta; ^b^Centre for Prions and Protein Folding Diseases, University of Alberta; ^c^Department of Medical Microbiology, Faculty of Medicine & Dentistry, University of Alberta

**CONTACT** M. Kumar manjeet2@ualberta.ca

**ABSTRACT**

Endoplasmic reticulum-associated degradation (ERAD) is critical to cellular protein homoestasis. The accumulation of unfolded or misfolded proteins in the ER challenges this proteostasis and has been implicated in neurodegeneration1. In prion disease, cellular prion protein (PrPC) is converted into a β-sheet rich infectious form (PrPSc)2. PrPC is a membranous protein that is variably N-glycosylated and, like other glycoproteins, depends on enzymes such as glucosidase I, II and glucosyltransferase for maturation of sugar residues as well as proper folding of the protein and its ultimate localization. Failure to properly glycosylate a membrane protein within the ER induces proteostasis stress in the ER and activates ER quality control machinery1,3,4. We hypothesize that trimming the glycans during PrPC synthesis will increase PrP levels in the ER and activate ER-associated degradation (ERAD). In the context of prion infection, this could both reduce available substrate for conversion and enhance clearance of misfolded PrP. We inhibited α- and β-glucosidases with four sugar analogues in CAD5 cells chronically infected with RML strain prions. Moderate dosages of individual analogues resulted in complete eradication of PrPSc within 72 h. Interestingly, treating with lower dosages in combination generated different PK-resistant prion species. Immunoblotting experiments also indicated a change in the protein level of OS-9, a major player in ER quality control and ERAD. OS-9 binds terminally misfolded non-glycoproteins as well as improperly folded glycoproteins and transfers them to ubiquitination machinery for targeted degradation5-7. We speculate that truncation of N-linked glycans on PrP with the combination of lower dose sugar analogues resulted in the formation of modified improperly folded mono- and di-glycosylated PrPC with altered conformational repertoire. These types of sugar analogues have high oral bioavailability, can easily penetrate the central nervous system, have extensively characterized biological and pharmacokinetic properties. As such, they may have therapeutic utility for prion diseases at moderate dose. In addition, they represent a novel tool for the experimental manipulation of prion strains.

### 

References[1]Rendleman, et al.
eLife. 2018;7:e39054[2]PrusinerSB.
Proc Natl Acad Sci. 1998;95:13,363–13,383[3]Fagioli, et al. J Biol Chem
2001;276:12,885–12,892;4.[4]Smith, et al. Science
2011;334:1086–1090.[5]Bernasconi, et al. J Biol Chem
2008;283:16,446–16,454;6.[6]Hosokawa, et al. J Biol Chem
2009;284:17,061–17068.[7]Alcock, et al. J Mol Biol
2009;385:1032–1042.10.1016/j.jmb.2008.11.04519084021

## 90. Estimation of the binding sites of inhibitory factors on recombinant PrP molecule by RT-QuIC

Akio Suzuki, Kazuhei Sawada, Takeshi Yamasaki and Motohiro Horiuchi

Laboratory of Veterinary Hygiene, Faculty of Veterinary Medicine, Hokkaido University, Sapporo, Japan

**CONTACT** Akio Suzukisusan@vetmed.hokudai.ac.jp

**ABSTRACT**

**Background**: Real-time quaking-induced conversion (RT-QuIC) is one of several highly sensitive methods for the detection of prions. However, the reaction is easily interfered by inhibitory factors in tissue homogenates. We previously showed that recombinant cervid PrP (rCerPrP) can react with prions even in the presence of tissue homogenates (Prion 2017, P152). To clarify the region of the PrP molecule that is affected by inhibitory factors, we produced a variety of rPrP mutants and analysed the reactivity of the PrP mutants to H- and L-BSE prions by RT-QuIC.

**Material and Methods**: Full-length recombinant sheep (ARQ) PrP (rShPrP), mouse PrP (rMoPrP), rCerPrP, and their C-terminal chimeras (rCerPrP-Mo^Cterm^ and rMoPrP-Cer^Cterm^), and their mutants that have point-mutations at ShPrP-specific amino acid (aa) 98 S and/or CerPrP-specific 173 N and 177 T (rMoPrP-S169N_Cer_/N173T_Cer_, rMoPrP-T94S_Sh_/N96S_Sh_/S169N_Cer_/N173T_Cer_ and rCerPrP-N173S_Mo_/T177N_Mo_/, rCerPrP-T98S_Sh_/N173S_Mo_/T177N_Mo_) were used as substrates. Brain homogenates (BH) of cattle infected with H-BSE and L-BSE were serially diluted with PBS or normal cattle BH and used as seeds. RT-QuIC reactions were performed under conditions containing 10 mM Na-phosphate (pH 7.4), 500 mM NaCl and 0.001% SDS at 37 °C.

**Results**: rShPrP, rMoPrP and rCerPrP could comparably detect H-BSE and L-BSE diluted at 10^−4^–10^−7^ with PBS. However, the reaction of rShPrP with L-BSE was delayed for 13.4 h on average and those of rMoPrP with H-BSE and L-BSE were delayed for 27.4 h and completely inhibited by 0.1% normal BH, respectively. Compared to authentic rPrPs, the reaction of rCerPrP-Mo^Cterm^ with L-BSE in the presence of normal BH was delayed for 10.7 h, whereas that of rMoPrP-Cer^Cterm^ was shortened by 21.9 h. These results suggest that the C-terminus of rMoPrP is more affected by the inhibitory factors compared with those of rCerPrP. On the other hand, the reaction of rCerPrP-N173S_Mo_/T177N_Mo_/and rCerPrP-T98S_Sh_/N173S_Mo_/T177N_Mo_ with L-BSE was delayed for 10.8 h and 13.8 h compared to rCerPrP, whereas those of rMoPrP-S169N_Cer_/N173T_Cer_ and rMoPrP-T94S_Sh_/N96S_Sh_/S169N_Cer_/N173T_Cer_ was shortened by 11.5 h and 14.0 h compared to rMoPrP. Taken together, the inhibitory factors may also interfere with the region including CerPrP specific N173, and T177.

**Conclusion**: Our results suggest that conversion efficiency in the RT-QuIC reaction may be interfered with by the inhibitory factors at least at two regions of the PrP molecule, one is the C-terminus and the other is the region including CerPrP-specific aa 173 N and 177 T. These regions have been showen to be adjacent in the three-dimensional structure of PrP^C^; therefore, inhibitory factors may bind to the site including these regions.

## 91. PrP regulates neural stem cell senescence through epidermal growth factor receptor

Bradley R. Groveman, Simote T. Foliaki, Aleksandar R. Wood, Brent Race and Cathryn L. Haigh

Rocky Mountain Laboratories, NIAID, NIH, Hamilton, MT, USA

**CONTACT** Bradley R. Groveman Bradley.groveman@nih.gov

**ABSTRACT**

The prion protein (PrP) is expressed in a wide variety of progenitor cells and is known to be involved in neural stem cell self-renewal processes. The mechanisms by which PrP acts to modulate cell homeostasis have, however, not been fully elucidated. Recent studies have shown that PrP has a direct role in preventing premature senescence [1], and that knockdown of PrP increases senescence markers and decreases the cell proliferative potential. PrP has also been shown to regulate epidermal growth factor receptor (EGFR) through Notch signalling. Notch signalling, which can upregulate EGFR, is disrupted in the absence of PrP [2], leading to a reduction in EGFR expression. Furthermore, inhibition of EGFR or depletion of EGF have been shown to suppress senescence in proliferative cells [3]. Herein we attempt to show the role of PrP in regulating cellular senescence through the EGFR pathway.

**KEYWORDS:** Prion; PrP; neural stem cell; senescence; epidermal growth factor receptor; EGFR

### 

References[1]BoilanE, et al.
Mech Ageing Dev. 2018;170:106–113.10.1016/j.mad.2017.08.00228800967[2]Martin-LanneréeS, et al. CellsStem
2017;35(3):754–765.10.1002/stem.250127641601[3]AlexanderPB, et al. ResCell
2015;25(1):135–138.10.1038/cr.2014.141PMC465058325367123

## 92. Region-specific interference of PrP^Sc^ accumulation in spleen and brain by poly-L-arginine (PLR)

Jieun Kim^a^, Taeyeon Kim^b^, A-ran Kim^a^, Hakmin Lee^a^, Trang H. T. Trinh^a^, Junwu Shin^a^, Sungeun Lee^a^ and Chongsuk Ryou^a^

^a^Department of Pharmacy, College of Pharmacy and Institute of Pharmaceutical Science and Technology, Hanyang University, Ansan, Gyeonggi-do, Republic of Korea; ^b^Division of Developmental Biology and Physiology, School of Biosciences and Chemistry, Institute for Basic science, Sungshin University, Seoul, Republic of Korea.

**CONTACT** Chongsuk Ryou cryou2@hanyang.ac.kr

**ABSTRACT**

**Background**: In our previous study, PLR was shown to inhibit prion propagation in the cell culture model of prion diseases (1). In current study, we investigated the effect of PLR *in vivo* whether it inhibits prions.

**Material and Methods**: Firstly, mice inoculated intraperitoneally (IP) with prions were administered with PLR twice a week for 4 weeks 10–40 mg/kg by the IP route. Spleen were collected from infected mice at every 21-day until 84 days post-inoculation. In another approach, to observe the delay of disease onset, mice infected intracerebrally with prions, were administered with PLR twice a week for 4 weeks 10–25 mg/kg PLR by the IP route. As disease onsets, the brain and spleen were collected. The level of PrP^Sc^ in the brain and spleen were detected by Western blotting. The accumulation of the PrP^Sc^ and glial fibrillary acidic protein were monitored using immunohistochemistry. Vacuolation in the brain was investigated using H&E stained brain sections.

**Result**: PrP^Sc^ propagation was suppressed in the spleen of PLR-treated group during the asymptomatic stage. Administration with 10–25 mg/kg PLR prolonged the incubation time of prion diseases over seven days. At the disease onset, the level of PrP^Sc^ reduced in the spleen, but unchanged in the brain. However, immunohistochemistry analysis showed that the level of PrP^Sc^ in white pulp of the spleen and specific areas of the brain, such as hypothalamus, cortex, and hippocampus, but not mid-brain, thalamus, and straitum, was reduced by PLR. Moreover, glial fibrillary acidic protein staining indicated that PLR decreased astrogliosis in thalamus, cortex, and polymorph layer 3 of cerebral cortex of brain but not in other brain regions. In addition, H & E staining indicated that the decrease of vacuolation area and vacuole counts in mid-brain, hypothalamus, hippocampus, and straitum regions of the brains from the PLR-administered mice.

**Conclusion**: Considering suppression of prion propagation in spleen during asymptomatic stage, significant increase of survival time and brain region-specific inhibition of neuropathological onsets, we demonstrated PLR as a potential therapeutic candidate for prion diseases and the value of further studies.

**KEYWORDS:** Prion; poly-L-arginine; PrPSc; spleen; brain

### 

Reference[1]Waqas, et al. Mol Cell Biochem
2017;428(1–2):57–66.10.1007/s11010-016-2916-6PMC589879828063003

## 93. Prion protein distribution among white-tailed deer and mule deer exocrine glands

Anthony M. Ness^a^^,^^b^, Kelsey Saboraki^c^, Camilo D. Velásquez^a^^,^^b^, Danielle Gushue^a^^,^^b^, Alicia O. Garcia^a^^,^^b^, Joshua Craig^a^^,^^b^, Taraneh Ashrafi^a^^,^^d^, Judd Aiken^a^^,^^d^, Susan Lingle^c^*, Debbie McKenzie^1,2*^

^a^Centre for Prions and Protein Folding Diseases, University of Alberta; ^b^Department of Biological Sciences, University of Alberta; ^c^Department of Biology, University of Winnipeg; ^d^Department of Agricultural, Food, and Nutritional Sciences, University of Alberta

**CONTACT** Anthony M. Ness amness@ualberta.ca

**ABSTRACT**

**Background**: Chronic wasting disease (CWD) is a contagious neurodegenerative prion disease afflicting cervids in Canada, the United States, South Korea, and Scandinavia. The precise mechanism of natural CWD transmission is poorly understood but is believed to involve both direct contact between cervids and exposure to environmental reservoirs of PrP^CWD^. The scent glands in the face and legs of deer are numerous and serve for direct and indirect inter-individual communication and social behaviours. Glandular innervation by the autonomic nervous system may act as a site for CWD prion uptake, neuroinvasion, and retrograde transport towards the central nervous system.

**Materials and Methods**: Facial and leg exocrine glands were collected from adult and white-tailed deer and mule deer males and females harvested from Canadian Forces Base Wainright, Alberta, Canada. Vomeronasal, forehead, pre-orbital, parotid, and nasal glands in the head, and the tarsal, metatarsal, and interdigital glands in the legs were homogenized for biochemical analysis or embedded for histological analysis. White-tailed deer and mule deer glandular PrP^C^ levels were determined by Western blot and ELISA. The distribution of PrP^D^ was evaluated by immunohistochemistry (IHC).

**Results and Discussion**: We show the relative PrP^C^ levels and distribution in the various facial and leg exocrine glands studied. IHC revealed PrP^D^ within select glands. The involvement of cervid exocrine glands in CWD transmission has been poorly studied; however, our observations suggest these glands act as sites for prion entry and shedding – deserving further investigation. Cervid exocrine glands may represent a biologically and ecologically important transmission route of CWD in deer. Further, concurrent research suggests that species-and sex-specific social behaviours, involving the glandular tissues, may provide insights as to why CWD is most prevalent in mule deer males and least prevalent in white-tailed females.

**KEYWORDS:** Chronic wasting disease; prion; deer; cervids; glands; transmission

## 94. Plasma tau and neurofilament light chain as biomarkers for the human prion diseases: evaluation in a large prospective natural history cohort

Andrew G. B. Thompson^a^, Prodromos Anastasiadis^a^, Akin Nihat^a^^,^^b^, Tze Mok How^a^^,^^b^, Peter Rudge^a^^,^^b^, Amanda Heslegrave^c^, Henrik Zetterberg^c^, John Collinge^a^^,^^b^, Graham S. Jackson^a^ and Simon H. Mead^a^^,^^b^

^a^MRC Prion Unit, University College London, UK; ^b^National Prion Clinic, University College London Hospitals NHS Foundation Trust, UK; ^c^Dementia Research Institute Biomarkers Laboratory, University College London, UK.

**CONTACT** Andrew GB Thompson

**ABSTRACT**

**Background**: Diagnostic methods for prion disease have advanced substantially, but unmet needs remain for biomarkers to fill a number of other important roles. These include monitoring of disease activity, predicting symptom onset in individuals at high risk (such as carriers of prion protein gene (*PRNP*) mutations), and enabling earlier diagnosis. Tau and neurofilament light chain (NfL) protein concentrations in blood are known to be elevated in prion disease, and have shown promise as quantitative markers of central nervous system disease activity in a range of other conditions.

**Materials and Methods**: We used ultrasensitive immuno-assays (*Simoa*) to measure tau and NfL concentrations in 709 plasma samples taken from 377 individuals with prion disease during their participation in a large prospective clinical study (the UK National Prion Monitoring Cohort), in addition to healthy and neurological control groups. This provides an unprecedented opportunity to analyse their clinical associations and potential as biomarkers.

**Results**: Plasma tau and NfL concentrations are increased across all prion disease types. For distinguishing sporadic CJD (sCJD) from a clinically relevant ‘prion mimic’ control group, both show moderate diagnostic value (area under ROC curves: tau 0.81, NfL 0.72). In sCJD, plasma NfL was substantially elevated in every sample tested, including during early disease with minimal functional impairment. Plasma tau was independently associated with *rate* of clinical progression in sCJD, while plasma NfL showed independent association with *severity* of functional impairment. In asymptomatic *PRNP* mutation carriers, plasma NfL was higher in samples taken within 2 years of symptom onset than in those taken earlier. We present biomarker trajectories for 9 *PRNP* mutation carriers who developed symptoms during follow-up, and show potential for plasma NfL as a ‘proximity marker’.

**Conclusions**: We conclude that plasma tau and NfL represent the best currently available candidates for a number of vital biomarker roles in prion disease. We discuss directions for future work, particularly focussing on ways that these biomarkers could be valuable for therapeutic research.

## 95. Gene-edited murine cell lines for propagation of chronic wasting disease prions

Rupali Walia^a^^,^^b^, Rohit Chandra^a^^,^^c^ and Hermann M. Schatzl^a^^,^^b^

^a^Department of Comparative Biology & Experimental Medicine, University of Calgary, Calgary, Canada; ^b^Calgary Prion Research Unit, University of Calgary, Calgary, Canada; ^c^Faculty of Science, University of British Columbia, Vancouver

**CONTACT** Rohit Chandra rohit.chandra@ucalgary.ca

**ABSTRACT**

Chronic wasting disease (CWD), a highly contagious prion disease, has recently emerged as a threat to both farmed and free ranging cervids across North America, South Korea, and Europe. There is thus a pressing need to design versatile cell culture models which can complement the animal studies on CWD and help to contain this disease. Defining such cell culture models is mostly a random process, and includes extensive subcloning and selection. To extend the range of cell models propagating CWD prions, we gene-edited mouse cell lines known to well propagate murine prions. Endogenous prion protein (PrP) was ablated in CAD5 cells and immortalized mouse embryonic fibroblasts (MEF), using CRISPR-Cas9 gene editing. PrP knock-out cells were reconstituted with mouse, bank vole (BV) and cervid PrP genes by lentiviral transduction. Reconstituted cells expressing mouse PrP provided proof-of-concept for re-established prion infection. The promiscuous nature of BV PrP^c^ was exploited to demonstrate the permissiveness of these reconstituted cell lines to infection by CWD prions, even without subcloning. Cells reconstituted with BV or cervid PrP and infected with two types of CWD prions, white-tailed deer and mule deer prions, tested positive in prion conversion assay, whereas non-reconstituted cells were negative. We here provide a successful strategy for designing cell lines with specific as well as broader susceptibility to prion infection, while retaining a common host background. Data obtained by infecting such gene-targeted and re-constituted cultured cells will provide important new insights into species and prion strain barriers and provide new experimental systems for studying CWD prions *in vitro*.

## 96. PrPSc aggregation state dictates biochemical properties of prions and disease pathogenesis

Sheng Chun Chang^a^, Samia Hannaoui^a^, Stefanie Czub^b^ and Sabine Gilch^a^

^a^Department of Ecosystem and Public Health, Faculty of Veterinary Medicine, Calgary Prion Research Unit, Hotchkiss Brain Institute, University of Calgary, Calgary, Canada; ^b^Canadian Food Inspection Agency Lethbridge Laboratories, Lethbridge, Canada

**CONTACT** Sheng Chun Chang shengchun.chang@ucalgary.ca

**ABSTRACT**

**Background**: Transmissible spongiform encephalopathies (TSE) are devastating neurodegenerative diseases caused by the conversion of the host-encoded cellular prion protein (PrP^C^) into the pathological misfolded isoform known as PrP^Sc^. Various strains of prions exist, each of which consists of a unique conformational, biochemical, and biological profile. Previous studies have indicated that the profile of PrP^Sc^ aggregation states also is strain specific. Our study investigates whether biochemical and biological properties of individual fractions of PrP^Sc^ are stable upon serial passage *in vivo*.

**Methods**: A pool of CWD2 elk brain homogenate was subject to a sedimentation velocity gradient and separated into 30 fractions. Alternating fractions were intracerebrally inoculated into transgenic mice overexpressing elk PrP^C^ (tgElk) and incubation times and clinical signs were monitored. Brains (first passage) were harvested upon the progression of terminal prion disease. Brains of three mice each inoculated with PrP^Sc^ fractions 2–6 (low molecular weight (MW)), 8–18 (medium MW), and 20–30 (high MW), respectively, were used for second passage into tgElk mice. Brain samples were subjected to digestion with increasing concentrations of Proteinase K (PK) to determine the PK concentration required to degrade 50% of PrP^Sc^ (cPK_50_).

**Results**: In the first passage, mice inoculated with low MW PrP^Sc^ aggregates exhibited symptoms of hyper-excitability, while those inoculated with high MW aggregates were lethargic and gained weight, and a prolonged incubation was observed in both groups, while those given medium MW aggregates had a decreased incubation time. Biochemical analysis showed that mice inoculated with low or medium MW aggregates had PrP^Sc^ that were markedly less resistant to PK digestion compared to the PrP^Sc^ from mice inoculated with high MW aggregates. These characteristics were no longer observed in the second passage, where the incubation period and the PK resistance among the groups were similar, though the observed symptoms in the mice inoculated with the low or high MW PrP^Sc^ were still hyper-excitability and lethargy, respectively, as in the first passage.

**Conclusion**: Our study demonstrates that inoculation of different PrP^Sc^ aggregation states separated by molecular weight can lead to differing pathogenesis and transient changes to the biochemical characteristics. This will add novel knowledge to our understanding of transmissible biochemical characteristics of prions.

## 97. *In vitro* seeding activity of glycoform-deficient prions from variably protease-sensitive prionopathy and familial CJD associated with PrP^V180I^ mutation

Zerui Wang^a^^,^^b^, Jue Yuan^a^, Pingping Shen^a^^,^^b^, Romany Abskharon^c^, Yue Lang^a^^,^^b^, Johnny Dang^a^, Alise Adornato^a^, Ling Xu^a^, Jiafeng Chen^b^, Jiachun Feng^b^, Mohammed Moudjou^d^, Tetsuyuki Kitamoto^e^, Jan Langeveld^f^, Brian Appleby^a^^,^^g^^,^^h^, Jiyan Ma^c^, Qingzhong Kong^a^^,^^g^^,^^h^, Robert B. Petersen^a^^,^^i^, Li Cui^b^ and Wen-Quan Zou^a^^,^^b^^,^^g^^,^^h^

^a^Department of Pathology, Case Western Reserve University School of Medicine, Cleveland, Ohio, USA; ^b^Department of Neurology, The First Hospital of Jilin University, Changchun, Jilin Province, the People’s Republic of China; ^c^Center for Neurodegenerative Science, Van Andel Research Institute, Grand Rapids, MI, USA; ^d^NRA, Université Paris-Saclay, UR892, Virologie Immunologie Moléculaires, Jouy-en-Josas, France; ^e^Center for Prion Diseases, Tohoku University Graduate School of Medicine, Sendai, Japan; ^f^Wageningen BioVeterinary Research, Lelystad, the Netherlands; ^g^National Prion Disease Pathology Surveillance Center, Case Western Reserve University School of Medicine, Cleveland, OH, USA; ^8^Department of Neurology, Case Western Reserve University School of Medicine, Cleveland, OH, USA; ^i^Foundation Sciences, Central Michigan University College of Medicine, Mount Pleasant, MI, USA

**CONTACT** Wen-Quan Zou WQZ wxz6@case.edu

**ABSTRACT**

Both sporadic variably protease-sensitive prionopathy (VPSPr) and familial Creutzfeldt-Jakob disease linked to the prion protein (PrP) V180I mutation (fCJD^V180I^) have been found to share a unique pathological prion protein (PrP^Sc^) pattern that lacks the protease-resistant PrP^Sc^ glycosylated at residue 181, apparently because two of four cellular PrP (PrP^C^) glycoforms are not converted into PrP^Sc^. To investigate the seeding activity of these unique PrP^Sc^ molecules, we conducted *in vitro* prion conversion experiments using serial protein misfolding cyclic amplification (sPMCA) and real-time quaking-induced conversion (RT-QuIC) assays with different PrP^C^ substrates. Unexpectedly, we observed that the seeding of PrP^Sc^ from VPSPr or fCJD^V180I^ in the sPMCA reaction with brain homogenates from normal human or humanized transgenic (Tg) mice generated PrP^Sc^ molecules that are dominated by the diglycosylated isoform, along with PrP^Sc^ monoglycosylated at residue 181. The efficiency of PrP^Sc^ amplification was significantly higher in MM than in VV human brain homogenate, whereas it was higher in TgVV than in TgMM mouse brain homogenate. PrP^C^ from the brain homogenate mixture of TgMM and Tg mice expressing PrP^V180I^ mutation (Tg180), but not that from TgV180I alone, was converted into PrP^Sc^ by seeding with the VPSPr or fCJD^V180I^. The RT-QuIC seeding activity of PrP^Sc^ from VPSPr and fCJD^V180I^ was significantly lower than that of sCJD. Our results suggest that the formation of glycoform-selective prions may be associated with an unidentified factor in the affected brain and the glycoform-deficiency of PrP^Sc^ does not affect the glycoforms of *in vitro* newly-amplified PrP^Sc^

**Funding**

Supported in part by the CJD Foundation and the National Institutes of Health (NIH) NS062787 and NS087588 to W.Q.Z., NS062787 and NS109532 to W.Q.Z., and Q.K., NS088604 to Q.K., the Centers for Disease Control and Prevention Contract UR8/CCU515004 to B.S.A., the National Natural Science Foundation of China (NNSFC) [No. 81,801,207] to PS, as well as NNSFC [No. 81,671,186] to LC.

## 98. Longitudinal comparison of real-time conversion and immunohistochemistry for detection of chronic wasting disease in orally exposed white-tailed deer

Nathaniel D. Denkers^a^, Davin M. Henderson^a^, Clare E. Hoover^a^^,^^b^*, Amy V. Nalls^a^, Erin McNulty^a^, Candace K. Mathiason^a^ and Edward A. Hoover^a^

^a^Prion Research Center, Department of Microbiology, Immunology, and Pathology, College of Veterinary Medicine and Biomedical Sciences, Colorado State University, Fort Collins, CO, USA

**CONTACT** Nathaniel D. Denkers nathaniel.denkers@colostate.edu

*Present address: AstraZeneca, Waltham, NJ

**ABSTRACT**

**Background**: Chronic wasting disease (CWD) continues to expand across North America and Canada, and more recently was discovered in Scandinavia. While the exact mechanism of CWD transmission has yet to be elucidated, early diagnosis of infected animals remains a priority in curtailing its spread. While immunohistochemistry (IHC) and enzyme linked immunosorbent assay (ELISA) remain the gold standards for diagnosing CWD, real-time quaking induced conversion (RT-QuIC) is capable of detecting substantially lower concentrations of prions, which may translate into detection earlier in disease course. In this study, we sought to compare the sensitivity of RT-QuIC and IHC on a series of longitudinal tonsil and recto-anal mucosal lymphoid tissue (RAMALT) biopsies from white-tailed deer during the course of CWD progression.

**Methods**: White-tailed deer were inoculated via the per os (PO) route with CWD(+) [*n* = 20] and CWD(-) [*n* = 4] inocula (brain homogenate or saliva) and paired tonsil and RAMALT biopsies were collected every 3 months thereafter. Biopsies were assayed for prion seeding activity by RT-QuIC and for PrP^CWD^ deposition by IHC.

**Results**: RT-QuIC detected seeding activity in 10 of 20 (50%) tonsil biopsies prior to IHC detection, on average of 5.1 months (3–15 months) earlier. In RAMALT biopsies, detectable seeding activity was observed in 13 of 20 (65%) samples prior to IHC detection, on average 4.6 months (3–15 months) earlier. In the remaining biopsies, detection was concurrent by both methods. At no sampling point did a positive IHC result precede a positive RT-QuIC result. Of note, for biopsy samples that had already been determined positive by both RT-QuIC and IHC in a previous collection, RT-QuIC detected seeding activity in seven (7) tonsil and four (4) RAMALT follow-up biopsies even though no lymphoid follicles were present in the paired samples to assess positivity by IHC. All biopsies from CWD(-) deer were negative by both assays throughout the study. Overall, these results demonstrate that RT-QuIC detected CWD positivity in both tonsil and RAMALT biopsies approximately 5 months prior to IHC. Moreover, seeding activity was detectable even when (due to poor biopsy sampling) lymphoid follicles were not present to permit meaningful IHC analysis.

**Conclusions**: These data indicate that RT-QuIC is more sensitive than IHC in detecting CWD infection, and could thereby be useful in determining future management strategies.

**Funding**

Supported by NIH R01-NS-061902, P01-AI-077774, F30-ODO-118,143, T32-OD0-10,437

## 99. Cellulose ethers interfere with CWD prion propagation *in vitro* and *in vivo*

Preetha Gopalakrishnan^a^, Jie Yu^a^, Samia Hannaoui^a^, Sheng Chun Chang^a^, Maria Arifin^a^, Katsumi Doh-ura^b^, Hermann Schatzl^c^ and Sabine Gilch^a^

^a^Department of Ecosystem and Public Health, Calgary Prion Research Unit, Faculty of Veterinary Medicine; Hotchkiss Brain Institute; University of Calgary, Calgary, Canada; ^b^Department of Neurochemistry, Tohoku University Graduate School of Medicine, Sendai, Japan; ^c^Department of Comparative Biology and Experimental Medicine, Faculty of Veterinary Medicine; Hotchkiss Brain Institute; University of Calgary, Canada

**ABSTRACT**

Chronic wasting disease (CWD) is a prion disease of free ranging and farmed cervids. CWD is highly contagious and the absence of an effective vaccine makes a complete eradication of CWD not realistic to date. Recently, a single subcutaneous injection of Cellulose Ethers (CEs) was reported to markedly extend the life span of transgenic mice infected with 263K prions. Despite the fact that the mechanisms of action of the CEs are still a matter of discussion, this approach could be a good strategy against CWD spreading, thus, limiting its potential in transmission to cervid and non-cervid animals and potentially to humans. In this study, we used the cellulose ethers TC-5RW and 60SH *in vitro* and *in vivo* to assess their efficacy to interfere with CWD prion propagation. *In vitro*, TC-5RW displays a dose dependant response to inhibit RT-QuIC reaction seeded with different CWD isolates from white-tailed deer (WTD), mule deer (MD) and elk. The inhibitory effect starts with a concentration as low as 0.1 μg/ml and totally inhibits conversion at 10 μg/ml. Groups of transgenic mice overexpressing elk PrP^C^ were subcutaneously (s.c.) injected with the CE compounds and inoculated intracerebrally (i.c.) with different CWD isolates (elk, WTD, and MD) the same day (group A) or one month after the CE injection (group B). All treated groups show a significantly prolonged incubation period. Survival is extended between +12% to +32% in mice of group A, and +20% to +31% in mice of group B, depending on the CWD isolate used for inoculation, when compared to the control group. The prolonged incubation period in the treated groups correlates to a significantly reduced resistance of prions to proteinase K (PK). This is demonstrated by Western blot analyses of 10% brain homogenate digested with a range of PK concentration. Interestingly, upon passage of brain homogenates form control and group B mice, tgElk mice inoculated with group B material showed a prolonged life span of +17% without further CE treatment, supporting the hypothesis of a modified state of infectivity in the mice treated during first passage. In summary, our results demonstrate that CEs are efficient in inhibiting CWD prion propagation independent of the origin of the isolates used for inoculation. We show that CE treatment alters biochemical properties and reduces infectivity of prions *in vivo*. Therefore, these compounds can be useful in limiting the spread of CWD.

## 100. RT-QuIC detection of pathological prion protein in subclinical goats following experimental oral transmission of L-type BSE

Alessandra Favole^a^, Maria Mazza^a^, Elena Vallino Costassa^a^, Antonio D’Angelo^b^, Guerino Lombardi^c^, Claudia Palmitessa^a^, Dell’Atti Luana^a^, Paola Crociara^a^, Elena Berrone^a^, Marina Gallo^a^, Monica Lo Faro^a^, Tiziana Avanzato^a^, Pier Luigi Acutis^a^, Cristina Casalone^a^ and Cristiano Corona^a^

^a^Istituto Zooprofilattico Sperimentale del Piemonte, Liguria e Valle d’Aosta; ^b^University of Turin, Department of Veterinary Science; ^c^Istituto Zooprofilattico Sperimentale della Lombardia e dell’Emilia Romagna

**CONTACT** Alessandra Favole alessandra.favole@izsto.it

Abstract not available.

### 

References[1]SimmonsMM, et al. ResVet
2016;47(1):112.[2]Vallino CostassaE, et al. OnePLoS
2018;13(5):e0198037.10.1371/journal.pone.0198037PMC596840529795663

## 101. Application of high-throughput, capillary-based Western immunoassays to modulated cleavage of PrP^C^

Andrew R. Castle^a^^,^^b^, Nathalie Daude^a^, Sabine Gilch^c^ and David Westaway^a^^,^^b^^,^^d^

^a^Centre for Prions and Protein Folding Diseases, University of Alberta, Edmonton, Canada; ^b^Department of Medicine, University of Alberta, Edmonton, Canada; ^c^Department of Ecosystem and Public Health, Calgary Prion Research Unit, Faculty of Veterinary Medicine and Hotchkiss Brain Institute, University of Calgary, Calgary, Canada; ^d^Department of Biochemistry, University of Alberta, Edmonton, Canada

**CONTACT** Andrew R. Castle acastle@ualberta.ca

**ABSTRACT**

The C-terminal fragment of the cellular prion protein (PrP^C^) that is generated physiologically by the alpha-cleavage pathway is resistant to misfolding and acts as a dominant-negative inhibitor of prion replication. This fragment, known as C1, also lacks the reported binding sites for neurotoxic beta- amyloid and alpha-synuclein oligomers that are present in full length (FL) PrP^C^. In contrast, a rarer proteolytic pathway – beta-cleavage – produces the longer C-terminal fragment C2 that retains the ability to misfold and may be resistant to cleavage at the ‘alpha’ site. Thus, modulators of PrP^C^ proteolysis that enhance alpha-cleavage or inhibit beta-cleavage are of interest therapeutically. To identify such modulators, we initiated cell-based screens of small molecule libraries to identify compounds with effects on PrP^C^ proteolysis. We decided to measure the levels of FL PrP^C^ and its cleavage fragments using an automated, capillary-based Western immunoassay system; this technique offers higher throughput and more accurate quantification than traditional Western blotting. Firstly, we optimised the detection and quantification of FL PrP^C^ and its cleavage fragments in cell lysate samples treated with peptide-N-glycosidase F to remove the N-linked glycans normally present. Secondly, we optimised the simultaneous quantification of PrP^C^ and beta-tubulin levels to allow for loading error correction. Thirdly, we developed a protocol for performing all cell culture, lysis and deglycoslyation steps in 96-well microplates before capillary Western analysis and demonstrated that this approach resulted in comparable intra-assay percentage coefficients of variation to using standard protocols for these steps. Finally, we screened the ApexBio DiscoveryProbe Protease Inhibitor Library by exposing RK13 cells expressing the artificial ‘S3’ variant of PrP^C^ to each compound at 20 µM concentration for 4 days; S3 PrP^C^ displays accentuated beta-cleavage in RK13 cells, thereby enabling C2 levels to be quantified reliably. Analysis of the screening data revealed distinct classes of inhibitors with differing effects on PrP^C^ fragmentation products.

**KEYWORDS:** Prion disease; neurodegeneration; protein misfolding; proteolysis; fragmentation; high- throughput screening; capillary Western

## 102. Distinctive PrP particles comprise hamster-adapted prion strains

Leonardo M. Cortez^a^^,^^b^, Camilo Duque Velázquez^a^^,^^b^, Debbie McKenzie^a^^,^^c^, Valerie L. Sim^a^^,^^b^

^a^Centre for Prions and Protein Folding Diseases, University of Alberta; ^b^Department of Medicine, Faculty of Medicine & Dentistry, University of Alberta; ^c^Department of Biological Sciences, Faculty of Science, University of Alberta

**CONTACT** Leonardo M. Cortez lcortez@ualberta.ca

**ABSTRACT**

**Background:** The conversion of PrP^C^ into a pathogenic conformation results in multimeric PrP^Sc^ particles that range in size from small detergent-soluble oligomers to large insoluble aggregates. The prion strain phenomenon, characterized by different phenotypes/pathologies of disease, may be explained by PrP^Sc^ conformational differences resulting in reproducible strain-specific PrP^Sc^ multimeric states. However, the complexity, variability, and properties of these PrP^Sc^ particle distributions are not well established. It has been hypothesized that prion strains are comprised of a cloud of PrP^Sc^ conformations with different aggregation properties. We present a detailed study of fractionated PrP^Sc^ particles to better understand prion strains, transmission barriers, drug resistance, and therapeutic targets.

**Methods:** Asymmetric-flow field-flow fractionation (AF4), a one-phase chromatography technique of size-based separation, was used to isolate soluble prion particles from brain homogenate of 263K, Hyper (HY) and Drowsy (DY) infected hamsters. We determined the hydrodynamic radius (Rh) and amount of total and PK-resistant PrP in each fraction using dynamic light scattering and immunoblotting. Using different AF4 buffer conditions, we studied the stability of different PrP oligomers. Particle size was correlated with RT-QuIC seeding activity.

**Results:** Each strain generated a reproducible distribution of PrP particle sizes. For HY and 263K, there was a population of smaller PrP particles (Rh = 2-8nm) that were sensitive to PK digestion but had strong seeding activity. A second population of larger PrP particles (Rh~15-200nm) was detergent and PK resistant but had less seeding activity than the smaller particles. In contrast, DY had predominantly a smaller population of PrP particles, peaking at Rh~7nm. These smaller DY particles were sensitive to PK but had less seeding activity than 263K and HY. The few large DY PrP particles were resistant to PK but sensitive to detergent, and their seeding activity was stronger than large HY and 263K PrP particles.

Interestingly, the size distribution of PK-resistant PrP particles was almost identical for all strains, with PK resistance being evident in particles with Rh>20nm. This suggests that PK resistance is related to the size of PrP particle.

**Conclusions:** The distinct PrP^Sc^ particle size distribution and oligomer properties in different strains suggest there are mechanisms to maintain a stable PrP^Sc^ population in each strain. Hyper and 263K, which have similar disease outcomes, are comprised of similar distributions of PrP^Sc^ particles; Drowsy, with very a different disease outcome, has different amounts of aggregates with different biochemical properties. PK sensitive PrP appears to play a central role in these differences.

## 103. Biochemical comparison of peripheral vs. central nervous system-derived PrP^CWD^ in white-tailed deer

Lindsay E. Parrie, Davin M. Henderson, Clare Hoover, Kristen A. Davenport, Sarah K. Cooper and Edward A. Hoover

Prion Research Center, Colorado State University, Fort Collins, CO, USA

**CONTACT** Lindsay E. Parrie lindsay.parrie@colostate.edu

**ABSTRACT**

In nature, cervids are infected with chronic wasting disease (CWD) by oral and nasal mucosal exposure. However, PrP^C^ expression is not the primary driver of PrP^CWD^ accumulation, as tissues with the highest PrP^C^ expression are not the first to accumulate PrP^CWD^. Studies of early CWD pathogenesis have implicated pharyngeal lymphoid tissues as the earliest sites of prion accumulation. PrP^CWD^ replication differs between peripheral tissues and the CNS. PrP^CWD^ deposits in lymphoid tissues are smaller and more diffuse than those found in brain, which is typically characterized by large amyloid plaques. Additionally, lymphoid-derived PrP^CWD^ appears to be more sensitive to proteinase K digestion than is brain PrP^CWD^, despite equivalent seeding activity as measured in RT-QuIC. Our studies address two main questions: (1) Do lymphoid prions have different biophysical properties than those from the CNS? (2) Do lymphoid prions have different infectivity properties than CNS prions? To investigate the potential differences between peripheral PrP^CWD^ and prions formed in the CNS, we orally exposed white-tailed deer (WTD) to very low doses of infectious CWD+ saliva or brain homogenate. Tissues were collected at clinical disease, and prions from retropharyngeal lymph node and obex compared as to biophysical properties, conversion seeding efficiency, protease sensitivity, and immuno-blotting profiles. To functionally assess infectivity of peripheral and CNS-derived PrP^CWD^, we will perform both cervid prion cell assay (CPCA) and bioassay of cervidized transgenic mice via oral inoculation. These studies will illuminate previously unknown features of CWD peripheral prion pathogenesis that likely relate to the efficient horizontal transmission of CWD.

**KEYWORDS:** Chronic wasting disease; lymph node; RT-QuIC

## 104. Development of a high-throughput assay for tau purification and aggregation

Allan Yarahmady and Sue-Ann Mok

Department of Biochemistry, University of Alberta, Edmonton, Canada

**CONTACT** Allan Yarahmady yarahmad@ualberta.ca

**ABSTRACT**

**Introduction**: To study the process of tau aggregation, researchers have commonly utilized *in vitro* experiments where tau expressed and purified from *E. coli* is aggregated via addition of inducers such as heparin. However, the structures of artificially obtained tau fibrils differ from those found in actual Alzheimer’s disease patients. Differences in structures suggest that the folding pathways of *in vitro* aggregation differ from those *in vivo*. These differences call into question the relevance of *in vitro* aggregation models. Development of potential treatments that inhibit or reverse tau aggregation would likely be aided by *in vitro* derived fibrils that more closely mimic the structures observed in Alzheimer’s patients. Point-mutations of tau have been shown to significantly alter the core structure of the generated fibrils. New methodologies such as deep mutational scanning of the tau sequence could identify specific mutants that give a fibril core structure identical to that of patient-derived fibrils. In order to perform a large-scale screen of tau mutants, methods for high-throughput expression, purification, and aggregate analysis of tau were optimized.

**Materials and Methods:** Tau was expressed in three millilitre cultures in 48-well deep plates. The pellets were lysed with lysozyme and boiled to denature contaminant proteins and cell debris. The lysates were spun down and the supernatants were isolated which contain soluble tau. The kinetics of tau aggregation were studied via a thioflavin T time-course assay using heparin as an inducer. Core structures of the aggregates from the kinetic assays were characterized using trypsin digestion and visualization by SDS-PAGE.

**Results**: It was found that tau was readily expressed and purified in small-scale. The yield from high-throughput small-scale expression of tau is consistent and the aggregation propensity of tau is able to be studied using a low-volume thioflavin T kinetic assay. The trypsin-resistant core of the fibrils derived from this assay resembles those obtained through traditional purification and *in vitro* aggregation techniques.

**Conclusions:** It is possible to obtain recombinantly expressed tau of high yield and purity using small-scale methods. The aggregation kinetics and fibril core structures of these tau extracts are also able to be characterized by thioflavin T assay and trypsin digestion, respectively. As such, a high-throughput deep mutational screen of tau is possible and can be used to test if the tau fibril structures observed in Alzheimer’s can be recapitulated *in vitro*.

## 105. Differential binding of prions to vegetation

Elizabeth Triscott^a^, Alsu Kuznetsova^b^, Debbie McKenzie^a^ and Judd Aiken^c^

^a^Centre for Prions and Protein Folding Diseases, Department of Biological Sciences, University of Alberta; ^b^Department of Renewable Resources, University of Alberta; ^c^Centre for Prions and Protein Folding Diseases, Department of Agriculture Food and Nutritional Sciences, University of Alberta

**CONTACT** Elizabeth Triscott etriscot@ualberta.ca

**ABSTRACT**

Chronic wasting disease (CWD), a prion disease of cervids, is present in North America and Europe. It affects a variety of captive and free ranging animals, including mule deer, white tailed deer, reindeer, moose, and elk. Preclinical and clinical animals shed infectivity in a number of bodily fluids, including saliva, faeces, and nasal secretions. These prions are resistant to degradation and can contaminate the environment for many years. Factors such as the surface composition of materials and the mineralogy of soils can affect where infectivity accumulates in the environment. Given, however, that in most environments prions will initially contact vegetation, understanding the factors that affect the interaction between prion infectivity and vegetation is of utmost importance. We developed an assay in which infectious brain homogenate is applied to vegetation samples, which are then rinsed. The proteins are precipitated from the rinse water and the amount of PrP that rinses off each vegetation sample was compared. Given that surface composition affects prion adherence, we first compared lichen, which is coated with polysaccharides, and grass, which is covered with a waxy cuticle. Our data indicate that less prions rinse off lichen than grass. Furthermore, the binding of CWD prions to a variety of plants shows that the prion-vegetation interaction is affected by properties of the plant, e.g. microarchitecture and precise chemical composition. Freezing and drying of the plants alters this interaction. These data give us insight in how infectivity moves through the environment and has ramifications for the plausibility of environmental CWD transmission. Understanding where prions accumulate will help in developing methods for environmental monitoring and decontamination.

## 106. Long-read nanopore sequencing provides fast and accurate identification of genetic variants in the human PRNP gene

Dimitriadis Athanasios, Francois Kroll, Tracy Campbell, John Collinge, Simon Mead, Emmanuelle Viré and John Collinge

MRC Prion Unit at UCL, Institute of Prion Diseases, Queen Square House, Queen Square, University College London, London, UK

**CONTACT** Dimitriadis Athanasios athanasios.dimitriadis.16@ucl.ac.uk

**ABSTRACT**

The human prion gene (PRNP) is a 15.5 kb sequence on chromosome 20 which encodes the prion protein (PrP). It is composed of two exons flanking a single large intron. The protein coding region is a 762-bp region within the second exon. Genetic variants identified in the PRNP gene are associated with prion diseases, such as Creutzfeldt-Jakob disease (CJD). Previous work has demonstrated that the number of OctaPeptide Repeats (OPRs) in the gene coding region is in general inversely correlated with age of disease onset [1]. Similarly, it is well established that genotype of the codon 129 strongly affects disease onset, severity, and phenotype [2]. This evidence highlights the necessity of accurate genetic testing in CJD management. Here we tested how long-read sequencing technologies compare to the well-established Sanger sequencing method, using a Nanopore MinION sequencer. First, we sequenced full-length PRNP in 9 individuals known to be carrying various types of OPR insertions (OPRIs), deletions (OPRDs), and SNVs (E200K, P102L) and confirmed their genotype. To process this data, we designed a bioinformatics pipeline based on minimap2, sniffles, nanopolish, and custom R scripts to automate the analysis and filter our results. Then, we sequenced the full-length prion gene in 10 patients with sporadic CJD and characterized polymorphisms in the intronic region and the promoter of the gene. Altogether our results reveal that long-read sequencing, using MinION, allows investigating the genetic makeup of the whole PRNP in patients, including the promoter, flanking regions, intron, and both exons of the gene. We propose an accurate, quick, and user friendly method that bypasses the needs for expensive instruments and which allows for sample multiplexing. Finally, our pipeline is also able to report the results in an easily readable text format.

**KEYWORDS:**

Nanopore sequencing; genetic prion disease; bioinformatics; structural variants; single-nucleotide variations

### 

References[1]BrownK, et al.
J Geriatr Psychiatry Neurol. 2010;23(4):277–298.10.1177/089198871038357620938044[2]SchmitzM, et al. NeurobiolMol
2017;54(6):4138–4149.10.1007/s12035-016-9918-y27324792

## 107. Global analysis of protein degradation rates in prion infected cells by dynamic SILAC

Charles R. Hutti, Kevin A. Welle, Jennifer R. Hryhorenko and Sina Ghaemmaghami

University of Rochester, New York

**CONTACT** Charles R. Hutti m.shafiq@uke.de

**ABSTRACT**

Prions diseases are rare neurological disorders caused by the misfolding of cellular prion protein (PrP^C^). The misfolded conformers aggregate into cytotoxic fibrils (PrP^Sc^) that facilitate the conversion of additional prion proteins into the misfolded form. Intracellular PrP^Sc^ aggregates primarily accumulate within late endosomes and lysosomes, organelles that participate in the degradation and turnover of a large subset of the proteome. Thus, intracellular accumulation of PrP^Sc^ aggregates may globally disrupt protein degradation kinetics within infected cells. To test this hypothesis, we analyased the effect of prion infection on protein degradation rates in N2a cells using dynamic stable isotopic labelling with amino acids in cell culture (dSILAC) and bottom-up proteomics. We were able to analyse the degradation rates of ~5000 proteins, constituting the largest proteome-wide analysis of degradation rates in prion infected cells to date. As expected, the data indicate that the degradation rate of the prion protein is significantly decreased in infected cells. Indeed, we demonstrate that cellular dilution due to cell division (rather than degradation) is the dominant factor in clearance of PrP^Sc^ in infected N2a cells. Conversely, the degradation kinetics of the remainder of the N2a proteome generally increases upon infection. This effect occurs concurrently with enhancements in cellular activities of the proteasome and lysosomal hydrolases. Our data indicate that N2a cells respond to prion infection by globally upregulating the cellular protein degradation machinery. The resulting enhancement in proteome flux may play a role in the survival of N2a cells in the presence of prion infection.

## 108. Crystal structural studies of Fab fragments bound to the prion molecule and perspectives on the loop connecting β2 to α2

Pravas Kumar Baral^a^, Mridula Swayampakula^a^, Adriano Aguzzi^b^ and Michael N. G. James^a^

^a^Department of Biochemistry, Faculty of Medicine and Dentistry, University of Alberta, Edmonton, Canada; ^b^Department of Pathology, Institute of Neuropathology, University Hospital Zurich, Zurich, Switzerland

**CONTACT** Michael N. G. James michael.james@ualberta.ca

**ABSTRACT**

Our laboratories have collaborated on determining the structures of the globular domains of prion molecules from several mammalian species by X-ray crystallography. We have used the advantage of co-crystallizing the folded domains of the cellular prion proteins (PrP^C^) with the Fab fragments of several immunoglobulins raised against the mouse prion. In particular, the Fab fragments of POM1 and POM6 have adjacent but slightly different binding epitopes on the mouse PrP^C^ globular domain. Biological studies on these antibodies are quite interesting; POM1 is a highly toxic antibody whereas POM6 is an innocuous antibody. We have determined the structures of POM1 Fab bound to the folded domains of the PrP^C^ s of mouse, human, bovine, deer, elk, and Syrian hamster. The structure of mouse PrP^C^ bound to the Fab fragment of the POM6 antibody has also been determined. We have compared the loop connecting the β2 strand to the α2 helix of the PrP^C^ molecules. In all structures determined by X-ray crystallography, this loop adopts very similar structures involving a 3_10_ helix and the α2 helix initiator residue Asn171. Another very important residue in this loop is Tyr169 and its interactions with residues on helix α2. We have compared the structures of this loop determined by X-ray crystallography to the structures of this loop as determined by NMR spectroscopy. This region differs in conformation by a substantial amount in the structures determined by the two techniques (the Cα atoms of the two Ser170 residues differ in position by 5.1Å between the X-ray structure and the NMR structure). Tighter contact between the residues of the β2-α2 loop and residues from the β1- β2 region seems to be the key in keeping the folded prion domain more stable.

## 109. Analysis of N-linked glycans of ovine prion protein by liquid chromatography coupled with electrospray mass spectrometry

Natali Nakić^a^, Thanh Hoa Tran^a^, Mislav Novokmet^b^, Jelena Šimunović^b^, Olivier Andreoletti^c^, Gordan Lauc^b^ and Giuseppe Legname^a^

^a^Laboratory of Prion Biology, Department of Neuroscience, Scuola Internazionale Superiore di Studi Avanzati (SISSA), Trieste, Italy; ^b^Genos Glycoscience Research Laboratory, Zagreb, Croatia; ^c^UMR INRA ENVT 1225- IHAP, École Nationale Vétérinaire de Toulouse, Toulouse, France

**CONTACT** Natali Nakić nnakic@sissa.it Laboratory of Prion Biology, Department of Neuroscience, Scuola Internazionale Superiore di Studi Avanzati (SISSA), Trieste, Italy

**ABSTRACT**

Prion diseases are characterized by a distinct pathology in the central nervous system, however, differing from one another in clinical symptoms, incubation time, biochemical properties, and in the deposition pattern. These differences arise from the fact that PrP^C^ can acquire multiple conformationally different PrP^Sc^ states, called strains. The question of how different strains display such a diversity in disease phenotypes remains to be answered. One of the ways strains are characterized is by immunoblotting, where each strain shows a specific three band pattern. The reason for this comes from the fact that the prion protein can carry up to two N-linked glycans. The knowledge about inter-individual or inter prion strain differences in N-glycan structures is still not fully understood. The aim of this study is to differentiate individual N-glycan structures on different sites and to evaluate functional aspects of variation regarding prion protein glycosylation. The analysis was performed on VRQ/VRQ sheep brain affected with classical scrapie. The ovine PrP^Sc^ was isolated and purified by immunopurification and SDS-PAGE. The isolation of the scrapie prion protein was shown to be somewhat challenging, since glycoproteins are heterogeneously glycosylated, this leads to a decreased concentration of each analyte (each specific glycan). After obtaining a sufficient amount and purifying the protein, the di- and monoglycosylated forms of the prion protein were extracted from the gel and in-gel trypsin digestion was performed. The tryptic glycopeptides were then analysed using reverse phase liquid chromatography coupled with electrospray mass spectrometry (LC-ESI-MS). The glycan structures found on the ovine PrP^Sc^ have been shown to contain ‘brain-specific’ N-glycans, which are high in fucose and sialic acids. Other features found, that are characteristic for brain N-glycans, include hybrid-type structures, bisecting *N*-Acetylglucosamine (GlcNAc) and core (α1-6)fucose, as well as outer arm (α1-3)fucose. We were able to develop a method for isolation of ovine PrP^Sc^ from infected tissue and analysing the glycopeptides with LC-ESI-MS. With this approach we plan to compare glycan structures between different ovine prion strains in order to better understand whether or not N-glycans contribute to prion strain differences.

**KEYWORDS:** Prion; N-glycan; LC-ESI-MS

## 110. Involvement of MAPK pathway and tau in pα-syn*-induced mitotoxicity

Diego Grassi^a^^,^^b^, Natalia Diaz-Perez^c^, Laura A. Volpicelli-Daley^d^ and Corinne Ida Lasmézas^a^^,^^b^

^a^Department of Immunology and Microbiology, The Scripps Research Institute, Scripps Florida, Jupiter, FL, USA; ^b^Department of Neuroscience, The Scripps Research Institute, Scripps Florida, Jupiter, FL, USA; ^c^Private address, Palm Beach Gardens, FL, USA; ^d^Center for Neurodegeneration and Experimental Therapeutics, University of Alabama at Birmingham, Birmingham, USA

**CONTACT** Corinne Ida Lasmézas lasmezas@scripps.edu

**ABSTRACT**

We recently identified a phosphorylated form of α-synuclein, pα-syn*, as a key neurotoxic α-synuclein species found in cultured neurons, as well as in mouse and Parkinson’s disease patients’ brains. Small pα-syn* aggregates localize to mitochondria and induce mitochondrial damage and fragmentation. Herein, we investigated the molecular basis of pα-syn*-induced toxicity. By immunofluorescence, we found phosphorylated MKK4, JNK, ERK5 and p38 MAPKs in pα-syn* inclusions. pJNK colocalized with pα-syn* at mitochondria and mitochondria-associated ER membranes where it was associated with BiP and pACC1. We also found that pα-syn* aggregates are tightly associated with small ptau aggregates of similar size. Pα-syn*/ptau inclusions localized to areas of mitochondrial damage and to mitophagic vesicles, showing their role in mitochondrial toxicity, mitophagy induction, and their removal along with damaged mitochondrial fragments. Several MAPKs may act cooperatively to phosphorylate tau, notably JNK, p38, and GSK3β, a non-MAPK that was also found phosphorylated in the vicinity of pα-syn*/ptau aggregates. Thus, pα-syn* appears to be the trigger of a series of kinase mediated pathogenic events and a link between α-syn pathology and tau, another protein known to aggregate in Parkinson’s disease and other synucleinopathies.

**KEYWORDS:** Alpha-synuclein; tau; Parkinson’s disease; primary neurons; kinases; neurotoxicity; mitochondria; mitophagy

## 111. Identification of rare genetic variants associated with sporadic CJD by exome sequencing

Helen Speedy^a^, Penny Norsworthy^a^, Holger Hummerich^a^, Gary Adamson^a^, James Uphill^a^, John Collinge^a^ and Simon Mead^a^

^a^MRC Prion Unit at UCL, UCL Institute of Prion Diseases, Courtauld Building, London, UK

**CONTACT** Helen Speedy h.speedy@ucl.ac.uk

**ABSTRACT**

**Background: **Prion diseases are progressive neurodegenerative conditions characterized by the misfolding and aggregation of prion protein. Sporadic Creutzfeldt-Jakob Disease (sCJD) is the most common human prion disease. The common polymorphism at codon 129 of the *PRNP* gene influences sCJD risk; however additional genetic factors are thought to contribute to disease susceptibility. Ongoing genome-wide association studies have identified common genetic variants associated with sCJD risk. In this study we use exome sequencing to investigate the role of rare, disruptive variants as determinants of susceptibility to sCJD.

**Materials and Methods: **This study incorporates exome sequencing of an in-house set of 249 sCJD cases as well as data from 126 sCJD cases from the Medical Research Council (MRC) Brain Bank Network. Controls are derived from publically available resources.

In-house, genomic DNA was extracted from peripheral blood. Exome capture using Agilent technology and paired-end sequencing on an Illumina HiSeq2000 sequencer was performed at Source Bioscience (Nottingham, UK). Sequencing reads were aligned to the human reference genome (hg19) using Burrows-Wheeler Aligner software. Alignments were processed using the Genome Analysis Tool Kit pipeline, according to best practices. Variants were filtered according to quality and frequency metrics, as well as predicted functional consequences. Single variant and gene-based association testing was performed.

**Results: **Initial analyses of exome sequencing data failed to detect a single rare, disruptive genetic variant significantly associated with sCJD risk, whilst no individual gene in burden testing surpassed genome-wide significance. Future work will focus on exploring alternative rare variant analysis methods and the subsequent prioritization of variants for validation and follow-up in additional sCJD cases. In conjunction, we will examine the burden of clinically relevant (novel or known pathogenic) mutations in known dementia genes, deriving candidate genes from the MRC Dementia Gene Panel, UK Genetic Testing Network Clinical Panels and through manual literature curation.

**Conclusions: **We have conducted an exome sequencing study involving 375 sCJD cases to examine the role of rare, disruptive genetic variants in sCJD risk. Although no single variant or gene of significant impact was discovered in initial analyses, further exploration of this dataset is ongoing.

**KEYWORDS:** Genetics; exome; rare variant; sporadic Creutzfeldt-Jakob disease

## 112. The low levels of nerve growth factor and its upstream regulatory kinases in prion infection is reversed by resveratrol

Chao Hu^a^^,^^b^*, Cao Chen^b^^,^^c^*, Jia Chen^a^, Kang Xiao^b^^,^^c^, Jing Wang^b^^,^^c^, Qi Shi^b^^,^^c^, Yue Ma^b^, Li-Ping Gao^b^, Yue-Zhang Wu^b^, Lian Liu^a^, Ying Xia^a^, Pu Yan^a^, Adalaiti Maimaitiming^b^, Dong-Hua Zhou^b^, Li-Na Zhang^b^, Zhi-Bao Chen^a^ and Xiao-Ping Dong^b^^,^^c^^,^^d^

^a^College of Life Science and Technology, Heilongjiang Bayi Agricultural University, Daqing, China; ^b^State Key Laboratory for Infectious Disease Prevention and Control, National Institute for Viral Disease Control and Prevention, Chinese Center for Disease Control and Prevention, Beijing, China; ^c^Collaborative Innovation Center for Diagnosis and Treatment of Infectious Diseases, Zhejiang University, China; ^d^Center for Global Public Health, Chinese Center for Disease Control and Prevention, Beijing, China

**CONTACT** C. Chen chencao@ivdc.chinacdc.cn; Z. B. Chen chenzhibao_2000@sina.com; X. P. Dong dongxp238@sina.com

* These authors contributes equally to this work.

**ABSTRACT**

Up to the present, none of the specific vaccines or therapeutic drugs has been documented to be effective in human or animal prion diseases. Recently, resveratrol shows the ability to eliminate prion replication. To obtain insights into the possible changes of brain neurotrophic factor (NGF) and its upstream regulatory cascade during prion infection and after removal of prion propagation, the levels of NGF and its upstream regulatory factors in various SMB cell lines and in the mice brains inoculated with various SMB cellular lysates were assessed with various methodologies. The levels of NGF were significantly decreased *in vivo* and *in vitro*, while recovered after removal of PrP^Sc^ by resveratrol. Morphological assays revealed that the NGF signals mainly colocalized within neurons, but not in the proliferative astrocytes and microglia. The upstream positive regulatory kinases, such asp-CREB, p-CaMKIV, CaMKK2 were also remarkably decreased in the prion infected cells and mice brains, whereas the negative regulatory one,p-CaMKK2, was increased. The aberrant situations of those kinases in prion infected cell lines or mice brains could be also partially reversed by removal of prion agent. Moreover, we demonstrated that the signals of CaMKK2 and p-CaMKK2 were also distributed predominately in neurons in the brain tissues. The data here illustrate a direct linkage of abnormally repressive NGF and its upstream regulatory kinases with prion infection. Resveratrol has not only the ability to inhibit prion replication, but also to improve the expression of NGF via CaMKK2/CaMKIV cascade, which might benefit the microenvironment in brains.

**KEYWORDS:** Prion; resveratrol; NGF; CaMKIV; CaMKK2

## 113. Diagnosis of CWD in a herd of farmed red deer

Ines Walther, Antanas Staskevicius, Andrei Soutyrine and Gordon Mitchell

National & OIE Reference Laboratory for Scrapie and CWD, Canadian Food Inspection Agency, Ottawa, ON, Canada

**CONTACT** Gordon Mitchell gordon.mitchell2@canada.ca

**ABSTRACT**

Chronic wasting disease (CWD) continues to adversely impact wild and farmed cervids of North America, affecting primarily white-tailed deer (*Odocoileus virginianus*), mule deer (*Odocoileus hemionus*), and elk (*Cervus canadensis*). Red deer (*Cervus elaphus*) are closely related to elk, and occasional cases of CWD have been reported in farmed red deer in the United States and the Republic of Korea, and in a wild red deer in Norway. CWD was recently detected in farmed red deer for the first time in Canada, in a region geographically distinct from all previous cases of CWD. Understanding the diagnostic features and pathogenesis of CWD in red deer is essential to developing effective disease detection and control strategies in this species. The index case of this herd, a clinically healthy 15-month old male red deer, was initially detected during routine slaughter surveillance testing by ELISA. Confirmatory testing by immunohistochemistry (IHC) demonstrated modest aggregates of pathological prion protein (PrP^CWD^) in restricted regions of the obex of the medulla and retropharyngeal lymph nodes. Western blot of the obex homogenate revealed a banding pattern consistent with that of other Canadian CWD cases, and distinct from what has been observed in red deer experimentally infected with bovine spongiform encephalopathy. Subsequent disease control measures in this herd resulted in the testing of over 1700 red deer and an additional 10 cases of CWD were identified in females ranging in age from 18 to 28 months. All cases were positive in the obex by ELISA, IHC, and Western blot, although the extent of PrP^CWD^ deposition detected by IHC was somewhat variable. A higher degree of variability in PrP^CWD^ accumulation was found in lymphoid tissues, with PrP^CWD^ not detectable by IHC in the tonsils of several cases. Sequencing of the prion protein gene from all positive cases and a subset of negative cases identified variability at several codons (98, 168, and 226) although the extent to which these polymorphisms may influence susceptibility to infection is not yet evident. Natural transmission of CWD occurs relatively efficiently amongst cervids, facilitating the geographical dissemination of disease to previously naive populations. Determining the unique characteristics of CWD in different cervid species is needed to inform the refinement of disease management approaches.

## 114. Structural analysis of human and transgenic mouse-derived tau protein aggregates

Shelaine C. Fleck^a^^,^^b^, Ghazaleh Eskandari-Sedighi^a^^,^^b^, Razieh Kamali-Jamil^a^^,^^b^, Mirka Kasirova^c^, Chae Kim^c^, Howard Young^b^, David Westaway^a^^,^^b^^,^^d^, Jiri Safar^c^ and Holger Wille^a^^,^^b^

^a^Centre for Prions and Protein Folding Diseases, University of Alberta, Edmonton, *AB*, Canada; ^b^Department of Biochemistry, University of Alberta, Edmonton, *AB*, Canada; ^c^Departments of Pathology and Neurology, Case Western Reserve University, Cleveland, OH, USA; ^d^Department of Medicine, University of Alberta, Edmonton, *AB*, Canada

**CONTACT** Shelaine C. Fleck shelaine@ualberta.ca

**ABSTRACT**

Tauopathies are a class of neurological disorders associated with the aggregation of the tau protein into neurofibrillary tangles. The most prominent tauopathy is Alzheimer’s disease (AD), which presents as two forms: early onset (genetic, gAD) and late onset (sporadic, sAD). sAD does not have a known cause; although environmental, lifestyle, and genetic factors are thought to contribute to the disease manifestation. gAD can be caused by one of three different single gene mutations: in the amyloid precursor protein (APP), presenilin 1, or presenilin 2. In both forms of AD, the tau protein aggregates to form intracellular fibrillary tangles, which spread along neuronal networks. Amyloid-β, the product of APP proteolysis, also contributes to AD through the formation of extracellular amyloid plaques. Together, these proteins lead to the extensive neuronal death seen in AD. The structure of tau fibrils in AD, either as Paired Helical Filaments (PHFs) or Straight Filaments (SFs), was solved using cryo-electron microscopy and 3D-reconstructions.^1^ By analysing PHFs and SFs from sAD, researchers found that filament cores consist of two identical protofilaments which adopt a cross-β structure [1].Later, researchers analysed additional samples from sAD and gAD cases, which confirmed the initial findings [2]. Other tauopathies, including Pick’s disease and frontotemporal dementia with parkinsonism linked to chromosome 17 (FTDP-17), are caused solely by the misfolding of the tau protein. Each of these diseases is characterized by the dementia they cause and notable brain atrophy. The structure of tau fibrils in Pick’s disease accumulating predominantly three-repeat tau differs from AD, it forms a single protofilament with nine beta-strands [3]. The structure of tau fibrils in FTDP-17 is currently unknown. The focus of our research is to further investigate the different conformations of the tau protein in these diseases. Using EM and 3D-reconstructions we plan to examine samples from gAD and sAD cases. Currently, we have examined samples from rapidly progressive AD and a transgenic mouse line expressing four-repeat human tau protein with a P301L mutation, which is found in some FTDP-17 patients [4]. Previously, three tau fibril morphologies: straight, coiled, and twisted-ribbon, were identified [4]. Through continued examination of these fibrils using 3D-reconstructions and volumetric visualization, we hope to see more detailed differences in the structure adopted by the tau protein. This will provide insights in to the role the tau protein plays in these tauopathies and how the structural differences may contribute to the distinct disease manifestations.

### 

References[1]Fitzpatrick, et al. Nature
2017;547:185–190.10.1038/nature23002PMC555220228678775[2]Falcon, et al.
Acta Neuropathol. 2018;136:699–708.10.1007/s00401-018-1914-zPMC620873330276465[3]Falcon, et al. Nature
2018;561:137–140.[4]Eskandari-Sedighi, et al.
Mol Neurodegener. 2017;12:72.10.1186/s13024-017-0215-7PMC562842428978354

## 115. Diversity of chronic wasting disease prion strains

Camilo Duque Velásquez^a^, Elizabeth Triscott^a^, Chiye Kim^a^, Jacques Van der Merwe^a^, Samia Hannaoui^b^, Trent Bollinger^c^, Christina Carlson^d^, Sylvie Benestad^e^, Sabine Gilch^b^, Judd Aiken^f^ and Debbie McKenzie^a^

^a^Department of Biological Sciences, Centre for Prions and Protein Folding Diseases, University of Alberta, Edmonton, Canada; ^b^Department of Ecosystem and Public Health, Calgary Prion Research Unit, University of Calgary, Calgary, Canada; ^c^Department of Veterinary Pathology, Canadian Wildlife Health Cooperative, University of Saskatchewan, Saskatoon, Canada; ^d^U.S. Geological Survey-National Wildlife Health Center, Madison, WI, USA; ^e^Norwegian Veterinary Institute, Oslo and Trondheim, Norway; ^f^Department of Agricultural, Food and Nutritional Sciences, Centre for Prions and Protein Folding Diseases, University of Alberta, Edmonton, Canada

**ABSTRACT**

Chronic Wasting Disease (CWD) prions affect a variety of cervid species. The expansion of the CWD geographic range and the increasing prevalence makes CWD a concern for wildlife, livestock, and human health. Prion pathogenesis results from the template-directed misfolding of cellular prion proteins (PrP^C^) into conformational species (e.g. PrP^CWD^) associated with distinct strains. We propose that cervid cellular prion protein (PrP^C^) polymorphisms have resulted in prion conformational diversification and speciation (i.e. by adaptive radiation) resulting in emergence of novel CWD strains. New prion strain conformers emerge following transmission between cervids expressing different PrP^C^ amino acid polymorphisms [1-4]. Emergent CWD strains can have novel transmission properties that enable them to infect host species previously considered resistant, suggesting an increase in zoonotic risk as strain-conformers diversify and evolve [2, 3]. We are comparing field CWD isolates of different cervid species from various regions of North America and Norway and have identified differences in biochemical properties of PrP^CWD^, *in vitro* and *ex vivo* propagation and transmission into transgenic mice expressing deer and elk PrP (tgDeer – 96G, tgDeer – 96S, and tgElk-E226) as well as C57Bl6 mice and hamsters. Variations in PrP-res type and protease sensitivity were observed following treatment with proteinase K. Comparison of the PMCA seeding activity using deer and elk PrP^C^ as substrates revealed differences between elk CWD isolates. ElK21 cells also responded differently to various cervid prions. Transmission was efficient for all isolates in tg33 and tgElk mice; however, only a few isolates were able to propagate in host expressing S96 deer PrP^C^, which has been shown to impose a strong transmission barrier, providing a means of differential selection of CWD strains [2]. Transmission differences were observed following interspecies transmission. While some white-tailed deer and mule deer isolates failed to transmit into hamsters, other isolates transmitted with a low attack rate but considerable sub-clinical infection. These isolates had a migration pattern similar to the hamster-passaged Wisc-1 strain. Two other isolates, transmitted more efficiently into hamsters and produced two different PrP-res migration profiles compared to Wisc-1-like PrP-res. Transmission of Norwegian moose and reindeer CWD isolates is ongoing; preliminary results will be presented. Our data indicate the existence of at least five different CWD strains based on transmission properties.

### 

References[1]JohnsonCJ, et al.
Prion protein polymorphisms affect chronic wasting disease progression. PloS ONE. 2011;6(3):e17450.2144525610.1371/journal.pone.0017450PMC3060816[2]Duque VelasquezC, et al.
Deer prion proteins modulate the emergence and adaptation of chronic wasting disease strains. J Virol. 2015;89(24):12,362–12,373.10.1128/JVI.02010-15PMC466524326423950[3]HerbstA, et al.
Chronic wasting disease prion strain emergence and host range expansion. Emerging Infect Dis. 2017;23(9):1598–1600.2882038410.3201/eid2309.161474PMC5572867[4]HannaouiS, et al.
Destabilizing polymorphism in cervid prion protein hydrophobic core determines prion conformation and conversion efficiency. PloS Pathog. 2018;13(8):e1006553.10.1371/journal.ppat.1006553PMC556844528800624

## 116. Characterization of distinct SOD1 prion strains

Jacob I. Ayers^a^^,^^b^, Kristy Dillon^a^, Jeffrey Diamond^a^, Eric Fagerli^a^, Susan Fromholt^a^ and David R. Borchelt^a^

^a^Department of Neuroscience, Center for Translational Research in Neurodegenerative Disease, University of Florida, Gainesville, FL, USA; ^b^Department of Neurology, Institute for Neurodegenerative Diseases, University of California San Francisco, San Francisco, CA, USA

**CONTACT** Jacob I. Ayers jacob.ayers@ucsf.edu

**ABSTRACT**

Mutations in the Cu-Zn superoxide dismutase 1 (*SOD1*) gene are known to be causative in 10–20% of familial amyotrophic lateral sclerosis (fALS) cases. The duration of disease in these patients can vary, such that some mutations are associated with disease of less than 3 years, whereas others are associated with disease of more than 20 years. One potential explanation for this variability in disease progression is that distinct SOD1 mutations exhibit conformational strain properties that may affect their ability to propagate within the CNS. To address this, we tested various preparations containing different SOD1 mutations in *in vivo* and *ex vivo* seeding assays to investigate several SOD1 properties. We injected multiple misfolded SOD1-containing preparations within the spinal cords of mice at postnatal day P0. These recipient mice express low levels of the G85R variant of human SOD1 fused to YFP (G85R-SOD1:YFP) and are permissive for motor neuron disease induction. Once MND was induced, the spinal cords were analysed for the presence and morphology of the induced G85R-SOD1:YFP aggregates by direct fluorescence. Additionally, as an *ex vivo* assay, these SOD1-containing preparations were added to organotypic spinal cord slice cultures prepared from G85R-SOD1:YFP mice to directly visualize the induced aggregates over time. We observed both distinct SOD1:YFP aggregate pathologies and incubation periods that bred true upon repeated passages. Additionally, we observed the same SOD1:YFP aggregate structures in spinal cord slice cultures as was seen in mice, when treated with a given SOD1 containing preparation. The distinct SOD1:YFP pathologies observed suggest prion strain-like properties for SOD1. Additionally, the fact that the morphology of these structures bred true upon second passage in mice with the same genotype suggests that the formation of these aggregates are produced by conformational templated misfolding, which is responsible for the propagation of prions. These results strongly suggest SOD1 prions are capable of exhibiting conformational strain-like variability in the misfolding and propagation of SOD1-linked ALS.

## 117. Bank vole bioassay for detection of prion infection in CWD-challenged cynomolgus macaques

Karla A. Schwenke^a^, Joo-Hee Waelzlein^a^, Hermann M. Schatzl^b^^,^^c^, Walter Schulz-Schaeffer^e^, Christiane Stahl-Hennig^f^, Stefanie Czub^c^^,^^d^ and Michael Beekes^a^

^a^Robert Koch Institute, Prion and Prionoid Research Unit, ZBS 6, Berlin, Germany; ^b^University of Calgary, Calgary Prion Research Unit, Calgary, Canada; ^c^University of Calgary, Faculty of Veterinary Medicine, Calgary, Canada; ^d^Canadian Food Inspection Agency (CFIA), Lethbridge, Canada; ^e^University of Homburg/Saar, Homburg, Germany; ^f^ German Primate Center, Goettingen, Germany

**CONTACT** Karla A. Schwenke SchwenkeK@rki.de WaelzleinJ@rki.de

**ABSTRACT**

Although Chronic Wasting Disease (CWD) was detected in cervids in the late 1960s, the risk of CWD transmission from cervids to humans is still puzzling. The zoonotic potential which might emanate from CWD was studied by several research groups using a multitude of *in vitro* and *in vivo* approaches. These studies provided evidence for a significant transmission barrier for CWD between cervids and humans, but do not allow the definite exclusion of such inter-species transmission. Old- world monkeys such as macaques are currently considered the most reliable animal model for testing the zoonotic potential of CWD prions. Using oral and parenteral transmission routes, our research consortium challenged cynomolgus monkeys with characterized CWD material starting 10 years ago. To test the presence or absence of prion infection in these macaques, we injected bank voles intracerebrally with homogenized brain, spinal cord and spleen tissue from monkeys sacrificed 5–7years after CWD inoculation. Bank voles have been shown to be a universal acceptor for prions derived from various species and were found to be particularly susceptible to infection with CWD prions. Therefore, in our study bank vole bioassays provide a method of choice for the detection of prion infection in the CWD-challenged cynomolgus monkeys. Potential prion disease of macaques will be detected by assessment of prion-specific clinical signs in bank voles. The clinical assessment includes weight monitoring and routine observation of the animals according to a clinical score sheet. Further, CNS and spleen tissue will be examined *post mortem* for PrP^res^ by Western blot analysis. In case of ambiguous results, we plan to perform second passage inoculations in bank voles. Taken together, these studies are expected to contribute to a definite evaluation of the zoonotic risk of CWD.

## 118. Effect of route of infection and *PRNP* genotype on detection of preclinical infection using blood samples from BSE-infected sheep

M. K. F. Salamat^a^, O. Andreoletti^b^, S. McCutcheon^a^, A. R. A. Blanco^c^, J. C. Manson^a^, E. F. Houston^a^

^a^The Roslin Institute, R(D)SVS, University of Edinburgh, Easter Bush, Midlothian, EH25 9RG, UK; ^b^UMR INRA ENVT 1225, Ecole Nationale Vétérinaire de Toulouse, Toulouse, France; ^c^Components Development Laboratory, NHS Blood and Transplant, Cambridge, UK

**CONTACT** E. F. Houston Fiona.Houston@roslin.ed.ac.uk

**ABSTRACT**

The possibility of on-going blood-borne transmission of variant Creutzfeldt-Jakob disease (vCJD) is a major concern for public health authorities, particularly in light of uncertainties surrounding the prevalence of subclinical vCJD infection in the human population. A robust and highly sensitive assay that can be applied to readily accessible samples (e.g. blood, urine), for detection of infected individuals before the onset of disease, would be of enormous benefit in preventing further transmission of vCJD. Using sheep experimentally infected with BSE as a model, we have previously demonstrated that the protein misfolding amplification assay (PMCA) can detect seeding activity in blood samples as early as 6 months post-infection. However, we also observed significant differences in incubation period, and transmission of infection by transfusion, associated with genetic variation at *PRNP* codon 141^1^. The aim of this study was to determine the relationship between codon 141 PRNP genotype and the onset of detection of seeding activity in preclinical blood samples from orally-infected (donor) and intravenously-infected (recipient) sheep.

Samples were analysed using a microplate-based, miniaturized bead serial PMCA protocol^2−3^, with brain homogenate from tgShpXI transgenic mice expressing sheep ARQ PrP (with added dextran sulphate) as substrate, and undiluted buffy coat samples from BSE-infected and uninfected sheep as seed. In orally challenged sheep, blood samples were collected at intervals from 2 months post-infection until the onset of clinical signs. The first time point at which blood samples tested positive was very variable, ranging from 4 months to 12 months post-infection and there was no obvious correlation between codon 141 genotype and the onset of detection. In sheep infected by blood transfusion, the first preclinical blood samples were collected at 6 months post-infection, and majority of animals tested gave positive PMCA results at this time point, regardless of codon 141 *PRNP* genotype. For both orally and intravenously-infected sheep, all samples collected at time points following the onset of detection tested positive in the PMCA. The results demonstrate that the route of infection appears to have a much more significant influence than *PRNP* genotype on the onset of detection of seeding activity in preclinical blood samples. They also demonstrate that once seeding activity is detected in blood samples from infected individuals, it is consistently detected in samples from subsequent time points throughout the incubation period. This data provides valuable insights into the factors that may influence the sensitivity of detection of preclinical prion infection using blood-based assays.

### 

References[1]Tan, et al. J Gen Virol
2012;93(Pt 12): 2749–275610.1099/vir.0.039008-022971821[2]Moudjou, et al.
MBio. 2013
31;5(1):e00829–1310.1128/mBio.00829-13PMC388405724381300[3]Lacroux, et al.
PLoS Pathog. 2014;10:e1004202.10.1371/journal.ppat.1004202PMC405579024945656

## 119. Host genetic factors impacting prion diseases: genomic analysis of phenotypically distinct bovine spongiform encephalopathy cattle

Sandor Dudas^a^^,^^b^, Graham Plastow^c^, James Cross^b^ and Stefanie Czub^a^^,^^b^

^a^Canadian Food Inspection Agency, National Center for Animal Disease Lethbridge; ^b^University of Calgary, Faculty of Veterinary Medicine; ^c^University of Alberta, Agriculture, Food and Nutritional Science

**CONTACT** Sandor Dudas sandor.dudas@canada.ca

Populations and development of diagnostics and effective therapeutics for AD.

**ABSTRACT**

**Background/Introduction**: Bovine spongiform encephalopathy (BSE)-challenged cattle have variable susceptibility, incubation times and in rare cases, even different disease phenotypes; our initial results suggest this may be related to host genetic factors. Attempts have been made to link bovine spongiform encephalopathy susceptibility with genotype using BSE field cases. These sample populations have a number of uncontrolled variables making analysis difficult and likely contribute to a lack of agreement between studies.

**Materials and Methods**: To identify genetic factors impacting BSE in a more controlled population, tissue samples from BSE-challenged cattle have been collected from the APHA (UK), the FLI (Germany), the USDA (USA), and the CFIA (Canada). DNA from these tissues are currently being genotyped to identify regions of the bovine genome which are associated with different BSE phenotypes. Once identified, targeted next generation sequencing will be used to find functionally relevant genetic changes. If important polymorphisms are found, gene-edited cell culture will be used to demonstrate the impacts these genetic variants have on prion expression and disease progression.

**Results**: Genotyping of short incubation BSE challenge cattle shows a significant link with the deletion genotype at a previously uncharacterized prion gene promoter location. Functional consequences of this genotype are currently being determined using a promoter luciferase assay. Non-prion genomic locations have also been identified as unique in abnormal and/or atypical BSE cases. These include the previously identified sites BTA6-97.6Mb (ANTXR2/BMP3), BTA10-21.4Mb (CHMP4A/MYH6), BTA20-4.90Mb (CART/STC2), and BTA20-39.85Mb (GDNF/SLC45A2) [1, 2]. We have also identified additional non-prion genomic regions unique to the atypical/abnormal BSE cattle genotyped so far. One example is a missense mutation at codon 439(G->S) in the phosphoserine rich protein encoded by the kiaa0232 gene. This amino acid (439G) is part of a conserved region in species naturally affected by prion diseases including sheep, goats and deer. The kiaa0232-encoded protein has also been identified as a binding partner of YHWAE/YHWAZ, members of the 14-3-3 protein family involved in signal transduction and apoptosis [3].

**Conclusions**: Genetic analysis of cattle with variable incubation or abnormal/atypical BSE could result in the identification of proteins/pathways involved in prion disease pathogenesis. Introducing these functionally relevant polymorphisms into cell culture models will hopefully result in changes in prion production/accumulation providing evidence for an altered disease process. This will further our understanding of processes important in BSE progression and could provide future direction for selective breeding or therapeutics.

### 

References[1]Murdoch, et al.
BMC Genet. 2010;11:20.10.1186/1471-2156-11-20PMC285348520350325[2]Thomson, et al.
Prion. 2012;6(5):461–469.10.4161/pri.21866PMC351085522918267[3]Bandyopadhyay, et al. Nature methods
2010;7(10):801–805.10.1038/nmeth.1506PMC296748920936779

## 120. Detection of CWD prion seeding activity in faeces demonstrates both consistent shedding and potential for environmental monitoring

Joanne M. Tennant^a^, Manci Li^a^, Davin M. Henderson^a^, Nicholas J. Haley^b^, Candace K. Mathiason^a^ and Edward A. Hoover^a^

^a^Prion Research Center, Department of Microbiology, Immunology, and Pathology, College of Veterinary Medicine and Biomedical Sciences, Colorado State University, Fort Collins, CO, USA; ^b^Department of Microbiology and Pathology, Midwestern State University, Glendale, AZ, USA

**CONTACT** Joanne M. Tennant Joanne.tennant@rams.colostate.edu

**ABSTRACT**

**Background:** Chronic wasting disease (CWD) is spreading in susceptible cervid populations in North America, Korea, and Europe. Environmental contamination and exposure to prions is considered to be a significant factor in horizontal CWD transmission. Currently disease surveillance is limited by the necessary presence and testability of infected animals. Previous studies have shown that excreta from CWD infected deer and elk contains prion seeding activity. The detection of CWD through excreta, specifically faeces, could be beneficial in passive monitoring of CWD prevalence in endemic and emerging habitats.

**Methods**: Longitudinal collections of faeces from low-dose CWD inoculated deer were evaluated by real-time quaking induced conversion (RT-QuIC). To emulate common environmental weathering conditions, CWD-positive faecal samples were dried and exposed to UV light and prion seeding activity was evaluated by RT-QuIC before and after treatment. In addition, faeces from premises known to contain either CWD positive or negative cervids were collected and tested in RT-QuIC for prion seeding activity in a blinded experiment.

**Results:** CWD prion seeding activity was detected by RT-QuIC in faeces of five out of six low-dose CWD inoculated 96GG deer. The RT-QuIC detectable prion shedding in these deer corresponded with detection of PrP^CWD^ by IHC in rectoanal mucosa-associated lymphoid tissue (RAMALT) biopsy and prion shedding was detected during both the pre-symptomatic and symptomatic stages of disease. Prion seeding activity was little affected by drying and exposure to ultraviolet light as both rectal collected and dried faeces showed prion seeding activity. Faeces collected from the pens containing CWD positive vs. negative deer correlated with presence vs. absence prion seeding activity, respectively.

**Conclusion:** These studies demonstrate that low-dose inoculated deer shed prion seeding activity in faeces through much of their disease course. Further, the prion seeding activity in faeces was still detectable after exposure to UV light and desiccation. These findings suggest that environmental monitoring of CWD via landscape faecal deposits may be possible as a means for monitoring CWD in populations of free-ranging deer and elk.

## 121. Tau prion strain propagation and cytotoxicity defined by live-cell imaging

Sang-Gyun Kang^a^^,^^b^, Ghazaleh Eskandari-Sedighi^a^^,^^c^, Nathalie Daude^a^^,^^b^, Hristina Gapeshina^a^, Jing Yang^a^ and David Westaway^a^^,^^b^^,^^c^

^a^Centre for Prions and Protein Folding Diseases; ^b^Division of Neurology; ^c^Department of Biochemistry, University of Alberta, Edmonton, Canada

**CONTACT** Sang-Gyun Kang sanggyun.kang@ualberta.ca

**ABSTRACT**

**Introduction**: Tauopathies are a group of neurodegenerative disorders including Alzheimer’s disease (AD), progressive supranuclear palsy (PSP), corticobasal degeneration (CBD), and frontotemporal dementia (FTD). Tauopathies can be heterogeneous in clinical and pathological presentation and may be characterized by the intracellular aggregation of microtubule-associated protein Tau in neurofibrillary tangles (NFTs). Also, abnormally phosphorylated Tau becomes dissociated from neuronal microtubules and accumulates in paired helical filaments (PHFs). The conformational changes of monomeric soluble Tau into oligomers and NFTs are thought to contribute to neuronal dysfunction and eventually cell death. As prion-like propagation of Tau (‘strain’ effects) has been invoked to explain phenotypic diversity in Tau pathogenesis, we have assessed this possibility using a cell culture system.

**Materials and Methods**: Previously, we reported different pathologic variations (classes 1–5) in low-expresser TgTauP301L congenic mice encoding human Tau (2N4R, Isoform Tau-F) [1]. Sarkosyl-insoluble Tau fractions (hereafter, referred to as Tau fibrils) were prepared from mouse brains of each pathology class. HEK293 cells stably expressing a YFP-tagged human Tau 4R repeat domain (RD) fragment that includes aggregation-prone mutations (P301L/V337M, LM; proaggregation) (‘HEK-TauRD-LM-YFP cells’) [2] were transduced with liposome-protein complexes derived from each class of Tau fibrils. Recombinant Tau RD fibrils (recTauRD) were also assessed. Transduced cells were split into six-well plates for the measurement of insoluble Tau and for live-cell imaging analysis.

**Results**: Each class of Tau fibrils (including recTauRD) could show heterogeneous morphologies for Tau inclusions in the HEK293-TauRD-LM-YFP cells. Nonetheless, we observed three putative Tau prion strains from brain samples as defined by striking differences in their subcellular distribution of Tau deposits; a large mass of aggregated Tau with no specific pattern (amorphous: ES1), juxtanuclear and nuclear membrane inclusions (juxtanuclear: ES2), and granular nuclear inclusions (speckles: ES3). Live-cell imaging analysis revealed that ES1 and ES2 forms were toxic to the cells, whereas cells with ES3-type inclusions showed no cell death over a period of 16 h. Interestingly, nuclear blebs were prominent and co-localized with ES1 and ES2 Tau fluorescent signals, indicating Tau-induced nuclear damage.

**Conclusions**: TgTauP301L mice, an inbred congenic model of genetic Tauopathy, generate more than one form of biologically active Tau, as revealed by differing patterns of propagation and cytotoxicity in the cell-based assays. We also conclude that nuclear deformation contributes to neurotoxicity in a sub-set of Tauopathies.

### 

References[1]Eskandari-SedighiDaude, et al.
Mol Neurodegener. 4 October
2017;12(1):72.10.1186/s13024-017-0215-7PMC562842428978354[2]KaufmanSanders, et al.
Neuron. 23 November
2016;92(4):796–812.10.1016/j.neuron.2016.09.055PMC539236427974162

## 122. Diagnosis and biomarkers of Parkinson’s and Dementia with Lewy bodies: rapid and ultra-sensitive quantitation of disease-associated α-synuclein by αSyn RT-QuIC

Christina D. Orrù^a^, Bradley R Groveman^a^, Andrew G Hughson^a^, Lynne D Raymond^a^, Gianluigi Zanusso^b^, Bernardino Ghetti^c^, Katrina J Campbell^a^, Natália do Carmo Ferreira^a^, Jiri Safar^d^, Douglas Galasko^e^, Oskar Hansson^f^^,^^g^ and Byron Caughey^a^

^a^Laboratory of Persistent Viral Diseases, Rocky Mountain Laboratories, National Institute of Allergy and Infectious Diseases, National Institutes of Health, Hamilton, MT, USA; ^b^Department of Neurosciences, Biomedicine and Movement Sciences, University of Verona, Verona, Italy; ^c^Indiana University School of Medicine, Indianapolis, IN, USA; ^d^Department of Pathology, Case Western Reserve University School of Medicine, Cleveland, OH, USA; ^e^Department of Neurosciences, University of California-San Diego, La Jolla, CA, USA; ^f^Clinical Memory Research Unit, Department of Clinical Sciences Malmö, Lund University, Lund, Sweden; ^g^Memory Clinic, Skåne University Hospital, Malmö, Sweden

**CONTACT** Christina D. Orrù christina.orru@nih.gov

**ABSTRACT**

Diagnosis of synucleinopathies such as Parkinson disease and dementia with Lewy bodies can be aided by assays for the detection and quantification of pathogenic disease-associated forms of α-synuclein (αSyn^D^). These assays need to be sufficiently sensitive, specific, and practical for analysis of accessible diagnostic samples. Two recent αSyn^D^ seed amplification assays have provided valuable prototypes for detection of αSyn^D^ in patients’ cerebrospinal fluid (CSF). However, these assays require 5–13 days to perform. We have developed an improved α-synuclein real time quaking-induced conversion (αSyn RT-QuIC) assay with similar sensitivity and specificity that provides quantitative results in 1–2 days. Our blinded analysis of cerebrospinal fluid from 29 synucleinopathy cases (12 Parkinson’s and 17 dementia with Lewy bodies) and 31 non-synucleinopathy controls, including 16 Alzheimer’s cases, yielded 93% diagnostic sensitivity and 100% specificity. End-point dilution analyses detected αSyn^D^ in as little as 0.2 μL of CSF. Furthermore, our test allowed us to quantitate relative amounts of αSyn^D^ in CSF samples. We are currently testing additional well-characterized blinded panels of CSF samples from individuals diagnosed with synucleinopathies and controls. Moreover, we have also developed a capture protocol for the detection of αSyn^D^ in human plasma spiked with αSyn positive brain homogenate and in endogenous CSF samples from individuals affected by synucleinopathies. We can now isolate αSyn^D^ from several biological samples for sensitive and specific detection by αSyn RT-QuIC.

## 123. Detection of prion seeds throughout the eyes of sporadic Creutzfeldt-Jakob disease patients

Christina Orru^a^, Katrin Soldau^b^, Christian Cordano^c^, Jorge Llibre-Guerra^d^, Ari J. Green^e^, Henry Sanchez^c^, Bradley R. Groveman^a^, Steven D. Edland^f,g^, Jiri G. Safar^h,i^, Jonathan H. Lin^b^, Byron Caughey^a^, Michael D. Geschwind^c^ and Christina J. Sigurdson^b,j^

^a^Laboratory of Persistent Viral Diseases, Rocky Mountain Laboratories, National Institute of Allergy and Infectious Diseases (NIAID), National Institutes of Health (NIH), Hamilton, MT, USA; ^b^Department of Pathology, University of California, San Diego, La Jolla, CA USA; ^c^Department of Neurology, Memory and Aging Center, University of California, San Francisco (UCSF), San Francisco, CA, USA; ^d^Cognitive and Behavioral Research Unit, National Institute of Neurology, Havana, Cuba; ^e^Department of Neurology, Multiple Sclerosis Center, University of California, San Francisco (UCSF), San Francisco, CA, USA; ^f^Departments of Family Medicine & Public Health and ^g^Neurosciences, University of California, San Diego, La Jolla, CA USA; ^h^Departments of Pathology and ^i^Neurology, Case Western Reserve University, Cleveland, OH, USA; ^j^Department of Pathology, Immunology, and Microbiology, University of California, Davis, Davis, CA, USA

**CONTACT** Christina Orru christina.orru@nih.gov

Orrù and Soldau, mBIO 2018 9:e02095–18. https://doi.org/10.1128/ mBio.02095-18

**ABSTRACT**

Cases of iatrogenic Creutzfeldt-Jakob disease (CJD) have been reported from corneal transplants, yet the levels of prions in the eye remain unknown. Approximately, 40% of sporadic CJD patients develop visual symptoms and often seek ophthalmological consultation. This study probed the occurrence, level, and distribution of prions in the eyes of patients with sCJD, the most common prion disease in humans. We used the highly sensitive real time quaking-induced conversion (RT-QuIC) assay to measure post-mortem levels of prion seeding activity in cornea, lens, ocular fluid, retina, choroid, sclera, optic nerve, and extraocular muscle. This is the largest series of sCJD patient eyes studied by any assay to date. We detected prion seeding activity in 100% of sCJD eyes representing three common sCJD subtypes with a four log-fold range of seeding activity observed across individuals. The retina consistently showed the highest prion seeding activity. Within the retina, prion deposits were also detected by immunohistochemistry in the outer plexiform layer, and in some eyes the inner plexiform layer. With increased distance from the brain prion seed levels by RT-QuIC declined in eye tissues. Collectively, these results reveal that sCJD patients accumulate prions throughout the eyes, indicating the potential diagnostic utility as well as the possible biohazard.

## 124. Longitudinal studies of CWD after low-dose oral exposure in white-tailed deer

Clare E. Hoover*, Nathaniel D. Denkers, Kristen A. Davenport, Davin M. Henderson, Amy V. Nalls, Erin McNulty, Joanne Tennant, Sarah Cooper, Manci Li, Lauren Bracchi, Candace K. Mathiason and Edward A. Hoover

Prion Research Center, Department of Microbiology, Immunology, and Pathology, College of Veterinary Medicine and Biomedical Sciences, Colorado State University, Fort Collins, CO, USA

**CONTACT** Edward A. Hoover edward.hoover@colostate.edu

*Present address: AstraZeneca, Waltham, NJ

**ABSTRACT**

**Background**: The facile transmission of chronic wasting disease (CWD) in North America, Asia and Europe continues despite exposure to very low concentrations of prions shed by infected cervids in secreta and excreta. Explanations for this enigma could be that excreted prions may have enhanced infectivity and/or that the infectious prion dose is just quite low. Historical studies exploring CWD pathogenesis in deer have used exposures to CWD-positive brain homogenates containing from 0.5 to 10 g of brain – doses much higher than those measured in excreta, and thereby likely to ever occur in nature. We thereby sought to more closely emulate natural exposure context in our current experimental studies.

**Methods**: To explore how the origin or infectious dose of CWD prions may influence disease transmission or pathogenesis, we orally exposed cohorts of *n* = 4/group white-tailed deer to low doses of CWD presented as either CWD-positive brain homogenate (containing either 1 mg or 300 ng of brain) or to an amount of pooled saliva from CWD+ donors and containing prion seeding activity (by RT-QuIC) equivalent to 300 ng of CWD+ brain [1]. Inoculated deer were then longitudinally monitored for: (a) onset of prion infection in biopsied tonsil and recto-anal lymphoid tissues by RT-QuIC and immunohistochemistry; (b) prion shedding in saliva and faeces (by RT-QuIC); (c) onset of clinical signs (weekly observation and scoring); and (d) time to clinical disease.

**Results**: We first detected CWD infection by RT-QuIC seeding activity in tonsil biopsies at 6 or 9 months post exposure, in all inoculation cohorts. Prion shedding in saliva was detected (by RT-QuIC or PMCA-RT-QuIC [2]) concurrent with the first positive tonsil biopsy. Detection of prion seeding activity in faeces was less common overall and its onset observed after positivity in recto-anal lymphoid tissue biopsies. In comparison to historical studies, in which >1,000-fold higher inoculation doses were used, the time to first detection on infection (by tonsil biopsy) was prolonged by ~6 months. However, among the low-dose inoculation cohorts in this study, neither the attack rate, prion shedding profile, nor long-term disease course differed significantly, including the deer exposed to prions of saliva vs. brain origin.

**Conclusions**: These studies demonstrate that (a) much lower doses of CWD prions that have been used historically for point source exposure studies are sufficient to induce prion infection, shedding, and disease; and (b) although time to first detectable positivity is lengthened, the qualitative aspects of disease pathogenesis thereafter are indistinguishable by inoculum source or exposure magnitude.

Our future studies will aim at establishing the minimum oral infectious dose for CWD in deer and on exploring exposure cofactors.

### 

References[1]HooverCE, et al.
in preparation, 2019[2]DavenportKA, et al.
J Clin Microbiol. 27 August
2018;56(9).10.1128/JCM.00947-18PMC611345429950332

### 

Supported by NIH R01-NS-061902, P01-AI-077774, F30-ODO-118,143, T32-OD0-10,437

## 125. Bioassay confirms the relevance of surf-PMCA for human prion decontamination studies

Maxime Bélondrade^a^, Simon Nicot^a^, Christelle Jas^a^^,^^b^, Vincent Béringue^b^, Chantal Fournier-Wirth^a^, Sylvain Lehmann^c^ and Daisy Bougard^a^*

^a^Pathogenesis and control of chronic infections, Etablissement Français du Sang, Inserm, Université de Montpellier, Montpellier, France; ^b^Institut National de la Recherche Agronomique (INRA), UR892, Virologie Immunologie Moléculaires, Jouy-en-Josas, France; ^c^CHRU de Montpellier and Université de Montpellier, IRMB, INSERM U1183, Laboratoire de Biochimie Protéomique Clinique, Montpellier, France

**CONTACT** Daisy Bougard daisy.bougard@efs.sante.fr

Abstract not available.

## 126. Dickinson, the forgotten man of the prion story

James Hope^a^ and Jason Bartz^b^

^a^One Health, Norwich, Norfolk, UK; ^b^Creighton University, Omaha, Nebraska, USA

**CONTACT** James Hope jameshope8@outlook.com


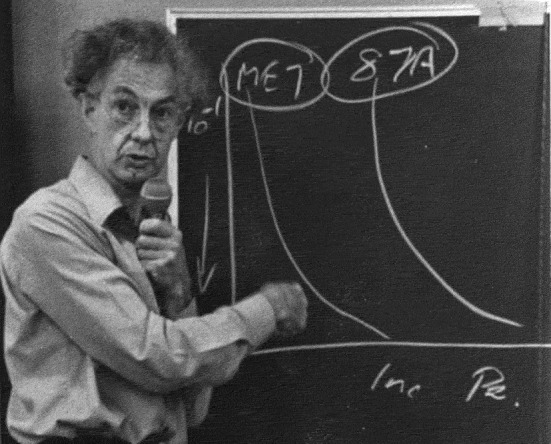


Alan Dickinson, geneticist, born 30 March 1930; died 27 September 2017

Alan Dickinson died just after the Prion 2017 meeting in Edinburgh [1] and, sadly, his iconoclastic stance (on everything) and working tenet of the importance of being awkward, has meant his contribution to our prion world has faded much too quickly. To redress the balance a wee bit, let’s be clear: he was a fantastic geneticist and, with Hugh Fraser and Moira Bruce, sorted out much of the confusion and mystery of scrapie in sheep that raged in the late 1950s and 1960s. His post-graduate training in genetics was meticulously applied to the breeding of mice and sheep, and he was able to biologically define alleles of a genetic locus (Sinc in mice) in both which appear to control the timing and tempo of the inoculated (and natural) disease with clockwork precision. The methods of strain typing using vacuolar pathology and mice of defined prion protein genotype (PrP is a product of the Sinc locus) are still used today in prion and prion-like disease research, and strain theory in TSEs and other neurodegenerative disease continues to intrigue and test our understanding of mechanisms of propagation, blocking and phenotype stability. It is interesting to speculate how much of his ‘awkward’ legacy has been explained by prions. What is our molecular explanation for breakdown of the 87A strain to ME7? How does one slow replicating strain prevent disease with the characteristics of a later-inoculated, short-incubation period prion? Current work does indicate that prion strains compete for PrP^C^ in a common population of cells [2], consistent with AGD’s original ‘replication site’ hypothesis [3]. And ‘strain-specific’ co-factors, the prion’s host-independent genome, are still sought after today, but remain elusive [4]. Crucially, he did use his insights for practical good, *inter alia* predicting the dangers of human growth hormone (hGH) therapy for pituitary dwarfism, and validating methods for its safe purification [5], decades before the current controversy of hGH therapy, ‘Alzheimer’ prions and Aβ pathology. He died continuing to search for an elusive second molecule to explain these biological phenomena – the iconoclast in him never accepted PrP (or the nomenclature) as the sole component of a naturally-infectious prion – and he ruffled a few feathers in old age by continually defining molecular biology as ‘fishing without a licence’, rephrasing Chargaff’s view of it as ‘the practice of biochemistry without a licence’ [6]. Even so, his life’s work deserves recognition to this day in the bibliographies of our publications.

**KEYWORDS:** virino; strains; *Sinc*; mouse; genetics; vacuolation; stability classes; hGH; history of science

### 

References[1]GuardianGreen, E
(Manchester, UK) 23 November 2017. Geneticist who carried out groundbreaking research into the behaviour of diseases including scrapie and CJD [cited 15 February
2018].[2]EcklandTE, ShikiyaRA, BartzJC.
Independent amplification of co-infected long incubation period low conversion efficiency prion strains. PLoS Pathog. 2018;14:e1007323.3033585410.1371/journal.ppat.1007323PMC6193734[3]DickinsonAG, OutramGW.
The scrapie replication-site hypothesis and its implications for pathogenesis. In: PrusinerSB, HadlowWJ, editor, Slow transmissible diseases of the central nervous system. Vol. 2. New York, NY: Academic Press, Inc.; 1979 p. 13–31.[4]SaáP, SferrazzaGF, OttenbergG, et al.
Strain-specific role of RNAs in prion replication. J Virol. 2012;86:10494–10,504.10.1128/JVI.01286-12PMC345731822811520[5]TaylorDM, et al.
Preparation of growth hormone free from contamination with unconventional slow viruses. Lancet. 1985
8
3; 8449:260–262.10.1016/s0140-6736(85)90302-22862429[6]GuardianWright, P.
(Manchester, UK) 2 July 2002. Disillusioned biochemist who pioneered our understanding of DNA [cited 7 February
2019].

## 127. Focal and diverse deposition in a mouse model of 4-repeat tauopathy is paralleled by distinct biochemical and cellular signatures; close relationship to Tau species in P301L patients

D. Westaway, N. Daude, G. Eskandari-Sedighi, S. Kang, C. Kim, S. Fleck, J. Yang, H. Wille, E. Gelpi and J. G Safar

**ABSTRACT**

**Background**: Mutations in the Tau (*MAPT*) gene can cause neurodegenerative diseases such as frontotemporal lobar degeneration (FTLD) but, strikingly, patients with the same mutation have very different clinical phenotypes; the cause of this diversity is not understood. Heterogeneities have also been observed in a transgenic (Tg) mouse line expressing low levels of mutant human P301L Tau and backcrosses of founder stocks of mice to inbred genetic backgrounds to attenuate contributions from modifier loci failed to eliminate variability in disease-related phenotypes (Eskandari-Sedighi et al., 2017). Here biochemical and cellular assays have been used to assess chemical and biological characteristics of P301L Tau species from Tg.P301L mice and from human FTLD.

**Methods**: We used Tg.P301L mice from C57BL/6Tac, 129/SvEvTac and FVB/NJ genetic backgrounds and brain material from eight Iberian FTLD patients with the same P301L mutation and similar *MAPT* locus haplotypes (Borrego-Ecija et al., 2017). Tau species were profiled using microfluidic western assays, immunostaining, conformation-dependent immunoassays (CDI), conformational stability assays (CSAs), trypsin digestion, as well as by kinetic and morphological performance measures in a cell-based seeding assay. Electron microscopy (EM) was used to investigate fibril morphology.

**Results**: TgP301L mice yielded six patterns of Tau deposition in neurons and astrocytes; these included brain stem and focal deposition and in Class VI, present in half of aged mice, tangle-like deposits in the *locus coeruleus*. As defined by biochemical assays, three distinct Tau signatures were present in the brains of inbred Tg mice, these signatures often correlating with pathology classes. EM revealed that brains of Classes I, II, III and IV animals exhibit at least three distinguishable fibril morphology; the most abundant being straight filaments with twisted ribbon-like filament and coiled filaments being present in some classes. Analyses of detergent-insoluble tau in the frontal cortex of FTLD patients demonstrated remarkable conformational heterogeneity with at least two Tau signatures that mimicked a CSA pattern found in a subset of aged TgP301L mice. Toxicity above baseline in transfected cells was greatest with brain-derived material, with different preparations differing in their kinetics of cell loss and fluorescent signals at the nuclear margin.

**Conclusions**: Heterogeneities in tau pathology and chemical signatures were observed within congenic stocks of TgP301L mice and in different P301L patients; crucially, signatures present in patients also manifested in a subset of mice. We conclude that our Tg model reveals non-synchronous, stochastic chemical events that initiate tau deposition and that different toxicity profiles of Tau isolates affect their ability to propagate.

## 128. Strain profiles of CJD in venison consumers and non-consumers from Alberta and Saskatchewan

Jennifer Myskiw^a^^,^^b^, Lise Lamourieux^a^, Michael Coulthart^c^, Valerie Sim^d^ and Stephanie Booth^a^^,^^b^

^a^Zoonotic Diseases and Special Pathogens, National Microbiology Laboratory, Public Health Agency of Canada, Winnipeg; ^b^Department of Medical Microbiology and Infectious Diseases, University of Manitoba, Winnipeg; ^c^Canadian CJD Surveillance System, Public Health Agency of Canada, Ottawa; ^d^Division of Neurology, Department of Medicine Centre for Prions and Protein Folding Diseases, University of Alberta, Edmonton

**CONTACT** Jennifer Myskiw Myskiwj@myumanitoba.ca

**ABSTRACT**

Chronic wasting disease (CWD) is a fatal neurodegenerative disease that is spreading rapidly through wild cervid populations in Alberta and Saskatchewan. The epidemic of CWD in Canada not only has implications for tourism and hunting but it is also a concern over possible zoonotic transmission to humans who eat venison from infected deer. When bovine spongiform encephalopathy (BSE) reached epidemic proportions, variant CJD was identified as an acquired form of BSE due to the unique biochemical fingerprint of the pathologic prion protein (PrP). While the Canadian CJD Surveillance System (CJDSS) is staying vigilant to identify CWD human cases, it is impossible to know what the phenotype of human CWD prions would present with or whether they would be distinct from sporadic CJD cases. Additionally, there is currently no convenient laboratory model to study these differences experimentally. For these reasons we are undertaking a systematic analysis of the molecular diversity of CJD cases in patients who resided in Alberta and Saskatchewan at their time of death. We are comparing CJD profiles of venison consumers and non-consumers using a variety of clinical imaging, pathological and biochemical markers. Firstly, MRIs have been reviewed and neuropathology of over 40 patients for whom CJD was confirmed as the cause of death analysed. In this way we are able to select clinically affected areas for further biochemical analysis of prion protein. We will present data including our analysis to date of levels of protease sensitive and resistant prion protein, temperature and detergent denaturation profiles, and glycoform typing. By combining these methodologies, we intend to extend the baseline CJD typing that is already completed by the CJDSS. Importantly we aim to increase our understanding of the human prion strains that affect the Canadian population. By doing so, we will establish a baseline for the identification of any future atypical CJD cases. For example, those that could arise as a result of exposure to CWD.

## 129. Deregulation of SINE RNA levels in Alzheimer’s disease patients

Yubo Cheng^a^, Luke Saville^a^, Babita Gollen^a^, Mitchell Liam^b^, Christopher Isaac^a^, Jogender Mehla^b^, Mohajerani Majid^b^ and Athanasios Zovoilis^a,b^

^a^Department of Chemistry and Biochemistry, University of Lethbridge, Lethbridge, Canada; ^b^Department of Neuroscience, University of Lethbridge, Lethbridge, Canada

**CONTACT** Athanasios Zovoilis athanasios.zovoilis@uleth.ca

**ABSTRACT**

As the human life span increases, the number of people in Canada suffering from Alzheimer’s disease (AD) is expected to rise dramatically. A number of studies have revealed that gene expression networks in AD are deregulated. However, the molecular mechanisms associated with this deregulation remain largely unknown. In a previous work, we have shown that mechanisms involving non protein-coding RNAs, such as Short Interspersed Nuclear Element (SINE) RNAs (Zovoilis et al., Cell 2016) are key in maintaining transcriptional homeostasis in cells. Recently, we showed that in mouse, RNA from one of the most frequent SINE repeat classes (B2 RNA) controls expression of immediate early genes (IEGs) through binding and inhibition of RNA Polymerase II. However, it remained unclear whether deregulation of this mechanism could underlie a pathological condition in human. Here, we applied an integrative RNA genomics and bioinformatics approach to dissect any connection between SINE RNAs and Alzheimer’s disease. Our study revealed that SINE RNAs are subject to abnormal processing in human AD patients and are connected with deregulation of gene expression.

## 130. Evaluation of the sensitivity of different RT-QuIC substrates in detecting and characterizing CWD prions in brains of Norwegian cervids

Edoardo Bistaffa^a^, Tram Thu Vuong^b^, Linh Tran^b^, Federico Angelo Cazzaniga^a^, Giulia Salzano^c^, Giuseppe Legname^c^, Giorgio Giaccone^a^, Sylvie Benestad^b^ and Fabio Moda^a^

^a^Division of Neurology 5 and Neuropathology, Fondazione IRCCS Istituto Neurologico Carlo Besta, Milano, Italy; ^b^Norwegian Veterinary Institute, Oslo, Norway; ^c^Laboratory of Prion Biology, Department of Neuroscience, Scuola Internazionale Superiore di Studi Avanzati (SISSA), Trieste, Italy

**CONTACT** Tram Thu Vuong tram.thu.vuong@vetinst.no

**ABSTRACT**

Chronic wasting disease (CWD) is a highly contagious prion pathology affecting captive and free-ranging cervid populations. From its first description 50 years ago, CWD has been detected in United States, Canada, South Korea and, most recently, in Norway. At present, we do not know if these diseases are caused by the same prion strain or whether they spontaneously arose in different countries. CWD spreading among cervids is one of the most important issue for public health due to the ability of this agent to infect a large number of animals and the possible transmission to other animal species, including humans and ovine. Indeed, possible transmission of CWD to ovine that share, in some countries, the same geographic areas of CWD infected animals cannot be excluded. For this reason, it is of fundamental importance to develop a strategy allowing identification of CWD infected samples and verify their ability to propagate within the same or different species. To this aim, we are optimizing RT-QuIC assay coupled with specific biochemical analysis for evaluating whether different CWD samples could produce final reactions products with peculiar differences useful for CWD strain’s identification. RT-QuIC were performed using different substrates of reaction (truncated PrP protein from hamster, reindeer and deer species) for evaluating their sensitivity and specificity in detecting CWD in brains of Norwegian moose, red deer and reindeer. Moreover, through biochemical assessments (PK digestion, guanidine hydrochloride evaluation and use of antibodies against different PrP epitopes), we evaluated whether and to what extent final RT-QuIC products were able to acquire distinct biochemical features according to the strain of CWD. Preliminaryresults showed that deer PrP detected CWD in all species with higher efficiency compared to hamster and reindeer. Notably, truncated deer-PrP was able to rapidly detect positive samples (within 3 h from the beginning of the reaction) compared to the other substrates. Although with less sensitivity, reindeer-PrP identified red deer, reindeer and moose CWD, with this latter being characterized by lower resistance to PK digestion and different PrP banding profile (at Western blot) compared to the others. Therefore, RT-QuIC can be exploited for detecting CWD infected species with high sensitivity and specificity, while biochemical data seem to indicate the possibility of identifying the species of CWD origin. If confirmed, such information will be of fundamental importance for planning actions to contain the spread of the infection (especially to other species, like ovines) in CWD affected regions.

**KEYWORDS:** Prion; CWD; RT-QuIC

## 131. Targeting a regulatory subunit of protein phosphatase 1 by Sephin-1 reduces prion infection in neuronal cells

Simrika Thapa, Dalia. H. Abdelaziz, Basant Abdulrahman and Hermann M. Schatzl

Department of Comparative Biology & Experimental Medicine, Hotchkiss Brain Institute and Calgary Prion Research Unit, University of Calgary, Calgary, Alberta, Canada

**CONTACT** Simrika Thapa sthapa@ucalgary.ca

**ABSTRACT**

Prion diseases are infectious and fatal neurodegenerative diseases in human and animals caused by misfolding of the cellular prion protein (PrP^C^) into the infectious isoform PrP^Sc^. These diseases have the potential to transmit within or between species, and no cure is available to date. Targeting the unfolded protein response (UPR) as an anti-prion therapeutic approach has been widely reported for prion diseases. Here, we describe the anti-prion effect of Sephin-1 which has been shown to protect in mouse models of protein misfolding diseases including amyotrophic lateral sclerosis by selectively inhibiting the stress-induced regulatory subunit of protein phosphatase 1, thus prolonging the eIF2a phosphorylation. In this study, we describe that Sephin-1 significantly reduced PrP^Sc^ in persistently prion-infected neuronal cells, 22L-ScN2a, RML-ScN2a and RML-ScCAD5 cells. Moreover, prion seeding activity was reduced in treated cells in RT-QuIC assay. No effect of Sephin-1 on the levels of PrP^C^ expression was observed in uninfected N2a cells. Importantly, we found that Sephin-1 significantly overcame the ER stress induced in treated cells as measured by lower expression levels of stress-induced aberrant PrP and stress markers (BiP and CHOP). At present, we are investigating effectiveness of intraperitoneal Sephin-1 treatment against RML prions *in vivo*. These results suggest that Sephin-1 could be a potential anti-prion drug selectively targeting one component of UPR pathway.

## 132. Metformin ameliorates prion infection in neuronal cells by enhancing autophagy

Dalia Abdelaziz, Simrika Thapa, Basant Abdulrahman and Hermann Schatzl

Department Comparative Biology & Experimental Medicine, Faculty of Veterinary Medicine, Calgary Prion Research Unit, and Hotchkiss Brain Institute, University of Calgary, Calgary, Alberta, Canada.

**CONTACT** Dalia Abdelaziz dalia.abdelaziz@ucalgary.ca

**ABSTRACT**

Metformin is the first-line medication for the treatment of type 2 diabetes and a well-known AMPK activator. Recently, substantial efforts have been invested in establishing the role of metformin in the treatment of neurodegenerative diseases, such as Alzheimer’s, amnestic mild cognitive impairment, Parkinson’s and Huntington’s disease. Metformin was shown to be neuroprotective and to promote adult neurogenesis under both physiological and pathological conditions *in vivo*. This prompted us to investigate the anti-prion effectiveness of metformin in two different persistently prion infected neuronal cell lines (RML-CAD5 and 22L-N2a). We found that metformin treatment, at non-toxic concentrations of 0.5 and 1 mM, decreased the PrP^Sc^ load in both 22L-N2a and RML-CAD5 cells, as shown by lower signals of PK resistant PrP in immunoblots and less conversion activity in RT-QuIC. On the other hand, metformin treatment had no effect on PrP^C^ levels in uninfected N2a cells. Since there is a solid body of evidence showing autophagy enhancing activity of metformin, we decided to test the level of autophagy markers in the treated cells. Notably, the LC3 II signal was found to be upregulated in both CAD5 and N2a cells after treatment with metformin, which confirms the autophagy inducing effect of metformin in both neuronal cell lines. Currently, we are investigating the effect of intraperitoneal metformin treatment on the survival of RML-infected mice. In conclusion, our findings describe for the first time the anti-prion effect of metformin, which can be at least partially explained by its autophagy inducing activity. In future studies, we are planning to do synergistic combination of metformin with other anti-prion candidate drugs to test if this will improve the anti-prion efficacy via working on different pathways.

## 133. Neurofilaments in blood: a new promising biomarker of scrapie

Marco Rossi^a^, Elena Bozzetta^a^, Cristina Casalone^a^, Cristiano Corona^a^, Maria C. Cavarretta^a^, Henrik Zetterberg^b^, Kaj Blennow^b^ and Daniela Meloni^a^

^a^Istituto zooprofilattico del Piemonte Liguria e Valle d’Aosta, Turin, Italy; ^b^Institute of neuroscience and physiology, University of Gothenburg, Gothenburg, Sweden

**CONTACT** Marco Rossi marco.rossi@izsto.it

**ABSTRACT**

**Introduction**: *In vivo* accurate, easy-to-perform, and early diagnostic tests are crucial for the identification of prion subjects during their early or preclinical phase of disease and finally for managing public health issues. Excellent biomarkers for prion diseases have been identified in the cerebrospinal fluid (CSF), but this tissue is difficult to get from sheep and is therefore of little utility for the control of scrapie. Recently, the measurement of neurofilaments light chain (NF-L), a surrogate biomarker of neuroaxonal degeneration, by the SIMOA ultra-sensitive single molecule array technology, revealed high NF-L levels in blood of patients with preclinical stages of human prion diseases. In this work, we adapted the SIMOA test for the measurement of NF-L in blood of scrapie infected sheep and healthy animals and it showed a promising utility of blood serum NF-L as biomarker of scrapie in sheep.

**Materials and Methods**: A set of 20 sheep blood serum blinded samples were tested by the SIMOA technology. Nine samples were from scrapie affected sheep and 11 from healthy animals. The gold standard was assessed by testing the *medulla oblongata* of sheep both with an EU approved Elisa rapid test followed by a confirmatory Western blot assay.

**Results**: Results revealed a clear capacity of Simoa NFL test to classify the two groups of samples according to the presence or absence of scrapie infection. All the positive samples according to the gold standard showed high NF-L levels, while only one of 10 samples from healthy animals showed NF-L values overlapping with positive samples.

**Conclusion**: These data suggest that neurofilaments are sensitive and specific blood biomarkers for the diagnosis of scrapie in sheep and might represent promising tools for an early diagnosis. We are now working for establishing the basal levels of NF-L in a larger group of healthy sheep and in sheep with different types of diseases other than scrapie. We are also working for determining the variation of NF-L levels in experimentally scrapie-infected sheep at various intervals of the incubation period and at the terminal stages of disease.

## 134. The transmissible spongiform encephalopathies surveillance in small ruminants in Romania for a period of 10 years

Florica Bărbuceanu^a^, Ioana Neghirla^b^, Theodora Chesnoiu^b^, Cristina Diaconu^a^, Stefania-Felicia Barbuceanu^c^ and G. Predoi^d^

^a^Institute for Diagnosis and Animal Health Bucharest, București, Romania; ^b^National Sanitary Veterinary and Food Safety Authority, București, Romania; ^c^Faculty of Pharmacy, Carol Davila University of Medicine and Pharmacy, București, Romania; ^d^Faculty of Veterinary Medicine Bucharest, București, Romania

**CONTACT** Florica Bărbuceanu barbuceanu.florica@idah.ro

**ABSTRACT**

The scarpie belongs to the TSE group and is a fatal degenerative disease, affecting the central nervous system of ovine and caprine. There are two types of scrapie: classical and atypical. The classical scrapie affects the animals aged from 2 to 5 years and is very contageous, while the atypical scrapie affects the animals older than 5 years and is considered to have a low degree of infectivity. The aim of this study is to present information on the Romanian TSE Surveillance Program, performed in compliance with the EU Commission and OIE requirements, as well as the TSE test results in small ruminants (animals of the category clinical suspicions, dead animals and animals slaughtered normally) performed in the national net for transmissible spongiform encephalopathies, between 2007 and 2017. Between 2007 and 2017 were tested in Romania approximatively 339,643 small ruminants. All the 787 cases of scrapie were confirmed by the TSE NRL (National Reference Laboratory) by immunoblotting and immunohistochemical tests, with the performance of the discriminatory and genotyping testing. All animals diagnosed with scrapie were affected by the classical form. For all suspicion cases, a differential diagnosis was performed against rabies, Aujeszky disease and listeriosis.

**KEYWORDS:** Classical scrapie; Romanian TSE surveillance program; NRL-TSE

## 135. Characterization of serum miRNA in prion infected elk and hamsters reveal putative biomarkers

Jessy Slota^a^, Sarah Medina^b^, Megan Klassen^c^ and Stephanie Booth^b^

^a^University of Manitoba, Winnipeg, Canada; ^b^Public Health Agency of Canada, Ottawa, Canada; ^c^Public Health Agency of Canada – NML

**CONTACT** Jessy Slota slotaj@myumanitoba.ca

**ABSTRACT**

Prion diseases are a group of fatal neurodegenerative diseases that arise in both humans and animals following misfolding of the cellular prion protein into a disease associated structural conformation. Afflicted individuals experience a decline in mental capacity owing to the death of brain cells and spongiform change, although the phenotypic characteristics of these diseases vary widely. This heterogeneity as well as a lack of robust diagnostic methodologies make the diagnosis of prion diseases very challenging. It is especially important to develop better diagnostic assays for prion diseases as diagnosis must precede the onset of clinical signs and symptoms as well as permanent neuronal damage for any potential therapeutics to be of benefit. Micro-RNAs (miRNAs) are small non-coding RNA molecules which regulate expression of mRNA through the RNA interference pathway and may be found within cells as well as circulating biological fluids such as serum and cerebrospinal fluid (CSF). Identification of signatures of altered levels of specific miRNAs within biofluids during disease states is a promising new avenue of research which may fuel the development of improved diagnostic assays. Therefore, identifying patterns of miRNAs deregulated within biofluids such as serum during prion infection may lead to the discovery of novel biomarkers, aiding in the diagnosis of these diseases. In the present study, Illumina next-generation sequencing was used to characterize the miRNA content of serum taken from Elk with chronic wasting disease (CWD) as well as hamsters infected with 263K scrapie prions. This analysis was also applied to CSF taken from the 263K infected hamsters. Multiple miRNAs were detected to be altered between both serum and CSF isolated from the infected animals compared to the healthy control animals. Subsequent analysis revealed a pattern of five miRNAs which were consistently down-regulated in serum samples taken from both elk and hamsters infected with prions, and therefore are strong candidate biomarkers for prion diseases. These results may lay the groundwork towards utilizing altered miRNA signatures as the basis for improved prion diagnostic assays.

## 136. Identification of novel microRNA candidates mediating alpha-synuclein uptake in cells

Elena De Cecco^a^, Remo Sanges^b^, Luca Braga^c^, Paola Zago^a^, Joanna Narkiewicz^a^, Fabio Perissinotto^d^, Irene Guadagnino^e^, Sandro Banfi^e^, Mauro Giacca^c^ and Giuseppe Legname^a,d^

^a^Laboratory of Prion Biology, Department of Neuroscience, Scuola Internazionale Superiore di Studi Avanzati (SISSA), Trieste, Italy; ^b^Computational Genomics Laboratory, Department of Neuroscience, Scuola Internazionale Superiore di Studi Avanzati (SISSA), Trieste, Italy; ^c^ICGEB International Centre for Genetic Engineering and Biotechnology, Padriciano, Trieste, Italy; ^d^ELETTRA Sincrotrone Trieste S.C.p.A, Basovizza, Trieste, Italy; ^e^TIGEM Telethon Institute of Genetics and Medicine, Pozzuoli, Napoli, Italy.

**CONTACT** Elena De Cecco edececco@sissa.it

**ABSTRACT**

**Background**: Alpha-synucleinopathies are a group of fatal neurodegenerative disorders for which neither effective therapies nor preclinical biomarkers are yet available. Indeed, one major characteristic of these syndromes is that the first clinical symptoms start to appear only when more than 70% of dopaminergic neurons in the *substantia nigra* and striatum have been lost, and brain functions are irreversibly compromised. Hence, the identification of early-stage biomarkers poses as a crucial goal in the fight against the progression of neurodegeneration, as well as for the development of more effective and specific drugs. Dysregulation of the expression of non-coding RNAs (ncRNAs) is a common feature among several neurodegenerative diseases. Micro RNAs (miRNAs) are the most studied among ncRNAs and have been shown to participate in Multiple System Atrophy and Parkinson’s Disease pathogenesis at various levels. This work aims at identifying miRNAs that play a role in the internalization of extracellular alpha-synuclein aggregates, which is a key step in the propagation of synucleinopathies.

**Materials and methods**: Our analysis focuses on the internalization of short fibrillar species of alpha-synuclein, as recent findings suggest that short fibres are easily internalized by cells and mediate the spreading of the pathology. To this aim, we set up a comprehensive miRNA screening approach that takes advantage of high throughput screening technology and allows us to screen thousands of miRNAs at the same time. The evaluation of the effect of each miRNA is based on the differential fluorescent labelling of internalized and non-internalized aggregates.

**Results**: Starting from a library of 2042 miRNAs, we found several candidates, which, upon overexpression, either promote or inhibit the internalization of alpha-synuclein short fibrils. Top hits from each phenotype were subjected to a detailed bioinformatics analysis resulting in the identification of multiple possible target genes, many of which are common targets of more top hit miRNAs. We are currently validating the role of some of the selected target genes in order to unravel the contribution of new molecular pathways to the complex phenomenon of aggregates propagation.

**Conclusions**: Our approach led to the identification of a number of miRNAs involved in alpha-synuclein short fibrils uptake, as well as of some target genes. These findings represent a significant advancement to better understand the complex miRNA regulation in diseased brains, and will eventually provide a starting point to design miRNA-based therapies against synucleinopathies.

## 137. Stem cells treatment ameliorate clinical signs and increase incubation periods in prion infected rodents

Paulina Soto, Enrique Antonio Armijo Fuentes, Carlos Kramm, Claudio Soto and Rodrigo Morales

Department of Neurology, The University of Texas Health Science Center at Houston, Houston, TX, USA.

**CONTACT** Paulina Soto paulina.i.sotosoto@uth.tmc.edu

**ABSTRACT**

Prion diseases or transmissible spongiform encephalopathies are a family of rare progressive neurodegenerative disorders that affect both humans and animals. Currently, these disorders have no cure. In the last decades, great efforts have been made to establish effective disease-modifying options to treat them. In recent years, the potential use of induce pluripotent stem cells (iPS) derived neuroprecursors (NPs) has been suggested as a promising cell-replacement therapy for neurodegenerative disorders. iPS-derived NPs have been shown to be effective in animal models of several neurodegenerative diseases including Parkinson’s and Huntington’s diseases, multiple sclerosis, and amyotrophic lateral sclerosis. We tested the therapeutic potential of NPs on prion infected rodents using different means of delivery. Briefly, C57BL/6J female mice were intra-cranially inoculated with RML prions and the potential benefits of NPs was evaluated in three different formats: (1) intra-cerebral administration when the animals showed the first prion-associated clinical signs, (2) intra-venous treatment before the onset of symptoms, and (3) i.v infusions on early symptomatic subjects. Our results show that in all cases NPs significantly extended the clinical phase and incubation periods of prions. Interestingly, peripheral administration of NPs-derived supernatants ameliorated the clinical signs associated to prion disease; however, did not have effect on the incubation periods. Our findings suggest that intracerebral andperipheral administration of iPS-derived NPs might be potentially used to treat prion diseases.

## 138. Human cerebral organoids propagate sporadic CJD prions

Bradley R. Groveman^a^, Simote Foliaki^a^, Christina D. Orru^a^, Gianluigi Zanusso^b^, James A. Carroll^a^, Brent Race^a^ and Cathryn L. Haigh^a^

^a^Laboratory of Persistent Viral Diseases, National Institute of Allergy and Infectious Diseases, Division of Intramural Research, Rocky Mountain Laboratories, National Institutes of Health, 903 South 4th Street, Hamilton, MT 59,840; ^b^Department of Neurosciences, Biomedicine and Movement Sciences, University of Verona, Verona, Italy

**CONTACT** Cathryn L. Haigh cathryn.haigh@nih.gov

**ABSTRACT**

**Introduction**: Human prion disease has traditionally been difficult to model in cell culture. Very few human cell lines show sustained prion propagation over time and none represent three-dimensional (3D) brain tissue. Recently it was found that astrocyte cultures differentiated from human induced pluripotent stem cells (Hu-iPSCs) take up and propagate sporadic CJD (sCJD) prions with the resulting infection influenced by the sCJD subtype [1]. Hu-iPSCs have also been utilized to differentiate self-patterning, structured human brain tissues called cerebral organoids [2,3]. We hypothesized that these cerebral organoids could be used to model human prion propagation *in vitro*.

**Materials and Methods**: Cerebral organoid cultures were generated from hu-iPSCs using the protocol developed by Lancaster and Knoblich [2]. Cerebral organoids were infected with one of two sCJD inocula and monitored for health, morphology, PrP seeding activity, PrP deposition and proteinase-k resistant PrP as compared with organoids that received a normal brain homogenate inoculum.

**Results**: Cerebral organoids showed uptake and propagation of prions, detected by RT-QuIC analysis, following exposure to either sCJD inocula. Inoculum specific differences were observed. One inoculum showed robust proteinase-K resistant PrP in all tested organoids and coarse, granular PrP staining in the organoid interiors. The other inoculum induced a more toxic phenotype that affected organoid health but displayed a low level of prion seeding activity with no detectible protease-resistant PrP.

**Conclusions**: Cerebral organoid cultures can be used to model human prion disease *in vitro*. These structured 3D mini-tissues offer a capacity to investigate aspects of prion diseases never previously available in a human neuronal cell model. Potential human disease-specific applications include delineating sCJD subtype pathogenesis and testing putative therapeutics.

**KEYWORDS:** Cerebral organoid; sporadic CJD; induced pluripotent stem cell; RT-QuIC

### 

References[1]KrejciovaZ, AlibhaiJ, ZhaoC, et al.
Human stem cell-derived astrocytes replicate human prions in a PRNP genotype-dependent manner. J Exp Med. 2017;214(12):3481–3495.2914186910.1084/jem.20161547PMC5716027[2]LancasterMA and KnoblichJA.
Generation of cerebral organoids from human pluripotent stem cells. Nat Protoc. 2014;9(10):2329–2340.2518863410.1038/nprot.2014.158PMC4160653[3]RennerM, LancasterMA, BianS, et al.
Self-organized developmental patterning and differentiation in cerebral organoids. Embo J. 2017;36(10):1316–13292828358210.15252/embj.201694700PMC5430225

## 139. Cryo-electron microscopy of chronic wasting disease prions

Sara Amidian^a,b^, Razieh Kamali-Jamil^a,b^, Ester Vázquez Fernández^a,b^, Maria Carmen Garza^a,b,$^, Camilo Duque Velásquez^a,c^, Xiongyao Wang^a,b,#^, Brian Tancowny^a,b^, Chiye Kim^a^, Judd Aiken^a,d^, Debbie McKenzie^a,c^, Howard Young^b^ and Holger Wille^a,b^

^a^Centre for Prions and Protein Folding Diseases, University of Alberta, Edmonton, Canada; ^b^Department of Biochemistry, University of Alberta, Edmonton, Canada; ^c^Department of Biological Sciences, University of Alberta, Edmonton, Canada; ^d^Department of Animal Health and Biomedical Sciences, University of Alberta, Edmonton, Canada

**CONTACT** Sara Amidian samidian@ualberta.ca

Present address: ^$^University of Zaragoza, Zaragoza, Aragón, Spain

Present address: ^#^School of Materials Science and Engineering, Harbin Institute of Technology, Weihai, Shandong, China.

**ABSTRACT**

**Introduction**: Chronic wasting disease (CWD) is a fatal neurodegenerative disease in cervids that belongs to a group of diseases known as prion diseases. CWD has been detected in Canada, United States, South Korea, Norway, and most recently in Finland. CWD is the most contagious prion disease and poses a risk for transmission to other species including humans.

**Aim**: PrP^Sc^ is the misfolded form of the cellular prion protein (PrP^C^) and the infectious agent that causes prion diseases. The molecular mechanism of PrP^C^ to PrP^Sc^ transformation is unknown. To understand this structural conversion, it is essential to understand the structure of PrP^Sc^. Thus, the goal of this project is to investigate the structure of PrP^Sc^ forms that are causing CWD, using negative stain and cryo electron microscopy (EM).

**Method**: PrP^Sc^ amyloid fibrils were purified from the brains of CWD-infected Tg33 mice, which express deer prion protein. To purify PrP^Sc^ amyloid fibrils, phosphotungstate anions and sarkosyl were used. In addition, enzymes such as proteinase K (PK) or pronase E (PE) were added to remove PrP^C^. To assess the quality and quantity of the PrP^Sc^ fibrils, the purified amyloid fibrils were negatively stained and visualized using EM. Cryo-EM and 3D fibril reconstruction are then performed on fibril preparations.

**Results and Conclusion**: PrP^Sc^ amyloid fibrils were successfully purified and visualized using negative stain EM. Both, PK- and PE-purified samples displayed complex yet similar morphologies. However, a dominant and recognizable type seen in both preparations consisted of filaments with a clear twisted ribbon-like morphology. 2D class averages and 3D reconstructions from the negatively stained micrographs could help to better classify these fibrils. Additionally, cryo-EM analyses could resolve the structural differences between conformers of distinct CWD prion strains and their fibrillization properties. PE-purified fibrils, on the other hand, exhibited striations that run perpendicular to the fibril axis. Interestingly, the distance between these striations is ~40 nm, which corresponds to the molecular height of two PrP^Sc^ molecules in a four-rung beta solenoid architecture that may adopt a head-to-head arrangement [1]. This observation could be explained by the presence of sarkosyl molecules associating with the hydrophobic GPI-anchor of the stacked PrP^Sc^ molecules. Further image processing analyses will provide a more complete understanding of the orientation of the GPI-anchor in the CWD fibrils.

### 

References[1]WilleH, RequenaJ.
The Structure of PrP^Sc^ Prions. Pathogens. 2018;7:20 DOI:10.3390/pathogens7010020.PMC587474629414853

## 140. Molecular dynamics simulations of cervid prion protein variants to assess protein stability and susceptibility towards chronic wasting disease

Sara Amidian^a,b^, Lyudmyla Dorosh^c,d^, Camilo Duque Velásquez^a,e^, Maria Stepanova^c,d^, Judd Aiken^a,f^, Debbie McKenzie^a,e^ and Holger Wille^a,b^

^a^Centre for Prions and Protein Folding Diseases, University of Alberta, Edmonton, Canada; ^b^Department of Biochemistry, University of Alberta, Edmonton, Canada; ^c^National Institute for Nanotechnology, University of Alberta, Edmonton, Canada; ^d^Department of Electrical and Computer Engineering, University of Alberta, Edmonton, Canada; ^e^Department of Biological Sciences, University of Alberta, Edmonton, Canada; ^f^Department of Animal Health and Biomedical Sciences, University of Alberta, Edmonton, Canada.

**CONTACT** Sara Amidian samidian@ualberta.ca

**ABSTRACT**

**Introduction**: Chronic wasting disease (CWD) is a prion disease that affects cervids. The central event in prion diseases is the structural conversion of the healthy, cellular prion protein (PrPC) to the disease- causing isoform, PrPSc. It is technically difficult to experimentally study the initial steps of the PrPC to PrPSc conversion; however, molecular dynamics (MD) simulation plays an important role in probing the misfolding events. One approach is to assess the effect of single-residue substitution on the structural stability of PrPC. Polymorphisms in the prion protein gene can influence prion disease susceptibility, disease progression, clinical presentation, and the propagation of distinct CWD strains associated with different PrPSc conformations. In this project, we use molecular dynamics simulation as a tool to characterize the effect of white-tailed deer (WTD) prion protein polymorphisms Q95H and G96S on the experimentally determined structure of the natively folded prion protein.

**Methods**: The initial model for the WTD prion protein (residues 93–233) was generated using the crystal structure of recombinant deer prion protein (PDB: 4YXH) as the template. The wild type (WT), 95H, and 96S models were subjected to minimizations, equilibrations, and production MD simulations using the Gromacs package. In total, three independent 50ns simulations were performed for each system.

**Results**: The 96S polymorphism displayed a higher root mean square deviation (RMSD) and root mean square fluctuation (RMSF) values, which are indicators of lower structural stability compared to WT and 95H forms. Also, 96S had a larger radius of gyration (Rg) values, indicating that the structure of 96S was less compactly folded than WT and 95H. Calculating the solvent accessible surface area (SASA) of 96S revealed that the structure of 96S was more exposed to the solvent. On the other hand, structural dynamics of the 95H polymorphism were closer to WT. RMSF and Rg values for 95H indicated higher stability and compactness of its structure. Additionally, the SASA values of 95H were very similar to WT.

**Conclusions**: Experimentally, the 96S allele is associated with slower disease progression and animals carrying this polymorphism have longer incubation time when inoculated with CWD prions. According to our MD results, it is apparent that the structure of WTD prion protein carrying the 96S polymorphism is less stable than the WT protein. The clear difference in the structural stability and local dynamics of 96S compared to WT, may be relevant to its partial resistance to prion disease, however, further analyses are required.

## 141. Propagation of human sCJD prions in organotypic slice culture

Grant Norman, Hailey Pineau and Valerie L. Sim

Department of Medicine, Division of Neurology, Faculty of Medicine & Dentistry, University of Alberta; Centre for Prions and Protein Folding Diseases, University of Alberta

**CONTACT** Valerie L. Sim valerie.sim@ualberta.ca

**ABSTRACT**

Prion diseases, or transmissible spongiform encephalopathies (TSEs), are neurodegenerative diseases that are invariably fatal. These diseases result from the conversion of the normal prion protein (PrPC) to a misfolded form (PrPSc) that can template itself and spread through the brain, triggering neurodegeneration. TSEs can affect many mammals, including cervids (Chronic Wasting Disease; CWD), sheep (scrapie), cattle (Bovine Spongiform Encephalopathy; BSE), and humans (Creutzfeldt-Jakob Disease; CJD). There are no treatments for any of the prion diseases, despite decades of research. One challenge to finding successful therapies is the fact that PrPSc can exist in a number of different conformations, each of which may have its own biophysical properties and cause distinct neuropathologies, giving rise to unique prion strain phenotypes. Treatments that target only one specific conformational change in may only work for one strain. Ideally, therapy studies for humans should be done with human prion strains. Here we demonstrate the propagation of human prions in prion organotypic slice culture with evidence of associated neuronal loss. Traditionally, prion strains are generated in animals infected with prion disease; however, these are lengthy experiments, often taking several months. By contrast, the prion organotypic slice culture assay (POSCA) can faithfully recapitulate aspects of prion pathology on a shortened timescale of 40–50 days [1,2]. This makes POSCA an ideal method for propagating prion strains. Originally, POSCA was developed to test mouse-adapted scrapie strains in cerebellar slices [2,3]. We have adapted POSCA to sporadic CJD strains from human samples and samples passaged through mice expressing human PrP. We have also adapted the system to whole brain coronal slices. The ability to propagate human prion strains *ex vivo* will allow investigation of human strain-specific properties during pathogenesis as well as development of strain-specific therapeutic interventions. Because POSCA is an open system, it also allows for testing treatments at discrete disease time points without the confounder of blood-brain barrier.

### 

References[1]FalsigJ, et al. ProtocNat
2008;3(4):555–62.10.1038/nprot.2008.1318388937[2]FalsigJ, et al. PathogPLoS
2012;8(11):e1002985.10.1371/journal.ppat.1002985PMC348691223133383[3]CampeauJ, et al. OnePLoS
2013;8(12):e81776.10.1371/journal.pone.0081776PMC384708824312586

## 142. Effects of Elk-PrP^C^ expression levels on CWD strain properties

Camilo Duque Velásquez^a^, Alicia Otero^a^, Chiye Kim^a^, Elizabeth Triscott^a^, Jeffrey Narayan^a^, Jacques Van der Merwe^b^, Judd Aiken^c^ and Debbie McKenzie^a^

^a^Department of Biological Sciences, Centre for Prions and Protein Folding Diseases, University of Alberta, Edmonton, Canada; ^b^Department of Medicine, Centre for Prions and Protein Folding Diseases, University of Alberta, Edmonton, *AB*, Canada; ^c^Department of Agricultural, Food and Nutritional Sciences, Centre for Prions and Protein Folding Diseases, University of Alberta, Edmonton, *AB*, Canada

**CONTACT** Alicia Otero oterogar@ualberta.ca

**ABSTRACT**

Chronic Wasting Disease (CWD) is a contagious prion disease affecting various species of free-ranging and/or captive cervids on three continents. Species-specific prion protein (PrP^C^) polymorphisms influence prion conversion into PrP^CWD^. PrP^C^ amino acid variation can also regulate disease susceptibility to particular prion strains and has been implicated in the diversification of prion strain conformers [1, 2, 3]. Elk and deer PrP^C^ differ at residue E226Q and this amino acid difference has been implicated in the selection of CWD1 and CWD2 prion strains [4]. As PrP^C^ expression has been suggested to affect prion strain evolution [5], we hypothesized that elk PrP^C^ levels affect CWD strain generation. To test this hypothesis, transgenic (tg) FVB mice over-expressing elk PrP^C^ [6] were crossed with *prnp* knock-out FVB mice to generate tg-elk with different PrP^C^ expression levels. Both tg-elk^+/+^ and tg-elk^±^ were exposed to white-tailed deer CWD strains (Wisc-1 and H95^+^) [2, 3]. The H95^+^ strain was a mixture generated on passage of Wisc-1 in deer heterozygous for H95G96 and Q95S96 [2]. Tg-elk^+/+^ mice succumbed to Wisc-1 with a mean incubation period of 116 ± 7 days post infection (dpi) compared to 164 ± 11 dpi for the H95^+^ strain mixture. Consistent with the reduced PrP^C^ expression, the same deer prion strains resulted in longer incubation periods (157 ± 21 dpi and >180 dpi, respectively) when passaged in tg-elk^±^ mice. After first passage, transmission of Wisc-1 and H95^+^ in tg-elk^+/+^ mice resulted in a single neuropathological profile that differed from the profile produced by passage of elk prions (described as the CWD2 strain [1]). Our results show that, upon first passage, the E226Q polymorphism did not affect the strain properties of deer prions and indicates a single strain (Wisc-1) was selected by the tg-elk^+/+^ mice. The comparative analysis of neuropathological profiles between high and low expression tg-elk on first and second passage will be presented.

### 

References[1]JohnsonCJ, HerbstA, Duque-VelasquezC, et al.
Prion protein polymorphisms affect chronic wasting disease progression. PLoS ONE. 2011;6(3):e17450.2144525610.1371/journal.pone.0017450PMC3060816[2]Duque VelasquezC, KimC, HerbstA, et al.
Deer prion proteins modulate the emergence and adaptation of chronic wasting disease strains. J Virol. 2015;89(24):12,362–12,37310.1128/JVI.02010-15PMC466524326423950[3]HerbstA, et al.
Chronic wasting disease prion strain emergence and host range expansion. Emerg Infect Dis. 2017;23(9):1598–1600.2882038410.3201/eid2309.161474PMC5572867[4]AngersR, et al.
Prion strain mutation determined by prion protein conformational compatibility and primary structure
Science. 2010;328, 5982, 1154–1158.10.1126/science.1187107PMC409767220466881[5]DurLe, et al.
Divergent prion strain evolution driven by PrPC expression level in transgenic mice. Nat Commun. 2017;8:14,170.2811216410.1038/ncomms14170PMC5264111[6]La Fauci et al. Passage of chronic wasting disease prion into transgenic mice expressing Rocky Mountain elk
(*Cervus elaphus nelsoni*) PrP^C^. J Gen Virol. 2006;87: 3773–3780.1709899710.1099/vir.0.82137-0

## 143. A role for molecular chaperones in the rate of Alzheimer’s disease

Sarah C. Hales^a^, Hailey Pineau^a,b^, Grant Norman^a,b^ and Valerie L. Sim^a,b^

^a^Centre for Prions and Protein Folding Diseases, University of Alberta; ^b^Department of Medicine, Division of Neurology, Faculty of Medicine & Dentistry, University of Alberta

**CONTACT** Sarah C. Hales shales@ualberta.ca

**ABSTRACT**

Despite similar pathological findings upon post-mortem examination of Alzheimer Disease (AD) brains (i.e. β-amyloid plaques and neurofibrillary tangles), the rate at which patients progress through this disease is highly variable, ranging from less than 2 years in very rare cases, to an average of 10 years. Previously, studies seeking to understand heterogeneity in disease progression have primarily looked for differences in the hallmark proteins associated with AD: amyloid-β (Aβ) and tau. In terms of Aβ, studies have found differences in Aβ plaque composition1 and peptide structure2 in more rapidly progressing forms of the disease. Furthermore, the presence of different tau ‘strains’ has been suggested to influence its ability to aggregate and spread.3 However, the structural changes in Aβ and tau have not shed light on why disease duration varies so dramatically between patients. Our research into fast and slow cases of AD has revealed an increase in Aβ42 in the more slowly progressing cohort of patients, a finding that was unexpected as Aβ42 is often thought to be the more toxic species of Aβ. This may indicate that other factors present in the cellular environment may drive the rate of disease over and above any differences in Aβ and tau. Given the established role for molecular chaperones in the regulation of proteostasis and mediation of protein aggregation, they are ideal candidates to investigate with regards to rate of AD progression.

To test the role of chaperones in rate of disease, we are measuring levels of DNAJA2, HSP70, and HSC70 in human brains from patients with fast versus slow AD. We are also manipulating the levels of chaperones to determine their effect on Aβ and tau aggregation in cell culture model systems (N2A cells containing the Swedish amyloid precursor protein mutation, HEK293 cells containing the tau P301L mutation, and HEK293 cells containing the repeat domain of tau). A summary of these interactions and correlations will be presented.

### 

References[1]DrummondE, et al.
Acta Neuropathol
2017; 133:933–954.2825839810.1007/s00401-017-1691-0PMC5503748[2]CohenML et al. Brain
2015; 138:1009–1022.2568808110.1093/brain/awv006PMC5014074[3]KaufmanSK, et al.
Neuron
2016; 92:796–812.2797416210.1016/j.neuron.2016.09.055PMC5392364

## 144. Deer prion protein polymorphisms influence *in vitro* aggregation kinetics

Helen Y. Yao^a^, Leonardo M. Cortez^a,b^, José Miguel Flores-Fernández^a,c^, Holger Wille^a,c^, Debbie McKenzie^a,d^ and Valerie L. Sim^a,b^

^a^Centre for Prions and Protein Folding Diseases, University of Alberta; ^b^Department of Medicine, Division of Neurology, Faculty of Medicine & Dentistry, University of Alberta; ^c^Department of Biochemistry, Faculty of Medicine & Dentistry, University of Alberta; ^d^Department of Biological Sciences, Faculty of Science, University of Alberta

**CONTACT** Helen Y. Yao hengyue@ualberta.ca

**ABSTRACT**

**Introduction**: Chronic wasting disease (CWD) is a transmissible neurodegenerative disease of cervids. It is caused by the misfolding and self-templated aggregation of the prion protein (PrP). Different polymorphisms of PrP are associated with different *in vivo* disease susceptibility, as shown in mice, elk and white-tailed deer [1]. Polymorphisms such as 95H and 96S in deer Prnp are associated with altered transmission properties, but the mechanism by which this happens is not clear. Deer heterozygous for the with G96S and/or Q95H polymorphisms have longer incubation periods than deer expressing wt Prnp [1]. Mice expressing 96S deer PrP are not susceptible to Wisc-1 strain CWD. Interestingly, PrP aggregates containing 95H are able to infect mice expressing 96S [2]. If these transmission differences are caused by different aggregation and seeding efficiencies of the three polymorphic PrP types, we may find similar correlations with *in vitro* aggregation studies: PrP containing 96S or 95H should be less effective as a substrate for conversion, unless the seed contains 95H-PrP. Similarly, we expect any aggregate with 95H to be more efficient than 96S as a seed.

**Methods**: Wild type, 96S and 95H deer PrP were expressed in *E. coli* and purified from inclusion bodies by affinity chromatography followed by HPLC. Monomeric PrP was aggregated using real-time quaking induced conversion (RT-QuIC) to study the kinetics of spontaneous, homologously seeded and heterologously seeded aggregation. Lag phases and final ThT fluorescence values were compared.

**Results**: As predicted, 96S was the most inefficient substrate for conversion, even when homologously seeded. Spontaneously formed aggregates of 96S were also the least efficient seeds. 95H, on the other hand, produced aggregates that were very efficient at seeding, even more than wildtype aggregates. 95H was also an excellent substrate for conversion, being more efficient than wildtype.

**Conclusions**: The inefficient aggregation and seeding ability of 96S PrP correlates with its long incubation period *in vivo*, suggesting this amino acid change could explain the *in vivo* findings. Similarly, the high seeding ability of 95H PrP aggregates correlates with the finding that 95H strains can infect otherwise resistance mice (96S mice). However, 95H was a very efficient substrate *in vitro* which is not what has been observed in deer heterozygous for 95H; whether deer homozygous for 95H would have faster incubation periods remains to be seen.

### 

References[1]JohnsonC, et al.
J Gen Virol
2006;87:2109–114.1676041510.1099/vir.0.81615-0[2]Duque VelasquezC, et al.
J Virol
2015;89:12,362–73.

## 145. Deciphering ovine scrapie complexity in Europe by using bioassay in two rodent models

Alba Marín-Moreno^a^, Patricia Aguilar-Calvo^a^, José Luis Pitarch^a^, Juan Carlos Espinosa^a^, Olivier Andreoletti^b^, Romolo Nonno^c^, Jan Langeveld^d^ and Juan María Torres^a^

^a^Centro de Investigación en Sanidad Animal (CISA-INIA), Madrid, Spain; ^b^École Nationale Vétérinaire de Toulouse, Toulouse, France; ^c^Istituto Superiore di Sanitá, Rome, Italy; ^d^Wageningen BioVeterinary Research, Lelystad, the Netherlands.

**CONTACT** Alba Marín-Moreno marin.alba@inia.es

**ABSTRACT**

Within the prion field, scrapie tends to be named as a uniform disease caused by a single prion strain. However, as the opposite that applies for Bovine Spongiform Encephalopathy, several scrapie strains producing different prion diseases phenotypes have been reported in the literature. Previous work using European goat prion isolates already pointed to this situation (Nonno et al.). Goat and sheep scrapie isolates proceeding from several European countries were intracranially inoculated into transgenic mice overexpressing ovine and bovine PrP proteins (Ov-Tg501 and Bo-Tg110 respectively). Two iterative passages were performed. The combination of the two different rodent models allows the classification of the isolates in four groups that may correspond to several scrapie strains. Abrogation of the transmission barrier by performing the second passage is necessary to correctly classify the isolates. In addition, Bo-Tg110 provides the most important information to allow discrimination between groups.

**KEYWORDS:** Scrapie; prion strain; transmission barrier; Europe; mouse bioassay.

**Funding**

Spanish Ministerio de Ciencia, Innovación y Universidades (AGL2016-78054R) and European Union projects CT-2006–36,353 and 219,235 ERA-NET FP7 EMIDA.

## 146. Conversion kinetics of sporadic CJD prions using multiple substrates and RT-QuIC-based detection and discrimination

Stephanie Booth^a^, Sarah Medina^b^, Lise Lamoureux^b^ and Debra Sorensen^c^

^a^PHAC; ^b^Public Health Agency of Canada; ^c^Public Health Agency of Canada-NML

**ABSTRACT**

Prions often maintain their conformational characteristics upon natural transmission to a new species and that this is dependent on the structural characteristics of both the ‘infecting’ PrP and the new host PrP. Based on the hypothesis that variations in prion conformation are intrinsic to strain characteristics and ultimately disease phenotypes, it seems logical to conclude that the seed – substrate pairing has a direct relationship to the conversion rate in assays such as real-time quaking-induced conversion (RT-QuIC). We have used RT-QuIC to assess the relative rates of conversion of CJD prions from over 40 patients for whom CJD was confirmed as the cause of death by the Canadian CJD Surveillance System. These patients included those diagnosed with five subtypes of sporadic CJD, sCJDMM1/sCJDMV1, sCJDVV2, sCJDMV2, SCJDMM2 and sCJDVV1. A number of different recombinant prion proteins including hamster, elk and human were used as substrate. Serial dilution of brain tissue from these individuals were used as seeds. We hypothesized that the degree of complementarity during seed-substrate pairing would provide a measure of strain conformation based on the relative efficiency of the initial amyloid conversion of a particular substrate by a seed. We measured the conversion rates by comparing the time at which fluorescence in each reaction exceeded a pre-defined threshold, i.e. the lag phase. Lag phases were extremely consistent for each CJD type in the different substrates so that we could statistically compare cases and assign to different types. Of interest, we found a small number of atypical cases whose conversion kinetics differed significantly from others within their subtype perhaps indicating different strain types. We also performed seeding studies on sCJD prions isolated from specific brain regions of interest providing information on the heterogeneity of prions related to the brain region in which they are propagated.

## 147. Novel, pan-PrP^Sc^-specific antibodies reveal a shared β-solenoid structure for all PrP^Sc^ strains tested

Holger Wille^a,b^, Andrew Fang^a,b^, Xinli Tang^a,b^, Madeleine Fleming^a,b^, Tolu Abodunwa^a,b^, Leonardo M. Cortez^a,c^, Claudia Y. Acevedo-Morantes^a,b^, Camilo Duque Velásquez^a,d^, Brian Tancowny^a,b^, Xiongyao Wang^a,b,$^, Judd Aiken^a,e^, Debbie McKenzie^a,d^ and Valerie L. Sim^a,c^

^a^Centre for Prions and Protein Folding Diseases, University of Alberta; ^b^Department of Biochemistry; ^c^Department of Medicine; ^d^Department of Biological Sciences; ^e^Department of Agricultural, Food, and Nutritional Science, Edmonton, Alberta, Canada.

**CONTACT** Holger Wille wille@ualberta.ca

^$^current address: School of Materials Science and Engineering, Harbin Institute of Technology, Weihai, Shandong, China.

**ABSTRACT**

**Background**: The high-resolution structure of PrP^Sc^ has eluded experimental determination due to its insolubility and its general propensity to aggregate. Nevertheless, the repeating nature of PrP^Sc^ amyloid fibrils has allowed the collection of lower-resolution structural data via X-ray fibre diffraction, cryo-electron microscopy, and other techniques. All of these approaches indicated that the structure of PrP^Sc^ is based on a four-rung, parallel β-solenoid architecture.

**Materials and Methods**: The aforementioned β-solenoid architecture allowed us to predict which amino acid side chains may be oriented towards the surface of PrP^Sc^ versus those that form the interior of the β-solenoid fold. Based on these predictions, we created a structural and immunological mimic for PrP^Sc^ by inserting outwards facing residues into an identical position in an innocuous protein scaffold based on the fungal prion HET-s. This structural and immunological mimic (=vaccine candidate) was then injected into wild-type mice and their immune response analysed. The resultant splenocytes were used to generate hybridoma cells for the production of monoclonal antibodies. These monoclonal antibodies were characterized for their ability to recognize native PrP^Sc^. Lastly, a systematic series of revertant mutants of the PrP^Sc^-based vaccine candidate towards the unmodified HET-s scaffold were used to further refine the epitopes that these antibodies recognize.

**Results**: Immunization with the structure-based vaccine candidate resulted in a PrP^Sc^-specific immune response in 8 out of 8 mice. The post-immune antiserum recognized only native PrP^Sc^, and did not bind to PrP^C^, recombinant PrP, denatured PrP, or any linear PrP peptides. The monoclonal antibodies that were derived from the immunized mice (IgGs and IgMs) recognized all natural PrP^Sc^ strains/isolates, including BSE, CWD, TME, scrapie, and human prions (familial: fCJD, FFI, GSS; sporadic: sCJD; infectious: vCJD). Moreover, the monoclonal antibodies did not recognize *in vitro* generated recPrP amyloid, which presumably adopted a parallel in-register intermolecular β-sheet (PIRIBS) architecture. Lastly, the HET-s revertant mutants helped to define the PrP^Sc^ epitopes within the framework of the HET-s β-solenoid structure.

**Conclusions**: Our novel, pan-PrP^Sc^-specific antibodies confirm the hypothesis that the structure of PrP^Sc^ contains a parallel β-solenoid fold. In particular, these antibodies recognize structural epitopes that are specific for the parallel β-solenoid fold of PrP^Sc^, but do not recognize alternative conformers of PrP including β-sheet rich PIRIBS structures. These antibodies and the original antigen are currently being studied for their potential use as passive or active immunotherapy agents and for use in diagnostic applications.

## 148. Characterization of infectious human prions

Claudia Y. Acevedo-Morantes^a,b^, Xiongyao Wang^a,b,*^, Brian Tancowny^a,b^, Susana Teijeira^c,d^, Beatriz San Millán^c,d^ and Holger Wille^a,b^

^a^Centre for Prions and Protein Folding Diseases; ^b^Department of Biochemistry, University of Alberta, Edmonton, *AB*, Canada; ^c^Galicia Sur Health Research Institute, IIS Galicia Sur, SERGAS-UVIGO, Vigo, Spain; ^d^Pathology Department, Complexo Hospitalario Universitario de Vigo (CHUVI), IIS Galicia Sur, SERGAS, Vigo, Spain.

**CONTACT** Claudia Y. Acevedo-Morantes cacevedo@ualberta.ca

*Current address: School of Materials Science and Engineering, Harbin Institute of Technology, Weihai, Shandong, China.

**ABSTRACT**

Human prion diseases are a family of rare and progressive neurodegenerative disorders. They present as sporadic, familial, infectious, or iatrogenic forms, and include Creutzfeldt-Jakob disease (CJD), Gerstmann-Sträussler-Scheinker syndrome (GSS), and Fatal Familial Insomnia (FFI). The causes for the wide range of phenotypic variation in the human prion diseases are unknown, but the disease is caused by aberrantly folded versions of the prion protein, termed PrPSc. Biochemical properties such as partial resistance to digestion by proteinase K, insolubility in non-ionic detergents, and sedimentation properties have provided evidence that distinct forms of PrPSc aggregates with heterogeneous morphologies regarding fibril length, width, shape, longitudinal twist, and the number of protofilaments that may characterize these disease entities. In this study, we are using several biochemical and biophysical approaches to analyse and compare PrPSc-fractions derived from the brains of patients affected by sporadic CJD (sCJD), familial CJD (fCJD), variant CJD (vCJD), GSS, and FFI. It is hypothesized that the structure of PrPSc is the key factor that determines its infectious nature, transmission route, and pathogenicity. However, the insolubility of PrPSc and its overall propensity to aggregate make it inaccessible to high-resolution structure analyses. Low-resolution approaches have provided information on the architecture of PrPSc to devise molecular models. Using tilted-beam transmission electron microscopy (TB-TEM), we are measuring the mass-per-length (MPL) value of individual PrPSc fibrils derived from the brains of patients affected by the aforementioned prion diseases. These results will provide a platform to evaluate the proposed models for the structure of PrPSc fibrils. Tobacco Mosaic Virus (TMV), with its well-determined structure and an MPL value of 131 kDa/nm, serves as an internal calibration standard for the MPL measurements. Preliminary results obtained with purified PrPSc fibrils from sCJD samples indicated a population of fibrils with a MPL value around 60 kDa/nm, which would support the four-rung β-solenoid model based on two protofilaments per fibril. Additional MPL measurements on GSS patient-derived samples are underway. Recombinant PrP amyloid fibrils as well as *in vitro* converted recombinant PrPSc will be used to round out the MPL comparison of prion fibril structures. MPL data impose a stringent constraint on viable fibril models when the subunit mass is known. By calibrating the image intensity of PrPSc to the internal standard, we will be able to limit the number of possible indexing schemes significantly, and provide independent experimental validation to the current proposed models.

## 149. Rationally designed, structure-based vaccines candidates for aβ, tau and α-synuclein

José M Flores-Fernández, Enrique Chimal-Juárez, Xiongyao Wang*, Brian Tancowny, Xinli Tang, Andrew Fang and Holger Wille

Centre for Prions and Protein Folding Diseases & Department of Biochemistry, University of Alberta, Edmonton, Alberta, Canada.

**CONTACT** José M Flores-Fernández floresfe@ualberta.ca

*current address: School of Materials Science and Engineering, Harbin Institute of Technology, Weihai, Shandong, China.

**ABSTRACT**

**Background**: Alzheimer’s and Parkinson’s diseases are mainly caused by the misfolding of Aβ, tau, and α-synuclein proteins, respectively. Each of these proteins adopts a beta-sheet rich conformation and aggregates into amyloid. Different attempts to develop vaccines for these diseases have used the respective proteins and peptides in their linear form and without control of their tertiary and quaternary structure, which caused a lack of specificity and subsequently a failure to achieve protection. In this study, we present a new approach to design vaccine candidates based on structurally defined proteins that result in disease-specific antigenicity.

**Materials and Methods**: The fungal prion Het-s was used as an innocuous scaffold protein because it natively adopts a conformation rich in beta-sheet. This scaffold protein was engineered based on the surface exposed amino acids of Aβ, tau, and α-synuclein aggregates to express antigenic determinants in a structurally controlled manner. The resulting vaccine candidates were produced in *Escherichia coli*, purified, refolded, and then analysed by EM for the proper folding of the proteins. Only those constructs that formed amyloid fibrils with the typical structure of Het-s amyloid were injected into mice. The resulting polyclonal antibodies were tested against unmodified Het-s, the engineered Het-s antigen, and disease-specific antigens in order to determine their specificity.

**Results**: After the purification and refolding of the engineered proteins, several vaccine candidates that targeted Aβ, tau, and α-synuclein showed the expected self-assembly into amyloid fibrils by negative stain electron microscopy. The self-assembled constructs were injected in mice and the collected antisera were tested for their specificity by ELISAs using Het-s and its engineered variants as antigens. The vaccine candidates Aβ4 and Tau4 elicited immune responses with higher reactivity for the engineered antigens than for unmodified Het-s.

**Conclusions**: Rationally designed, structure-based vaccine candidates targeting the misfolded proteins responsible for Alzheimer’s and Parkinson’s disease are feasible with this approach.

## 150. Characterization of the glycosylation profile of water-soluble prion protein from brain and blood of hamster using lectins

Hanin Abdel-Haq

Istituto Superiore di Sanità, Rome, Italy

**CONTACT** Hanin Abdel-Haq Hanin.abdelhaq@iss.it

**ABSTRACT**

**Background**: The disease-associated water-soluble form of hamster prion protein (ws-PrP^Sc^) has recently been found to be less stable than classical PrP^Sc^. Since the stability of PrP to enzymatic degradation correlates with its glycosylation level, the aim of this study was to investigate whether there are differences between the glycosylation of ws-PrP^Sc^ and classical PrP^Sc^ of hamster.

**Materials and methods**: To achieve this aim, ws-PrP and classical PrP were captured from noninfected or scrapie-infected hamster brain homogenate [high-speed supernatant (S^HS^) and high-speed pellet (P^HS^)] and blood plasma by anti-PrP antibodies (3F4 and 6H4) and subjected to screening for glycans by seven biotinylated lectins under denaturing or nondenaturing conditions in a sandwich lectin enzyme-linked immunosorbent assay (lectin-ELISA).

**Results**: Glycans have been found in minor quantities and differently exposed on ws-PrP^Sc^ from S^HS^ and plasma compared with classical PrP^Sc^ from P^HS^. These differences have been shown to be potentially responsible for the instability of ws-PrP^Sc^. Treatment of infected blood with guanidine hydrochloride (GdnHCl) significantly (*P* <0.01) increased the detection of ws-PrP^Sc^ in ELISA, reflecting an increase in its stability, and showed efficacy in removing high-abundance proteins in silver-stained gels. This increase in ws-PrP^Sc^ stability is due to an interaction of GdnHCl not only with high-abundance proteins but also with the ws-PrP^Sc^ glycosylation with particular regard to the mannose sugar. Analysis of lectins immunoreactivity towards total proteins from plasma collected before and at different time points after infection, revealed that mannose might exert a stabilizing effect towards all of hamster blood glycoproteins, regardless of scrapie infection. Since low levels of ws-PrP^Sc^/soluble-infectivity have been estimated both in blood and brain of hamster, this glycosylation-related instability may have negatively influenced the propensity of ws-PrP^C^ to convert to ws-PrP^Sc^ both in blood and the brain.

**Conclusions**: Glycosylation characteristics of cellular prion protein (PrP^C^) may provide a tool for the determination risk of prion transmissibility among different species and tissues. These results could be representative for other misfolded proteins that are characterized by having different glycoforms, including the soluble form, such as amyloid beta in Alzheimer’s disease.

**KEYWORDS:** Glycosylation; Prion protein; Guanidine-induced protein stabilization; Lectins; Blood; Misfolded proteins.

## 151. Early detection of prion protein aggregation with pFTAA using spectral confocal microscopy

Waqas Tahir^a^, Anastasiia Stepanchuk^b^, Hermann M Schaetzl^a^ and Peter K Stys^b^

^a^Department of Comparative Biology and Experimental Medicine, Faculty of Veterinary Medicine, University of Calgary, Calgary, Alberta, Canada; ^b^Department of Clinical Neuroscience, Hotchkiss Brain Institute, University of Calgary, Calgary, Alberta, Canada

**CONTACT** Waqas Tahir waqas.tahir1@ucalgary.ca

**ABSTRACT**

**Background**: Prion diseases are fatal neurodegenerative diseases characterized by the formation of amyloidogenic prion aggregates in the CNS. Prion aggregates are formed by conformational alteration of host encoded cellular prion protein into the scrapie form. Quick progression in disease pathology after the onset of symptoms makes such diseases deadlier. Early detection of prion aggregates can help to better understand the pathophysiology and spread of prion infection in the brain. In this regard, amyloid sensitive fluorescent probes and advanced analytical tools to quantify emission spectra of those fluorescent probes can help to detect these pathological events at earlier stages of disease progression.

**Objective**: Early detection of protein aggregates in prion infected mouse brains by examining changes in emission spectrum of brain sections stained with the amyloid-sensitive fluorescent probe pFTAA by using spectral confocal microscopy.

**Materials and Methods**: FVB mice were intra-cerebrally inoculated with 20 μL of brain homogenates (mock or 22 L infected) from terminal mice. Prion infected and age matched mock inoculated mice were euthanized for pre-symptomatic stages (at 75 and 100 dpi), symptomatic stage (125 dpi) and terminal stage. Brain tissues were extracted and fixed with formalin. Brain sections (5 μm) were hydrated followed by incubation with pFTAA (3 µM) for 45 min. Stained sections were imaged with confocal microscope equipped with a 32-channel spectral detector. Image analysis was done in custom-written software ImageTrak using a non-linear spectral unmixing algorithm.

**Results**: Fluorescence spectral analysis of prion-infected brain sections from pre-symptomatic stages (75 and 100 dpi) showed increased fluorescence intensity as well as a characteristic red shift in the emission spectrum as compared to age matched mock brain sections. Differences in emission spectrum between prion infected and mock inoculated brain sections increased at later stages of the disease. Spectral differences at pre-symptomatic stages were mainly seen in somatosensory cortex, hippocampus and thalamus. Pathological alterations represented by changes in emission spectrum during symptomatic and terminal stages spread to further brain regions including olfactory bulb, hypothalamus, cerebellum, and brainstem.

**Conclusions**: Advanced spectral imaging analysis of pFTAA stained brain sections infected with 22 L prions showed robust differences in emission spectrum during prion infection. This shows the potential of such methods for early detection of prion aggregates, possibly also in the tissues other than only brain, and also can offer better understanding about mechanism of prion spread in protein misfolding disorders with higher sensitivity.

## 152. Tissue tropism of prions is dictated by the balance between PrP^Sc^ formation and degradation

Ronald A. Shikiya^a^, Katie A. Langenfeld^b^, Thomas E. Eckland^c^, Jonathan Trinh^a^, Sara A. M. Holec^a^, Candace K. Mathiason^d,e^, Anthony E. Kincaid^a,e^ and Jason C. Bartz^a^

^a^Department of Medical Microbiology and Immunology, Creighton University, Omaha, NE, USA; ^b^Biology Department, University of Nebraska at Omaha, Omaha, NE, USA; ^c^Department of Neurology, University of Texas Health Science Center, Houston, TX, USA; ^d^Department of Microbiology, Immunology and Pathology, Colorado State University, Fort Collins, CO, USA; ^e^Department of Pharmacy Science, Creighton University, Omaha, NE, USA

**CONTACT** Ronald A. Shikiya ronaldshikiya@creighton.edu

**ABSTRACT**

Prion strains are characterized by strain-specific differences in neuropathology, incubation period, clinical disease, host-range, and tissue tropism. In hamsters, the hyper (HY) and drowsy (DY) prion strains differ in tissue tropism and susceptibility to disease when extraneural routes of infection are used. Notably, hamster susceptibility to the DY strain is restricted when inoculation occurs via extraneural routes. In contrast to the HY strain, DY is not detected in secondary lymphoreticular system (LRS) tissues of infected hosts regardless of the route of infection. Our experiments show that, similar to the lymphotropic strain HY, DY crosses the mucosal epithelia, enters the draining lymphatic vessels in underlying laminae propriae, and is transported to LRS tissues. Since DY is able to cause disease once it enters the peripheral nervous system, the restriction in DY pathogenesis is not due to a failure of transport but may be due to its inability to stablish infection in LRS tissues. To determine if DY can propagate in LRS tissues, we performed protein misfolding cyclic amplification using DY PrP^Sc^ as the seed and spleen homogenate as the source of PrP^C^. Our results show that the spleen environment can support DY PrP^Sc^ formation, albeit at lower levels when compared to other lymphotropic prion strains, suggesting that the restriction in DY pathogenesis is not due to the absence of a strain-specific co-factor in spleen. In addition, DY PrP^Sc^ is more susceptible to degradation compared to PrP^Sc^ from other lymphotropic strains. Based on these results, we suggest that the equilibrium between PrP^Sc^ formation and clearance plays a role in prion tissue tropism.

## 153. Molecular mechanisms of Aβ fibril elongation, secondary nucleation, and fibril dissociation studied *in-silico*

Lyudmyla Dorosh^a,b^ and Maria Stepanova^b,c^

^a^Department of Electrical and Computer Engineering, University of Alberta, Edmonton, Canada; ^b^National Institute for Nanotechnology NRC; Edmonton, Canada; ^c^Department of Electrical and Computer Engineering, University of Alberta, Edmonton, Canada

**CONTACT** Lyudmyla Dorosh dorosh@ualberta.ca; Maria Stepanova ms1@ualberta.ca

**ABSTRACT**

Prevalence in ageing population, difficulties in early diagnosis, and lack of efficient therapies make Alzheimer’s disease (AD) one of the most devastating neurodegenerative disorders of our time. AD is associated with misfolding and formation of toxic aggregates of amyloid β (Aβ) peptide, subsequently producing β-rich amyloid deposits [1]. Evidence has emerged that amyloid fibrils may catalyse the process of corruptive misfolding through a positive-feedback reaction loop [2]. However, detailed molecular mechanisms of this process remain elusive. In an effort to better understand molecular mechanisms of Aβ misfolding and fibrillization, we performed all-atom molecular dynamics (MD) simulations of interaction of Aβ_1-42_ chains with fragments of β-rich amyloid fibrils in water. As the fibril models, we employed the solution NMR model 2NAO [3] and the cryo-EM model 5OQV [4]. Four disordered Aβ_1-42_ chains were placed in random positions at a distance from the fibrils, and evolution of the entire systems was investigated. Multiple independent simulations were done for each system. For each fibril model, we observed attachment of Aβ_1-42_ chains to the fibril. Importantly, both threading of Aβ_1-42_ chains around the edge of the fibril (precursor of fibril elongation) and attachment of the chains to fibril’s side surface (precursor of secondary nucleation) were detected with each of two fibril models. For the 5OQV model, threading around both the ‘ridge’ and ‘groove’ ends of the fibril were observed. Surprisingly, attachment of Aβ_1-42_ chains to side surface of 5OQV fibrils occurred with the Aβ chains’ preferential orientation along the fibril axis, rather than along cross-β strands. In one of simulations with the 2NAO fibril model, we observed detachment of an Aβ chain caused by its interaction with a different Aβ chain in solution (precursor of fibril dissociation). In contrast, the 5OQV fibrils have exhibited a remarkable structural stability in our simulations. In conclusion, the MD simulations have shed light into early stages of two major molecular mechanisms of misfolding and aggregation of Aβ_1-42_, fibril elongation and secondary misfolding, as well as captured a fibril dissociation event, revealing important molecular-level details behind these processes.

### 

References[1]Eisenberg & Jucker, Cell
2012; 6:1188–1203.10.1016/j.cell.2012.02.022PMC335374522424229[2]Cohen et al., PNAS
2013; 110:9758–9763.[3]Walti et al., PNAS
2016; 113:E4976-E4984.10.1073/pnas.1600749113PMC500327627469165[4]Gremer et al., Science
2017; 358:116–119.

## 154. TSEs in European goats discriminated by Western blotting into five types based on five robust molecular parameters

L Pirisinu^a^, O Andreoletti^b^, I Lantier^c^, PL Acutis^d^, C Acin^e^, W Goldmann^f^, T Sklaviadis^g^, L Ekateriniadou^h^, C Fast^i^, P Papasavva – Stylianou^j^, S Simon^k^, J Spiropoulos^l^, U Agrimi^a^, JG Jacobs^m^, A Bossers^m^, LJM van Keulen^m^, M Mazza^d^, R Nonno^a^ and JPM Langeveld^m^

^a^Istituto Superiore di Sanità, Department of Food Safety, Nutrition and Veterinary Public Health, Rome, Italy; ^b^UMR INRA ENVT 1225- IHAP, École Nationale Vétérinaire de Toulouse, Toulouse, France; ^c^INRA-Centre Val de Loire, Infectiologie et Santé Publique, Nouzilly, France; ^d^Istituto Zooprofilattico Sperimentale del Piemonte, Liguria e Valle d’Aosta, Torino, Italy; ^e^Centro de Encefalopatías y Enfermedades Transmisibles Emergentes, Facultad de Veterinaria, Universidad de Zaragoza, Zaragoza, Spain; ^f^The Roslin Institute and Royal (Dick) School of Veterinary Studies, University of Edinburgh, Easter Bush, United Kingdom; ^g^Laboratory of Pharmacology, School of Health Sciences, Department of Pharmacy, Aristotle University of Thessaloniki, Thessaloniki, Greece; ^h^National Agricultural Research Foundation, Veterinary Research Institute, Thessaloniki, Greece; ^i^Institute of Novel and Emerging Infectious Diseases, Friedrich-Loeffler-Institute, Greifswald-Isle of Riems, Germany; ^j^Veterinary Services, Nicosia, Cyprus; ^k^Service de Pharmacologie et Immunoanalyse (SPI), Laboratoire d’Etudes et de Recherches en Immunoanalyse, CEA, INRA, Université Paris-Saclay, Gif-sur-Yvette, France; ^l^Animal and Plant Health Agency, New Haw, Addlestone, Surrey, United Kingdom; ^m^Wageningen BioVeterinary Research, Lelystad, the Netherlands

**CONTACT** Laura Pirisinu laura.pirisinu@iss.it

**ABSTRACT**

Contrasting the knowledge about prion diseases or TSEs in sheep, only a very limited number of strain typing studies are available in goats. Two cases deriving from the zoonotic bovine BSE epidemic were however detected in goats. During 2004–2012, over 70 TSE goat brain samples were collected from seven European countries and evaluated for TSE type/strain variation. A selection of these materials was chosen for in-depth analysis based on various criteria: tissue quality, genotype, broad geographical distribution, potential type variation. Of these, 37 cases were biochemically analysed (present study) and a subset of these subjected to bioassays in seven rodent models (see parallel study of Nonno et al.). Analyses of these isolates showed that in goats different PrP^Sc^ types exist, comparable to those found in sheep such as classical scrapie, CH1641-like scrapie and Nor98/atypical scrapie. However, classical scrapie isolates could be further differentiated in two molecular subtypes based on the PK susceptibility of the N-terminus and on the molecular weight of PrP^res^. Importantly, the two subtypes of classical scrapie showed different geographical distribution, as one subtype was only detected in goats deriving from Italy and France. These analyses also show, that none of the field samples exhibited BSE-like features and offer a comprehensive set of robust molecular PrP^res^ properties to exclude suspicion for the presence of BSE. These are: molecular mass of the triplet bands, presence of N-terminus epitope, glycoprofile markers, a dual population marker and the structural stability of PrP-core.

This work was made possible by national support and by the following European grants: Neuroprion (FP6-FOOD-506,579), GoatBSE (FOOD-CT-2006–36,353) and GOAT-TSE-FREE (EMIDA-ERA NET). We want to memorize Jorg G Jacobs, who died in 2017. Jorg developed and performed the Triplex-WB system as well as characterized antibodies like 94B4, 12B2, 9A2, 6C2, SAF84 and L42. He also was crucial in the distinction of C-, L- and H-type BSE and in 2011 he showed elegantly the allotype composition of prion material in heterozygous sheep by the use of Endo-LysC.

## 155. Expert opinions on the potential role of Indigenous peoples in wildlife management in Alberta

Arlana Bennett

University of Alberta

**CONTACT** Arlana Bennett bennettc@ualberta.ca

**ABSTRACT**

**Background**: Natural resource management in many parts of the world are increasingly based around the recognition of Indigenous knowledge and the rights of resource users [1]. In Alberta, there has been a historical lack of consideration of the knowledge of First Nations and Métis peoples and decisions about the development, use, management and monitoring of natural resources [2–5]. As a result of these histories, Indigenous peoples and their knowledge have little influence within provincial wildlife management regimes in contemporary Alberta [6]. Wildlife diseases such as Chronic Wasting Disease (CWD) have added further challenges for wildlife managers in Alberta. CWD is a fatal form of transmissible spongiform encephalopathy (TSE) primarily found in cervids (e.g. deer, moose, elk, and caribou). CWD in Alberta as of the 2017/2018 hunting season, has continued to spread westward along the Red Deer/South Saskatchewan/Bow watershed, the Battle watershed, and north along the Alberta/Saskatchewan border. Incidence of deer that have tested positive for CWD has increased markedly since the 2016/2017 hunting seasons, and several new Wildlife Management Units (WMU) will be added to the mandatory surveillance list for the 2018/19 hunting season [7]. First Nations and Métis communities in the Treaty 6 area – including Saddle Lake First Nation, Frog Lake First Nation, Kehewin First Nation, Fishing Lake Métis settlement, and the Elizabeth Métis settlement – are within the mandatory surveillance zone yet it remains unclear whether these communities are being actively engaged in the process of management.

**Methods**: This research sought to explore diverse expert perspectives on the role of Indigenous Knowledge in wildlife monitoring and management in relation to the issue of CWD; and better understand the key challenges and opportunities regarding wildlife management in Alberta. The methods used in this thesis include a modified qualitative expert elicitation, probabilistic sampling, and thematic analysis.

**Results**: The major thematic results experts discussed include: the lack of Indigenous compliance in cervid monitoring with varying reasons provided; the necessity of both scientists and Indigenous communities to engage in intercultural and technical capacity development; and the need for both scientists and Indigenous communities to form a functional and mutually beneficial working relationship. This research is a preliminary investigation into the social, cultural, and economic aspects of CWD management, and is intended to provide further insights towards this end with a focus on future areas of research.

### 

References[1]Sacred EcologyBerkes F.
2018.[2]SandlosJ.
Hunters at the margin. 2007.[3]LooT.
States of nature. 2006.[4]FumoleauR.
As long as this land shall last. 2004.[5]CalliouBL.
Losing the game. 2000.[6]Natcher, et al. Applied Anthropology
2009;68:245–257.[7]AEP (Alberta Environment and Parks). CWD Updates
2018.

## 156. Screening and characterization of unusual sCJD cases in a CWD endemic state in the USA

Yihui Liu^a^, Manuel Camacho^a^, Wenquan Zou^a,b,c^, Qingzhong Kong^a,b,c^

^a^Department of Pathology, Case Western Reserve University (CWRU), Cleveland, USA; ^b^Department of Neurology, CWRU, Cleveland, OH, USA; ^c^National Center for Regenerative Medicine, CWRU, Cleveland, USA

**CONTACT** Qingzhong Kong qxk2@case.edu

**ABSTRACT**

**Background**: Chronic wasting disease (CWD) has spread to 26 states in the USA and three provinces in Canada, and it has been detected recently in Norway and Finland. Potential CWD zoonosis is a serious public health concern. It is unclear whether CWD transmission to humans has already occurred. We aim to start to address this question by examining all available sCJD cases from a CWD endemic state in the USA.

**Methods**: Frozen brain tissues from all available sCJD cases archived in the National Prion Disease Pathology Surveillance Center from a US state that has been significantly impacted by CWD were sampled at five brain regions. These brain samples were subjected to detailed biochemical analysis to look for unusual patterns, characteristics, and/or distribution of PrP^Sc^ in comparison with sCJD samples from states that have not detected CWD. Unusual cases are further scrutinized for their clinical presentations, histopathological features, and history of cervid hunting and venison consumption.

**Results and Conclusions**: We have found some unusual sCJD cases in this CWD endemic state. We will report our preliminary findings on their features. Currently there is no convincing evidence to support a direct link to CWD for any of these unusual sCJD cases.

**Disclaimer**: The findings and conclusions in this report are those of the authors and do not necessarily represent the position of the National Prion Disease Pathology Surveillance Center.

## 157. Utility of RT-QuIC in predicting case status of potential Creutzfeldt-Jakob Disease (CJD) cases

Natalie S. Marzec^a^, Marie-Ange Smith^b^, Jennifer A. House^a^

^a^Colorado Department of Public Health and Environment, Denver, CO, USA; ^b^Colorado School of Public Health, Aurora, CO, USA

**CONTACT** Natalie S. Marzec natalie.marzec@state.co.us

**ABSTRACT**

**Background**: Ante-mortem diagnosis of human prion disease is limited by the high false positivity rate of the testing methods available for cerebrospinal fluid (CSF). This has resulted in a lack of efficiency when investigating potential prion disease cases for surveillance purposes. In 2018, the real time quake-inducing conversion (RT-QuIC) test was added to the CDC diagnostic criteria for Creutzfeldt-Jakob Disease (CJD), the most common prion disease reported in humans. RT-QuIC appears to be a better predictor of Definite or Probable CJD case status than the older Tau and 14-3-3 protein tests. The purpose of this project was to evaluate the utility of RT-QuIC to predict case status of potential CJD cases.

**Methods**: Potential cases of CJD reported in Colorado between 1 January 2015, and 31 December 2017 (prior to the change in the CDC diagnostic criteria), were reviewed for final case status, testing methods, and ante-mortem test results. RT-QuIC, Tau, and 14-3-3 test results were compared to final case status to determine the positive predictive value (PPV), negative predictive value (NPV), sensitivity, and specificity of each test. Cases that met either Definite or Probable case definitions were considered to be true cases.

**Results**: Sixty-six potential cases of CJD reported in the specified time period were investigated and assigned a case status of Definite, Probable, Possible, or did not meet case definition. Of these cases, 27 (41%) were assigned a Definite or Probable case status. RT-QuIC was found to have a PPV of 92.8%, a NPV of 100%, a sensitivity of 100% and a specificity of 95.8%. Tau protein had a PPV of 48.6%, a NPV of 93.8%, a sensitivity of 94.7%, and a specificity of 55.9%. 14-3-3 protein had a PPV of 32.4%, a NPV of 83.3%, a sensitivity of 92.3%, and a specificity of 16.7%.

**Conclusions**: RT-QuIC testing is a more useful ante-mortem predictor of Definite or Probable CJD case status than either Tau or 14-3-3 protein testing. Public health investigators can place more weight on RT-QuIC testing than other ante-mortem CJD disease testing when triaging cases for investigation. To our knowledge, this is the first time RT-QuIC testing has been studied in relation to predicting public health surveillance case status.

**KEYWORDS:** Creutzfeldt-Jakob disease; RT-QuIC; public health; surveillan

## 158. Familial Creutzfeldt-Jakob disease with D178N and Met129Val

Nurit Omer^a^, Esther Kahana^b^, Sharon Simhoni^c^, Anat Bar-Shira^c^, Tova Naiman^c^, Avi Orr-Urtreger^c^, Nir Giladi^d^ and Noa Bregman^a^

^a^CJD clinic, Neurological institute, Tel Aviv Sourasky Medical Center, Tel Aviv, Israel; ^b^Department of Neurology, Barzilai Medical Center, Ashkelon, Israel; ^c^The Genetic Institute, Tel-Aviv Sourasky Medical Center, Tel-Aviv, Israel; ^d^Neurological institute, Tel Aviv Sourasky Medical Center, Tel Aviv, Israel

**CONTACT** Nurit Omer nurito@tlvmc.gov.il

**ABSTRACT**

Fatal familial insomnia (FFI) and Creutzfeldt-Jakob disease (CJD) are two phenotypes that share a common point mutation at codon 178 of the prion protein gene (*PRNP*), with the substitution of aspartic acid to asparagine (D178N). The phenotype depends on the polymorphism at codon 129, when methionine (129M) is associated with FFI, and Valine (129V) with CJD. We here report on a Jewish family from Iraqi origin with D178N-129V genotype presenting with overt insomnia, cerebellar ataxia, and an atypical duration of disease. Patient 1, a 56-year-old woman without family history of neurological illness, presented in 1996 with rapidly progressive dementia and gait ataxia. MRI showed frontal cortical but not basal ganglia or thalamic involvement. CSF was positive for protein 14-3-3, and genetic testing for E200K mutation was negative. Brain biopsy showed typical spongiform changes, and she was diagnosed with sporadic CJD. She passed away 7 years after symptoms onset. Her son (patient 2) presented on 2012 with rapidly progressive dementia at the age of 41 and was diagnosed with CJD. He died a few months later. Patient 3, her daughter, presented on 2018 at the age of 50, with cerebellar ataxia and mild cognitive impairment. She described severe insomnia for more than a year before arrival. Brain MRI showed overt restriction in diffusion across the frontal, parietal and temporal cortices, with marked involvement of basal ganglia and thalami. A genetic analysis of the *PRNP* gene revealed a heterozygous D178N mutation (sequence variant: c.532G>A) in combination with the polymorphism Met129Val (sequence variant: c.385A>G) on both alleles (homozygous). Patient 4, her third daughter, 45 years old, is currently (2019) undergoing medical evaluation due to reported progressive cognitive decline for 6 months. The phenotype of patient 1 was clearly different from that of FFI and more suggestive of CJD, although the long duration of the disease is highly unusual for CJD. Severe insomnia was reported in the ‘prodromal’ phase in patient 3, with marked thalami involvement in MRI, but with a genetic signature that is consistent with CJD. Our findings support the notion that there is a phenotypic variability in the D178N genotype within the same family, and emphasize the heterogeneity of inherited prion disease and the possibility that genetic and other modifiers exist.

## 159. Therapeutic and clinical strategy to prevent or delay onset of genetic prion disease using prion protein-lowering antisense oligonucleotides

Eric Vallabh Minikel^a,b,c^, Hien Tran Zhao^d^, Deb Cabin^e^, Stuart L. Schreiber^a,f^, Jeffrey B. Carroll^e^, Holly Kordasiewicz^d,g^, Steven E. Arnold^b,h^, Byron Caughey^i^ and Sonia M. Vallabh^a,b,c^

^a^Broad Institute of MIT and Harvard, Cambridge, MA, USA; ^b^Harvard Medical School, Boston, MA, USA; ^c^Prion Alliance, Cambridge, MA, USA; ^d^Ionis Pharmaceuticals Inc, Carlsbad, CA, USA; ^e^McLaughlin Research Institute, Great Falls, MT, USA; ^f^Harvard University, Department of Chemistry and Chemical Biology, Cambridge, MA, USA; ^g^Western Washington University, Bellingham, WA, USA; ^h^Massachusetts General Hospital, Department of Neurology, Boston, MA, USA; ^i^Laboratory of Persistent Viral Diseases, Rocky Mountain Laboratories, National Institute of Allergy and Infectious Diseases, National Institutes of Health, Hamilton, MT, USA

**CONTACT** Sonia M. Vallabh svallabh@broadinstitute.org

**ABSTRACT**

**Background**: Genetic proofs of concept suggest that reduction of prion protein (PrP) in the brain should be an effective and well tolerated therapeutic strategy for prion disease. We are therefore investigating PrP-lowering antisense oligonucleotides (ASOs) as a potential prion disease therapeutic. Based on initial observations of ASO efficacy in prion-infected mice (see complementary abstract by Dr Byron Caughey) here we assess parameters of ASO efficacy including dose responsiveness, strain specificity, and impact of treatment timepoint. As early treatment conveys greater benefit, we also describe a framework for testing PrP-lowering therapeutics in presymptomatic prion disease mutation carriers, including biomarker development, establishment of a carrier cohort and regulatory engagement.

**Materials and methods**: All mice received bolus doses of active ASOs or saline by stereotactic intracerebroventricular injection, then were monitored for symptoms with a primary outcome of terminal prion disease. ASO doses, prion strains and treatment initiation timepoint varied across experiments. To evaluate CSF PrP as a biomarker for PrP-lowering therapeutics, *N *= 217 human CSF samples spanning a range of diagnoses and prion disease subtypes were subjected to PrP quantification by ELISA.

**Results**: ASO efficacy is dose responsive, universal across all prion strains tested, and more efficacious if administered early. In an ongoing experiment, PrP-lowering ASOs were administered every 90 days beginning at −14 dpi; all saline-treated animals have reached prion endpoint (mean 143 dpi) while all but one ASO-treated animal is living at 316 dpi, corresponding to a ≥ 2.2x extension of survival. CSF PrP is detectable in all human CSF samples tested, reliably quantifiable by ELISA subject to proper handling, appears CNS-derived, and exhibits excellent short-term within-subject test-retest reliability (mean CV = 8.5%) in genetic prion disease mutation carriers, suggesting that ASO-mediated PrP lowering should be readily detectable in the context of a trial. In a recent Critical Path Innovation Meeting, we discussed with FDA scientists a path to presymptomatic trials in genetic prion disease mutation carriers based on CSF PrP lowering as surrogate endpoint for provisional approval of an ASO.

**Conclusions**: These data support advancement of PrP-lowering ASOs to the clinic, and support the feasibility of primary prevention trials in healthy genetic prion disease mutation carriers. We are working closely with our pharmaceutical partner, Ionis Pharmaceuticals, as well as with FDA to advance ASOs to the clinic within the next few years. Genetic prion disease is poised to become a model for primary prevention in neurodegeneration.

## 160. Rapid bioassay of mammalian prions in *Drosophila*

Alana M. Thackray and Raymond Bujdoso

Department of Veterinary Medicine, Cambridge University, Cambridge, UK

**CONTACT** Raymond Bujdoso rb202@cam.ac.uk

**ABSTRACT**

Mammalian prions cause fatal neurodegenerative diseases such as Creutzfeldt-Jakob disease (CJD) in humans, Bovine Spongiform Encephalopathy (BSE) in cattle, chronic wasting disease (CWD) in cervids and scrapie disease of sheep. These conditions are transmissible between individuals of the same or different species and as a consequence, animal prion diseases pose a realistic threat to human health through their zoonotic potential. Prions lack a conventional nucleic acid-based genome. The ‘protein only’ concept predicts that infectious prion particles consist of PrP**^Sc^** in the form of aggregates of misfolded conformers of the normal host protein PrP**^C^**, although the exact molecular nature of this transmissible entity is undefined. As a consequence, the only reliable method to detect mammalian prion infectivity is by bioassay in an appropriate experimental host. It is important to be able to detect prion infectivity in tissues and fluids from individuals affected by prion disease in order to protect human and animal health. In addition, it is important to address the zoonotic potential of animal prion infectivity in order to ensure the safety of the human food chain. The detection of prion infectivity by bioassay in mammalian species, most commonly mice, is time consuming and expensive, and consequently, low numbers of samples are routinely assessed. We have previously demonstrated *Drosophila* can bioassay mammalian prions (Thackray, et al. Brain 2018; 141:2700–2710). Our studies have shown that ovine PrP transgenic *Drosophila* develop a transmissible neurotoxic phenotype, associated with Proteinase-K resistant PrP**^Sc^**, after exposure to exogenous ovine prions. We have recently generated *Drosophila* transgenic for human, bovine or cervid PrP and have demonstrated that these flies can be used successfully to bioassay variant CJD, BSE or CWD samples, respectively. In addition, we have shown that human PrP transgenic *Drosophila* can be used to model the zoonotic potential of animal prions. Collectively, our novel data show that *Drosophila* can detect human and animal prion infectivity in a rapid and efficient manner, and can contribute to an understanding of the zoonotic potential of animal prion disease.

## 161. Rodent models allow BSE discrimination of goat prions and reveal geographical differences in the biological properties of scrapie

R. Nonno^a^, A. Marin-Moreno^b^, J.C. Espinosa^b^, C. Fast^c^, L. Van Keulen^d^, J. Spiropoulos^e^, I. Lantier^f^, O. Andreoletti^g^, L. Pirisinu^a^, M.A. Di Bari^a^, P. Aguilar-Calvo^b^, T. Sklaviadis^h^, P. Papasavva‑Stylianou^i^, P.L. Acutis^j^, C. Acin^k^, A. Bossers^d^, J.G. Jacobs^d^, G. Vaccari^a^, C. D’Agostino^a^, B. Chiappini^a^, F. Lantier^f^, M. Groschup^c^, U. Agrimi^a^, J.M. Torres^b^ and J.P.M. Langeveld^d^

^a^Istituto Superiore di Sanità, Department of Food Safety, Nutrition and Veterinary Public Health, Rome, Italy; ^b^Centro de Investigación en Sanidad Animal, CISA-INIA, Madrid, Spain; ^c^Institute of Novel and Emerging Infectious Diseases, Friedrich-Loeffler-Institute, Greifswald-Isle of Riems, Germany; ^d^Wageningen BioVeterinary Research, Lelystad, the Netherlands; ^e^Animal and Plant Health Agency, New Haw, Addlestone, Surrey, United Kingdom; ^f^INRA-Centre Val de Loire, Infectiologie et Santé Publique, Nouzilly, France; ^g^UMR INRA ENVT 1225- IHAP, École Nationale Vétérinaire de Toulouse, Toulouse, France; ^h^Laboratory of Pharmacology, School of Health Sciences, Department of Pharmacy, Aristotle University of Thessaloniki, Thessaloniki, Greece; ^i^Veterinary Services, Nicosia, Cyprus; ^j^Istituto Zooprofilattico Sperimentale del Piemonte, Liguria e Valle d’Aosta, Torino, Italy; ^k^Centro de Encefalopatías y Enfermedades Transmisibles Emergentes, Facultad de Veterinaria, Universidad de Zaragoza, Zaragoza, Spain

**CONTACT** Romolo Nonno romolo.nonno@iss.it

**ABSTRACT**

Classical scrapie (CS) is a contagious TSE of small ruminants known to circulate in Europe for centuries. More than one TSE strain is responsible for CS, still our ability to identify scrapie strains remains limited, as it has been difficult so far to reconcile data obtained in different rodent models. After the discovery of BSE in two goats, a European-broad effort was undertaken in order to understand the diversity of goat TSE strains and to discriminate BSE from them. Here, we studied goat TSEs by their relative transmission efficiency in different animal models and by the PrP^Sc^ type(s) propagated in rodents. Thus, the biological properties of TSE isolates were inferred by their specific interaction with different recipient PrP species rather than by serial passage in a single model. Goat TSE isolates from seven countries and experimental goat-BSE were inoculated in seven models: tg-mice overexpressing sheep/goat ARQ, sheep VRQ, cattle or mouse PrPs; RIII mice and bank voles. The attack rate and the survival time were combined to obtain the ‘transmission efficiency’, a parameter used to build the ‘transmission profiles’ of individual isolates across all models. Goat-BSE and Nor98 showed distinctive transmission profiles, different among them and from all other isolates. Goat-BSE was the only isolate inducing 19kDa PrP^Sc^ in all rodent models, while Nor98 was associated with 8kDa PrP^Sc^. CS isolates showed a strong variability of transmission profiles, which was in part explained by their geographical distribution, and could be grouped into four distinct categories. CS isolates in category 1 induced the propagation solely of 21kDa PrP^Sc^, while isolates from CS categories 2-to-4 also induced, to a different extent depending on the model, a 19kDa PrP^Sc^ type distinct from BSE. Further investigation of CNS and LRS tissues from a goat herd showed that this phenomenon reflects the selective amplification in rodents of at least two co-existing ‘sub-strain components’, 21kDa and 19kDa, whose relative amount varies with different goat tissues/isolates. Our findings show that BSE can be discriminated from a wide range of biologically and geographically diverse goat TSEs. The geographical variation observed in CS suggests the existence of distinct CS strains, as also supported by the parallel studies by Pirisinu et al. and Marin-Moreno et al. Yet, CS isolates in categories 2-to-4 were mixtures of discrete sub-strains, where it could be envisaged that competition among sub-strains play a role in defining the biological properties of individual isolates and their evolution.

## 162. ‘Multi-species anti-prion oral vaccine for familial and transmissible spongiform encephalopaties’

Fernando Goñi^a^, Analia Elisei^b^, Lucia Yim^c^, Mitchell Marta-Ariza^a^, Jose A Chabalgoity^c^, Thomas Wisniewski^a,d^

^a^Department of Neurology New York University, NY, USA; ^b^INTA, Buenos Aires Argentina; ^c^Biotech Department of Universidad de la Republica, Uruguay; ^d^Depts of Pathology and Psychiatry NYUSoM, USA

**CONTACT** Fernando Goñi Fernando.goni@nyumc.org

**ABSTRACT**

**Background/Introduction**: Transmissible prion diseases have become a worldwide problem of enormous proportions with no prevention or cure in sight. The North American and northern Europe Chronic Wasting disease (CWD) epidemic in free range moose, elk and deer has not been contained. We were successful in developing an oral vaccination with PrP molecules delivered by an attenuated Salmonella carrier to prevent transmission and infection of susceptible mice, and achieve, in white-tailed deer, partial mucosal and systemic antibody protection to an oral challenge with an infective CWD-prion [1,2]. We have now refined the constructs to small peptides with specificities for multiple mammalian PrP molecules for a more effective oral vaccine.

**Materials and Methods**: We designed and constructed 20–30 residues long multi-species PrP-like peptides with amino acids specific for at least three different species and encompassing the whole PrP molecule. Peptides were expressed in an attenuated Salmonella delivery system, or controlled glutaraldehyde polymerized with and without cholera toxin-B (CTB). Vaccine preparations were kept for 7 days at 4°C and then for 36 h at RT. All preparations were inoculated by gavage into mice transgenic for different species PrP. After three weekly inoculations antibody production was measured in faeces and serum.

**Results**: Viability of the vaccines after 10 days was between 87% and 95%. Using a reduced number of inoculations, all Tg CD-1 mice expressing human, elk, sheep or mouse PrP and PrP-KO produced a sustained antibody response (both IgA and IgG) that cross-reacted with prion proteins from different species.

**Conclusions**: Stability and ease of production of these new vaccine formulations would allow for up scaling and use in larger animals of any species administered as food supplement in enclosed facilities or potentially as bait in the wild. The cross-reactivity of the antibodies produced would help neutralize any infective prion from oral transmission and inhibit spread of the β-sheet structure responsible for PrP^C^ to PrP^Sc^ conversion [3].

**KEYWORDS:** CWD; pan-vaccine; multi-species; oral vaccine

### 

References[1]Goñi, et al
Neuroscience
2008; 153:679686.[2]Goñi, et al
Vaccine
2015; 33:726733.[3]Wisniewski and Goñi. Handbook of Clinical Neurology
2018; 153:419–430.10.1016/B978-0-444-63945-5.00023-429887149

## 163. Structural characterization of α-synuclein prions via cryo-electron microscopy

Gregory E. Merz^a^, Eric Tse^a^, Victor Banerjee^a^, Amanda L. Woerman^a,b^, William F. DeGrado^a,d^, Daniel R. Southworth^a,c^ and Stanley B. Prusiner^a,b,c^

^a^Institute for Neurodegenerative Diseases, Weill Institute for Neurosciences, University of California, San Francisco, CA, USA; ^b^Department of Neurology, University of California, San Francisco, CA, USA; ^c^Department of Biochemistry and Biophysics, University of California, San Francisco, CA, USA; ^d^Department of Pharmaceutical Chemistry, Cardiovascular Research Institute, University of California, San Francisco, CA, USA

**CONTACT** Gregory E. Merz gregory.merz@ucsf.edu

**ABSTRACT**

Recent structural characterization via cryo-electron microscopy (cryo-EM) has confirmed at high-resolution that different fibrillar structures of tau prions (or tau strains) exhibit clinically distinct tauopathy disorders. Such findings are consistent with the hypothesis that the biological properties of prion strains are determined by their misfolded conformations. Similarly, several groups have recently demonstrated that the synucleinopathies of Parkinson’s disease (PD) and multiple system atrophy (MSA) are caused by α-synuclein aggregates with distinct biological activities. Given that the structure of prions determines their biological properties, we hypothesize that α-synuclein prions in PD and MSA have unique fibrillar conformations. We are investigating this structural hypothesis by characterizing α-synuclein prions at high resolution using cryo-EM. Several strains of α-synuclein prions will be purified from human brain as well as homogenates passaged through Tg mice and cultured cells. We will perform cryo-EM on proteins purified from brain homogenates to obtain the high-resolution information necessary to make structural comparisons between strains at the molecular level. The overall folds of prions, as well as inter- and intra-molecular interactions, will be evaluated across prion strains. Mutation sites in α-synuclein causing familial synucleinopathies (e.g. A53T) are of particular interest, and the interactions of these residues within the larger fibrillar fold will be closely studied. The unequivocal assignment of specific α-synuclein conformations with the resulting disease is likely to represent a major step forward in our understanding of the pathogenesis of synucleinopathies.

## 164. Overview of scrapie cases in Iceland 2004–2018

Eva Hauksdottir and Stefania Thorgeirsdottir

Keldur, the Institute for Experimental Pathology, University of Iceland

**CONTACT** Eva Hauksdottir evahauks@hi.is

**ABSTRACT**

**Introduction**: Since 1978 Iceland has actively screened healthy slaughtered (HS) sheep for scrapie, however, in 2004 the screening method changed from histopathological staining to a rapid ELISA test. With this new method scrapie cases in HS were identified for the first time, as before index scrapie cases had only been detected in sheep with clinical symptoms (CS). Use of this sensitive method also led to the detection of the first atypical scrapie case (Nor98) in Iceland in 2004.

**Materials and Methods**: During 2004–2018 there were 50,515 HS samples tested with ELISA, along with 558 samples from fallen stock and clinical suspects. In this overview, the cases detected during this time, will be analysed further, e.g. origin, age, PrP genotype and number of additional cases from each farm detected after culling of the scrapie flock.

**Results**: In the years 2004–2018 there were 29 scrapie cases identified, where one case refers to a single affected farm. Two thirds of the classical scrapie cases originate from CS sheep, while Nor98 is generally identified in HS samples rather than CS. The genotype most associated with susceptibility to classical scrapie is frequent in the classical scrapie cases, however a neutral genotype is found in many of those cases as well. After culling, where a scrapie flock (or a portion of it) was tested, additional positive samples of classical scrapie were frequently detected, but only once has an additional Nor98 case been found in a culled flock.

**Conclusion**: The comparison of classical and atypical scrapie in Iceland shows that they have many different qualities in relation to origin, symptoms, genotype and flock infectivity.

## 165. Familial Parkinson’s point mutation abolishes multiple system atrophy prion replication

Amanda L. Woerman^a,b^, Sabeen Kazmi^a^, Smita Patel^a^, Abby Oehler^a^, Kartika Widjaja^a^, Daniel A. Mordes^c^, Steven H. Olson^a,b^ and Stanley B. Prusiner^a,b,d^

^a^Institute for Neurodegenerative Diseases, Weill Institute for Neurosciences, University of California, San Francisco, CA, USA; ^b^Department of Neurology, University of California, San Francisco, CA, USA; ^c^C.S. Kubik Laboratory for Neuropathology, Department of Pathology, Massachusetts General Hospital, Boston, MA, USA; ^d^Department of Biochemistry and Biophysics, University of California, San Francisco, CA, USA

**CONTACT** Amanda L. Woerman amanda.woerman@ucsf.edu

**ABSTRACT**

In the neurodegenerative disease multiple system atrophy (MSA), α-synuclein misfolds into a self-templating conformation to become a prion. To compare the biological activity of α-synuclein prions in MSA and Parkinson’s disease (PD), we developed nine α-synuclein-YFP cell lines expressing point mutations responsible for inherited PD. MSA prions robustly infected wild-type, A30P, and A53T α-synuclein-YFP cells, but they were unable to replicate in cells expressing the E46K mutation. Coexpression of the A53T and E46K mutations was unable to rescue MSA prion infection *in vitro*, establishing that MSA a-synuclein prions are conformationally distinct from the misfolded α-synuclein in PD patients. This observation may have profound implications for developing treatments for neurodegenerative diseases.

## 166. α-cleavage of PrPC by Aspirin suppresses prion infection

Huyen Trang Trinh^a^, Taeyeon Kim^b^, A-Ran Kim^a^, Junwoo Shin^a^, Hakmin Lee^a^, Jieun Kim^a^, Sungeun Lee^a^, Chongsuk Ryou^a^

^a^Department of Pharmacy, College of Pharmacy and Institute of Pharmaceutical Science and Technology, Hanyang University, Ansan, Gyeonggi-do, Republic of Korea; ^b^Division of Developmental Biology and Physiology, School of Biosciences and Chemistry, Institute for Basic Science, Sungshin University, Seoul, Republic of Korea

**CONTACT** Huyen Trang Trinh ds.trinhthihuyentrang@gmail.com; Chongsuk Ryou cryou2@hanyang.ac.kr

**ABSTRACT**

**Background:** The conversion of the cellular prion protein, PrPC, into its pathogenic form, PrPSc, is the hallmark of prion diseases. In physiological conditions, PrPC can be cleaved at α-site, which generates the C1 and N1 fragments, and disrupts a region critical for conversion into PrPSc. Plasmin is known as an enzyme that cleaves PrPC at α-site; however, the function of plasmin in prion propagation remains elusive. In the current study, the role of plasmin and Aspirin, known to stimulate plasmin activity, in α-cleavage of PrPC and prion suppression was investigated.

**Material and Methods:** The effect of plasmin and Aspirin was evaluated using an in vitro assay, which mimics the aggregation process of prion protein into amyloids. In the cell-based model, ScN2a cells were incubated with Aspirin and endogenous plasmin activity was measured. The generation of C1 and N1 fragments were also detected by immunoblotting. The elimination of PrPSc by Aspirin was investigated in ScN2a. Finally, the ability of Aspirin to interfere with the susceptibility of N2a to prions was determined using standard scrapie cell assay.

**Results:** Aspirin promoted plasmin activity by inducing α-cleavage of PrP, resulting in the inhibition of PrP aggregation in vitro. Moreover, the endogenous plasmin activity in the ScN2a was significantly enhanced by the action of Aspirin. The generation of C1 fragment was increased in the membrane fraction of the cells incubated with Aspirin, indicating that Aspirin activated α- cleavage of PrPC. In addition, Aspirin significantly reduced the level of PrPSc in cells permanently infected with prions. Finally, Aspirin significantly decreased the number of N2a cells infected with prions.

**Conclusions:** Aspirin promoting plasmin-mediated α-cleavage of PrPC suppressed PrPSc propagation.

**KEYWORDS:** Aspirin; plasmin; α-cleavage; C1 fragment

## 167. Two different conformers of type 1 prion protein propagate as distinct strains in transgenic mice

Ignazio Cali^a,b,^* Juan Carlos Espinosa^c,^*, Tetsuyuki Kitamoto^d^, Alba Marin-Moreno^c^, Brian S. Appleby^a,b,e^, Juan Maria Torres^c^ and Pierluigi Gambetti^a^

^a^Department of Pathology and ^b^National Prion Disease Pathology Surveillance Center (NPDPSC), Case Western Reserve University, School of Medicine, Cleveland, OH 44,106, USA; ^c^Centro de Investigación en Sanidad Animal, CISA-INIA, Madrid, Spain; ^d^Department of Neurological Science, Tohoku University Graduate School of Medicine, Sendai, Japan; ^e^Department of Neurology, University Hospitals Cleveland Medical Center, Beachwood, OH 44,122, USA.

**CONTACT** Ignazio Cali ignazio.cali@case.edu

*These authors contributed equally to this work

**ABSTRACT**

A modern classification recognizes five subtypes of sporadic Creutzfeldt-Jakob disease (sCJD)-based on the pairing of methionine (M)/valine (V) polymorphic genotype at codon 129 of the prion protein (PrP), which determines the MM, MV and VV 129 genotypes, and the type 1 or 2 of the disease-associated PrP (PrP^D^)^1^. These two PrP^D^ types are distinguished on the basis of their different electrophoretic mobility as the PK-resistant PrP^D^ (resPrP^D^) type 1 and type 2 migrate to ~21 and ~19 kDa, respectively. In sCJDVV1, the electrophoretic profile of resPrP^D^ is distinct from that of resPrP^D^ type 1 associated with the sCJDMM(MV)1 subtype. It features a heterogeneous component, which in its unglycosylated form appears in three versions: a single band of ~20 kDa (T1^20^), of ~21 kDa (T1^21^) or as a doublet with both ~20 and ~21 kDa bands (T1^20−21^). Although these bands are a minor component resPrP^D^, their consistent presence raises the issue as to whether they are a feature that distinguish the PrP^D^ strain associated with sCJDVV1 from that associated with sCJDMM(MV)1 and other type 1 strains. We have carried out a retrospective examination of brain homogenates from subjects with the definitive diagnosis of sCJDVV1 that were collected at the National Prion Disease Pathology Surveillance Center (NPDPSC). Western blot (WB) analysis confirmed the presence of the three distinct electrophoretic profiles of unglycosylated resPrP^D^. To determine whether these distinct molecular signatures of resPrP^D^ associated with sCJDVV1 could be propagated *in vivo*, brain homogenates from sCJDVV1 cases harbouring each of the three profiles were inoculated to transgenic (Tg) mice expressing human PrP-129V. The T1^20^ and T1^21^ WB profiles were faithfully replicated in the Tg mice. Although as in sCJDVV1 they were associated with undistinguishable histopathological phenotypes the incubation periods following T1^20^ and T1^21^ inoculations differed. Moreover, similar inoculations to Tg mice expressing PrP-129M, i.e. mismatched at codon 129 with the PrP-codon 129 of the inoculum, hampered transmission of T1^21^ and delayed that of T1^20^. Combined, these data indicate that T1^21^ and T1^20^ represent two distinct human prion substrains which however are not able to determine distinct phenotypes.

**Funding**

Supported by National Institutes of Health Grants R01 NS083687 and P01 AI106705, and The Charles S. Britton Fund (to P.G.). This study was also supported by a grant from the Spanish Ministerio de Ciencia, Innovación y Universidades (AGL2016-78054-R – AEI/FEDER, UE).

### 

References[1]Parchi et al
Ann Neurol. 1999;46(2):224–233.10443888[2]Parchi et al
Acta Neuropathol. 2011;121:91–11210.1007/s00401-010-0779-621107851

## 168. Clinical prediction of type 1 and type 2 mixed histopathology distribution in MM-type sporadic Creutzfeldt-Jakob disease

Toshimasa Ikeda^a,b^, Yasushi Iwasaki^a^, Keita Sakurai^c^, Akio Akagi^a^, Maya Mimuro^a^, Hiroaki Miyahara^a^, Tetsuyuki Kitamoto^d^, Noriyuki Matsukawa^b^, Mari Yoshida^a^

^a^Department of Neuropathology, Institute for Medical Science of Aging, Aichi Medical University, Nagakute, Japan; ^b^Department of Neurology and Neuroscience, Nagoya City University Graduate School of Medical Sciences, Nagoya, Japan; ^c^Department of Radiology, Teikyo University School of Medicine, Tokyo, Japan; ^d^Department of Neurological Science, Tohoku University Graduate School of Medicine, Sendai, Japan

**CONTACT** Toshimasa Ikeda c161706@ed.nagoya-cu.ac.jp

**ABSTRACT**

**Background/Introduction**: Although the sporadic Creutzfeldt-Jakob disease (sCJD) subtypes, with their distinctive clinicopathological features, have been identified based on two types of the abnormal prion protein (PrP^Sc^), type 1 and type 2, and polymorphic codon 129, there have been cases of both PrP^Sc^ types being present. The mixed PrP^Sc^ type features, especially in MM-type CJD, have been best identified with neuropathological examination; the MM1-type involves fine vacuole-type spongiform change (FV), whereas the MM2C-type involves large confluent vacuole-type spongiform change (LCV). Presently, it is impossible to detect the concurrence of both PrP^Sc^ types before death. The purpose of this study was to clinically predict the concurrence of MM-type sCJD with another PrP^Sc^ type in the same individual.

**Materials and Methods**: We retrospectively identified seven MM-type sCJD cases with both FV and LCV among 49 sCJD cases genetico-pathologically diagnosed in our institute between 2008 and 2016. We reviewed clinical features, pathological findings, and radiological abnormalities. We also conducted a regional systemic study to associate the spongiform change pattern with hyperintensity on magnetic resonance diffusion-weighted imaging (DWI) using the signal intensity index (SII).

**Results**: The one patient with dominant LCV showed longer disease duration, later onset of typical symptoms, no periodic sharp wave complexes in electroencephalography, and negative 14–3-3 protein findings compared to the six FV-dominant patients. LCV-dominant lesions tended to show higher intensity on DWI than FV-dominant lesions in respective patients. In the regional systemic study, LCV-dominant regions showed significantly higher SII on DWI than did the FV-dominant regions.

**Conclusions**: Mixed MM-type sCJD showed clinical features correlating with the dominant pathological burden. The SII may be clinically useful for investigating the concurrence of PrP^Sc^ type 2 in cases with the typical clinical course of MM1-type sCJD.

**KEYWORDS:** Creutzfeldt-Jakob disease; histopathology; diffusion-weighted imaging (DWI); magnetic resonance imaging (MRI); spongiform change

## 169. Stable propagation of hamster prions in CRISPR/Cas9-engineered CAD5 cells

Matthew Bourkas^a,b^, Hamza Arshad^a,b^, Gerold Schmitt-Ulms^a,c^, Jason Bartz^d^ and Joel Watts^a,b^

^a^Tanz Centre for Research in Neurodegenerative Diseases, Toronto, Canada; ^b^Department of Biochemistry, University of Toronto, Toronto, Canada; ^c^Department of Laboratory Medicine and Pathobiology, University of Toronto, Toronto, Canada 4 Department of Medical Microbiology and Immunology, Creighton University, Omaha, USA

**CONTACT** Matthew Bourkas matthew.bourkas@mail.utoronto.ca

**ABSTRACT**

**Background:**Prion research has been hindered by a lack of cellular paradigms for studying the replication of prions from different species. Although hamster prions have been widely used to study prion replication in animals and within *in vitro* amplification systems, they have proven challenging to propagate in cultured cells. Since the murine catecholaminergic cell line CAD5 is uniquely susceptible to a diverse range of mouse prion strains, [1,2] we hypothesized that it might also be capable of propagating non-mouse prions.

**Materials and Methods**: We generated CAD5 cells lacking endogenous PrP^C^ (CAD5-PrP^−/-^ cells) using CRISPR/Cas9-mediated genome editing. CAD5-PrP^−/-^ cells were stably transfected with a plasmid encoding hamster PrP and then challenged with brain homogenates containing various strains of hamster prions.

**Results**: When exposed to the 263K, HY, or 139H strains, hamster PrP-expressing CAD5-PrP^−/-^ cells stably propagated high levels of protease-resistant PrP. Moreover, when these cells were challenged with 10-fold serial dilutions of 263K prions, PK-resistant PrP was obtained with dilutions up to and including 0.000002% brain homogenate. Cellular homogenates from 263K- infected cells exhibited prion seeding activity in the RT-QuIC assay and were infectious to naïve cells expressing hamster PrP. The presence of endogenous mouse PrP in hamster PrP- expressing CAD5 cells completely blocked the replication of hamster prions. Interestingly, murine N2a neuroblastoma cells ablated for endogenous PrP expression were susceptible to mouse prions, but not hamster prions upon expression of cognate PrP.

**Conclusions**: Our results demonstrate that CAD5 cells, but not N2a cells, can propagate prion strains from non-mouse species following the ablation of endogenous mouse PrP. This suggests that CAD5 cells either possess cellular factors that enhance or lack factors that restrict the diversity of strains that can be propagated. We conclude that transfected CAD5-PrP^−/-^ cells may be a useful tool for assessing the biology of prion strains and dissecting the mechanism of prion replication.

### 

References[1]Mahal et al., Proc Natl Acad Sci USA
2007;104(52):20,908–13.[2]Berry et al., Proc Natl Acad Sci USA
2013;110(44):E4160–9.10.1073/pnas.1317164110PMC381648324128760

## 170. Biochemical characterization of PrPSc species at pre-clinical stages of disease in a mouse model of prion disease (RML)

Ghazaleh Eskandari-Sedighi^a,b,^ Leonardo Cortez^a,c,^ Jing Yang^a^, Hristina Gapeshina^a^, Nathalie Daude^a^, Valerie Sim^a,c^ and David Westaway^a,b,d^

^a^Centre for prions and protein folding diseases, University of Alberta, Edmonton, Alberta, Canada; bDepartment of Biochemistry, Faculty of Medicine and Dentistry, University of Alberta, Edmonton, Alberta, Canada; cDepartment of Medicine, Division of Neurology, Faculty of Medicine and Dentistry, University of Alberta, Edmonton, Alberta, Canada; dDepartment of Medicine, Faculty of Medicine and Dentistry, University of Alberta, Edmonton, Alberta, Canada

**CONTACT** Ghazaleh Eskandari-Sedighi ghazaleh@ualberta.ca

**ABSTRACT**

**Background**: It is known that the amount of PrPSc and infectious titre in brains of infected animals can plateau at sub-clinical stages, suggesting a division of prion infectivity and toxicity phases [1]. Previous work in our lab has shown that a post-translational downregulation of PrPC could account the plateau effect [2,3]. These data also suggested that diminishing levels of PrPC (i.e. the substrate for PrPSc formation), as well as the rise in accumulated PrPSc are important determinants in the transition from sub-clinical to clinical stage. Although transcriptomic studies point at major role of inflammation in prion diseases [4], and a recent study on global metabolomics in mice points at changes in brain chemistry at a mid-stage in disease progression [5], there is still ambiguity about the causal relationship between these changes, neuronal loss and clinical manifestation of the disease.

**Material and Methods**: Using mice inoculated with the RML isolate of mouse-adapted scrape, we applied different techniques to track the aforementioned parameters and the biochemical features of the PrPSc particles. The isotropic fractionator technique [6] was used to quantify the number of neural and non-neural cells in the brain of infected animals. Asymmetric-flow field- flow fractionation (AF4), a one-phase chromatography technique that separates particles based on their size, was used to isolate the PrPSc particles from brains; size of the isolated particles was determined by dynamic light scattering and the seeding activity was evaluated by RT-QuIC. Histopathology was used to monitor spongiosis changes, PrPSc accumulation in the brain and astrocytes activation.

**Results**: Spongiosis, PrPSc accumulation in the brain and astrocyte activation were detected in brains from 90 dpi. However, significant neuronal loss was only validated at the terminal stage of the disease, ca. 150 dpi. Biochemical characterization of brain-derived PrPSc species at different timepoints is currently in progress.

**Conclusions**: Our quantitative data measuring NeuN positive nuclei concur with previous histological assessments in indicating that neuronal loss is a late event in prion diseases and thus cannot account for preclinical reductions in steady-state levels of PrPC. Yet earlier chemical changes can be detected in brain as early as 90 dpi. We aim to pinpoint the early PrPSc transition events by the FFF technique and understand the causal events that trigger clinical symptoms of the disease.

### 

References[1]Sandberg et al., Nature
2011;470:540–542.[2]Mays et al., J Clin Invest. 2014;24(2):847–858.10.1172/JCI72241PMC390462824430187[3]Mays et al., J Virology
2015;89(24):12,418–1242.[4]Vincenti et al., J Virology
2016;90(6):3003–3017.10.1128/JVI.02613-15PMC481062226719249[5]Bourgognon et al., Cell Death and Differentiation
2018;25:1408–1425.10.1038/s41418-018-0148-xPMC611328329915278[6]Herculano-Houzel, J Neurosci
2005;5(10):2518–2521.10.1523/JNEUROSCI.4526-04.2005PMC672517515758160

## 171. Efficacy of short-term exposure to sodium hypochlorite (20,000 ppm) or 4% acetic acid sodium lauryl sulfate (4% acetic SDS) to inactivate chronic wasting disease infectivity in a laboratory setting

Erin E McNulty, Amy V Nalls, Laura A Pulscher, Candace K Mathiason

Colorado State University, Fort Collins, Colorado, USA

**CONTACT** Erin E McNulty eem@colostate.edu

**ABSTRACT**

Prions are highly resistant to inactivation by conventional sterilization processes and disinfecting agents. The Center for Disease Control (CDC) recommendation for decontamination of the infectious agent associated with prions include exposure to 20,000 parts per million (ppm) sodium hypochlorite for 30–60 min, or high pressure-autoclave at 134 C for 1 h. High pressure-high temperature autoclaving is typically used to inactivate prions present on plastics, personal protective equipment and within small quantity tissues/homogenates routinely used by laboratory personnel. Laboratory benches, equipment and instruments are routinely exposed to 20,000 ppm sodium hypochlorite generated by diluting household bleach 1:2.5. Due to the corrosive nature of high sodium hypochlorite concentrations investigators have searched for effective means to inactivate prions that will result in less damage to the laboratory setting. To this end 4% acetic acid sodium lauryl sulfate (4% acetic SDS) has been incorporated into prion laboratory cleaning regimens. In this study we explore the efficacy of short-term exposure to 20,000 ppm hypochlorite or 4% acetic SDS to inactivate prion infectivity in a laboratory setting by use of amplification and bio-assays.

To emulate a more typical laboratory scenario, a film of brain homogenate (10% w/v) from naïve or chronic wasting disease (CWD)-infected deer was applied to a laboratory bench free of prion contamination. Each film was treated by mist of water, 20,000 ppm hypochlorite or 4% acetic acid and left for 30–60 s prior to cleanup. A swab was used to swipe the area post cleanup. Fluid retrieved from the swab was assessed for infectivity in TgCer5037 mouse bioassay.

We found that 0/9 mice inoculated with 20,000 ppm hypochlorite-treated and 5/6 mice inoculated with 4% acetic SDS-treated CWD inoculum demonstrated clinical disease consistent with prion infection. Development of terminal clinical disease in mice inoculated with 4% acetic SDS was prolonged by 50% as compared to no treatment controls. PrP^Sc^ deposition and amyloid seeding activity was demonstrated by immunohistochemistry and PMCA in all mice showing clinical disease. Mice inoculated with similarly treated naïve brain homogenate remained free of prion disease.

A contemporary dilutional bioassay permitted us to estimate the infectivity remaining in mice inoculated with treated inoculum that succumbed to prion infection. We demonstrate that a laboratory cleaning regimen including short-term exposure to 20,000 ppm hypochlorite results in sterilizing inactivation of CWD prion infectivity while 4% acetic SDS decreased CWD burden by 17%. This study provides findings to guide development of prion laboratory cleaning procedures.

## 172. Establishment of PrP^CWD^ extraction and detection methods in the farm soil

Kyung Je Park, Hoo Chang Park, In Soon Roh, Hyo Jin Kim, Hae-Eun Kang and Hyun Joo Sohn

Foreign Animal Disease Division, Animal and Plant Quarantine Agency, Gimcheon, Gyeongsangbuk-do, Korea

**ABSTRACT**

**Introduction**: Transmissible spongiform encephalopathy (TSE) is a fatal neurodegenerative disorder, which is so-called as prion diseases due to the causative agents (PrP^Sc^). TSEs are believed to be due to the template-directed accumulation of disease-associated prion protein, generally designated PrP^Sc^. Chronic wasting disease (CWD) is the prion disease that is known spread horizontally. CWD has confirmed last in Republic of Korea in 2016 since first outbreak of CWD in 2001. The environmental reservoirs mediate the transmission of this disease. The significant levels of infectivity have been detected in the saliva, urine, and faeces of TSE-infected animals. Soil can serve as a stable reservoir for infectious prion proteins. We found that PrP^CWD^ can be extracted and detected in CWD contaminated soil which has kept at room temperature until 4 years after 0.001 ~ 1% CWD exposure and natural CWD-affected farm soil through PBS washing and sPMCAb.

**Materials and Methods: Procedure of serial PMCAb**. CWD contaminated soil which has kept at room temperature (RT) for 1 ~ 4 year after 0.001%~1% CWD brain homogenates exposure for 4 months collected 0.14 g. The soil was collected by the same method once of year until 4 year after stop CWD exposure. We had conducted the two steps. There are two kinds of 10 times washing step and one amplification step. The washing step was detached PrP^Sc^ from contaminated soil by strong vortex with maximum rpm. We harvest supernatant every time by 10 times. As the other washing step, the Washed soil was made by washing 10 times soil using slow rotator and then harvest resuspended PBS for removing large impurity material. Last step was prion amplification step for detection of PrP^CWD^ in soil supernatant and the washed soil by sPMCAb. Normal brain homogenate (NBH) was prepared by homogenization of brains with glass dounce in 9 volumes of cold PBS with TritonX-100, 5 mM EDTA, 150 mM NaCl and 0.05% Digitonin (sigma) plus Complete mini protease inhibitors (Roche) to a final concentration of 5%(w/v) NBHs were centrifuged at 2000 g for 1 min, and supernatant removed and frozen at −70 C for use. CWD consisted of brain from natural case in Korea and was prepared as 10%(w/v) homogenate. Positive sample was diluted to a final dilution 1:1000 in NBH, with serial 3:7 dilutions in NBH. Sonication was performed with a Misonix 4000 sonicator with amplitude set to level 70, generating an average output of 160W with two teflon beads during each cycle. One round consisted of 56 cycles of 30 s of sonication followed 9 min 30 s of 37°C incubation. **Western Blotting (WB) for PrP^Sc^ detection**. The samples (20 µL) after each round of amplification were mixed with proteinase K (2 mg/ml) and incubated 37°C for 1 h. Samples were separated by SDS-PAGE and transferred onto PVDF membrane. After blocking, the membrane was incubated for 1 h with 1st antibody S1 anti rabbit serum (APQA, 1:3000) and developed with enhanced chemiluminescence detection system.

**Results**: We excluded from first to third supernatant in view of sample contamination. It was confirmed abnormal PrP amplification in all soil supernatants from fourth to tenth. From 0.01% to 1% contaminated washed soils were identified as abnormal prions. 0.001% contaminated washed soil did not show PrP specific band (Fig 1). The soil was collected by the same method once of year until 4 year after stop CWD exposure. After sPMCAb, there were no PrP^CWD^ band in from second to fourth year 0.001% washed soil. but It was confirmed that the abnormal prion was amplified in the washing supernatant which was not amplified in the washed soil. we have decided to use soil supernatant for soil testing (Fig. 2). After third rounds of amplification, PrP^Sc^ signals observed in three out of four sites from CWD positive farm playground. No signals were observed in all soil samples from four CWD negative farm (Fig. 3).

**Conclusions**: Our studies showed that PrP^CWD^ persist in 0.001% CWD contaminated soil for at least 4 year and natural CWD-affected farm soil. When cervid reintroduced into CWD outbreak farm, the strict decontamination procedures of the infectious agent should be performed in the environment of CWD-affected cervid habitat.

### 

References[1]NagookaK et al., Sensitive detection of scrapie prion protein in soil. Biochem Biophys Res Commun. 2010;397:626–630.2057065110.1016/j.bbrc.2010.06.013[2]GeorgssonG et al., Infectious agent of sheep scrapie may persisit in the environment for at least 16 years. J of Gen Virol. 2006;87:3737–3740.1709899210.1099/vir.0.82011-0[3]SaundersSE et al., Prions adhere to soil minerals and remain infectious. Plos Pathog. 2006;2:296–302.10.1371/journal.ppat.0020032PMC143598716617377[4]TamguneyG et al., Asymptomatic deer excerete infectious prions faces
Nature. 2009;529–532.10.1038/nature08289PMC318644019741608

## 173. Antisense oligonucleotides for the treatment of neurodegenerative diseases

Hien T Zhao, Holly Kordasiewicz, Dan Norris, Anne Smith, Roger Lane, C. Frank Bennett and Eric Swayze

Ionis Pharmaceuticals, Inc. Carlsbad, CA,USA

**CONTACT** Hien T Zhao hzhao@ionisph.com

**ABSTRACT**

Antisense oligonucleotides (ASOs) are emerging as a viable therapeutic approach to alter production of proteins implicated in currently untreatable neurodegenerative diseases. ASOs are single stranded nucleotides, typically 20 bases in length, that bind complementary target RNA through Watson and Crick hybridization. Depending on ASO design, hybridization can lead to selective degradation of the target RNA, alteration of RNA splicing or another highly specific post-transcriptional modification. Foundational science tools are available to facilitate transition from the bench to the clinic, as evidenced by regulatory approval of an ASO for the treatment of spinal muscular atrophy and initiation of clinical trials for ASOs in Huntington’s disease, amyotrophic lateral sclerosis and other neurological conditions. Tools that guide drug development and mitigate risk include pharmacology studies in rodents to demonstrate proof-of-concept phenotypic benefit of ASOs and biodistribution studies in larger species to characterize pharmacokinetics and pharmacodynamics of the ASO and key target-engagement markers. Additionally, PKPD models can be built using preclinical data to predict ASO effects in humans, enabling informed selection of ASO doses for clinical trials and interpretation of data collected in the clinic. Here, we describe our experiences in ASO development for neurodegenerative diseases, including proof-of-concept and biodistribution animal studies, PKPD model-building and clinical trial design and interpretation. These experiences provide a blueprint for advancement of ASO therapies designed to treat neurodegenerative diseases, including prion disease.

## 174. Developing a gene reporter system allowing real-time *in-vivo* monitoring of prion disease progression

Matthew J. Martin^a^ and Stephanie A. Booth^a,b^

^a^University of Manitoba; ^b^National Microbiology Laboratory, Public Health Agency of Canada

**CONTACT** Matthew J. Martin Matthew.martin4@canada.ca

**ABSTRACT**

**Introduction**: Prion diseases are a fatal neurodegenerative disease caused by the misfolding of the cellular prion protein, PrP^C^ into an infectious isoform, PrP^Sc^. Accumulation of this infectious isoform leads to the damage and death of neurons, and the progression of the disease. The molecular events involved in the pathogenesis of this disease are poorly understood and uncovering them has proven challenging due to the difficulties of identifying and isolating degenerating neurons for analysis. Ultimately, the aim of this project is to engineer adeno-associated viruses (AAV) to deliver genetic tools into neurons to drive the expression of reporter molecules during neurodegeneration. This would enable real-time monitoring of the disease *in vivo*, and identification of specific neurons to be isolated for further analysis.

**Materials and Methods**: AAV rh10 was used to deliver a genetic construct containing a short hairpin RNA sequence targeting the cellular prion gene to test the expression system. This virus construct was injected into newborn mice via their superficial temporal vein. This short hairpin RNA is designed to post-transcriptionally silence prion expression, inducing immunity against prions to the transduced cell. The genetic construct also expresses a red fluorescent protein to be used as a biomarker signalling transduced cells. At 7 weeks of age, the mice were infected with Rocky Mountain Laboratory prions, or mock-infected as a control. Brain, liver, lungs, spleen, and serum samples was collected at 60, 90, and 141 days post-infection. IHC is used to determine the presence of the virus in these tissues and Laser Capture Microdissection used to specifically isolate transduced cells for further analysis of their RNA and protein content.

**Results**: The distribution of the AAV rh10 throughout multiple regions of mouse brain will be described as well as the relative transduction of neurons versus microglia and astrocytes. In addition, the kinetics of the vector concentration in the brain over time will be described. This work is the basis for engineering vectors capable of targeting specific neurons in mouse brain tissue in future studies to understand prion pathogenesis and prion strains *in vivo*.

## 175. Environmental pathways of CWD prions

Alsu Kuznetsova^a^, Debbie McKenzie^b^ and Judd M. Aiken^a^

^a^Agricultural Life and Environmental Sciences Faculty, University of Alberta, Edmonton, Canada; ^b^Faculty of Science, University of Alberta, Edmonton, Canada

**CONTACT** Alsu Kuznetsov alsu@ualberta.ca

**ABSTRACT**

Soil can serve as a reservoir and route for horizontal transmission of chronic wasting disease (CWD) by interaction with the infectious prion protein (PrPCWD) shed by infected animals. Prion fate in environment is determined by structure of ecosystems including soil and vegetation types. In boreal ecosystems, lichen and shrubs cover sandy textured, quartz-illite soils with surface plant litter horizon. In prairie ecosystems, grassy meadows grow on humus-enriched, loamy or clay montmorillonite soils. Once shed onto the soil surface, prion persistence in the environment primarily depends on the binding and transportation capacity of soils. Factors responsible for prion migration are soil properties such as mineralogical composition, texture, soil organic matter content and pH. We investigated PrPCWD migration in soil profiles by comparing their passage through pure soil minerals and diverse soils using lab-scale soil columns. PrP-res was detected by western blot or protein misfolding cyclic amplification in leachates of columns containing quartz, surface plant litter – soil organic horizon (LFH) of boreal Luvisolic and Brunisolic soils, illite, or the mineral horizons of Luvisol and Brunisol. Infectivity in leachates from quartz, illite and Luvisolic columns was confirmed by bioassay. Analysis of the solid phase of the columns confirmed the migration of PrPCWD to lower layers in the illite, while the PrPCWD in the montmorillonite column remained close to the column surface. While mineralogical composition of soils determines movement of prions, other constituents, such as soil organic matter, microbial population, composition of soil solutions, might affect prion infectivity. To investigate the impact of humic acids (a major component of soil organic material), we incubated CWD agent with humic acids for various lengths of time. Analysis by western blot and bioassay demonstrated that humic acids can reduce PrPres and infectivity over time. Thus, in soils of temperate regions, including the Chernozems of Northern America and Cambisols of Europe, prions would remain at the soil surface due to strong binding to montmorillonite, while in boreal and tundra regions soils (Luvisols, Podzols, and Brunisols), most of the prions would be transported through the plant litter LFH, and partially through the upper soil mineral horizon into lower horizons. Although these soils also have lower levels of humic acids, a soil organic matter compound that has the capacity to reduce CWD infectivity levels, soils of the boreal and tundra regions are less favourable to the horizontal transmission of CWD as these soils make PrPCWD less bioavailable.

## 176. Ultrasensitive detection and seeding selectivity of tau aggregates of Alzheimer disease and chronic traumatic encephalopathy

Allison Kraus^a^, Eri Saijo^a^, Michael M. Metrick^a^, Kathy Newell^b^, Christina J. Sigurdson^c^, Gianluigi Zanusso^d^, Bernardino Ghetti^e^ and Byron Caughey^a^

^a^LPVD, Rocky Mountain Laboratories, NIAID, NIH, Hamilton, MT, USA; ^b^University of Kansas School of Medicine, Kansas City, KS, USA; ^c^Department of Pathology, UC San Diego, La Jolla, CA, USA; ^d^University of Verona, Verona, Italy; ^e^Indiana University School of Medicine, Indianapolis, IN, USA

**CONTACT** Allison Kraus allison.kraus@nih.gov

**ABSTRACT**

In Alzheimer disease (AD) and chronic traumatic encephalopathy (CTE) approximately equivalent amounts of 3-repeat (3R) and 4-repeat (4R) tau isoforms accumulate in tau filaments in the brain. To investigate the basis for AD 3R/4R tau filament propagation and to improve detection of 3R/4R tau aggregates as potential biomarkers, we have exploited the growth mechanism of tau filaments to develop a highly selective and ultrasensitive cell-free tau seed amplification assay optimized for AD (AD real-time quaking induced conversion or AD RT-QuIC). This reaction is based on the capacity of AD tau aggregates to seed formation of amyloids made of certain tau fragments. Seeding activity was detected in AD brains (n = 16) at dilutions as extreme as 10^7^–10^10^-fold but was 10^2^–10^6^-fold less responsive when seeded with brain from most cases of other types of tauopathy with roughly comparable loads of tau aggregates composed predominantly of either 3R or 4R tau isoforms. AD brains had average seeding activities that were orders of magnitude higher than Pick disease brains with predominant 3R tau deposits; however, we previously observed the opposite using our prototypic Pick- optimized tau RT-QuIC assay. CTE brains (n = 2) had seed concentrations comparable to some of the AD specimens, and higher than three of four specimens with 3R/4R primary age-related tauopathy (i.e., PART). AD seeds shared properties with the neurofibrillary tangles found in AD brains in terms of being sarkosyl-insoluble, protease-resistant, and reactive with tau antibodies.

Moreover, the AD RT-QuIC assay detected as little as 16 fg of pure synthetic tau fibrils. The distinctive seeding activity shown by AD and CTE tau filaments in comparison to those of other types of tauopathies suggests a templating specificity that may underpin their consistent strain- like propagation as conformers in patients with 3R/4R tau diseases. Importantly, AD RT-QuIC also provides a means of rapid and ultrasensitive quantitation of 3R/4R tau seeding activity as a biomarker. We are currently adapting the assay to the detection of AD and CTE tau seeds in biologically accessible specimens to aid in diagnostics and therapeutics development.

**KEYWORDS:** Alzheimer disease; chronic traumatic encephalopathy; tau; seed; diagnostics; biomarkers; RT-QuIC

### 

Reference[1]KrausA et al., NeuropathActa
2018:[Epub ahead of print].

## 177. Assessing the importance of Tau mislocalization to the dendrites in Alzheimer’s disease

Emily A. McNamara, Sue-Ann Mok

Department of Biochemistry, University of Alberta, Edmonton, Canada

**CONTACT** Emily A. McNamara emcnamar@ualberta.ca

**ABSTRACT**

Alzheimer’s disease is a fatal neurodegenerative condition primarily characterized by a decline in cognitive function and a loss of motor skills^1^. This disease affects cells of the central nervous system (neurons) and is the leading cause of all dementias^1^. While the exact cause of Alzheimer’s disease is not yet fully understood, one of the main components thought to be associated with disease pathology is the abnormal accumulation of the protein, Tau^2^. Tau is a microtubule-associated protein found primarily in the neuronal axon, where it plays a role in assembling microtubules that are critical for axonal transport^3^. Studies have shown that Tau mislocalization to the dendritic spines can occur during or preceding a disease state, causing synaptic disruption and overall brain dysfunction^4^. Specific signals in the 3ʹ Untranslated Region (UTR) of Tau mRNA transcripts have been shown to play a key role in subcellular mRNA localization and therefore local protein translation^5^.

Our work aims to modify the human Tau 3ʹ UTR in order to localize it to the neuronal axon or dendritic spines. Following this, we will be able to explore Tau’s interactions with various cellular components at either subcellular location and potentially uncover key components involved in disease pathology.

We have designed constructs of the Tau 0N4R isoform with a modified 3ʹ UTR. The 3ʹ UTR’s utilized are derived from human Tau or mouse Tau, which localize to the axon, or another microtubule associated protein, MAP2, which localizes to the dendrites. While it has been shown that mouse Tau 3ʹ UTR is capable of directing Tau to the axons^5^, this has not yet been replicated with human Tau. The constructs will be transfected into human neuronal cells using lentiviral vectors. Fluorescent antibodies and mRNA probes recognizing specific regions of exogenously-expressed Tau will be used to detect the correct localization of the protein and transcript. While the analysis of Tau interactions at both the axon and dendrites are part of my long-term project, HEK cell lines that express Tau 0N4R have been transfected with DNAJ2 and Hsc70; two chaperones known to be upregulated during Alzheimer’s Disease. HEK cells will be induced to express Tau, and potential aggregation will be monitored using confocal microscopy. Results from this assay may pinpoint DNAJ2 and Hsc70 as key players in Tau aggregation, and results will be replicated in a human neuronal model.

Understanding the importance of Tau localization in neurodegenerative disease pathology may uncover potential key therapeutic targets for the treatment of Alzheimer’s disease.

**KEYWORDS:** Alzheimer’s disease; Tau; neurodegeneration; 3ʹ UTR

### 

References[1]Alzheimer’s Association
2015
Alzheimer’s Disease facts and figures. Alzheimer’s & Dementia. 2015;11(2015):332–384.10.1016/j.jalz.2015.02.00325984581[2]MusiN., ValentineJM. et al., Tau protein aggregation is associated with cellular senescence in the brain. Wiley Aging Cell. 2018:1–13. DOI:10.1111/acel.12,840PMC626091530126037[3]BuéeL. et al., Tau protein isofroms, phosphorylation and role in neurodegenerative disorders. Brain Research Reviews. 2000;33:95–130.1096735510.1016/s0165-0173(00)00019-9[4]HooverBR. et al., Tau mislocalization to dendritic spines mediates synaptic dysfunction independently of neurodegeneration. Cell Press Neuron. 2010;68:1067–1081.10.1016/j.neuron.2010.11.030PMC302645821172610[5]AronovS. et al., Axonal Tau mRNA localization coincides with tau protein in living neuronal cells and depends on axonal targeting signal. The Journal of Neuroscience. 2001;21(17):6577–6587.1151724710.1523/JNEUROSCI.21-17-06577.2001PMC6763080

## 178. Distinct strain of Amyloid beta and pathogenic tau protein in iatrogenic Creutzfeldt-Jakob disease

Jiri G. Safar^a^, Chae Kim^a^, Tracy Haldiman^a^, Anushruti Ashok^a^, Ignazio Cali^a^, Mark Cohen^a^, Stéphane Haїk^b^, Piero Parchi^c^, Giorgio Giaccone^d^, Steven Collins^e^, Nicolas Privat^b^, Véronique Sazdovitch^b^, Charles Duyckaerts^b^, Diane Kofskey^a^, Han Wang^a^, Catriona McLean^e^, Jean-Philippe Brandel^b^, Tetsuyuki Kitamoto^f^, Ermias Belay^g^, Ryan Maddox^h^, Fabrizio Tagliavini^d^, Maurizio Pocchiar^i^, Brian Appleby^a^, Pierluigi Gambetti^j^, Ele Gelpi^k^, Ann McKee^l^, James Ironside^m^ and Lawrence Schonberger^n^

^a^Case Western Reserve University; ^b^Sorbonne Universités; ^c^University of Bologna; ^d^Istituto Neurologico Carlo Besta; ^e^University of Melbourne; ^f^Tohoku University; ^g^Center for Disease Control and Prevention; ^h^CDC; ^i^Istituto Superiore di Sanità; ^j^Department of Pathology, Case Western Reserve University, School of Medicine, Cleveland, OH, USA; ^k^Hospital Clínic Institut d’Investigacions Biomèdiques August Pi i Sunyer; ^l^Boston University; ^m^University of Edinburgh; ^n^US Centre for Disease Control and Prevention

**ABSTRACT**

**Objectives**: Whether some strains of prion-like protein aggregates (prionoids) such as amyloid beta (Aβ) and tau propagating in cell culture and transgenic experiments can be infectious and transmit the neurodegenerative diseases from man-to-man is unknown. We reported recently an increased frequency of amyloid β (Aβ) plaques and angiopathy in a cohort of 27 iatrogenic cases of Creutzfeldt-Jakob disease (iCJD), acquired from the prion-contaminated growth hormone (GH) extracted from human cadaveric pituitary glands, or from cadaveric dura mater grafts (DM) during neurosurgery. As in human prion diseases, critical questions raised by these findings are (a) whether the pathology was transmitted by a unique strain of Aβ or tau with a high potential to initiate or accelerate Alzheimer’s disease (AD), and (b) if such Aβ or tau strains can be identified whithin the spectrum of AD.

**Methods**: With new biophysical and structural tools, we analysed conformational strain characteristics of Aβ and pathogenic tau aggregates in 14 cases of iCJD, and compared the data with 21 cases of age-matched sporadic CJD (sCJD), 30 cases of Alzheimer’s diseases with rapid progression (rpAD), 18 cases with slow AD progression (spAD), 7 cases of chronic traumatic encephalopathy (CTE), and 8 cases of frontotemporal lobar degeneration (FTLD).

**Results**: The levels of oligomeric and aggregated Aβ42 as well as the Aβ42/Aβ40 ratios were in iCJD cases elevated signficantly above the levels found in sCJD controls. The conformational characteristics of Aβ42 acccumulating in iCJD were distinctly different from sCJD and reminiscent of small oligomers of Aβ42 found most frequently in AD with rapid progression. The levels of detergent-insoluble pathogenic tau aggregates were increased in ~14% of iCJD above the levels found in sCJD but were generally lower than in AD and other tauopathies. Notably, the conformations of detergent-insoluble tau found in hippocampus of iCJD cases were significantly different from those found in sCJD.

**Conclusions**: The levels of oligomeric and aggregated forms of Ab42 and pathogenic aggregates of tau protein are in iCJD cases significantly higher than in sCJD controls and their conformational characteristics are in the range corresponding to the strains found most frequently in rapidly progressive AD. Although the contributions of other factors, including GH deficiency cannot be discounted, our findings argue that this AD pathology was acquired by a distinct strain of amyloid beta conformers through a mechanism resembling that of prion diseases.

## 179. PrP^CWD^ detection in CWD-infected TgElk mice model using RT-QUIC

Hyun Joo Sohn, Kyung Je Park, In Soon Roh, Hyo Jin Kim, Hae Eun Kang

Foreign animal disease division, Animal and Plant Quarantine Agency, Gimcheon, Gyeongsangbuk-do, Korea

**ABSTRACT**

**Introduction**: Chronic wasting disease (CWD) is the only prion disease affecting free-ranging animals, reported in North America, South Korea, and Norway. Unlike in most other prion disease CWD agents are shed in blood, urine, and faeces which most likely contribute to the horizontal transmission between cervid species. The developments of amplification-based seeding assays have been instrumental in the detection of low levels of prions in clinical samples. Using real-time quaking-induced conversion (RT-QUIC), we established an ultrasensitive detection method for PrP^CWD^ in the urine from CWD-infected sequentially sampled transgenic mice overexpressing elk prion protein (TgElk mice). In addtion, RT-QUIC was performed in the kidney and brain of theses mice model to trace abnormal prion.

**Materials and Methods**: 44 brain and kidney, urine samples from sequentially collected from CWD-infected TgElk mice (TgElk CWD) were stored at −80°C. In brain and kidney, 10% (w/v) homogenate was prepared in 0.9% sterilized saline. In urine 100 µL of each sample was mixed with 10 µL 2.8% sodium phosphotungustic acid (NaPTA) and incubated for 1hr at 37°C with shaking at 1,350 rpm. Samples were centrifuged for 30 min at 16,100 g. The pellet was resuspended in 10 µL of 0.1% SDS/PBS for 30 min at 55°C. RT-QUIC reactions were set up in 96-well clear bottom optic plates and consisted of 98 µL RT-QUIC buffer [final concentrations of 1XPBS, 1 mM EDTA, 10 µM Thioflavin, 300 mM NaCl buffer and 0.1 mg/ml recombinant Syrian hamster recombinant protein (23–231), and 2 µL of sample. The RT-QUIC assay was performed on a FLUOstar Omega fluorescence plate reader that was preheated to 42°C for 60 h with 90 s shaking at 700 rpm followed by 1 min incubation.

**Results**: Five randomly selected mice were sequentially culled on every 15 days from 30dpi to 120dpi during CWD infected TgElk mice reached terminal stage. Rough hair coats among clinical signs were showed from 90 dpi. PrP^CWD^ in the brain in TgElk CWD was detectable persistently from early stages (30dpi), and in the kidney PrP^CWD^ was also detectable in clinical and terminal stages (90 dpi and 120dpi). PrP^CWD^ in the urine in TgElk CWD reached the highest levels at 120dpi. NaPTA/RT-QUIC was applied to measure PrP^CWD^ in urine samples collected on every 15 days from 30dpi to 120dpi when CWD infected TgElk mice reached terminal stage. PrP^CWD^ in the urine in TgElk CWD reached the highest levels at 90dpi. PrP^CWD^ was also detectable in late and terminal stages (120dpi).

**Conclusions**: We demonstrate that CWD prions can be detected by RT-QUIC or NaPTA/RT-QUIC in the brain, kidney and urine of TgElk mice at the early and terminal stages of disease. Based on these data, we suggest that PrP^CWD^ is excreted into only urine until 90 dpi and then slowly accumulated in kidney. Our results can be used in designing future study of CWD pathogenesis in TgElk mice.

### 

References[1]HendersonDM et al., Rapid antemortem detection of CWD prions in deer saliva. PLOS one. 2013:e74377
1–1210.1371/journal.pone.0074377PMC377061124040235[2]NagookaK, YoshikaM, ShimozakiN. et al., Sensitive detection of scrapie prion protein in soil. Biochem Biophys Res Commun. 2010;397:626–630.2057065110.1016/j.bbrc.2010.06.013

## 180. Cell assay detection of chronic wasting disease (CWD) prion infectivity

Su Bi Ahn^a^, Hyo-Jin Kim^a^, Jifeng Bian^b^, In-Soon Roh^a^, Hae-Eun Kang^a^, Hyun-Joo Sohn^a^

^a^Foreign Animal Disease Division, Animal and Plant Quarantine Agency, Gimcheon, Gyeongsangbukdo, Korea; ^b^Prion Research Center (PRC), Colorado State University, Fort Collins, CO, USA

**CONTACT** Hyun-Joo Sohn

**ABSTRACT**

Chronic wasting disease (CWD) is a prion disease affecting the animals in family Cervidae. Prions are the infectious agents that cause prion disease. Mouse prion bioassay is the most commonly used method for determine prion infectivity. Application of more efficient measurement method was required as several cases of CWD have been reported in South Korea. In this study, we adapted the modified cervid prion cell assay (CPCA) using Elk21^−^ cells. The susceptible Elk21^−^ cells were exposed to various CWD brain homogenates, ranging from 10^−2^ to 10^−5^. The CPCA titers of elk CWD brain homogenates were 10^5.8^ [2001(E190Y+229Y)] and 10^6.5^ [16FC050] units/g of brain, respectively. We also found the CWD prion titer of red and sika deer brain inocula in 2016 CWD cases, producing CPCA titers of 10^5.8^ and 10^5.4^ units/g of brain. These results were confirmed by western blotting. Comparing mouse titers of mouse ic LD50/g of cervid brain with CPCA titers, sensitivity of the cell-based measurement was similar to that of the mouse bioassay. Also, CPCA titers does not depend on CWD species, relate with mouse bioassay survival time. Consequently, our results demonstrate that CWD prion infectivity can be efficiently and quantitatively detected by cell based titration of CWD.

## 181. Application of the TSE-infected cell and RT-QUIC assay to discover novel natural products

Hyo-Jin Kim, Su Bi Ahn, In-Soon Roh, Kyung-Je Park, Hae-Eun Kang, Hyun-Joo Sohn

Foreign Animal Disease Division, Animal and Plant Quarantine Agency, Gimcheon, Gyeongsangbukdo, Korea

**CONTACT** Hyun-Joo Sohn

**ABSTRACT**

Conformational conversion of the normal prion protein (PrP^C^) into an infectious PrP^Sc^ causes pathogenesis in prion diseases or transmissible spongiform encephalopathies (TSEs). TSE-infected cell lines have been efficiently used to study this conversion process, as well as to screen for potentially effective anti-prion compounds. We employed fragment molecular orbital (FMO)-based virtual screening and BSE-infected cell-based assay system to discover two natural products with antiprion activity exhibited good binding interactions, with hotspot residues within the PrP^C^ binding site, and effectively reduced PrP^Sc^ levels in a standard scrapie cell assay (SSCA). We also investigated the prion inhibition ability of two novel natural products in Elk 21^+^ (CWD) or SMB (Scrapie) cells. Cytotoxicity tests showed no significant cell death in either BNP-03 or BNP-08 treatment. Elk21^+^ and SMB cells were subjected to two effective natural products for reducing PrP^Sc^, BNP-03, and BNP-08. Efficiency of PrP^Sc^ clearance for two natural products was estimated by evaluating the signal sensitivity for PrP^Sc^ on SSCA, WB, and EIA. Antiprion activity of the compounds was detected by RT-QUIC assay seeded with TgElk CWD (E190Y+229Y). These natural products showed the inhibitory effects in the TSE-infected cells. Further experiments in animal models will be necessary to confirm the effect of two novel natural products *in vivo*.

## 182. Species and sex differences in contact during the breeding season may explain variation in Chronic Wasting Disease prevalence

Kelsey Saboraki and Susan Lingle

Department of Biology, University of Winnipeg

**CONTACT** Kelsey Saboraki ksaboraki@gmail.com

**ABSTRACT**

**Background**: Chronic Wasting Disease (CWD) is a transmissible and fatal neurodegenerative prion disease that threatens wild populations of ungulates. Prevalence of CWD is higher in mule deer than in white-tailed deer and in males than in females of both species. Prions are spread through direct and indirect contact with saliva, urine, faeces and to an unknown extent, glandular secretions. Species and sex differences in rates of direct contact with infected individuals or indirect contact with environmental contaminants may result in species and sex differences in pathogen transmission and disease prevalence. Social behaviours and the potential for pathogen transmission change seasonally, and interactions during the breeding season may explain differences in prevalence. We therefore tested the hypothesis that species and sex differences in behaviour during the breeding season underlie species and sex differences in CWD prevalence in mule and white-tailed deer.

**Methods**: We observed behavioural interactions between deer during the breeding season on an open grassland site, the McIntyre Ranch, in southern Alberta to assess whether species and sex differences in direct physical contact (deer to deer) and indirect contact (deer to environment to deer) correspond to the higher prevalence of CWD in mule deer and in males. We observed 86 deer (21 mule deer females, 21 mule deer males, 20 white-tailed females, and 24 white-tailed males) and recorded the occurrence and duration of deer-deer and deer-environment-deer contact and the number of partners with which deer engaged in contact with over time.

**Results and Discussion**: We found that males exhibit sex-specific behaviours, such as marking vegetation and flehmen, that have the potential to elevate their risk of prion transmission relative to females. During courtship interactions, between sex contact was more common in mule deer than in white-tailed deer, which could explain species differences in prevalence. As females are in estrous for only 1–2 days while a successful male engages in high-risk courtship throughout the rut, these results show that males of both species should have a higher cumulative potential for prion transmission throughout the breeding season. By collecting empirical data on the fine-scale behaviour of sympatric mule deer and white-tailed deer during the breeding season, we identified mechanisms that may be responsible for the higher prevalence of CWD in mule deer and in males.

**KEYWORDS:** Chronic wasting disease; mule deer; white-tailed deer; behaviour; transmission

## 183. Morphometric analysis of PrP deposition in two models of prion diseases

Pawel P. Liberski0000-0001-6507-4682^a^, Agata Gajos0000-0002-3753-6197^b^, Beata Sikorska0000-0002-6166-0729^a^ and Janusz Moryś0000-0002-4048-1721^c^

^a^Laboratory of Electron Microscopy and Neuropathology, Department of Molecular Pathology and Neuropathology, Chair of Oncology, Medical University Lodz, Poland; ^b^Department of Extrapyramidal Diseases, Chair of Rehabilitation, Medical University of Lodz, Lodz, Poland; ^c^Department of Anatomy and Neurobiology, Medical University of Gdansk, Gdansk, Poland

**CONTACT** Pawel P. Liberski

**ABSTRACT**

The Echigo-1 strain of Creutzfeldt-Jakob disease (CJD) was isolated from a case of a 33-years-old female with a panencephalopathic type of CJD. The Fujisaki (Fukuoka 1) was isolated from a case of Gerstmann-Straussler-Scheinker disease (GSS)

**Material and Methods**: The morphometrical analysis was done using the Zen Blue v. 2.6 and its automated analysis module for calculation of percentage of total area of all immunopositive objects in relation to the area of the measurement frame as well as the mean intensity of grey values of an analysed region and the pathological changes.

**Results**: The significant differences in the distribution of pathological changes were observed in the studied two groups of animals. In the Echigo-1 animals the most intense changes were observed in the lateral dorsal nucleus (49.8% of studied area was occupied by the pathological protein) and ventrolateral nucleus (20.3%) of the thalamus, auditory (37.8%) and somatosensory (32.9%) cortices and the hilus (22.4%), and CA2 (28.6%) sector of the hippocampus. The smallest changes were present in the dentate gyrus (1.4%) and putamen (5.1%). The highest reaction of the microglia was observed in the somatosensory cortex, lateral dorsal nucleus, and in the hippocampal sectors. Very weak reaction of the microglia was present in the dentate hilus, putamen, and ventrolateral thalamus.

In the second group of animals the most dramatic changes were observed in the thalamus (ventrolateral nucleus – 56.2%; lateral dorsal nucleus – 50.3%, and lateral posterior nucleus – 40.7%) as well as in the hippocampus (CA1 – 48.1%, CA3 – 40.3%, and CA2 – 35.6). The moderate reaction was present the somatosensory and auditory cortex, basolateral amygdala, and putamen. The weak reaction was observed in the dentate gyrus and endopiriform nucleus. The intensive reaction of microglia was present in the somatosensory cortex, in the thalamus and basolateral amygdala. The small reactivity of the microglia was observed in the dentate gyrus and its hilus as well as in the CA1 sector. The microglia reactivity was almost twice bigger in the present in the animals injected by the Fujisaki strain than Echigo-1, also the intensity of immunoreactivity for prion protein distribution was biggest in the first group of animals.

**Funding**

PPL and BS are supported by National Science Centre Poland, grant number UMO-2015/19/B/NZ4/03234

## 184. Complex pathways for native folding and misfolding of SOD1 observed directly by single-molecule force spectroscopy

Supratik Sen Mojumdar, Craig R. Garen and Michael T. Woodside

University of Alberta

**CONTACT** Michael T. Woodside

**ABSTRACT**

Superoxide dismutase 1 (SOD1) is a β-barrel dimer that undergoes prion-like misfolding in the context of ALS, but its misfolding mechanism remains unclear. We studied the folding of SOD1 monomers and dimers by using laser tweezers to unfold and refold single molecules held under mechanical tension. Monomers were found to fold via numerous intermediate states, in contrast to the two-state folding deduced from ensemble studies. A novel pathway analysis indicated that these intermediates involve the addition of individual β-strands to the native structure. Several misfolded states were also observed, branching off from the native pathway after the formation of a stable native-like core [Sen Mojumdar et al., Nat. Commun. 2017]. In dimers, the interface between domains changed the folding noticeably, in several ways. First, the number of intermediates was lower than expected for independent monomers, indicating increased cooperativity. Second, the dimer interface was found to modulate the folding pathways by changing the relative stability of the monomer segments close to the interface. Third, the dimer misfolded only half as much as expected from the monomer misfolding rate, suggesting the interface helps protect against misfolding, in part by reducing the prevalence of partially-folded intermediates that lead to misfolding. Finally, inserting the point mutation G85R, which is associated with familial ALS, vastly increased the rate of misfolding.

## 185. Probing cortico-cortical and hippocampal-cortical interactions in a second-generation mouse model of Alzheimer’s disease

Surjeet Singh, Jogender Mehla, Robert J. Sutherland and Majid Mohajerani

Department of Neuroscience, Canadian Center for Behavioral Neuroscience, University of Lethbridge, Alberta, Canada

**CONTACT** Surjeet Singh

**ABSTRACT**

It is known that Alzheimer’s disease (AD) is associated with defects of synaptic connectivity. Such defects may not be restricted to local neuronal interactions but may extend to long-range brain activities, such as slow-wave oscillations that are particularly prominent during non–rapid eye movement (non-REM) sleep and are important for integration of information across distant brain regions involved in memory consolidation. Using a newly characterized knock-in mouse model of AD (n = 8, 12 months old) that has humanized amyloid beta (Aß) sequence knocked-in to the murine amyloid precursor protein gene along with the Swedish, Arctic, and Beyreuther/Iberian mutations (APP NL-G-F), and wide-field optical imaging of cortical voltage responses (VSDI) combined with local field potential (LFP) recording from CA1, we investigated that how Aß deposition in these mouse model impacts cortical functional connectivity and how different cortical regions interact with hippocampus during slow wave sleep. We found that Aß deposition in knock-in mouse model of AD (APP NL-G-F) impairs cortical functional connectivity and intrinsic hippocampal circuit that generates SWR. Coordination of cortical up-states and hippocampal sharp-wave ripples (HPC-SWR) during slow wave sleep is believed to play a major role in the consolidation of recently acquired memories. It was observed that HPC-SWR show strong correlation with cortical activation in retrosplenial cortex (RSC). However, the magnitude of ripple power and cortical activation in RSC is reduced in AD mice as compared littermate controls, suggesting impairment in hippocampal-cortical interaction during slow wave sleep. We further analysed cortical VSDI data form 19 region of interests (ROIs) with two nonlinear methods: entropy (En) and auto mutual information (AMI). En quantifies regularity in data, while AMI detects linear and nonlinear dependencies in time series. We observed that En was lower in AD mice, and AMI decreased more slowly with time delays in AD as compared to control mice, for both En and AMI significant differences were observed in occipital, somatosensory and motor cortex (*p* <0.05). These results provide new insights into brain dysfunction associated with Aß deposition in APP NL-G-F mouse model of AD.

## 186. Serial detection of hematogenous prions in CWD-infected deer

Amy V. Nalls, Erin E. McNulty, Nathaniel D. Denkers, Edward A. Hoover and Candace K. Mathiason

Department of Microbiology, Immunology, and Pathology, Colorado State University, Fort Collins, CO, USA

**CONTACT** Amy V. Nalls amy.nalls@colostate.edu

**ABSTRACT**

Blood contains the infectious agent associated with prion disease affecting several mammalian species, including humans, cervids, sheep, and cattle. It has been confirmed that sufficient prion agent is present in the blood of both symptomatic and asymptomatic carriers to initiate the amyloid templating and accumulation process that results in this fatal neurodegenerative disease. Yet, to date, the ability to detect blood-borne prions by *in vitro* methods remains difficult.

We have capitalized on blood samples collected from longitudinal chronic wasting disease (CWD) studies in the native white-tailed deer host to examine hematogenous prion load in blood collected minutes, days, weeks and months post exposure. Our work has focused on refinement of the amplification methods RT-QuIC and PMCA. We demonstrate enhanced *in vitro* detection of amyloid seeding activity (prions) in blood cell fractions harvested from deer orally-exposed to 300 ng CWD positive brain or saliva.

These findings permit assessment of the role hematogenous prions play in the pathogenesis of CWD and provide tools to assess the same for prion diseases of other mammalian species.

## 187. Impact of new Creutzfeldt-Jakob disease mouse models in prion research

Juan Maria Torres^a^, Juan Carlos Espinosa^a^, Olivier Andreoletti^b^, Isidre Ferrer^c^, Inga Zerr^d^, Franc Llorens^c,d^

^a^Centro de Investigación en Sanidad Animal-INIA, Madrid, Spain; ^b^French National Institute for Agricultural Research (INRA)-ENVT UMR 1225, Toulouse, France; ^c^Bellvitge Biomedical Research Institute, Barcelona, Spain and Network Center for Biomedical Research of Neurodegenerative Diseases, Institute Carlos III, Ministry of Health, Spain; ^d^Department of Neurology, University Medical School, Göttingen, Germany

**CONTACT** Franc Llorens franc.llorens@gmail.com

**ABSTRACT**

Studying animal prion diseases models is an appealing approach to dissect the temporal-dependent physiopathology of human prion diseases. In the recent years, new mouse models have been developed based on the genetic deletion of the endogenous mouse prion protein gene (PrnP 0/0) and the overexpression of the human prion protein (*PRNP*). After their inoculation with human brain homogenates, the animals develop a prion pathology, which highly recapitulates the neuropathological and biochemical features of human prion diseases in a subtype-dependent manner.

These humanized mouse models have shown to be crucial for the understanding of the genetic susceptibility to prions, the transmissibility properties of different prion subtypes and the zoonotic potential of prion strains. However, the precise molecular and signalling features occurring during the progression of disease pathology in these models have not been yet scrutinized in detail.

We used the tg340-MM mouse model (transgenic mice overexpressing the *PRNP* with Met/Met at its codon 129 inoculated with sCJD MM1 brain homogenate) as a model of sCJD subtype MM1 in order to gain insight into the molecular mechanism driving to human prion pathogenesis. Targeted and screening studies were carried out in a temporal and regional-dependent fashion and main outcomes were systematically compared to those obtained in sCJD MM1 post-mortem cases.

In the tg340-MM mice, we detected temporal and regional dependent alterations in neuro-inflammatory profiles, miRNA, gene expression signatures, prion protein deposition patterns and pathogenic-associated cell signalling pathways that were highly similar than those detected in sCJD MM1 cases. Additionally, the study of the tg340-MM mice allowed us to profile the molecular signatures at pre-clinical and early-clinical stages of the disease.

Altogether, we proved the usefulness of new transgenic humanized CJD models as a valid tool for the study of the molecular features associated with human prion diseases.

## 188. Full atomistic model of PrPSc structure and conversion

Giovanni Spagnolli^a^, Marta Rigoli^a^, Simone Orioli^a^,^b^, Alejandro M Sevillano^c^, Pietro Faccioli^a^,^b^, Holger Wille^d^, Emiliano Biasini^a^, Jesus R Requena^e^

^a^University of Trento, Italy; ^b^Italian Institute for Nuclear Physics (INFN) - TIFPA, Italy; ^c^University of California San Diego, USA; ^d^University of Alberta, Canada; ^e^University of Santiago de Compostela, Spain

**CONTACT** Giovanni Spagnolli giovanni.spagnolli@unitn.it

**ABSTRACT**

Since their discovery, prions have stood as enigmatic agents that defy the classical concept of genetic inheritance due to the capacity of propagating their conformationally encoded information in absence of nucleic acid [1].However, after more than 30 years, the high resolution structure of PrPSc, required to understand the molecular features of prion infectivity, has remained elusive [2].Different PrPSc models have been proposed but they are now inconsistent with recent results [2].In this work, using molecular modelling techniques, we constructed an all-atom model of mouse PrPSc, derived from the integration of a wide array of experimental constraints, such as: (i) cryo-EM and X-ray fiber-diffraction studies, which showed that the fold of a mouse infectious PrP is compatible with a 4-rung-β-solenoid (4RβS) architecture [3,4];(ii) circular dichroism and FTIR spectroscopy, which ruled out the presence of α-helices [5];(iii) mass spectrometry analyses, indicating the presence of an intact disulphide bond between residues C178 and C213 [6],as well as mapping Proteinase K sensitive residues, which reflect amino acids likely excluded from the resistant core of the protein [7];and (iv) the possibility of accommodating complex glycans at positions N180 and N196 [8]. The stability of this new PrP_Sc_ model, assessed by Molecular Dynamics simulations, was found to be comparable to that of the prion forming domain of Het-s, a naturally-occurring β-solenoid protein. Finally, we coupled the information of the 4RβS structure with Ratchet and Pawl Molecular Dynamics [9],to perform for the first time a simulation of the conformational transition from PrPC to PrP_Sc_. The result is a transition pathway with atomistic resolution in which the C-terminal rung of the solenoid acts as a primary conversion surface for PrPC unstructured N-terminus, followed by a cascade of conformational changes where each newly formed rung templates the formation of the following one, ultimately leading to the complete conversion of PrPC into PrP_Sc_. This structure is, to the best of our knowledge, the first physically plausible PrP_Sc_ model, and represents a unique workbench for interpreting future structural data or available biological evidence, such as the effects of variations in the *PRNP* gene favoring or disfavoring prion propagation. Furthermore, it allowed us to reconstruct how the information encoded into the conformation of a protein could be propagated in a directional fashion: a concept underlying the infectious nature of prions.

**KEYWORDS:** PrP_Sc_ Structure; Prion Propagation; Molecular Modelling; Molecular Dynamics

### 

References[1]Prusiner SB. Science
1982;216(4542):136-144.[2]Wille H, Requena JR. Pathogens
2018;7(1):20.[3]Vazquez Fernandez E, et al. PLoS Pathog
2016;12(9):e1005835.[4]Wille H, et al. Proc Natl Acad Sci USA
2009;106(40):16990-16995.[5]Requena JR, Wille H. Prion
2014;8(1):60-66.10.4161/pri.28368PMC703090624583975[6]Welker E, et al. J Biol Chem
2002;277(36):33477-33481.[7]Vazquez Fernandez E, et al. PLoS Pathog
2012;14(1):e1006797.[8]Baskakov IV, Katorcha E. Front Neurosci
2016;10:358.10.3389/fnins.2016.00358PMC497611127551257[9]Tiana G, Camilloni C. J Chem Phys
2012;137(23):235101.10.1063/1.476908523267502

## 189. Prion substrain specific tropism for the spleen: role of PrPC expression levels

Fabienne Reine^a^, Laetitia Herzog^a^, Philippe Tixador^a^, Thanh-Lan Laï^a^, Johann Castille^b^, Bruno Passet^b^, Human Rezaei^a^, Olivier Andréoletti^c^, Jean-Luc Vilotte^b^, Hubert Laude^a^ and Vincent Béringue^a^

^a^VIM, INRA, Université Paris-Saclay, Jouy-en-Josas, France; ^b^GABI, INRA, AgroParisTech, Université Paris-Saclay, Jouy-en-Josas, France; ^c^IHAP, INRA, ENVT, Toulouse, France

**CONTACT** Vincent Béringue vincent.beringue@inra.fr

**ABSTRACT**

Prions are proteinaceous pathogens formed from misfolded conformers (PrPSc) of the host encoded cellular prion protein (PrPC). Prions replicate by templating the conversion and polymerization of PrPC by an autocatalytic process. Multiple strains of prions are recognized phenotypically within the same host species. Strains are conformational variants of PrPSc at the level of the tertiary and the quaternary structure. In infected hosts, prion strains exhibit a stereotyped biological phenotype, that can include specific tropism for the lymphoid tissue. Prion isolates and strains can be composed of several substrain components. These substrains can propagate in distinct tissues of the same infected individual (e.g. brain and spleen), in both homotypic and heterotypic PrP transmission contexts [1–3]. The reason for such tissue selectivity is unclear. Previously, we demonstrated that PrPC expression levels in the brain govern prion substrain selection during homotypic PrP transmission events [4]. Thus, transmission of natural sheep scrapie isolates (termed LAN) to ovine PrP transgenic mice expressing different levels of PrPC in the brain resulted in the phenotypic expression of the dominant LAN substrain in mice expressing near physiological PrPC levels, whereas a minor substrain was preferentially selected on high-expressors.

Considering that PrPC expression levels are markedly decreased in the spleen compared to the brain in wild-type and PrP transgenic mouse models1, we questioned whether PrPC amount could drive prion substrain spleen- selectivity. The outcome of LAN sheep scrapie isolates transmission in the spleen from high-expressors correlated with the replication rate dependency on PrPC amount, with exclusive and prominent colonization of the spleen by the substrain preferentially replicating on low-expressors. After intraperitoneal inoculation of LAN isolates, such early spleen colonization allowed the neuropathological expression of this otherwise brain-unfavoured substrain. In addition, a pair of strain variants resulting from the adaptation of cortical MM2 CJD subtype to ovine high- expressors, and exhibiting differing brain vs. spleen tropism in these mice [3], showed opposite tropism in low- expressors.

Collectively, our results support the view that PrPC expression levels are instrumental in prion substrain specific tropism for the lymphoid tissue.

### 

References[1]BeringueV. et al., Science. 2012;335:472–475.10.1126/science.121565922282814[2]BeringueV. et al., OnePLoS
2008;3:e1419.[3]ChapuisJ. et al., Acta Neuropathol Commun. 2016;4:2–15.10.1186/s40478-016-0284-9PMC474341526847207[4]Le DurA. et al., CommunNat
2017;8:14,170.

## 190. Independent amplification of co-infected long incubation period low conversion efficiency prion strains

Thomas E. Eckland, Ronald A. Shikiya and Jason C. Bartz

Department of Medical Microbiology and Immunology, School of Medicine, Creighton University, Omaha, NE, USA

**CONTACT** Jason C. Bartz jbartz@creighton.edu

**ABSTRACT**

Prion diseases are caused by a misfolded isoform of the prion protein, PrP^Sc^. Prion strains are encoded by strain-specific conformations of PrP^Sc^ and prions can interfere with each other when a long-incubation period strain inhibits the conversion of a short-incubation period strain. Prion strain interference influences the emergence of a strain from a mixture and prion strain dynamics. It is unknown, however, if two long-incubation period strains can interfere with each other. Here, we show that co-infection of animals with combinations of long-incubation period prion strains failed to identify evidence of strain interference. To exclude the possibility that this inability of strains to interfere *in vivo* was due to a failure to infect common populations of neurons we used protein misfolding cyclic amplification strain interference (PMCAsi). Consistent with the animal bioassay studies, PMCAsi indicated that both co-infecting strains were amplifying independently, suggesting that the lack of strain interference is not due to a failure to target the same cells but is an inherent property of the strains involved. Importantly PMCA reactions seeded with long incubation-period strains contained relatively higher levels of remaining PrP^C^ compared to reactions seeded with a short-incubation period strain. Mechanistically we hypothesize that co-infection with prion strains with relatively low prion conversion efficiencies, the abundance of PrP^C^ is not limiting *in vivo* or *in vitro*. Overall, this observation changes the paradigm of the interactions of prion strains and has implications for interspecies transmission and emergence of prion strains from a mixture.

## 191. Neutralization of TACE α -secretase by ROCK and PDK1 kinases contributes to neurodegeneration in prion diseases: loss-of-PrP^C^ protective function involved

Mathéa Pietri^a^, Aurélie Alleaume-Butaux^a^, Juliette Ezpeleta^a^, Vincent Baudouin^a^, Zaira Arellano-Anaya^a^, Anne Baudry^a^, Stéphane Haik^b^, Yannick Bailly^c^, Jean-Michel Peyrin^d^, Odile Kellermann^a^, Jean-Marie. Launay^e^ and Benoit Schneider^a^

^a^Université Paris Descartes, Inserm UMR-S 1124, Paris, France; ^b^INSERM UMR-S 1127, CNRS UMR 7225, Université Pierre-et-Marie Curie, Paris, France; ^c^CNRS UPR 3212, Strasbourg, France; ^d^CNRS UMR 8246, Université Pierre-et-Marie Curie, Paris, France; ^e^INSERM UMR-S 942, Hôpital Lariboisière, Paris, France & Pharma Research Department, Hoffmann La Roche, Basel, Switzerland

**CONTACT** Benoit Schneider benoit.schneider@parisdescartes.fr

**ABSTRACT**

The α -secretase TACE exerts a protective role at the neuronal cell surface by catalysing the cleavage of several substrates, including (i) TNF α receptors (TNFRs), which confers physiological sensitivity to TNF α, and (ii) the cellular prion protein PrP^C^, which limits its conversion into pathogenic prions PrP^Sc^. We have assessed whether deregulation of TACE contributes to neurodegeneration in prion diseases.

Using the 1C11 neuronal cell line, primary cultures of neurons and mice infected with prions, we show TACE deficit at the surface of prion-infected cells. Mechanistically, PrP^Sc^ promotes the internalization of TACE into caveolin-1-enriched vesicles, which diverts TACE activity away from TNFRs and PrP^C^. This renders neurons highly sensitive to TNF α -associated inflammation and amplifies the production of PrP^Sc^ in prion-infected neurons. Whether the neuropathological effect of PrP^Sc^-induced TACE internalization also depends on the uncoupling of the α -secretase from other substrates is currently investigated.

We provide evidence that TACE internalization in prion-infected neurons originates from a loss-of-PrP^C^ control of TACE localization at the cell surface upon PrP^C^ conversion into PrP^Sc^. The silencing of PrP^C^ in uninfected neurons or the conversion of PrP^C^ into PrP^Sc^ in prion-infected neurons both promote plasma membrane aggregation of β 1 integrins and a rise of β 1 integrin signalling. This leads to the downstream overactivation of the kinase ROCK that complexes with another kinase, PDK1, and overstimulates PDK1 activity. Overactivated PDK1 then triggers the displacement of TACE from the plasma membrane to caveolin 1-enriched vesicles. The inhibition of ROCK or PDK1 is sufficient to target TACE back to the plasma membrane where TACE recovers its protective activity and thereby limits the production and neurotoxicity of PrP^Sc^ or the impact of a TNF α inflammatory insult. As the pharmacological inhibition of ROCK or PDK1 mitigates prion diseases in mice, the ROCK-PDK1 duo emerges as a way to combat prion diseases.

## 192. Prion misfolding activity in ultracentrifugated supernatants of ScN2a cell extracts

Margarita Villavedra and David Cullis-Hill

Sylvan Pharmaceutical Pty. Ltd., Sydney, Australia

**CONTACT** David Cullis-Hill david@cullis-hill.com

**ABSTRACT**

Heparan sulphate proteoglycans (HSPG) are involved in many biological processes. There is evidence that suggests they are involved in the conformational conversion of PrP^C^ to the proteinase resistant PrP^SC^. PrP^SC^ accumulates, together with HSPG, in the cerebral prion amyloid plaques which are a characteristic feature in the pathology of the transmissible spongiform encephalopathies (TSE). Further, depletion of HSPG in N2a cells limited the formation of PrP^SC^. Also, sulphated glycans like pentosan polysulfate (PPS) showed capacity to protect PrP^C^ from its conversion to PrP^SC^ in cell culture. Prion proteins have two heparan binding sites and it has been suggested that the sulphated glycans compete with the HS that induce the misfolding of PrP^C^.

In order to study the role of HSPG in the prion misfolding process we obtained supernatants of ultracentrifugated extracts of N2a and ScN2a cells solubilized in PBS, 0.5%Triton, 150mM NaCl, 1mM EDTA, pH 7.2. The misfolding capability of these supernatants were tested by RT-QuIC assay using recombinant 112–231 PrP^C^ (TPrP) or 23-231PrP^C^ (FLPrP).

Our results show that significant misfolding activity persist in the supernatant after centrifugation at 100,000 g for 1h at 4°C. Since PrP^SC^ polymerises into amyloid rods it is expected to be completely restricted to the pellet under these centrifugation conditions. The HSPGs on the other hand are soluble and will remain in the supernatant. Our preliminary results showed that, particularly using FLPrP, the reactivity of some supernatants in the RT-QuIC assay can be similar to that obtained for the original ScN2a homogenates. Although it is not possible to rule out any of the PrP^SC^ being present in the supernatant, its levels would be expected to be very significantly reduced, which does not correspond to our results obtained in the quake assay. We propose that HSPG may play a role in the misfolding activity of this ultracentrifugated supernatant.

## 193. Validating the effect of peptide aptamers on the interaction between Amyloid beta oligomer and Prion protein *in vitro* and *in vivo*

Antonia N. Klein, Sabine Gilch

Department of Ecosystem and Public Health, Calgary Prion Research Unit, Faculty of Veterinary Medicine and Hotchkiss Brain Institute, University of Calgary, Calgary, Canada

**CONTACT** Antonia N. Klein Antonia.Klein@ucalgary.ca

**ABSTRACT**

**Introduction**: Alzheimer’s disease (AD) is a neurodegenerative disease that affects 46.8 Mio people worldwide (estimated in 2015). Aβ monomers aggregates to toxic Aβ oligomers (AβO) and higher aggregates. Studies suggest that the AβO-PrP^C^-interaction at PrP23-27 (low-affinity side) and PrP95-110 (high-affinity side) is required for AβO-induced cell death. Previously, we selected peptide aptamers (PA) binding to PrP^C^ to inhibit the conformational conversion of PrP^C^ to infectious PrP^Sc^ associated with prion diseases. These PAs consist of a 16 amino acid peptide motif integrated into bacterial thioredoxin A (trxA) as a scaffold protein. One PA, termed PA8, binds to PrP^C^ at position 100–120. Furthermore, we developed PA8 variants, termed 46K, 46Q, and 47H, with single amino acid replacements to improve the binding affinity. The PAs increases total PrP^C^ on the cell surface and enhances α-cleavage of PrP^C^ into neuroprotective N1- and C1-fragments. The goal of the study presented here was to validate these PAs for treatment of AD by inhibiting the AβO-PrP^C^-interaction.

**Methods**: Synthetic Aβ(1-42) was used to generate AβO, which were characterized by density gradient centrifugation. AβO-PrP^C^ interaction was analysed by incubating wtN2A cells with FITC-AβO with or without PA, PrP^C^ immunostaining and subsequent analysis of FITC-Aβ and PrP^C^ colocalization. Toxicity induced by the AβO-PrP^C^-interaction was analysed by MTT assays and Western blot analysis of active Caspase 3. In the ongoing *in vivo* study, 7 weeks old 5xFAD mice are treated with PA for 12 weeks intraventricularly using osmotic Alzet® pumps. Behavioural tests like novel object recognition test and Morris water maze will be performed within the last treatment week.

**Results**: The here used PAs inhibited AβO-PrP^C^-interaction. Treatment of wtN2A cells with AβO led to a reduced cell viability of 34 ± 7%. Concomitant treatment of wtN2A cells with PA8 and 46K for 24 h resulted in a significant increase of cell viability to 44 ± 8% and 48 ± 16%, respectively. Treating SH-SY5Y cells (not detectable PrP^C^ levels) with PAs and AβO, followed by MTT assay confirmed that the enhanced cell viability is PrP^C^ dependent. Western blot analysis of active Caspase 3 proved reduced Caspase 3 activation after treatment of wtN2A cells with PAs. Whether these effects ultimately result in memory improvement will be evaluated through the *in vivo* studies.

**Conclusion**: In conclusion, the here used PA were able to inhibit the AβO-induced toxicity *in vitro*. These findings indicate that PAs are interesting candidates for the treatment of both prion diseases and AD.

## 194. The photodynamic inactivation reduces prion infectivity in phthalocyanine treated RML brain homogenate

Marie Kostelanska^a^, Zdenka Backovska Hanusova^a^, Jakub Soukup^a^, Radoslav Matej^b^ and Karel Holada^a^

^a^Institute of Immunology and Microbiology, First Faculty of Medicine, Charles University and General University Hospital in Prague, Czech Republic; ^b^Department of Pathology and Molecular Medicine, Thomayer Teaching Hospital, Prague, Czech Republic

**CONTACT** Jakub Soukup marie.kostelanska@lf1.cuni.cz

**ABSTRACT**

**Introduction**: Prions are highly resistant to conventional sterilization procedures and currently recommended inactivation methods include treatment with highly corrosive chemicals like 2% NaClO or 1M NaOH. Such harsh treatment is incompatible with a number of delicate medical tools. We have previously demonstrated that nontoxic disulfonated hydroxyaluminum phthalocyanine (AlPcOH(SO_3_)_2_) can be used to initiate photodynamic inactivation (PDI) of prions at ambient pressure and temperature [1]. The aim of this study was to demonstrate the effectiveness of PDI of prions by bioassay in laboratory mouse.

**Methods**: The PDI was performed on 1% RML or uninfectious CD1 brain homogenates (BH) treated with 25 µg ml^−1^ of AlPcOH(SO_3_)_2_ and exposed to red light (18 min, 4 × 2.5W LED). Control RML inoculum was prepared similarly but was kept in the dark. In mouse bioassay, CD1 female mice (n = 10) were intracerebrally inoculated with a 25-µl aliquot of AlPcOH(SO_3_)_2_-light treated RML BH or control RML BH containing AlPcOH(SO_3_)_2_ but not irradiated by light. Other controls included mice (n = 5) inoculated with untreated, uninfectious CD1 BH or CD1 homogenate containing AlPcOH(SO_3_)_2_, either irradiated or nonirradiated. For comparison groups of mice (n = 5) were inoculated with 10-fold dilutions of RML BH (10^−2^ to 10^−6^). The animals were sacrificed after reaching the terminal disease phase. The presence of PrPres in mouse brains was detected by WB and evaluation of PrPres distribution in brains by immunohistochemistry is ongoing.

**Results**: Mice inoculated with PDI-treated RML BH (at a 10^−2^ dilution) survived significantly longer (204 ± 23 days) than mice inoculated with the homogenate containing an identical amount of AlPcOH(SO_3_)_2_, that was not exposed to light (165 ± 11 days). Their survival was also significantly longer than that of mice inoculated with straight 10^−2^, 10^−3^, and 10^−4^ dilutions of the RML BH, who survived 169 ± 11, 165 ± 12, and 176 ±15 days, respectively. The transmission rate was 100% in all groups of mice including serial dilution (10^−2^ to 10^−6^). A comparison of survival time based on the regression line suggested a decrease in the prion infectivity titer of the RML BH of more than four orders of magnitude after PDI treatment.

**Conclusions**: Our results suggest that PDI with AlPcOH(SO_3_)_2_ leads to substantial decrease (~4 log_10_) of prion infectivity in RML BH. The PDI of prions by light induced photocatalytic activity of phthalocyanines represents promising way of prion decontamination.

**KEYWORDS:** Prion; phthalocyanine; photodynamic inactivation; decontamination; mouse bioassay

The project was supported by AZV NV18-04–00179.

### 

Reference[1]Janouskova et al., J Gen Virol. 2012; 93(Pt11): 2512–2517.10.1099/vir.0.044727-022855785

## 195. When anchorage determines fate: How the GPI-anchor signal sequence impacts on prion protein biology and prion disease in mice

Berta Puig^a^^,^^b^, Hermann C. Altmeppen^a^, Luise Linsenmeier^a^, Karima Chakroun^a^, Ulrike K. Piontek^c^, Jörg Tatzelt^c^^,^^d^, Clive Bate^e^, Tim Magnus^b^ and Markus Glatzel^a^

^a^Institute of Neuropathology; ^b^Department of Neurology, Experimental Research in Stroke and Inflammation (ERSI), University Medical Center Hamburg-Eppendorf (UKE), Hamburg, Germany; ^c^Adolf Butenandt Institute, Ludwig Maximilians University, Munich, Germany; ^d^Institute of Biochemistry and Pathobiochemistry, Biochemistry of Neurodegenerative Diseases, Ruhr University, Bochum, Germany; ^e^Department of Pathology and Pathogen Biology, Royal Veterinary College, Hawkshead Lane, North Mymms, Herts, UK

**CONTACT** Hermann C. Altmeppen h.altmeppen@uke.de

**ABSTRACT**

Correct anchoring and localization of the prion protein (PrP) at the neuronal surface are essential for its processing, functions and roles in disease. Several studies have revealed a compensatory network regulating PrP membrane homeostasis, including proteolytic or extracellular vesicle release, and internalization followed by retrograde transport and degradation. In this study however, we asked if the GPI-anchor signal sequence (GPI-SS) -though not part of the mature protein- affects composition, trafficking, membrane localization and, hence, biology of PrP in the first place, and whether alterations in that regard would impact on prion disease in particular. We generated different lines of transgenic mice expressing a mutant PrP (PrP^C^GPIThy-1) containing the GPI-SS of the neuronal protein Thy-1, a resident of dense cores of lipid rafts. This modification indeed resulted in a different GPI-anchor composition (now lacking the rare mammalian sialic acid typical for PrP), altered sorting in polarized cells (such as primary neurons), and reduced proteolytic shedding by the metalloprotease ADAM10. When inoculated with either RML or 22L prions, survival of mice was significantly prolonged for all transgenic lines, despite similar or even higher expression levels of generally signalling- and misfolding-competent PrP^C^GPIThy-1 compared to wild-type controls. Fittingly, MAP kinase signalling as well as glial activation and spongiform lesions were reduced in brains of our transgenic mice. Using PrP as a model, this is the first *in vivo* study demonstrating that the GPI-SS determines the later remodelling and composition of a given protein`s GPI-anchor and sorting, with likely consequences for its biology. Besides drastic effects on different pathomechanistic aspects underlying the course of prion diseases, our findings may also have wider implications for the field of GPI-anchor biology.

**KEYWORDS:** ADAM10; glycosylphosphatidylinositol (GPI)-anchor; lipid rafts; prion protein; shedding; signalling; sorting

### 

Reference[1]PuigAltmeppen et al., PathPLoS
2019.

## 196. Reverting prion disease in neuronal progenitor cells as a cell replacement therapy

Arielle Hay, Lindsay Parrie, Glenn C. Telling, Mark Zabel and Julie A. Moreno

Prion Research Center, Department of Microbiology, Immunology & Pathology, Colorado State University, Fort Collins, CO, USA

**CONTACT** Arielle Hay Arielle.Hay@colostate.edu

**ABSTRACT**

We propose the use of gene edited stem cells as therapy for neurodegenerative diseases. Olfactory neuronal progenitors (ONPs) can differentiate into neurons in adulthood. We hypothesize they will regenerate neurons that have been lost due to aggregation of disease- associated proteins. Mesenchymal stem cells (MSCs) can be derived from adipocytes of adult mice and further differentiated to neural stem cells (NSCs), which we plan to use as an alternative to ONPs. These therapies will be modelled by prion infected mice, which display the typical features of neurodegenerative disease, including clinical signs and loss of neurons. Prion diseases are the result of misfolding of the prion protein (PrP), which is highly expressed in neurons. PrP knockout mice are resistant to prion diseases. Two populations of ONPs will be made using CRISPR/Cas9 gene editing to delete the *prnp* gene in N2a cells and WT ONPs. These cells will be subjected to prion cell assays to determine if they can be infected. The second population of ONPs will be from mice with a mutation associated with the human prion disease Gerstmann–Sträussler–Scheinker syndrome (GSS), which is modelled in mice by a proline to leucine switch at codon 101 of moPrP. We plan to use CRISPR/Cas9 to revert this mutation. Alternatively, we plan to edit the *prnp* gene in M/NSCs so they express a secretable, dominant- negative PrP. Single cell sorting will be used to produce homogenous populations of edited cells, which will be sequenced to identify relevant mutations. We will engraft gene edited ONPs using stereotactic injection and M/NSCs using nasal instillation into the brains of prion infected WT and GSS transgenic mice [1]. We hypothesize that these cells will resist prion infection. ONPs will migrate throughout the brain to restore damaged neurons and M/NSCs will populate the olfactory bulb and secrete dominant negative PrP and anti-inflammatory cytokines throughout the brain.

## 197. Proteolytic shedding of PrP can be manipulated in a substrate-specific manner by certain PrP-binding ligands

Luise Linsenmeier^a^, Behnam Mohammadi^a^, Mohsin Shafiq^a^, Simrika Thapa^b^, Karl Frontzek^c^, Berta Puig^a^, Chaoyang Li^d^, Michaela Schweizer^e^, Tania Massignan^f^, Emiliano Biasini^f^, Antonia Klein^b^, Sabine Gilch^b^, Luca Varani^g^, Hermann Schätzl^b^, Adriano Aguzzi^c^, Hermann C. Altmeppen^a^* and Markus Glatzel^a^*

^a^Institute of Neuropathology, University Medical Center HH-Eppendorf, Hamburg, Germany; ^b^Calgary Prion Research Unit, Faculty of Veterinary Medicine, University of Calgary, Canada; ^c^Institute of Neuropathology, University Hospital Zurich, Switzerland; ^d^Wuhan Institute of Virology, Chinese Academy of Science, Beijing, China; ^e^Center for Molecular Neurobiology Hamburg, Morphology and Electron Microscopy Unit, University Medical Center HH-Eppendorf, Hamburg, Germany; ^f^Department of Cellular, Computational and Integrative Biology (CIBIO), University of Trento, Italy; ^g^Institute for Research in Biomedicine, Università della Svizzera italiana, Bellinzona, Switzerland

*These authors contributed equally to this work.

**CONTACT** Luise Linsenmeier l.linsenmeier@uke.de

**ABSTRACT**

The prion protein (PrP^C^) impacts on development and maintenance of the nervous system and plays a deleterious role in fatal and progressive prion diseases. Moreover, in other neurodegenerative proteinopathies it acts as a receptor for toxic oligomers such as Aβ in Alzheimer’s disease (AD). PrP^C^ is released from the cell surface into the extracellular space by the metalloprotease ADAM10. We have shown that this cleavage significantly impacts on prion disease, and soluble PrP fragments may be key in understanding the multitude of functions suggested for PrP^C^, such as in myelin maintenance, neuroprotection, synapse formation or neuroimmune crosstalk.

We expect the ADAM10-mediated shedding of PrP^C^ to have therapeutic potential against neurodegenerative proteinopathies. However, a substrate-specific approach to stimulate this cleavage would likely be superior to targeting of the protease, given the plethora of ADAM10 substrates throughout the body and, thus, the risk of severe side-effects. We found that several PrP-directed antibodies significantly increase shedding in different cell culture models and organotypic slice cultures. Crosslinking of PrP is not a prerequisite since treatment with single chain antibodies and other PrP-binders likewise resulted in increased cleavage. We thus assume that binding of certain ligands to PrP per se stimulates its shedding. While we also identified some antibodies that blocked shedding, one particular antibody (likely due to its unique binding characteristics) led to strong surface clustering, fast internalization and degradation of PrP rather than to proteolytic release from the membrane.

Of note, PrP-directed antibodies or compounds have been experimentally suggested or are currently tested as a treatment option in fatal neurodegenerative proteinopathies. It remains to be investigated whether stimulated shedding is (at least partially) underlying the protective mechanism. In contrast, given the recently suggested harmful roles of shed PrP in brain cancer and HIV-associated neuropathogenesis, the shedding-inhibiting ligands identified in our study may also hold therapeutic potential in pathological conditions affecting the brain.

## 198. Predicting Blood-Brain Partitioning and Skin Permeability using 3D-RISM-KH Theory Based Solvation Energy Descriptors

Andriy Kovalenko

Department of Mechanical Engineering, University of Alberta, 10–203 Donadeo Innovation Centre for Engineering, Edmonton, Alberta, Canada; Nanotechnology Research Centre, Edmonton, Alberta, Canada

**ABSTRACT**

Compartmentalization of drug molecules between plasma and brain is important for desired activity. The difficulty in obtaining the blood-brain permeability of drug (like) substances experimentally resulted in the development of several theoretical quantitative structure activity relationships (QSAR) towards predicting capability of a given substrate to pass across tight junction, known as the blood-brain barrier, both qualitatively and quantitatively. We have developed a novel method based on the three-dimensional reference interaction site model with the Kovalenko-Hirata closure relation (3D-RISM-KH) molecular solvation theory for predicting blood-brain barrier permeability coefficients of molecules in diverse structures with a minimum set of descriptors derived from solvation energetics [1]. Our findings reveal the importance of solvation free energy based descriptors in quantitatively modelling blood-brain barrier permeability. Further, we use the state-of-the-art molecular solvation theory to predict skin permeability of a large set of compounds with experimental logKP [2]. We obtained encouraging results pointing to applicability of the model using statistical physics based 3D-RISM-KH theory for solvation free energy calculations as a primary descriptor with relative mean square error of 0.77 units.

### 

References[1]RoyD, HingeVK, KovalenkoA.
Predicting blood-brain partitioning of small molecules using a novel minimalistic descriptor based approach via the 3D-RISM-KH molecular solvation theory. *ACS Omega*. 2019
(accepted).10.1021/acsomega.9b01512PMC679693031646222[2]HingeVK, RoyD, KovalenkoA.
Predicting skin permeability using the 3D-RISM-KH theory based solvation energy descriptors for a diverse class of compounds. *J Pharm Sci*, 2019
(submitted).10.1007/s10822-019-00205-z31087228

## 199. Chronic wasting disease transmission studies to non-human primates and transgenic mice

Brent Race, Katie Williams, Christina D. Orrù, Andrew G. Hughson, Lori Lubke and Bruce Chesebro

National Institute of Allergy and Infectious Diseases, Laboratory of Persistent Viral Diseases, Rocky Mountain Laboratories, Hamilton, MT, USA

**CONTACT** Brent Race raceb@niaid.nih.gov

**ABSTRACT**

**Introduction:** Chronic wasting disease (CWD) is the only prion disease affecting free-ranging animals, reported in North America, South Korea and Norway. Unlike in most other prion disease CWD agents are shed in blood, urine and feces which most likely contribute to the horizontal transmission between cervid species. The developments of amplification-based seeding assays have been instrumental in the detection of low levels of prions in clinical samples. Using real-time quaking-induced conversion (RT-QUIC), we established an ultrasensitive detection method for PrPCWD in the urine from CWD-infected sequentially sampled transgenic mice overexpressing elk prion protein (TgElk mice). In addtion, RT-QUIC was performed in the kidney and brain of theses mice model to trace abnormal prion. **Materials and Methods:** 44 brain and kidney, urine samples from sequentially collected from CWD-infected TgElk mice (TgElk CWD) were stored at -80℃. In brain and kidney, 10% (w/v) homogenate was prepared in 0.9% sterilized saline. In urine 100uL of each sample was mixed with 10uL 2.8% sodium phosphotungustic acid (NaPTA) and incubated for 1hr at 37℃ with shaking at 1,350 rpm. Samples were centrifuged for 30min at 16,100g. The pellet was resuspended in 10uL of 0.1% SDS/PBS for 30min at 55℃. RT-QUIC reactions were set up in 96-well clear bottom optic plates and consisted of 98uL RT-QUIC buffer [final concentrations of 1XPBS, 1mM EDTA, 10uM Thioflavin, 300mM NaCl buffer and 0.1mg/ml recombinant Syrian hamster recombinant protein (23-231) and 2uL of sample. The RT-QUIC assay was performed on a FLUOstar Omega fluorescence plate reader that was preheated to 42℃ for 60hr with 90sec shaking at 700rpm followed by 1min incubation. **Results:** Five randomly selected mice were sequentially culled on every 15 days from 30dpi to 120dpi during CWD infected TgElk mice reached terminal stage. Rough hair coats among clinical signs were showed from 90 dpi. PrPCWD in the brain in TgElk CWD was detectable persistently from early stages (30dpi), and in the kidney PrPCWD was also detectable in clinical and terminal stages (90 dpi and 120dpi). PrPCWD in the urine in TgElk CWD reached the highest levels at 120dpi. NaPTA/RT-QUIC was applied to measure PrPCWD in urine samples collected on every 15 days from 30dpi to 120dpi when CWD infected TgElk mice reached terminal stage. PrPCWD in the urine in TgElk CWD reached the highest levels at 90dpi. PrPCWD was also detectable in late and terminal stages (120dpi). **Conclusions:** We demonstrate that CWD prions can be detected by RT-QUIC or NaPTA/RT-QUIC in the brain, kidney and urine of TgElk mice at the early and terminal stages of disease. Based on these data, we suggest that PrPCWD is excreted into only urine until 90 dpi and then slowly accumulated in kidney. Our results can be used in designing future study of CWD pathogenesis in TgElk mice.

**KEYWORDS:** Cynomolgus macaques; squirrel monkeys; non-human primate; transgenic mice; CWD; RT-QuIC; prion; cross-species transmission; barrier; chronic wasting disease

### 

References[1]Henderson DM et al
Rapid antemortem detection of CWD prions in deer saliva PLOS one
2013, 397, 626-630.10.1371/journal.pone.0074377PMC377061124040235[2]Nagooka K, Yoshika M, Shimozaki N. et al
Sensitive detection of scrapie prion protein in soil. Biochem Biophys Res Commun. 2010
e74377 1-12.10.1016/j.bbrc.2010.06.01320570651

## 200. DNA methylation array based machine learning classification of human prion disease

Fernando Guntoro, Luke Dabin, John Collinge, Emmanuelle Vire, and Simon Mead

MRC Prion Unit at UCL, UCL Institute of Prion Diseases, London, UK

**CONTACT** Fernando Guntoro f.guntoro@ucl.ac.uk

**ABSTRACT**

**Background:** Variant Creutzfeldt-Jakob disease (vCJD) was caused by the widespread dietary exposure of the United Kingdom (UK) and other populations to bovine spongiform encephalopathy (BSE) prions. Unlike other forms of CJD, abnormal prion proteins (PrP) in vCJD are readily detectable by conventional histopathology outside the central nervous system, particularly in lymphoreticular system (LRS) tissues such as tonsillar and appendix tissues. To date, six prevalence studies have been conducted; the fifth (Appendix II) and sixth (Appendix III) studies reported abnormal PrP immunoreactivity in 16 out of 32,441 appendix samples and 2 out of 14,692 samples, respectively, indicating a surprisingly high prevalence abnormal PrP. Given the 178 vCJD cases hitherto reported in the UK, there appears to be a discordance between the population prevalence data and the number of observed clinical cases, which challenges the assumption of a perfect association between PrP deposition in LRS tissues and vCJD disease. To provide insight into this discordance, we aim to develop a classifier based on epigenomic profiles that distinguishes prion infection from healthy controls.

**Materials and Methods:** A unique archive of fixed and frozen LRS tissues including tonsil and appendix from vCJD patients, blood and brain samples from a range of human prion disease patients. Samples have been age/sex-matched to controls not diagnosed with neurodegenerative disease. For DNA methylation profiling, extracted genomic DNA has been subjected to bisulfite conversion and, subsequently, hybridized to the Illumina 450K or 850K EPIC arrays. Training of a deep learning neural network model was performed using these samples.

**Results:** Initial results explored the diagnostic potential of an Illumina 450K dataset using deep learning neural network classifier. This DNA methylation study was conducted using whole bloods from 106 healthy controls and 114 sCJD patients (age/sex-matched). Using data from the 1000 most significantly altered methylation loci, this data was randomized and partitioned into training and test set of 1:1 case-control ratio. After 10-fold validation, the model had an accuracy of 85.64% (± 1.14%) with an upper limit of 90.00% accuracy. The trained neural network model demonstrated a better performance compared to a basic Random Forest classifier with an AUC of 0.965 and 0.841, respectively.

**Conclusions:** As a proof of concept, we have developed an accurate classification tool using relatively small numbers of differentially methylated loci in blood. We are extending these results to a wider panel of neurodegenerative dementias and LRS tissues.

**KEYWORDS:** Epigenetics; EWAS; DNA methylation; Variant Creutzfeldt-Jakob; Machine learning; Appendix; Tonsil; Lymphoreticular tissue

## 201. TDP-43 induces SOD1 aggregation and toxicity via tryptophan interactions that present a novel drug target

Michèle G. DuVal^a^, Edward Pokrishevsky^b^, Vijaya K. Hinge^c^^,^^d^, Jenna Bratvold^a^, Richard Kanyo^a^^,^^e^, Natalie Snyder^a^, Nikolay Blinov^c^^,^^d^, Andriy Kovalenko^c^^,^^d^, Neil R. Cashman^b^ and W. Ted Allison^a^^,^^e^^,^^f^

^a^Department of Biological Sciences, University of Alberta, Edmonton, *AB*, Canada; ^b^Djavad Mowafaghian Centre for Brain Health, University of British Columbia, Vancouver, BC, Canada; ^c^National Institute for Nanotechnology, Edmonton, *AB*, Canada; ^d^Department of Mechanical Engineering, University of Alberta, Edmonton, *AB*, Canada; ^e^Centre for Prions and Protein Folding Diseases, University of Alberta, Edmonton, *AB*, Canada; ^f^Department of Medical Genetics, University of Alberta, Edmonton, *AB*, Canada

**CONTACT** Michèle G. DuVal mgduval@ualberta.ca

**ABSTRACT**

Amyotrophic lateral sclerosis (ALS) is a devastating motor neurodegenerative disease which features a diversity of protein inclusions. Aggregates of superoxide dismutase 1 (SOD1) or of TAR-DNA binding protein-43 (TDP-43) have been identified in familial and sporadic cases. Misfolded wildtype SOD1 can spread in a prion-like fashion, evidenced by induction of wildtype SOD1 misfolding by mutant SOD1 or by TDP-43 variants, as observed in cells and cultured neurons [1–3]. The sole tryptophan in SOD1 at position 32 (W32) is instrumental in SOD1 misfolding [1,2,4], and we hypothesize that TDP-43 also induces SOD1 misfolding via interactions between their respective tryptophan residues. We measured the contribution of these tryptophans to SOD1 aggregation *in vitro* and toxicity *in vivo* by expressing variants with combinations of tryptophan substitutions.

In HEK293 cells, TDP-43 lacking all six tryptophans induced little SOD1 inclusion formation, which was replicated with TDP-43 variants lacking two specific tryptophans. In a zebrafish model of motor neuron disease [5–7], disturbed motor axon morphology caused by TDP-43 and SOD1 co-expression was similarly rescued, with congruent results for substitutions of specific tryptophans in TDP-43. Thus TDP-43 can induce SOD1 aggregation and toxicity via interactions between tryptophans on the two proteins, which may occur under pathological conditions and thereby advance disease. Similarly, mutating W32 in the human SOD1 delivered to zebrafish lead to significant reduction of motor neuron phenotypes (vs wildtype SOD1), including preservation of axon morphology and of motor output (swimming).

Candidate therapeutic compound 5’-fluorouridine, known to bind at the W32 region of SOD1 [8], successfully rescued cross-seeding of SOD1 by TDP-43 and subsequent motor neuron toxicity. Next, to identify additional compounds that would bind at W32, high throughput virtual and pharmacophore screening was performed with modelling of the W32 binding site and screening of FDA-approved drugs that closely match key features of 5’-fluorouridine. From among top-ranked compounds, telbivudine significantly rescued SOD1-induced motor neuron phenotypes [9]. In conclusion, we found that tryptophan 32 in SOD1 is a significant contributor to SOD1 aggregation and toxicity, including via novel TDP-43-SOD1 interactions. These interactions represent new pharmacologic targets and we identified FDA-approved compounds, predicted to interact with SOD1 at W32, that are effective in preventing inclusion formation and toxicity.

**KEYWORDS:** SOD1; TDP-43; prion-like; ALS; tryptophan; 5’-fluorouridine; telbivudine; zebrafish

### 

References[1]Grad et al., PNAS. 2011;111:3620–3625.[2]Taylor et al., J Biol Chem. 2007;282:16329–16,335.10.1074/jbc.M61011920017389599[3]Pokrishevsky et al., Sci Rep. 2016;6:22155.10.1038/srep22155PMC477200926926802[4]Pokrishevsky et al., Sci Rep. 2018; 8:15590.10.1038/s41598-018-32835-yPMC619719630349065[5]Sakowski et al., Mol Neurodegener. 2012;7:44.10.1186/1750-1326-7-44PMC350651522938571[6]DuVal et al., PLoS One
2014;9:e8918310.1371/journal.pone.0089183PMC393846224586579[7]Edens et al., eLife. 2017; 6:e25453[8]Wright et al., Nat Commun. 2013;4:175810.1038/ncomms2750PMC364408723612299[9]DuVal et al., Neurobiol Dis
2019; 124: 297–31010.1016/j.nbd.2018.11.02530528257

## 202. Competitive ELISAs to determine PrP^Sc^ content in crude homogenates without the need for denaturation or protease treatments

Xinli Tang^a^^,^^b^, Andrew Fang^a^^,^^b^, Leonardo M. Cortez^a^^,^^c^, Camilo Duque Velásquez^a^^,^^d^, Claudia Y. Acevedo-Morantes^a^^,^^b^, Brian Tancowny^a^^,^^b^, Valerie L. Sim^a^^,^^c^, Judd Aiken^a^^,^^e^, Debbie McKenzie^a^^,^^d^ and Holger Wille^a^^,^^b^

^a^Centre for Prions and Protein Folding Diseases, University of Alberta; ^b^Department of Biochemistry; ^3^Department of Medicine; ^d^Department of Biological Sciences, ^e^Department of Agricultural, Food, and Nutritional Science, Edmonton, Alberta, Canada

**CONTACT** Xinli Tang xinli@ualberta.ca

**ABSTRACT**

**Background:** The lack of readily accessible, PrP^Sc^-specific monoclonal antibodies has hampered the detection of PrP^Sc^ in brain homogenates and other samples. The presence of PrP^C^ and other, non-infectious PrP conformers confounds diagnostic assays that lack specificity for native PrP^Sc^. Currently used diagnostics often rely on the partial protease resistance of PrP^Sc^, which is used to differentiate PrP^Sc^ from PrP^C^ and other PrP conformers. Furthermore, the aggregated nature of PrP^Sc^ obscures many epitopes, making denaturation of PrP^Sc^ a prerequisite for many detection assays. Together these requirements affect the specificity and sensitivity of the detection assays and increase the necessary labour, time, and resources to determine if a sample contains PrP^Sc^.

**Materials and Methods:** We immunized mice with a structural and immunological mimic for PrP^Sc^, which was based on the fungal HET-s prion domain as a scaffold. At the end of the immunization schedule we used their spleens to generate hybridomas for the production of monoclonal antibodies. Here, we tested the resulting antibodies in conventional and competitive ELISAs for their ability to detect native PrP^Sc^ from different prion isolates and to distinguish PrP^Sc^ from PrP^C^ and other PrP conformers.

**Results:** Immunization with our newly developed, structural mimic gave rise to a PrP^Sc^-specific immune response in 8/8 mice. The resulting monoclonal antibodies (IgGs and IgMs) recognized native PrP^Sc^ in crude brain homogenates without any pre-treatment, but did not bind to PrP^C^, recombinant PrP, denatured PrP, unseeded PrP amyloid, or any linear peptide of PrP. We tested a variety of assay formats and obtained the most reliable results using a competitive/inhibition ELISA. Here, our original antigen was used to coat the assay plates and to compete with PrP^Sc^ in the samples (typically crude brain homogenate) for binding of unconjugated antibodies. A conventional detection system using a secondary antibody (HRP-goat-anti-mouse IgG or IgM) generated the final colorimetric readout. The assay recognized native PrP^Sc^ from all prion isolates tested, including CWD, BSE (C-, H-, and L-type), scrapie (RML), TME (Hyper and Drowsy), sCJD, vCJD, fCJD, FFI, and GSS, indicating its ability to detect a widely-shared PrP^Sc^-specific epitope.

**Conclusions:** Our new, PrP^Sc^-specific monoclonal antibodies allowed the development of a competitive ELISA for the detection of native PrP^Sc^ without protease or denaturant pretreatment. Furthermore, the broad specificity of these antibodies opens the possibility to develop a widely applicable and sensitive diagnostic tool for the direct detection of PrP^Sc^.

## 203. Seeding of a fast acting Aß peptide accelerates AD-like pathology in APP^NL-G-F^ mouse model with no impairment in learning and memory

Sean G. Lacoursiere, Majid H. Mohajerani and Robert J. Sutherland

Canadian Centre for Behavioural Neuroscience, University of Lethbridge

**CONTACT** Sean G. Lacoursiere sean.lacoursiere@uleth.ca

**ABSTRACT**

Alzheimer’s disease (AD) is a neurodegenerative dementia characterized by the stereotypical conformational misfolding of proteins. Of specific interest is amyloid-ß (Aß) due to its prion-like properties evidenced by the seeding of Aß accelerating plaque deposition in brain. However, the role of Aß in AD remains unclear. Many hypothesize that increasing Aß impairs cognition; and therefore, accelerating Aß pathology should increase the extent of cognitive decline to the point of dementia. We performed bilateral intracerebral microinjections of a fast-acting Aß peptide into the medial entorhinal cortex of the knock-in APP^NL-G-F^ mouse model of AD at 2 months of age. We found deposition of Aß plaque occurred sooner and to a greater extent compared to controls but without obvious impairment in cognition or overall health. We found that at 6 months the APP^NL-G-F^ mice showed similar memory performance in a no-platform Morris Water Task probe test compared to controls. Immunohistochemical labelling showed dramatically increased 82E1 and Iba1 label in these mice 1 month following injection, indicating increased Aß and microgliosis pathology, respectively. Our results replicate previous reports of the seeding properties of Aß and its prion-like behaviour. They also support notions that Aß may not be sufficient to cause cognitive decline in Alzheimer’s disease. The Aß pathology in AD may be a response to some other trigger. The seeding properties of Aß may be an adaptive response to control some other process.

## 204. Alteration to the prion protein’s central region sequence retains structure but results in toxicity

Graham P. Roseman^a^, Bei Wu^b^, Mark Wadolkowski^a^, David A. Harris^b^ and Glenn L. Millhauser^a^

^a^University of California Santa Cruz; ^b^Boston University School of Medicine

**CONTACT** Graham P. Roseman groseman@ucsc.edu

**ABSTRACT**

The central region (CR) of the prion protein (PrP^C^) is one of the most highly conserved sequences across species. Deletion of the entire prion protein gene in mice is fairly tolerated, but deletion of just the CR (∆CR: 105–125) causes neonatal fatality in mice [1] and toxicity in cell culture as seen by spontaneous currents using patch clamp electrophysiology experiments. There is a great interest in understanding the toxicity of this deletion mutant because the neurotoxicity has similarities to that of classic prion diseases except without the aggregation of PrP^C^. Biophysical studies have shown that ∆CR causes two main changes: weakening of the metal-driven *cis* interaction between the N- and C- terminal domains [2] and deletion of the region of the protein where regulatory proteolysis occurs, termed α-cleavage. What is not known is whether the neurotoxicity that ∆CR causes is a result of a weakened *cis* interaction or elimination of proteolytic sites. This study aims to ask that question by using a designed PrP^C^ construct that replaces the central region with a flexible glycine-serine (GS) rich linker that retains the metal-driven *cis* interaction of wild type PrP^C^ while blocking the cleavability of this region. Results from the GS linker show that the *cis* interaction is retained as tested by NMR, α-cleavage was blocked *in vitro* and in cell culture, and toxicity was seen using electrophysiological experiments. To test if the toxicity generated from the GS linker construct was due from eliminating α-cleavage, a rescue construct was designed by reintroducing the canonical α-cleavage sequence into the GS linker backbone. α-cleavage was successfully reintroduced and the metal-driven *cis* interaction was retained. However, when this construct was tested using the electrophysiology experiments, spontaneous currents still persisted, indicating that there was still toxicity. Overall these results implicate that the sequence of the central region is extremely important for the structure and function of the prion protein, and when deleted or mutated, spontaneous neurotoxicity occurs.

**KEYWORDS:** Metal binding; copper; cleavage; NMR; electrophysiology

### 

References[1]Li et al., EMBO. 2007;26:548–558.10.1038/sj.emboj.7601507PMC178344817245437[2]Wu et al., eLife. 2017;6:e23,473

## 205. Deep learning-based pre-mortem diagnostic methods for sporadic Creutzfeldt-Jakob disease using multiple CSF biomarkers

Sol Moe Lee^a^^,^^b^, Jae Wook Hyeon^a^, Soo-Jin Kim^b^, Heebal Kim^b^, Ran Noh^a^, Seonghan Kim^a^, Yeong Seon Lee^a^, Sung Soon Kim^a^ and Su Yeon Kim^a^

^a^Division of Bacterial Disease Research, Center for Infectious Diseases Research, Korea National Institute of Health, Centers for Disease Control and Prevention, Cheongju-si, Chungcheongbuk-do, South Korea; ^b^Department of Agricultural Biotechnology and Research Institute of Agriculture and Life Sciences, Seoul National University, Seoul, South Korea

**CONTACT** Sol Moe Lee arabe@korea.kr; Su Yeon Kim tenksy@korea.kr

**ABSTRACT**

Creutzfeldt-Jakob disease (CJD) is a fatal neurodegenerative disorder which is characterized by the abnormal accumulation of misfolded prion protein (PrP^Sc^) affecting the central nervous system. The diagnosis of CJD can only be confirmed by abnormal protease-resistant prion protein accumulation in post-mortem brain tissue. Sporadic CJD (sCJD) is the most common subtype, and the relationships between sCJD and cerebrospinal fluid (CSF) proteins such as 14–3-3, tau, and α-synuclein (a-syn) have been investigated for their potential values in pre-mortem diagnosis. Deep learning (DL), a machine learning (ML) based on neural networks, has provided a powerful remedy for analysing large-scale data in diverse fields such as computer vision, natural language processing, and signal modelling for several years. Recently, it is reported that DL has attracted large attention in neurodegenerative disease research as well. Here, ML and DL analysis were performed using multiple CSF biomarkers related with neuronal diseases including CJD and Alzheimer’s diseases. We performed ML analysis including decision trees and SVMs using WEKA (Waikato Environment for Knowledge Analysis 3.8.2), and DL analysis was conducted with Keras neural network library. Enzyme-linked immunosorbent assays were performed on phospho-tau (p-tau), total-tau (t-tau), a-syn, and β-amyloid (1-42), and western blot analysis was performed for 14–3-3 protein from CSF samples of 49 sCJD and 256 non-CJD Korean patients, respectively. The best performing ML model demonstrated 78.85% accuracy. The deep neural network structure which showed the best DL performance was comprised of one input, five hidden, and one output layers, with 20, 40, 30, 20, and 12 hidden unit numbers per hidden layer, respectively. For the analysed CSF biomarkers, the DL model demonstrated 90.38% accuracy, 83.33% sensitivity, and 92.5% specificity for the three-protein combination of t-tau, p-tau, and a-syn. DL-aided pre-mortem diagnosis may provide a suitable tool for discriminating CJD patients from non-CJD patients. For further study, we plan to perform DL analysis using stacked sample sizes via ELISA and western blot analysis to establish a DL model with greater high performance and classification accuracy.

## 206. Characterizing the molecular environment of the bank vole prion protein using mass spectrometry

Hamza Arshad^a,b^, Declan Williams^a^, Lech Kaczmarczyk^c^, Walker Jackson^c^, Gerold Schmitt- Ulms^a,d^, Joel Watts^a,b^

^a^Tanz Centre for Research in Neurodegenerative Diseases, Toronto, Canada; ^b^Department of Biochemistry, University of Toronto, Toronto, Canada; ^c^Wallenberg Center for Molecular Medicine, Linköping University, Linköping, Sweden; ^d^Department of Laboratory Medicine and Pathobiology, University of Toronto, Toronto, Canada

**CONTACT** Hamza Arshad hamza.arshad@mail.utoronto.ca

**ABSTRACT**

**Background:** The bank vole prion protein (BVPrP) is able to replicate a wide range of prion strains from different species and has been characterized as a universal acceptor of prions [1,2]. The sequence of mature BVPrP differs from that of mouse PrP (MoPrP) at only eight different amino acid positions. We hypothesized that these unique residues may allow BVPrP to tailor its molecular environment through novel protein-protein interactions. To address this issue, we generated knock-in (ki) mice that express BVPrP (M109 isoform) at physiological levels in the brain. The PrP interactomes in ki mice expressing BVPrP and wild-type mice expressing MoPrP were compared using quantitative mass spectrometry.

**Materials and Methods:** BVPrP ki mice, wild-type C57Bl/6 mice, and PrP knockout mice were subjected to time-controlled transcardiac perfusion crosslinking (tcTPC) and then PrP- containing protein complexes were isolated from the brain by immunoprecipitation [3]. Triplicate samples were analysed by quantitative mass spectrometry to permit a direct comparison of the interactomes of MoPrP and BVPrP. The interactome analysis was conducted twice, using two different anti-PrP antibodies recognizing distinct epitopes, to ensure that all relevant interactions were captured.

**Results:** The molecular environment of BVPrP was very similar to that of MoPrP. Many known MoPrP interactors were found in our experiments, including NCAM, 4F2, basigin, as well as subtle interactors such as ZIP6 and ZIP10. We found that there was significant overlap between the top interactors found in the BVPrP and MoPrP samples. However, we also identified several proteins that were reproducibly enriched in the samples derived from BVPrP ki mice. These hits are currently being validated using cultured cell models.

**Conclusions:** We conclude that the molecular environments of BVPrP and MoPrP in the brains of mice are very similar. Nonetheless, several proteins appear to preferentially interact with BVPrP. These proteins may help to explain the unique behaviour of BVPrP.

**KEYWORDS:** Bank vole; mass spectrometry; interactome; species barrier

### 

References[1]Watts et al., PLoS Pathog. 2014;10(4):e1003990.10.1371/journal.ppat.1003990PMC397487124699458[2]Agrimi et al., PLoS Pathog. 2008;4(7):e1000113.1865463010.1371/journal.ppat.1000113PMC2453331[3]Schmitt-Ulms et al., Nat Biotech. 2004;22(6):724–731.10.1038/nbt96915146195

## 207. Neurodegeneration in the enteric nervous system in an MPTP induced model of Parkinson’s disease

Laura J Ellett^a^^,^^b^^,^^c^ David I Finkelstein^c^ and Victoria A. Lawson^a^^,^^b^^,^^c^

^a^The Department of Pathology; ^b^The Department of Microbiology and Immunology; ^c^The Florey Institute for Neuroscience and Mental Health, The University of Melbourne, Victoria, Australia

**CONTACT** Victoria A. Lawson vlawson@unimelb.edu.au

**ABSTRACT**

The spread of α -synuclein pathology in Parkinson’s disease has been described as prion like with pathology thought to spread from the peripheral to central nervous system in at least some forms of the disease. Prion pathology and gastrointestinal dysfunction has also been reported in some forms of prion disease.

Following MPTP-lesioning, a well characterized model of Parkinson’s disease, C57BL/6 mice develop a constipation phenotype characterized by reduced stool frequency. We have recently correlated this dysfunction with enteric glial cell reactivity and the loss of specific neuronal populations in the enteric nervous system [1]. In the present study we investigated the mechanism of MPTP induced neurodegeneration in the enteric nervous system.

MPTP induced toxicity is dependent on the uptake of its toxic metabolite MPP+ by dopamine active transporter (DAT) expressed on dopaminergic neurons. DAT expression is highest in the ileum of the gastrointestinal tract. Consistent with this tyrosine hydroxylase (TH) immunoreactive (IR) dopaminergic neurons were significantly reduced in the ileum of MPTP-lesioned mice, but unaffected in the colon where DAT expression is lower.

Although MPTP-lesioning is not generally associated with Lewy pathology, MPTP-lesioning led to a change in localization of α -synuclein in DAT-IR neurons of the ileum. MPTP treatment did not affect α -synuclein localization in the colon.

Non-dopaminergic neurons identified by the expression of neurofilament (NFM-IR neurons) were also reduced in the myenteric plexus of the ileum, but not colon of MPTP lesioned mice.

We hypothesize that MPTP-initiated loss of non-dopaminergic cell populations be may in response to MPTP induced changes in α -synuclein and/or the response of enteric glial cells to MPTP induced injury. This hypothesis is consistent with a toxic insult triggering Parkinson’s disease pathology in a vulnerable cell population within the gastrointestinal tract, with secondary neurodegeneration caused by the spread of α -synuclein aggregates or neuroinflammation.

### 

Reference[1]Ellett et al., Sci Rep. 2016;6:30269.10.1038/srep30269PMC496586627471168

## 208. Binding prions to soils impact PrP^CWD^ recovery but not infectivity

Alsu Kuznetsova^a,b^, Debbie McKenzie^b^ and Judd M. Aiken^a^

^a^Agricultural Life and Environmental Sciences Faculty, University of Alberta, Edmonton, Canada; ^b^Faculty of Science, University of Alberta, Edmonton, Canada

**CONTACT** Alsu Kuznetsova alsu@ualberta.ca

**ABSTRACT**

Chronic wasting disease (CWD) is a contagious prion disease affecting cervids. Soil can act as an environmental reservoir resulting in CWD transmission. CWD prions shed from animals at the preclinical and clinical stages of disease into the environment remain infectious even after prolonged periods. In this study, we used soil minerals (quartz, illite, montmorillonite (Mte)) as well as six surface soil horizons of four different soils: horizons LFH (plant litter horizon) and Ae (illite-enriched mineral horizon) of Luvisol, horizons LFH (plant litter) and Bf (illite and iron enriched mineral horizon) of Brunisol, and Ah (humic horizon) horizons of two Chernozemic soils. Collected soil samples represent soil cover of boreal, tundra (Luvisols and Brunisols) and prairie regions (Chernozems).

Long-term incubation of prions with illite, Mte and soils results in decreased recovery of PrP^CWD^. Binding of prions to minerals and soils became more avid and irreversible with time; PrP signal on immunoblots continuously declined until it was no longer detectable after 30 weeks incubation in soils with loamy-clay texture and Mte minerology. This continual decline of PrP^CWD^ immunoreactivity did not correlate with prion infectivity levels as no significant differences were found in incubation periods between mice intraperitoneally inoculated with pure 1% brain homogenate and with the CWD-BH pre-incubated with quartz or Luvisolic Ae horizon for 1 and 30 weeks. After 55 weeks incubation with Chernozem and Luvisol, bound PrP^CWD^ was undetectable by immunoblotting but remained infectious.

We show that prions bind to soils and remain infectious after extended periods of time (at least up to 55 weeks). Our findings indicate that analysis of environmental samples for PrP^CWD^ will be complicated as prolonged incubation and lack of detection by western blot does not correspond to a decrease in PrP^CWD^ infectivity.

## 209. Comparative analysis of the neuroinvasion of Chronic Wasting Disease strains (Wisc-1 and H95^+^) in transgenic mice expressing different cellular prion proteins

Danielle Gushue^a^, Camilo Duque Velásquez^a^, Chiye Kim^a^, Judd Aiken^b^ and Debbie McKenzie^a^

^a^Department of Biological Sciences, Centre for Prions and Protein Folding Diseases, University of Alberta, Edmonton, Canada; ^b^Department of Agricultural, Food and Nutritional Sciences, Centre for Prions and Protein Folding Diseases, University of Alberta, Edmonton, *AB*, Canada

**CONTACT** Danielle Gushue dgushue@ualberta.ca

**ABSTRACT**

Chronic wasting disease (CWD) is a contagious prion disease of free-ranging and captive cervids from North America, Scandinavia, and Korea. The transmission cycle of CWD among cervids relies on the lymphotropic nature of CWD prions and the environmental persistence of prion infectivity shed by affected animals. Multiple prion strains circulate in CWD enzootic areas and their diversity is modulated by disease transmission between cervids expressing different PrP^C^ molecules. Prion strains neuroinvade with different efficiency and this process is dependent on the route of exposure, the infectious dose and the structural compatibility between prion conformation and host PrP^C^. To evaluate if the route of exposure affects strain adaptation, we compared the neuroinvasion properties of pure Wisc-1 strain and a mixture of this one with the H95^+^ strain. We previously demonstrated these strains have distinct biological properties, including conformational differences, brain targeting areas, and transmission host range. Two transgenic mouse lines expressing PrP^C^ allelotypes, Q95G96 or Q95S96, were exposed either orally or intraperitoneally (IP) and the phenotypic properties produced by each strain-route-host combination were compared. Consistent with previous observations in host expressing wt PrP^C^, the Wisc-1 strain had higher disease penetrance and shorter incubation periods compared to the H95^+^. The intraperitoneal route was more efficient than the oral route, which lacked complete attack rates. The incubation period of Wisc-1 doubled compared to the intracranial (IC) route. Interestingly, the differences in incubation between the host exposed to Wisc-1 or the H95^+^ mixture were smaller than previously reported by IC route. The Wisc-1 host-specific titre in the H95^+^ mixture is at least two-fold lower than pure Wisc-1, suggesting the route of exposure influenced strain selection from the mixture. This also indicates Wisc-1 is highly adapted for neuroinvasion of deer expressing wt-PrP^C^. With the potential for new prion strains to emerge and the presence of multiple strains in a single host, it is important to characterize their transmission properties, including their capacity to neuroinvade and their tissue tropism.

## 210. Neurofilament light chain (NfL) as a possible biomarker for drug efficacy in mouse models of neurodegenerative diseases

Masakazu Hirouchi^a^^,^^b^*, Atsushi Aoyagi^a^^,^^b^, Annalise Bond^a^, Kurt Giles^a^^,^^c^, Carlo Condello^a^^,^^c^ and Stanley B. Prusiner^a^^,^^c^

^a^Institute for Neurodegenerative Diseases, Weill Institute for Neurosciences, University of California, San Francisco, CA, USA; ^a^Daiichi Sankyo Co., Ltd, Tokyo, Japan; ^c^Department of Neurology, University of California, San Francisco, CA, USA

**CONTACT** Masakazu Hirouchi hirouchi.masakazu.m7@daiichisankyo.co.jp

**ABSTRACT**

Neurofilament light chain (NfL) is known as a promising fluid biomarker of neurodegenerative disease progression. NfL changes in plasma have been found in humans with neurodegenerative diseases (NDs) and transgenic (Tg) mouse models. Here, we report an elevation of plasma NfL in Tg mice modelling a synucleinopathy caused by multiple system atrophy (MSA). Brain extracts from deceased MSA patients caused disease in Tg(SNCA*A53T) mice expressing mutant α-synuclein(A53T). NfL levels in plasma correlated with α-synuclein prions and phosphorylated α-synuclein in the brain. By measuring NfL in plasma, we were able to predict the life span of Tg(SNCA*A53T) mice inoculated with MSA brain homogenates. Our findings suggest that plasma NfL levels can be used to predict disease progression in Tg mouse models of NDs. Preclinical methods for measuring neurodegeneration in Tg mouse models are valuable systems for assessing efficacy in therapeutic studies. To investigate this idea further, we employed a PrP disease model in which RML prions are used to infect FVB mice, which were treated with the aminothiazole IND24, a previously published anti-prion compound. Plasma NfL levels increased in inoculated FVB mice without treatment, but NfL was suppressed in mice treated with IND24, which was consistent with their extended survival. We are currently measuring PrP^Sc^ in brain and NfL in plasma levels in scrapie prion–infected FVB mice to understand the relationship between drug target and biomarker in our study.

## 211. Prion infection dysregulates retromer mediated trafficking

Govinda Sharma, Li Lu and Sabine Gilch

Department of Ecosystem and Public Health, Calgary Prion Research Unit, Faculty of Veterinary Medicine, Hotchkiss Brain Institute, University of Calgary

**CONTACT** Govinda Sharma govinda.sharma@ucalgary.ca)

**ABSTRACT**

**Background**: Vesicle trafficking is impaired in most of the neurodegenerative diseases caused by protein misfolding. We have previously reported that membrane bound Rab7 is reduced in prion infected cells. Rab7 regulates the trafficking from early endosome to late endosomes. Similarly, retromer complex mediates the trafficking between endosomes and the Golgi apparatus. It has been reported that the VPS35, a component of retromer complex, is downregulated in the brains of Creutzfeldt Jacob disease (CJD) patients. Here we studied how prion infection affects the retromer based trafficking in cultured cells and analysed the levels of VPS35, in prion infected mice as well as cultured cells.

**Methods**: Non-infected or prion-infected (22L-CAD5) cells were allowed to bind fluorescently labeled Cholera toxin subunit B (CTB) for 30 min at 4°C. Then the transport of CTB was chased at 37°C at different time points followed by immunostaining with Rab5 or Rab6 to visualize early endosomes or the Golgi complex, respectively. Confocal microscopy (Zeiss LSM700) and the software Zen were used to analyse the colocalization of CTB with Rab5 or Rab6. Similarly, the intracellular localization of VPS35 in 22L- or non-infected Neuro2A (N2A) was studied using immunofluorescent staining and confocal microscopy. Prion infection was confirmed by assessing the presence of proteinase K (PK) resistant prion protein (PrPSc) in cell lysates. Western blot analysis was used to evaluate the levels of VPS35 in non-infected or 22L-N2A cells, as well as in brains of 22L prion- or mock (PBS)-inoculated mice 125 days post intracerebral infection (dpi).

**Results**: We observed that GM1, a sphingolipid present in the lipid raft, is upregulated in prion-infected cells. CTB binds to GM1 and follows the endocytic route to the Golgi via retromer-mediated transport. We found that CTB transport from Rab5 positive early endosomes to the Golgi is delayed in prion-infected CAD5 as well as N2A cells. Also, the intracellular localization of the retromer component VPS35 is different in prion-infected N2A cells when compared to non- infected cells. It has been reported that VPS35 levels are downregulated in CJD patient’s brain. We found a similar decrease in VPS35 levels in 22L-CAD5 cells as well as in brains of mice inoculated with 22L prions when compared to mock (PBS) inoculated controls at 125 dpi.

**Conclusion**: These findings indicate that prion infection dysregulates retromer mediated trafficking, potentially due to the decreased VPS35 levels. The dysregulation of intracellular vesicle trafficking could be a mechanism of neuronal damage caused by prion infection and represents a novel therapeutic target.

## 212. Effects of E200K genetic predisposition to prion disease on cardiomyocyte cellular function, cytoskeleton structure, and protein expression

Aleksandar Wood^a^, Simote Foliaki^a^, Bradley Groveman^a^, Jue Yuan^b^, Leslie Cooperman^b^, Paul Tesar^b^, Wen-Quan Zou^b^ and Cathryn Haigh^a^

^a^National Institute of Allergy and Infectious Diseases (Laboratory of Persistent Viral Diseases); ^b^Case Western Reserve University School of Medicine

**CONTACT** Aleksandar Wood aleksandar.wood@nih.gov

**Background/Introduction**: The prion protein (PrP) has been shown to have a role in cellular protection against oxidative stress in numerous cell types, including murine heart tissue. Likewise, cardiac autonomic impairment has been linked with prion disease. In genetic Creutzfeldt-Jakob Disease (CJD) caused by the E200K single amino acid mutation, intraneuronal PrP deposition is found in the brainstem, which may influence cardiovascular autonomic control. However, the direct functional influence of the E200K mutation on cardiomyocytes has not been investigated. We hypothesized that the E200K mutation may cause a loss of PrP functional capacity resulting in increased oxidative damage and deficient function.

**Materials and Methods**: Cardiomyocytes were differentiated from several human induced pluripotent stem cell lines. The underlying cells were either ‘normal’ (no predisposition to dementia), contained the E200K mutation within the prion gene (predisposed to developing genetic CJD), or trisomy 21 (causing Down’s Syndrome in the donor). Baseline electrophysiological recordings were collected, measuring peak-to-peak amplitude as well as changes in overall channel activity over time. Experimental treatments were added and recordings were continued at specified time intervals, spanning from hours to days. Treatments were used to stimulate mitochondrial function, which would be expected to increase oxidative stress, and to inhibit contractility, reducing workload and therefore reducing oxidative stress. Twenty-four hours post-treatment, protein expression levels were assessed via Western Blot and Immunofluorescence.

**Results**: Preliminary electrophysiology recordings of cardiomyocyte cultures revealed that contractility of the cells varied basally and was altered by stimulating mitochondria. Expression levels of the superoxide dismutase (SOD) family was assessed as an indication of increased load from oxidative stressors; higher levels of expression can indicate compensatory efforts to negate the increased stress. E200K cardiomyocytes revealed increased SOD2 expression compared with controls, as well as increased prion protein (PrP) expression and increased B-Cell Lymphoma 2 (BCL2) expression – an anti-apoptotic marker.

**Conclusions**: The function, structure, and protein expression of cardiomyocytes was influenced by the E200K mutation. An impaired response to oxidative stress was indicated by changes in the localization and expression of SODs, PrP itself, and the anti-apoptotic protein, BCL2.

**KEYWORDS:** Prion; oxidative stress; reactive oxygen species; neurodegeneration; spongiform encephalopathy; cardiomyocyte; iPSC

## 213. Prenatal noise stress aggravates cognitive decline and the onset and progression of β-amyloid pathology in a mouse model of Alzheimer’s disease

Zahra Jafari, Megan Okuma, Hadil Karem, Jogender Mehla, Bryan E. Kolb and Majid H. Mohajerani

Department of Neuroscience, Canadian Centre for Behavioural Neuroscience (CCBN), University of Lethbridge, Lethbridge, AB, Canada

**CONTACT** Zahra Jafari Zahra.jafari@uleth.ca

**ABSTRACT**

Environmental distresses occurring during the sensitive periods of early-life may exacerbate the vulnerability to develop physical and mental diseases in old age. Studies have shown the impact of prenatal stress (PS) on the endocrine development and reprogramming of hypothalamic-pituitary-adrenal (HPA) axis functions in association with cognitive development and susceptibility to neuropsychiatric disease. Long-term exposure to glucocorticoids can damage the brain and intensify the progression of Alzheimer’s disease (AD)-like neuropathological changes, especially in females. There is, however, less information as to the link between PS and the risk of developing AD pathology during the lifespan. Male and female APP^NL-G-F/NL-G-F^ offspring of dams exposed to gestational noise stress were compared with the control offspring in corticosterone alternations, cognitive and motor performances, and the onset age and development of amyloid beta (Aβ) plaques across age. The hyperactivity of the HPA axis, spatial learning, and Aβ development were sex-specific showing persistent high levels of stress and further memory loss in females than males, especially in PS mice. The Aβ deposition was started earlier, by 2–3 months, and exhibited a heightened progression in PS animals. The PS also created a long-lasting anxiety-like behaviour and impairment in cognitive function and motor coordination. The findings suggest PS as a risk to exacerbate AD-like neuropathological changes during the lifespan, with higher susceptibility of females. The findings were discussed according to the most likely mechanisms for the PS effects, i.e., dysregulation of the neuroendocrine system and the placenta by the PS.

**KEYWORDS:** Prenatal stress; Alzheimer’s disease; Aβ plaque; cognitive decline; motor impairment; prepulse inhibition; HPA axis

## 214. Identification of novel CWD strains

Debbie McKenzie^a^, Camilo Duque Velasquez^a^, Allen Herbst^a^, Elizabeth Triscott^a^, Jacques van der Merwe^a^, Samia Hannaoui^b^, Leonardo Cortez^a^, Sara Amidian^a^, Valerie Sim^a^, Holger Wille^a^, Hermann Schaetzl^b^, Sabine Gilch^b^ and Judd Aiken^a^

^a^Centre for Prions and Protein Folding Diseases, University of Alberta, Edmonton, *AB*, Canada; ^b^Calgary Prion Centre, University of Calgary, Calgary, *AB*, Canada

**CONTACT** Debbie McKenzie Debbie.mckenzie@ualberta.ca

**ABSTRACT**

Chronic wasting disease is a set of prion diseases infecting captive and wild cervids. As different CWD agents are transmitted between different cervid species and between the same species having different Prnp polymorphisms, novel CWD strains can be generated. These new strains can exhibit different biochemical properties as well as different conformers. Identification of novel CWD strains is critically important as the different strains may vary in their host ranges, increasing the potential for transmission to economically important species as well as zoonotic transmission.

A rate limiting step in the characterization of CWD strains is the identification of deer samples that potentially contain novel strains. Much of the strain generation likely occurs during early passage between different Prnp genotypes (of the same or different species). Traditionally these differences have been identified following passages into rodent models (strains of lab mice, transgenic mice expressing different Prnp sequences and/or hamsters). Incubation periods can be long and the number of potential isolates is high making transmission experiments a slow, tedious, and expensive process. To streamline the process, we have identified a panel of *in vitro* analyses to help target samples for further characterization. For some isolates, potential new strains have been identified by western blot analysis, others by cervid cell assay, changes in the Prnp sequence, folding and/or aggregation differences as well as ability to seed reactions in RT-QuIC. Isolates of interest are then further characterized by transmission in a variety of different tg mouse lines, wild-type mice, hamsters, and voles. Using these criteria, five potential CWD strains have been identified.

## 215. Water quality improvement agent (new type CAC-717) also asts as prion disinfectant

Takashi Onodera^a^, Koichi Furusaki^b^, Makoto Haritani^a^ and Akikazu Sakudo^c^

^a^University of Tokyo; ^b^Mineral Activation Technical Research Center; ^c^University of Ryukyus

In Prion 2018 we reported electrically charged material (CAC-717) by continuously applying an electric field to mineral water containing calcium hydrogen carbonate. A Teflon insulation-coated electrostatic field electrode (N-800N, Mineral Activation Technical Research Center; Japan Patent No. 5864010) was used to create the electric field at a voltage of 2 × 10^4^ V for 48 h. CAC-717 solution in distilled water (Japan Patent No. 5778328, FDA/USA Regulation No. 880.6890 Class 1 disinfectant) had a pH of 12.39 ± 0.03 and contained calcium hydrogen carbonate particles (1,120 mg/L) and carbonate complex microparticles (50–500 nm) with a mesoscopic structure. CAC-717 works as a soil amendment was evaluated by growth of green tea leaves in Japanese farm lands. In Prion2019 we report that CAC-717 absorbed by ceramics were applied to fresh or sea water to work as water quality improvement agents.

Mouse prion (Chandler) infected N2a cell (ScN2a) lysate was subsequently mixed with equal volume of CAC-717 solution. As a negative control, uninfected N2a cell lysate was diluted 2-fold with PBS or RIPA buffer. 200 μg lysate protein was diluted in 100 μg of buffer (PBS or RIPA) and mixed with 100 μℓ of PBS. Mixtures were incubated at room temperature for 1 h then immediately diluted in PBS before determining the amount of prion in each sample for Western blotting with SAF83 monoclonal antibody. Prion was not detected in the ScN2a cell line lysate mixed with CAC-717 solution after testing with Western Blotting. However, PrP^C^ levels was intact in both uninfected N2a cell and infected ScN2a cells, detected by Western Blotting.

CAC-717 ceramics placed inside concrete blocks were sprayed into sea water by the seashore of Shimane Prefecture, Japan. Then alteration of water environment was observed for 6 months. Number of sea urchin, sea cucumber, and abalones were calculated inside the concrete blocks with CAC-717 ceramics. Yield, as calculated by the number of these marine animals, was then determined. No animal was observed in the blocks with ceramics without CAC-717. While large amounts of animals were attracted and growing inside the blocks with CAC-717 ceramics.

Evaluation of water environment, nutrient content, composition, and microbiological activity tend to increase following use of water quality improvement agents. Numerous studies have therefore to be examined for the effect of water quality improvement agent, beside animal yield and quality. Our future aims are to determine the microbiological activity of CAC-717 ceramics after spraying with CAC-717.

## 216. Molecular dynamics study of PrP conversion

Lyudmyla Dorosh^a^^,^^b^, Min Wu^a^, Sandipan Chakraborty^c^, Holger Wille^d^^,^^e^ and Maria Stepanova^a^^,^^b^

^a^Department of Electrical and Computer Engineering, University of Alberta, Edmonton, Canada; ^b^National Institute for Nanotechnology NRC; Edmonton, Canada; ^c^Department of Microbiology, University of Calcutta, Kolkata, India; ^d^Department of Biochemistry, University of Alberta, Edmonton, Canada; ^e^Centre for Prions and Protein Folding Diseases, University of Alberta, Edmonton, Canada

**CONTACT** Lyudmyla Dorosh dorosh@ualberta.ca

**ABSTRACT**

Prion diseases are believed to be caused by a conversion of native cellular prion protein (PrP^C^) into infectious β-rich aggregates (PrP^Sc^). The structures of these aggregates and the molecular mechanisms of the conversion remain unknown, although numerous models for the structure of PrP^Sc^ have been suggested [1]. In order to elucidate mechanisms of the conversion, we have done extensive all-atom molecular dynamics (MD) simulations tackling early stages of this process.

We employed several β-helical models of PrP^Sc^. These include the recently proposed [2] 4-rung right-handed β-helical (RHBH) head-to-tail moPrP^Sc^(89–230) model based on cryo-EM and X-ray fiber diffraction data and corrected with MD simulations; hypothetical 4-rung RHBH head-to-head and head-to-tail moPrP^Sc^(89–230) models [3]; and a 4.5 rung left-handed β-helical (LHBH) head-to-head huPrP^Sc^(114–220) model built by computational threading of the PrP sequence onto published β-solenoid architectures. In simulations of the PrP^C^ conversion, matching native PrP^C^ constructs (solution NMR) were also included.

In our MD simulations of the above-mentioned PrP^Sc^ models, the head-to-tail RHBH dimer [2] showed a higher stability in comparison to other constructs. However in different dimeric models, a preference for head-to-head configurations was found. A greater structural stability and an increase of β-content were also found in the dimers in comparison to monomers. The β-content and morphology were somewhat less stable in the LHBH model than in the RHBH models.

Simulations of the PrP^C^ conversion containing native PrP^C^ and PrP^Sc^ revealed early unfolding events in PrP^C^. Head-to-head contact of PrP^C^ with a RHBH PrP^Sc^ resulted in unfolding and formation of β-strands distant from the contact area, in regions of loop LH2H3 and helices H2 and H3, in first 100 ns. These changes remained stable for the next 150 ns. In systems with two PrP^C^ molecules, similar trends towards partial unfolding of H2 and H3 with formation of β-strands were observed. During 155 ns simulations of native PrP^C^ in a head-to-tail contact with monomeric RHBH PrPSc [3], a pronouncedly better connection was observed. Distinct from other systems, the S1S2 bundle of PrP^C^ approached the β-solenoid, partial unfolding of helix H1 occurred, and some of secondary structure around the loop LH2H3 was lost. However, our results suggest that fast conversion of PrP^C^ has a low probability. The connection between S1, S2, and H2H3 bundle is not interrupted, stabilizing the remaining secondary structure in PrP^C^.

### 

References[1]WilleRequena
Prion. 2014;8:60–66.10.4161/pri.28368PMC703090624583975[2]Spagnolli
et al., bioRxiv 2018; Preprint
10.1101/505271.[3]RequenaChakraborty, personal communication.

## 217. The presence of autoantibodies against the prion protein is independent of *PRNP* mutations

Karl Frontzek^a^, Manfredi Carta^a^, Marco Losa^a^, Mirka Epskamp^a^, Georg Meisl^b^, Ulrike Camenisch^c^, Tuomas Knowles^b^, Simone Hornemann^a^ and Adriano Aguzzi^a^; the THAUTAN-MC Investigators

^a^Institute of Neuropathology, University of Zurich, Zurich, Switzerland; ^b^Department of Chemistry, University of Cambridge, Cambridge, UK; ^c^Institute of Surgical Pathology, University of Zurich, Zurich, Switzerland

**CONTACT** Karl Frontzek karl.frontzek@usz.ch

**ABSTRACT**

**Background**: Prion diseases are incurable diseases of the central nervous system, which not only occur as sporadic and infectious forms, but can also be transmitted through the germ line as autosomal dominant traits. What is more, genetic prion diseases usually manifest in late age, indicative of protective factors. Here we reasoned that subtle conformational alterations of pathogenic prion protein (PrP^C^) variants could stochastically generate immunogenic *neo*-epitopes, raising the question whether such protective factors may consist, at least in part, of anti-PrP^C^ autoantibodies. We therefore conducted an extensive search for such autoantibodies in persons carrying pathogenic *PRNP* mutations and, for control, in their unaffected relatives.

**Methods**: In this case-control study, we have collected blood samples from individuals (*n* = 134) expressing a wide spectrum of disease-associated variants of the *PRNP* gene. Individuals with a positive family history of genetic prion disease (*n* = 79) but lacking disease-associated *PRNP* mutations served as controls. Antibody reactivity was measured using an indirect ELISA for the detection of human IgG_1-4_ antibodies against full-length human prion protein. We evaluated the effects of age, gender, *PRNP* mutations, *PRNP* codon 129 polymorphism, clinical signs of prion disease, and total IgG levels on autoantibody reactivity. In a subset of patients, autoantibody reactivity was assessed longitudinally for up to 2 years after baseline measurements.

**Results**: We found that antibody reactivity was present in a subset of both *PRNP* mutation carriers and controls. Elder individuals were at lower odds for the presence of anti-PrP^C^ autoantibodies, independently of total IgG levels or *PRNP* genotype. Gender, clinical signs of prion disease, pathogenic *PRNP* variants or p. 129 polymorphism did not influence autoantibody reactivity. Moreover, levels of anti-PrP^C^ IgG were stable over up to 2 years.

**Conclusions**: This study shows that pathogenic *PRNP* variants do not notably stimulate antibody-mediated anti-PrP^C^ immunity. Moreover, anti-PrP^C^ IgG autoantibodies are independent of overt signs of prion disease. The presence of anti-PrP^C^ autoantibodies in the general population without any disease-specific association suggests that relatively high titers of naturally occurring antibodies are tolerated. This observation suggests that such antibodies, if administered to humans, may not induce severe adverse reactions and may therefore serve as anti-prion therapeutics.

## 218. Single-molecule studies show α-synuclein aggregation is inhibited by anti-prion compounds and novel computationally-derived ligands

Chunhua Donga^a^, Lindsay Shearera^a^, Craig R. Garena^a^, Pascal Mercierb^b^, Nils O. Petersenc^c^, and Michael T. Woodsidea^a^

^a^Department of Physics, University of Alberta, Edmonton, AB, Canada; ^b^NANUC National High Field Nuclear Magnetic Resonance Centre, Edmonton, AB, Canada; ^c^Department of Chemistry, University of Alberta, Edmonton, AB, Canada; Edmonton, AB, Canada

**ABSTRACT**

The intrinsically disordered protein α-synuclein (αS) is enriched in neurons and has been shown to be linked to Parkinson’s disease (PD) and other related neurodegenerative disorders. Single αS molecules are postulated to undergo a misfolding/aggregation cascade that ultimately yields Lewy bodies, the histopathological hallmark of PD. The main neurotoxic species in this process have been shown experimentally to be pre-fibrillar oligomeric intermediates. We have investigated the inhibitory effects of various ligands on αS aggregation using single-molecule fluorescence cross correlation spectroscopy techniques and traditional ensemble ThT assays. These methods monitor respectively the influence of the ligands on the nucleation of small oligomers and the formation of fibrils. We characterized several inhibitors of αS aggregation from among known anti-prion agents and from a small library of compounds that were predicted to inhibit dimer formation based on computer simulations of αS dimer structures elucidated using single molecule force spectroscopy measurements of dimer aggregation. The ability of the same ligands to inhibit PrP aggregation and αS aggregation suggests there may be common features in the aggregation mechanisms that can be targeted for inhibition. The success of novel aggregation inhibitors discovered through computational simulations suggests that this approach provides a viable way to search for new inhibitors. Looking forward, we will broaden our search for additional novel candidates for development of PD therapeutics by testing αS with known inhibitors of other proteins that form amyloids (Aβ, tau, huntingtin, prion etc.), and by expanding the in silico discovery platform.

## 

References[1]Spillantini et al.
PNAS USA. 1998;95:6469-73.10.1073/pnas.95.11.6469PMC278069600990[2]Masliah et al. Science. 2000;287:1265-69.10.1126/science.287.5456.126510678833[3]Volles et al. Biochem. 2003;42:7871-8.10.1021/bi030086j12834338[4]Schwille et al. Biophys. J. 1997;72:1878–86.[5]Saeed. Et al. Am. J. Clin. Pathol. 1967;309:274-84.[6]Priola et al. Science 2000;287:1503-6.10.1126/science.287.5457.150310688802[7]Dee et al. Biochim. Biophys. Acta. 2012;1824:826-32.10.1016/j.bbapap.2012.03.01122480824

## 219. Domain-specific quantification of PrP in cerebrospinal fluid by targeted mass spectrometry

Eric Vallabh Minikel^a,b,c^, Eric Kuhn^d^, Alexandra Cocco^d^, Sonia M Vallabh^a,b,c^, Chrissy Hartigan^d^, Andrew G Reidenbach^a^, Franc Llorens^e,f^, Inga Zerr^e^, Sabina Capellari^g,h^, Piero Parchi^g,i^, Stuart L. Schreiber^a,j^, Steven A. Carr^d^

^a^Center for the Science of Therapeutics, Broad Institute of MIT and Harvard, Cambridge, MA, USA; ^b^Program in Biological and Biomedical Sciences, Harvard Medical School, Boston, MA, USA; ^c^Prion Alliance, Cambridge, MA, USA; ^d^Proteomics Platform, Broad Institute of MIT and Harvard, Cambridge, MA, USA; ^e^National Reference Center for TSE, Georg-August University, Göttingen, Germany; ^f^Biomedical Research Networking Center on Neurodegenerative Diseases (CIBERNED), L’Hospitalet de Llobregat, Barcelona, NA, Spain; ^g^IRCCS – Institute of Neurological Sciences, Bologna, Italy; ^h^Department of Biomedical and Neuromotor Sciences, University of Bologna, Bologna, Italy; ^i^Department of Experimental, Diagnostic and Specialty Medicine, University of Bologna, Bologna, Italy; ^j^Department of Chemistry & Chemical Biology, Harvard University, Cambridge, MA, USA

**CONTACT** Eric Vallabh Minikel eminikel@broadinstitute.org

**ABSTRACT**

Antisense oligonucleotides in preclinical development for prion disease will seek to lower PrP expression in the brain [1]. Trials of PrP-lowering therapies are likely to rely on quantification of PrP in cerebrospinal fluid (CSF) as a pharmacodynamic biomarker and possibly as a trial endpoint [1,2]. Studies using PrP ELISA kits have reproducibly shown that CSF PrP is lowered in the symptomatic phase of disease [2–6], a potential confounder for reading out the effect of PrP-lowering drugs in symptomatic patients. To date it has been unclear PrP’s lowered abundance in CSF results from its incorporation into plaques [7], retention in intracellular compartments [8], downregulation as a function of the disease process [9], or other factors. Because misfolding or proteolytic cleavage could potentially render PrP invisible to ELISA even if its concentration were constant or increasing in disease, we sought to establish an orthogonal method for CSF PrP quantification. We developed a targeted mass spectrometry method based on multiple reaction monitoring (MRM) [10] of nine PrP tryptic peptides quantified relative to known concentrations of isotopically labelled standards. Analytical validation experiments showed technical replicate coefficients of variation below 15%, strong dilution linearity and spike recovery, and applicability to both CSF and brain homogenate and across humans as well as preclinical species of interest. In *N *= 55 CSF samples from individuals referred to prion surveillance centres with rapidly progressive dementia, all six human PrP peptides, spanning the N- and C-terminal domains of PrP, were uniformly reduced in prion disease cases compared to non-prion diagnoses, replicating the findings from ELISA studies. Our study provides evidence that lowered CSF PrP concentration in prion disease is a genuine result of the disease process and not merely an artefact of ELISA-based measurement, and we provide a novel method suitable for preclinical and clinical quantification of CSF PrP as a tool for drug development.

### 

References[1]Vallabh, et al., In preparation.[2]Vallabh, et al., bioRxiv
2018 DOI:10.1101/295063.[3]Meyne, et al., J Alzheimers Dis.
2009;17:863.[4]Dorey, et al., JAMA Neurol. 2015;72:267.10.1001/jamaneurol.2014.406825559883[5]Abu Rumeileh et al, J Alzheimers Dis
2017;55:1471.10.3233/JAD-160740PMC518167727886009[6]Villar-PiqueSchmitz et al., NeurobiolMol
2018 DOI:10.1007/s12035-018-1251-1.[7]Parchi et al., Ann Neurol.
1999;46:22410443888[8]Goold, et al., J Cell Sci. 2013, 126, 3552.10.1242/jcs.120477PMC374402423813960[9]Mays, et al., J Clin Invest. 2014;124:847.10.1172/JCI72241PMC390462824430187[10]Carr, et al., Mol Cell Proteomics. 2014;13:907.10.1074/mcp.M113.036095PMC394591824443746

## 220. Light and electron microscopic studies of the 139A-H(Ha) strain of scrapie passaged in hamsters

Pawel P. Liberski0000-0001-6507-4682^a^, Agata Gajos0000-0002-3753-6197^b^, Beata Sikorska0000-0002-6166-0729^a^ and Janusz Moryś0000-0002-4048-1721^c^

^a^Laboratory of Electron Microscopy and Neuropathology, Department of Molecular Pathology and Neuropathology, Chair of Oncology, Medical University Lodz, Poland; ^b^Department of Extrapyramidal Diseases, Chair of Rehabilitation, Medical University of Lodz, Lodz, Poland; ^c^Department of Anatomy and Neurobiology, Medical University of Gdansk, Gdansk, Poland

**ABSTRACT**

We report here the light and electron microscopic neuropathology of the 139A-H strain of scrapie passaged in Syrian golden hamsters.

**Material and methods:** This strain was derived from the Scrapie Sheep Brain Pool (SSBP)-1 lineage [3,5]. Briefly, scrapie from a natural source was passaged through Cheviot sheep, Welsh Mountain sheep, goats, mice and led to the isolation of the ‘Chandler’ scrapie strain. The clone 139A strain was derived following passage in hamsters and this strain was made available for us by Dr. Richard I Carp, IBR, Staten Island, USA.

**Analysis of PrP immunostaining:** The intensity of the immunostaining were quantified in a single hamster brain using the images obtained from the AxioScan.Z1 system (Zeiss, Germany) using the Zeiss Zen 2.3 (Blue Edition) Software.

**Results:** The general neuropathological picture consisted of spongiform change and severe astrocytic gliosis. The topography of PrP was variable, the highest signal was observed in the CA2-molecular layer, CA1-pyramidal and entorhinal cortex. Double immunofluorescence stainings showed dot-like immunoreactivity of microtubule associated protein (LC3) in the cell bodies while axons more often showed fibrillar pattern. Interestingly, some neuronal processes showed only neurofilament protein and LC3 positive structures were depleted of NFP immunoreactivity. The electron microscopy consisted of:

1. Spongiform vacuoles – these are always membrane bound and contained secondary vacuoles (i.e. membrane-bound compartments or vesicles within vacuoles) and curled membraned fragments.
2. Tubulovesicular structures (TVS) – these are vesicular structures of approximate 27 nm in diameter within neuronal processes – i.e. axonal terminal or dendrites. TVS are smaller and of higher electron density than synaptic vesicles. The significance of TVS remains unknown.
3. Dystrophic neurites. Dendrites or axonal preterminals and terminals filled with electron-dense bodies, including small autophagic vacuoles.
4. Apoptotic cell nuclei.
5. ‘Whorls’, a concentric arrays of membranes were visible. A significance of those structures is unknown.

PPL and BS are supported by National Science Centre Poland, grant number UMO-2015/19/B/NZ4/03234

**KEYWORDS:** Scrapie; electron microscopy; topography of lesion

## 221. Purification of small, non-fibrillar and infectious prion particles from the brain of patients with GSS-A117V

Ilaria Vanni^a^, Laura Pirisinu^a^, Claudia Y. Acevedo-Morantes^b^, Michele A. Di Bari^a^, Claudia D’Agostino^a^, Stefano Marcon^a^, Geraldina Riccardi^a^, Elena Esposito^a^, Pierluigi Gambetti^c^, Umberto Agrimi^a^, Holger Wille^b^ and Romolo Nonno^a^

^a^Istituto Superiore di Sanità, Department of Food Safety, Nutrition and Veterinary Public Health, Rome, Italy; ^b^Centre for Prions and Protein Folding Diseases & Department of Biochemistry, University of Alberta, Edmonton, Alberta, Canada; ^c^Department of Pathology, Case Western Reserve University, Cleveland, Ohio, USA

**CONTACT** Ilaria Vanni ilaria.vanni@iss.it

**ABSTRACT**

Gerstmann-Sträussler-Scheinker disease (GSS) is an inherited prion disease associated with mutations in the PRNP gene. We recently reported that GSS with the A117V mutation, characterized by PrP^Sc^ with a protease resistant core made up of N- and C-terminally cleaved PrP peptides of ~7 kDa (7 kDa PrP^res^), is transmissible in bank voles. However, definite proof of the infectious nature of this truncated PrP^Sc^ type is still missing. We thus attempted to evaluate the GSS-A117V 7 kDa PrP^res^ role in terms of infectivity, strain characteristics, and structural features.

At first, we determined if the infectivity of GSS-A117V PrP^Sc^ is resistant to PK, by parallel end-point titrations in voles of PK-treated or untreated brain homogenates. Unexpectedly, GSS-A117V showed an extremely high infectious titer (10^9.3^ ID_50_ U/g), which was not significantly affected by PK treatment (10^9.0^ ID_50_ U/g). We then aimed at purifying the PK-resistant GSS-A117V prions by using a recently published protocol^1^ in order to determine the amount of infectivity and PrP^res^ in the different fractions, alongside with the morphological characteristics of purified PrP^res^ aggregates by EM.

Purified pellet fractions from GSS-A117V contained the expected N- and C-terminally cleaved 7 kDa PrP^res^, although the yield of PrP^res^ was lower than that of a scrapie sample used as a control. We found that this lower yield depended on the low density/small size of GSS-A117V PrP^res^, as it was mainly retained in the last supernatant fraction. All fractions were highly infectious, thus confirming the infectious nature of the 7 kDa PrP^res^, with infectivity levels that directly correlated with the PrP^res^ amount detected. Finally, EM analysis of these fractions showed no presence of amyloid fibrils, only very small and indistinct, non-fibrillar PrP^Sc^ particles were detected. In contrast, PrP^Sc^ fibrils with the expected morphology were recovered from the scrapie control sample.

Our study demonstrates that purified aggregates of 7 kDa PrP^res^ are fully infectious and encode the biochemical and biological strain features of the original sample, implying that the C-terminus of PrP^Sc^ is dispensable for infectivity and strain features. Finally, the non-fibrillar morphology of these unglycosyilated and non-GPI-anchored, small and indistinct prion particles, coupled to the very high levels of infectivity detected in GSS-A117V, is reminiscent of previous data on non-fibrillar prion particles as the most efficient initiators of TSE diseases [2]. Our findings provide new molecular and morphological constraints on the structure of infectious prions.

### 

References[1]Wenborn, et al., 2015.[2]Silveira, et al., 2005.

## 222. The impact of cellular prion protein expression levels in 5xFAD mouse behaviour and lifespan

Angela Silva-Correia^a^, Matthias Schmitz^b^ and Inga Zerr^c^

^a^Medical University of Göttingen; ^b^Georg-August University; ^c^National TSE Reference Center, Göttingen, Germany

**ABSTRACT**

Alzheimer’s disease is the most prevalent dementia causing progressive impairment of cognition, mood, speech, task performance ability, behaviour, and memory. Since all treatments for AD are primarily symptomatic, the development of disease modifying approaches continues to be an area of intense focus.

Previous studies already indicated an important role of the cellular prion protein (PrPC) as a regulator of the A β mediated toxicity by acting as a potential receptor. In this project we intended to evaluate different expression levels of PrPC and its impact of amyloid pathology in our animal model. We had already established three different bi-transgenic mouse line 5xFADPrnp^+^/^+^, 5xFADPrnp^±^ and 5xFADPrnp^−^/^−^ by crossing of PrPC knockout (Prnp^−^/^−^) mice with 5xFADPrnp^+^/^+^ mice (Tg6799) exhibiting mutations in the APP and presenilin genes. We observed a direct influence of PrPC expression levels on A β -induced behaviour deficits in our mice lines. 5xFADPrnp^−^/^−^ mice showed the biggest delay of behaviour impairment (cognition, anxiety or nest building) and 5xFADPrnp^+^/^+^ mice showed the fastest onset of behaviour impairment. Since a PrPC-knockout cannot prevent A β -induced deficits, we propose that further proteins and co-factor are involved in the uptake of A β and the accompanied cellular dysfunctions.

## 223. Scrapie in white-tailed deer: a strain of the CWD agent that efficiently transmits to sheep?

Justin J. Greenlee^a^, Robyn D. Kokemuller^a^, S. Jo Moore^a^ and Heather West Greenlee^b^

^a^Virus and Prion Research Unit, National Animal Disease Center, USDA, Agricultural Research Service, Ames, IA, USA; ^b^Department of Biomedical Sciences, Iowa State University College of Veterinary Medicine, Ames, IA, USA

**CONTACT** Justin J. Greenlee Justin.Greenlee@ars.usda.gov

**ABSTRACT**

Scrapie is a transmissible spongiform encephalopathy of sheep and goats that is associated with widespread accumulation of abnormal prion protein (PrP^Sc^) in the central nervous and lymphoid tissues. Chronic wasting disease (CWD) is the natural prion disease of cervid species, and the tissue distribution of PrP^Sc^ in affected cervids is similar to scrapie in sheep. There are several lines of evidence that suggest that multiple strains of CWD exist, which may affect the agent’s potential to transmit to hosts of the same or different species. We inoculated white-tailed deer with the scrapie agent from ARQ/ARQ sheep, which resulted in 100% attack rates by either the intracranial or oronasal route of inoculation. When examining tissues from the brainstems or lymphoid tissues by traditional diagnostic methods such as immunohistochemistry or western blots, it is difficult to differentiate tissues from deer infected with scrapie from those infected with CWD. However, there are several important differences between tissues from scrapie-infected white-tailed deer (WTD scrapie) and those infected with CWD (WTD CWD). First, there are different patterns of PrP^Sc^ deposition in the brains of infected deer: brain tissues from deer with WTD scrapie had predominantly particulate and stellate immunoreactivity whereas those from deer with WTD-CWD had large aggregates and plaque-like deposits. Secondly, the incubation periods of WTD scrapie isolates are longer than CWD isolates in mice expressing cervid prion protein. Most notably, the transmission potential of these two isolates back to sheep is distinctly different. Attempts to transmit various CWD isolates to sheep by the oral or oronasal routes have been unsuccessful despite observation periods of up to 7 years. However, WTD scrapie efficiently transmitted back to sheep by the oronasal route. Upon transmission back to sheep, the WTD scrapie isolate exhibited different phenotypic properties when compared to the sheep receiving the original sheep scrapie inoculum including different genotype susceptibilities, distinct PrP^Sc^ deposition patterns, and much more rapid incubation periods in transgenic mice expressing the ovine prion protein. The scrapie agent readily transmits between sheep and deer after oronasal exposure. This could confound the identification of CWD strains in deer and the eradication of scrapie from sheep.

## 224. NMR fragment screening to discover low molecular weight prion protein binders

Andrew Reidenbach, Eric Minikel, Sonia Vallabh, Alison Leed, Jenna Yehl, Jayme Dahlin, Florence Wagner, Chris Lemke, Colin Garvie, Virendar Kaushik, Mike Mesleh and Stuart Schreiber

Broad Institute of MIT and Harvard

**CONTACT** Andrew Reidenbach areidenb@broadinstitute.org

**ABSTRACT**

Prion disease is a rapidly progressive neurodegenerative disorder caused by misfolding and aggregation of the prion protein (PrP^C^), and there are currently no therapeutic options. Attempts to target and disrupt misfolded PrP (PrP^Sc^) have resulted in emergent drug resistance and molecules that lack efficacy on human prion strains. Ligands of PrP^C^ could antagonize prion formation by stabilizing the native protein or by targeting it for degradation, but no validated small-molecule binders have been discovered to date. We screened 6631 low molecular weight fragments for recombinant human PrP binding using ^19^F and saturation transfer difference (STD) NMR. 80 actives were re-tested using protein-observed transverse relaxation optimized spectroscopy (TROSY), an orthogonal 2D NMR method. A single benzimidazole derivative validated in concentration-response and its analogues exhibited a structure-activity relationship, albeit with weak affinity (*K*d >1 mM). The exceptionally low hit rate and weak affinity observed here suggest that PrP is a difficult target for small molecule binders. Nevertheless, our studies may provide a starting point for structural biology and medicinal chemistry efforts to identify more potent ligands through structure-based drug design.

**KEYWORDS:** Fragment screening; NMR; binders

## 225. Public acceptability of chronic wasting disease management approaches in Canada: a paired comparison approach

Geoffrey Durocher, Marty (M.K.) Luckert, Ellen Goddard and Vic (W.L.) Adamowicz

Department of Resource Economics and Environmental Sociology, University of Alberta, Edmonton, Alberta, Canada

**CONTACT** Geoffrey Durocher durocher@ualberta.ca

**ABSTRACT**

**Background**: Chronic Wasting Disease (CWD) is a prion disease found among both captive and wild populations of cervids and its prevalence has been increasing over time. As CWD spreads, a number of stakeholders are affected, including hunters, landowners, and the general public. When deciding upon management options for CWD, it is important to consider the preferences of the stakeholders involved. The goal of this research is to investigate the acceptability of CWD management approaches through the eyes of stakeholders.

**Methods**: We employ a paired comparison approach, where respondents indicate their preferences across a series of proposed pairs of CWD management options. Management options were developed based on practices that have been used in CWD-endemic areas and based on ideas expressed by focus groups that we conducted with decision-makers representing various stakeholder interests. We develop separate series of management options for the general public, hunters, and landowners. Responses were collected from approximately 5200 individuals across Canada using an internet-based survey and a sample from an online panel. Respondents were asked to choose between pairs of management options, while considering their preferences associated with the alternative approaches. After each pair of options, respondents were asked whether they preferred their chosen option, or no action be taken. Preferences were analysed using conditional logistic regressions-based on random utility models.

**Results**: Little heterogeneity existed in the preferences of different stakeholders from different demographic groups. For the most part, reducing deer numbers in high risk CWD areas through the increased use of hunters was preferred to using government sharpshooters. Likewise, restricting the movement of carcasses and hunted products was also generally positively received by most groups, with the notable exception of hunters. There were also options that were almost universally disliked. For example, increasing incentives for private landowners to allow hunting on their lands, by allowing them to charge hunters for access, was even disliked by landowners. We also find that most respondents prefer some action to inaction.

**Conclusions**: As CWD increases in spread and prevalence, policymakers are deciding which management options should be pursued. Our results provide policymakers with important information regarding public preferences for some management options that are available for CWD. When formulating management plans, consideration of these preferences could help establish and maintain public support for addressing CWD. With changing technology regarding the monitoring of CWD, new options are emerging that will warrant further inquiries into public preferences.

**KEYWORDS:** Chronic wasting disease; preferences; paired comparison

## 226. Efficient RT-QuIC seeding activity for α-synuclein in olfactory mucosa samples of patients with Parkinson’s disease and Multiple System Atrophy

Chiara Maria Giulia De Luca^a^, Antonio Emanuele Elia^b^, Sara Maria Portaleone^c^, Federico Angelo Cazzaniga^a^, Martina Rossi^d^, Edoardo Bistaffa^a^, Elena De Cecco^d^, Joanna Narkiewicz^d^, Giulia Salzano^d^, Olga Carletta^a^, Luigi Romito^b^, Grazia Devigili^b^, Paola Soliveri^b^, Pietro Tiraboschi^a^, Giuseppe Legname^d^, Fabrizio Tagliavini^e^, Roberto Eleopra^b^, Giorgio Giaccone^a^ and Fabio Moda^a^

^a^Unit of Neurology 5 and Neuropathology, Fondazione IRCCS Istituto Neurologico Carlo Besta, Milano, Italy; ^b^Unit of Neurology I – Parkinson and Movement Disorders Unit, Fondazione IRCCS Istituto Neurologico Carlo Besta, – Milano, Italy; ^c^Department of Health Sciences, Università degli Studi di Milano, Otolaryngology Unit, San Paolo Hospital, Milano, Italy; ^d^Department of Neuroscience, Scuola Internazionale Superiore di Studi Avanzati (SISSA), Laboratory of Prion Biology, Trieste, Italy; ^e^Scientific Directorate, Fondazione IRCCS Istituto Neurologico Carlo Besta, Milano, Italy.

**CONTACT** Fabio Moda fabio.moda@istituto-besta.it

**ABSTRACT**

Parkinson’s disease (PD) is a neurodegenerative disorder whose diagnosis is often challenging because symptoms may overlap with neurodegenerative parkinsonisms. PD is characterized by intraneuronal accumulation of abnormal α-synuclein in brainstem while neurodegenerative parkinsonisms might be associated with accumulation of either α-synuclein, as in the case of Multiple System Atrophy (MSA) or tau, as in the case of Corticobasal Degeneration (CBD) and Progressive Supranuclear Palsy (PSP), in other disease-specific brain regions. Their definite diagnosis can be formulated only neuropathologically by detection and localization of these aggregates, considered disease-specific biomarkers (DSB), in the brain. Compelling evidence shows that trace amount of DSB can appear in peripheral tissues, including receptor neurons of the olfactory mucosa (OM), but their concentration is well below the limits of detection of the conventional diagnostic techniques.

We have therefore exploited the ultrasensitive Real Time Quaking Induced Conversion (RT-QuIC) assay for the analysis of OM collected from patients with a clinical diagnosis of PD, MSA, CBD and PSP. Our results showed that PD and MSA samples induced stronger RT-QuIC seeding activity for α-synuclein compared to CBD and PSP samples. Notably, the final RT-QuIC aggregates obtained from MSA and PD samples demonstrated peculiar biochemical and morphological features useful for their discrimination. These results suggest that RT-QuIC represents an important tool for the diagnostic workup for PD and neurodegenerative parkinsonisms

**KEYWORDS:** RT-QuIC; olfactory mucosa; α-synuclein; Parkinson’s Disease; parkinsonisms

## 227. Investigating the effect of familial mutations in tau on the conformation and biological activity of tau prions

Victor Banerjee^a^, Gregory E. Mertz^a^, Eric Tse^a,b^, Nick A. Paras^a,c^, Amanda L. Woerman^a,c^, Daniel R.Southworth^a,b^,and Stanley B. Prusiner^a,b,c^

^a^Institute for Neurodegenerative Diseases, Weill Institute for Neurosciences, University of California, San Francisco, San 11865 Francisco, CA, USA; ^b^Department of Biochemistry and Biophysics, University of California, San Francisco, San Francisco, CA, USA; ^c^Department of Neurology, University of California, San Francisco, San Francisco, CA, USA

Abstract not available.

## 228. Cervid *Prnp* polymorphism at codon 116 generates new and distinct CWD strains

Samia Hannaoui^a^, Elizabeth Triscott^b^, Camilo Duque Velásquez^b^, Debbie McKenzie^b^, Sabine Gilch^a^

^a^Department of Ecosystem and Public Health, Calgary Prion Research Unit, Faculty of Veterinary Medicine; Hotchkiss Brain Institute; University of Calgary, Calgary, Alberta, Canada; ^b^Center for Prions and Protein Folding Diseases, University of Alberta, Edmonton, Alberta, Canada

**ABSTRACT**

Chronic wasting disease (CWD) is a prion disease of cervids. To assess the role of strain emergence and adaptation in CWD, we have characterized the impact of the 116 (A >G) polymorphism of the white-tailed deer (WTD) prion protein on CWD infection. Previously, we compared CWD prion isolates from WTD encoding wild-type (116AA) or polymorphic (116AG) PrP^C^. We found that 116AG-prions were conformationally less stable and more sensitive to proteases; infection of primary neuronal cultures with these isolates showed reduced infectivity associated with the WTD-116AG isolate and a lower seeding activity in a cell-free conversion assay. Molecular dynamics simulations suggested that the structure of 116G-PrP^C^ was more flexible than 116A-PrP^C^.

To understand the transmission and strain properties of the WTD-116AG isolate, we serially passaged it and the WTD-116AA (Wisc-1) prions in tg(CerPrP)1536^+/+^ mice overexpressing deer 116AA-PrP^C^. Upon the first passage, the 116AG isolate had a longer incubation period than the Wisc-1 isolate; interestingly, the differences in biochemical properties between Wisc-1 and WTD- 116AG were retained supporting the characterization of at least one new prion strain amongst the 116AG-prions.

Upon secondary passage, infectivity of 116AG-prions was significantly enhanced in 80% of the mice and the conformational features were retained. Remarkably, this passage allowed us to distinguish two populations of mice with a short and a long incubation period. Although on first passage, only one homogenous population was obvious, subsequent passages allowed a better distinction and characterization of two 116AG-prions (short and long incubation). Biological cloning in tg(CerPrP)1536^+/+^ mice through inoculation of 10-fold serial dilutions of the 116AA and 116AG deer brain homogenates confirmed the presence of a second strain in the 116AG isolate, yet the slow disease progression feature of the polymorphic isolate was still striking for both of the 116AG-prions (short and long incubation) compared to the rapid disease progression of the 116AA. Interestingly, upon deglycosylation, PK-resistant fragments with different electrophoretic mobility are observed. Further confirmation of two strains present in 116AG was obtained following passage of this inocula in Syrian Golden Hamsters. Ten of twelve hamsters infected with 116AG were positive for PrP^res^. Western blot analysis of PrP^res^ showed two different migration patterns, high molecular weight (7/10) and low molecular weight migration pattern (3/10). Our data support the hypothesis that two strains co-existed, as dominant and minor strains, in the 116AG deer.

Our findings give insight into the variability of CWD strains, which can have important implications in terms of their possible distinct capacities to cross species barriers into both cervid and non-cervids.

## 229. Aberrations of Amyloid-β in slow and rapidly progressive dementias

Aneeqa Noor^a^*, Saima Zafar^a^*, Hassan Dihazi^b^ and Inga Zerr^a^

^a^Prion Research Group, National Reference Center for TSE Surveillance, Department of Neurology, Georg-August University, Göttingen, Germany; ^b^Department of Nephrology and Rheumatology, Georg-August University, Göttingen, Germany

*These authors contributed equally to this work

**CONTACT** Aneeqa Noor aneeqa.noor@med.uni-goettingen.de

**ABSTRACT**

Amyloid-β (Aβ), one of the key players in Alzheimer’s disease (AD), has the capability to exist as various proteoforms [1]. Over the last decade the self-seeding capability and relative pathogenicity of these proteoforms has been an active target of research however, their distinct clinical involvement in neurodegeneration is yet to be elucidated. For this purpose, we aimed to decipher the prion-like structural and functional aspects of Aβ aggregation in rapidly progressive dementias.

We used affinity-based proteomic profiling to isolate Aβ from brains of AD, rapidly progressive AD (rpAD) and non-demented controls and established a signature of brain-derived proteoforms, adding to the evidence that Aβ_40_ and Aβ_42_ are not sole players in progression of AD. Our preliminary experiments reveal a differential involvement of 26 different proteoforms in AD and rpAD brain. Quantification of various Aβ cleaving enzymes in the same samples supported the generation of distinct proteoforms in AD and rpAD. To further understand the pathological significance of Aβ in slow and rapidly progressive dementias, we isolated interacting partners of Aβ from AD, rpAD, non-demented controls and Creutzfeldt-Jacob Disease (CJD) brains. In addition to commonly known partners like Tau and Cellular Prion Protein, we were able to isolate around 400 different proteins that showed a disease specific involvement. Primary targets were found to be involved in energy metabolism, neurotransmission, neuronal development and stress response. Assessment of the clinical relevance of these aberrations and interactions is currently underway in various AD models.

Our study presents a novel insight in the Aβ-based differences among slow and rapidly progressive dementias, primarily the rapidly progressive variant of AD. In addition to providing the evidence that rpAD features the involvement of discrete molecular machinery, it also provides insights into the players involved in placing dementias on a course of rapid progression.

### 

Reference[1]Portelius, et al.
*Acta Neuropathol*. 2010;120:185–193.2041930510.1007/s00401-010-0690-1PMC3568930

## 230. Critical structural and kinetic impact of cofactors on host-to-host transmissibility of human prions

Jiri Safar, Chase Kim, Shugui Chen, Tracy Haldiman, Vitautas Smirnovas, Krystyna Surewicz, Nicholar Maurer, Qingzhong Kong, Qinzhong Kong and Witold Surewicz

Case Western, Reserve Univeresity

**ABSTRACT**

**Objectives**: Molecular characteristics determining the serial host-to-host transmissibility of human prions causing Creutzfeldt-Jakob disease (CJD) are unknown, and several recent clinical therapeutic trials failed. The key reasons behind these disappointing results are (a) fundamental structural differences between human prions causing diseases and cloned laboratory prions, and (b) distinct replication mechanism of human prions that is not determined by their confromational stability, as it is in yeast and some murine prions, but by growth rate of prion aggregates, which is in turn controled by their specific structural features. How these aspects impact transmissibility of prions to man is an imperative question in light of history of iatrogenic and zoonotic transmissions and recent findings of human prions in the skin and body fluids.

**Methods**: We investigated the effect of distinct cofactors on the structure and replication kinetics of major human prion strains. Using these data, we synthesized with variable cofactors a series of new human prions from the recombinant human prion protein, and to asses the impact of glycosylation, monitorred their replication rate and infectivity in serial passage in transgenic mice that were expressing physiological levels of human prion proteins with or without glycosylation.

**Results**: We report that different lipid and polyanionic cofactors have a major structural and kinetic impact on the human prion replication *in vitro*, infectivity *in vivo*, and strain characteristics. Within the synthetic prion spectrum, recombinant human prion generated with ganglioside GM1 cofactor caused unique and stable neurologic dysfunction with incubation times comparable to natural CJD prion strain in both first and second passage, respectively. Unique hybrid MM1/MM2 CJD-like prion neuropathology, high replication rate of reisolated prions, and biophysical profiling indicate that a novel particularly infectious and aggressive human prion strain was created.

**Conclusions**: Lipid and polyanion cofactors have fundamental impact on human prion strain characteristics and transmisibility. Their seemingly subtle effects on structural organization of prions are coupled with large differences in replication rates, and together with posttranslational modifications, play a crucial role as determinants of infectivity, host range, and extraordinary precission in targetting specific brain structures.

## 231. Prp1 is necessary for seizure-susceptibility in *appa* zebrafish knockout and new *in vivo* anti-convulsants

Richard Kanyo^a^, Laszlo Locskai^a^, Patricia L. A. Leighton^a^, Harley T. Kurata^b^ and W. Ted Allison^a,c^

^a^Centre for Prions & Protein Folding Disease, and Department of Biological Sciences, University of Alberta, Edmonton, *AB*, Canada; ^b^Department of Pharmacology, University of Alberta, Edmonton, *AB*, Canada; ^c^Department of Medical Genetics, University of Alberta, Edmonton *AB*, CANADA

**CONTACT** Richard Kanyo rkanyo@ualberta.ca

**ABSTRACT**

**Background**: Cellular prion protein (PrPC) has been linked to Alzheimer’s disease (AD), the most prevalent dementia. PrPC directly interacts with amyloid-β oligomers and amyloid precursor protein (APP) and the genetic interaction plus mechanism was further explored here. Because 10–22% of AD patients display epileptic episodes, potential anti-convulsants, ML213 and ICA73, which target Kv7.2/3 channels have been investigated *in vivo*. Zebrafish enable economical high-throughput drug screening platforms that faithfully represent the complexity of the central nervous system and are therefore an excellent model for these goals.

**Methods**: *appa;prp1* compound mutant (*Appa* and *Prp1* are disrupted) fish were engineered by targeted mutagenesis. Seizure-susceptibility was compared in mutant and wild type larvae via treatment with convulsants, pentylenetetrazol (PTZ) or 4-aminopyridine (4AP). Locomotor activity was tracked using Ethovision behavioural tracking software. Neural activity of free-swimming animals was measured using the novel ratiometric fluorescence-based Ca2+ indicator CaMPARI.

**Results**: PTZ dose-responses of locomotor activity revealed that *Appa* and *Prp1* single mutants exhibit enhanced seizure-susceptibility compared to wild type fish. However, seizure-susceptibility is dampened/reduced in compound mutants compared to either individual mutant. Measuring neural activity with CaMPARI, and using 4AP as a convulsant, ML213 and ICA73 were demonstrated to have anti-convulsant activity *in vivo*. ML213 was a very potent anti- convulsant, whereas ICA73 had similar potency to retigabine (RTG), an FDA approved drug used previously to treat epilepsy. When measuring locomotor activity, ML213 and RTG paralleled our results obtained with CaMPARI, but ICA73 did not and displayed an increase in locomotor activity highlighting the importance of using multiple assays to complement one another.

**Conclusion**: *appa* and *prp1* mutants both exhibited enhanced seizure-susceptibility. Interestingly, *prp1* function was required for maximum seizure-susceptibility in *appa* mutants suggesting a mechanism/pathway by which *prp1* functions as a rheostat of seizure-susceptibility or neural hyperactivity. Therefore a bi-functional mechanism may explain our recently published findings [1], where compound *prp1;prp2* mutants are more susceptible to seizures than *prp1* mutants, but less than *prp2* mutants. ML213 and ICA73 were established here as anti-convulsants *in vivo* and thus may provide a route in future mechanistic studies to gain sufficient insight and treat AD patients that display seizures. Together, this work using engineered zebrafish and anti-convulsants could illuminate the PrP-APP pathway as a potential aetiological link between AD and epilepsy.

### 

Reference[1]LeightonKanyo et al. JBC. 2018;293:12576–12592.10.1074/jbc.RA117.001171PMC609324429903907

## 232. ‘Novel compounds for the treatment of prion disease’

Robert C.C. Mercer^a^, Nhat T. T. Le^a^, Doug Fraser^b^, Aaron Beeler^b^ and David A. Harris^a^

^a^Boston University School of Medicine, Department of Biochemistry; ^b^Boston University, Department of Chemistry

**ABSTRACT**

Prion diseases are invariably fatal neurodegenerative diseases of humans and other animals for which there are no currently available treatments. Using a prion infected mouse neuroblastoma cell line (ScN2a) as an assay system, many anti-prion compounds have been identified, but none have resulted in a clinically effective therapy. Interestingly, while several anti-prion compounds exert their effects through a direct interaction with PrP^C^ or PrP^Sc^, the mechanisms underlying the anti-prion effects of other compounds remains enigmatic. A recent report from our laboratory [1] describes the identification of a novel class of anti-prion compounds, phenethyl piperidines. These were discovered using a high throughput screen for molecules that prevent mutant PrP toxicity. JZ107, the most potent derivative, can permanently cure ScN2a cells of infection and rescue hippocampal neurons from PrP^Sc^ induced synaptotoxicity *in vitro*. Further study of JZ107 has led, unexpectedly, to the discovery of a number of additional, novel anti-prion compounds, some of which have previously identified molecular targets and have been used in other clinical settings. None of these compounds bind directly to PrP, making the identity of their interaction partners of significant interest. To further validate the anti-prion effects of these compounds, we assessed their ability to prevent the retraction of hippocampal neuron dendritic spines following exposure to purified prion preparations. We have begun assessing the anti-prion effects of the most potent of these compounds *in vivo*.

### 

Reference[1]ImberdisT, et al.
Identification of anti-prion compounds using a novel cellular assay. *JBC*. 2016;291:26164–26176.10.1074/jbc.M116.745612PMC520708427803163

## 233. The many phases of prion protein as observed by NMR

Lauren E. Klein^a^, Mikail A. Kostelov^b^, Marcus D. Tuttle^a^, Stephen M. Strittmatter^b^ and Kurt W. Zilm^a^

^a^Department of Chemistry, Yale University, New Haven, Connecticut, USA; ^b^Department of Neurology, Yale School of Medicine, New Haven, Connecticut, USA

**CONTACT** Kurt W. Zilm kurt.zilm@yale.edu

**ABSTRACT**

Nuclear magnetic resonance (NMR) has proven invaluable in characterizing all sorts of functionally important macromolecular aggregates from amyloids to membrane-less organelles. In this contribution we describe the discovery and characterization by NMR of multiple phase states of prion protein [1] with relevance to Alzheimer’s disease (AD). The binding of amyloid-beta oligomers (A β o) to PrP^C^ has been demonstrated to lead to synapse loss and the onset of symptoms in an animal model of AD. Biophysical investigation of the mode of PrP^C^/A β o association found formation of a stoichiometric complex. When studied by magic angle spinning (MAS) NMR, this condensate was revealed to be a hydrogel phase. We find liquid-like mobility for PrP^C^ in the hydrogel, while the A β o instead behave as a relatively rigid molecular solid. Association of PrP^C^ to A β o is mediated via lysine clusters, which can be detected by observation of large differential mobility between bound and unbound lysines by ^15^N MAS NMR. Exploration of the PrP^C^/ A β o phase space further lead to discovery of a PrP^C^ rich viscous liquid phase, also studied by ^13^C MAS NMR methods.

Repetitive motifs and clustering of identical residues makes PrP^C^ a difficult target for NMR resonance assignment. Nevertheless, this same attribute makes it possible to directly associate many resonances with specific domains. Of the 210 aa in PrP^C^, 43 are glycines, with all but 3 located in the N-terminal 21–131 segment. Six of the 8 alanines are located in the palindromic AGAAAAGA run from 113–120, and 8 of the 12 threonines are located at the end of the junction between the C-terminal helices encompassing aa 183–201. Large scale conformational transitions can be detected by NMR when such aa clusters exhibit simultaneous shifts characteristic of a change in secondary structure. In this manner we observe both the N-terminal fragment and the palindromic sequence to transition from random coil to a largely helical conformation upon A β o binding. Interestingly, the formation of the PrP^C^ liquid phase is found to be accompanied by a melting of the threonine rich helical segment, and the apparent adoption of an unusual ^χ^_1_ for the tryptophan residues in the octa-repeat segment.

Biophysical characterization of these phase states of PrP^C^ is proving relevant to development of possible treatments for AD. Compounds that disrupt the hydrogel phase have been shown to relieve memory deficits in AD mice, and make it possible to detect the selective dissolution of the hydrogel phase in Alzheimer’s brain lysates [2].

### 

References[1]KostylevMA, et al. CellMol
2018;72(3):426–443.10.1016/j.molcel.2018.10.009PMC622627730401430[2]GuntherEC, et al. RepCell
2019;26(1):145–158.10.1016/j.celrep.2018.12.021PMC635872330605671

## 234. The European Union Summary Report On Surveillance For The Presence Of Transmissible Spongiform Encephalopathies (TSE): The Situation In 2017

Angel Ortiz Pelaez^a^, Valentina Rizzi^a^, Giuseppe Ru^b^, Francesco Ingravalle^b^ and Yves Van der Stede^a^

^a^Unit on Biological Hazards and Contaminants, Department of Risk Assessment & Scientific Assistance, European Food Safety Authority (EFSA); ^b^Biostatistics, Epidemiology and Risk Analysis (BEAR) Unit. Istituto Zooprofilattico Sperimentale di Piemonte, Liguria e Valle d ’Aosta, Torino (Italia)

**CONTACT** Angel Ortiz Pelaez angel.ortizpelaez@efsa.europa.eu

**ABSTRACT**

The European Food Safety Authority publishes a yearly summary of the surveillance activities on transmissible spongiform encephalopathies (TSE) in bovine animals, sheep, goats, cervids, and other species, in the European Union (EU), and in Iceland, Norway and Switzerland. Target groups include: animals clinically suspected of being infected by TSE; animals culled under TSE eradication measures; animals with clinical signs at ante-mortem; emergency slaughtered; fallen stock/not slaughtered for human consumption and healthy slaughtered animals for human consumption, for cervids also hunted and road or predator-injured or killed.

For the first time since bovine spongiform encephalopathy (BSE) had been reported, no cases of classical BSE were reported world-wide in 2017 [1]. Six atypical BSE cases were reported by Spain (1 H /2 L), France (1 H/1 L) and Ireland (1 L), out of the 1,312,714 cattle tested by 28 EU Member States (MS) and 18,526 tested by three non-MS.

In total 431,815 small ruminants were tested in 2017 in the EU. Compared with 2016, there was a 36.2% increase in the number of cases of classical scrapie (CS) in sheep (933), mostly reported by Greece, Spain, Italy and Romania, although over 75% of the cases were sourced in infected flocks. Atypical scrapie (AS) was confirmed in 94 animals. In goats, a decrease of 10% in the number of cases of classical scrapie (567) were reported, 84% in Cyprus. Atypical scrapie was confirmed in nine animals.

Ten-year trend analysis showed a statistically significant decrease in the sheep proportion of CS cases per 10,000 tested animals and an increase in goats. For AS, 10-year data did not detect any statistically significant trend in both species.

After the discovery of chronic wasting disease (CWD) in Norway in 2016, TSE testing in cervids increased in the EU: 10 MS tested 3,585 cervids (75% in Romania, 98.5% from wildlife), all negative. Norway tested 25,736 deer in 2017, leading to the detection of the first case of CWD in a red deer, nine cases in wild reindeer and one in a wild moose. Following EFSA recommendations, the European Commission introduced a 3-year mandatory surveillance programme for six member states starting on 1 January 2018.

By the time of submitting this abstract, CS/AS cases were not yet available, but one new classical BSE case was confirmed in Scotland (2018), one L-type BSE case in Poland (2019) and one case of CWD in a wild moose in Finland in March 2018.

**KEYWORDS:** BSE; scrapie; CWD; EU; surveillance

### 

Reference[1]EFSA (European Food Safety Authority), 2018
The European Union summary report on surveillance for the presence of transmissible spongiform encephalopathies (TSE) in 2017. EFSA J. 2018;16(11):5492, 64 pp. DOI:10.2903/j.efsa.2018.5492PMC700976532625769

## 235. Mutations in the functional amyloid PMEL lead to clinical glaucomatous neurodegeneration: Potential for insights into avoiding amyloidosis

W. Ted Allison^a^^,^^b^^,^^c^, Tim Footz^b^, Gavin J Neil^a^^,^^c^ and Michael A. Walter^b^

^a^Centre for Prions & Protein Folding Disease, U Alberta, Edmonton, *AB*, Canada; ^b^Department of Medical Genetics, U Alberta, Edmonton, *AB*, Canada; ^c^Department of Biological Sciences, U Alberta, Edmonton, *AB*, Canada

**CONTACT** W. Ted Allison ted.allison@ualberta.ca

**ABSTRACT**

Pigmentary glaucoma (**PG**) is a major form of glaucoma, a leading cause of blindness worldwide. Glaucoma is neurodegeneration of retinal ganglion cells, which are neurons of the CNS. We have, for the first time, discovered a causative gene for this CNS neurodegeneration in humans. Our results reveal that mutations of the premelanosome gene (*PMEL*) cause PG. We identified *PMEL* mutations in an Alberta family with PG and in four isolated patients. In parallel, our colleagues Drs. Wiggs (Harvard Medical School) and Craig (U Flinders, Australia), identified *PMEL* mutations in separate PG cohorts, strongly supporting that *PMEL* mutations cause PG (Lahola-Chomiak et al. 2018 *Human Molecular Genetics* doi: 10.1093/hmg/ddy429) [1].

Intriguingly, PMEL protein is a rarity in vertebrates: a **functional** amyloid. PMEL forms amyloid fibril structures in pigmented cells of vertebrates, including humans, and these amyloid fibrils are structurally critical for deposition of melanin pigment. The patient *PMEL* mutations we identified result in impaired processing of PMEL protein and altered amyloid structure, however the central question of how these changes lead to CNS neurodegeneration remains unknown. Our zebrafish modelling of *PMEL* loss via CRISPR/Cas9 mutagenesis produced recessive inheritance of swollen eye morphology in larvae that is strikingly reminiscent of increased ocular pressure in glaucoma; this implies that loss of the functional PMEL amyloid can contribute to some aspects of PG aetiology. This is in contrast with our patient pedigrees and mutations of *PMEL* homologs in other animals (dogs, horses, etc.) where dominant inheritance implies a toxic gain-of-function; this predicts that abnormal PMEL amyloid might lead to neurodegeneration via a (prion-like?) templating of aberrant amyloid structure.

Future work is warranted to delineate these contributions of gained toxicity vs. reduced protein function during (prion-like?) neurodegeneration [2], including defining whether both of these contributions are required for death of CNS neurons. Such work has potential to give unique insights into amyloidosis: We identify several mutations in patients with glaucomatous neurodegeneration, and these now guide our predictions of which aspects of PMEL are the key to human CNS cells thriving through a lifetime of high amyloid content.

### 

References[1]Lahola-Chomiak, et al
Hum Mol Genet. 2018 DOI:10.1093/hmg/ddy429[2]Allison, et al. Int J Mol Sci
2017;18:E2223
DOI:10.3390/ijms18102223

### 

SCHMITT-ULMS, Gerold 2019

## 236. A mechanism-based small-molecule approach to the treatment of prion diseases that targets PrP^C^

M Mehrabian^a,b^, D Williams ^a,b^, X Wang^a,b,c^, F Ghodrati^a,b,c^, M Güneş,^a,b^ and G Schmitt‐Ulms^a,b,c^

^a^Tanz Centre for Research in Neurodegenerative Diseases, University of Toronto, Toronto, Ontario, Canada; ^b^Krembil Discovery Centre, Toronto, Ontario, Canada; ^c^Department of Laboratory Medicine & Pathobiology, University of Toronto, Toronto, Ontario, Canada

**ABSTRACT**

A wide range of observations in humans and animals indicate that a reduction in steady‐state levels of the cellular prion protein (PrP^C^) is both safe and would delay or prevent prion diseases. To derive rational approaches for suppressing PrP^C^ levels at the plasma membrane, we and others have been studying the molecular environment and function of PrP^C^.

This work led us to recognize that prion genes evolved from a ZIP zinc transporter ancestral molecule with roles in a morphogenetic programme known as epithelial‐to‐mesenchymal transition (EMT). The latter guided us to investigate the possible involvement of PrP^C^ in EMT, revealing PrP^C^ to control the polysialylation of the neural cell adhesion molecule 1 (NCAM1), a well‐studied post‐translational modification that serves as a molecular marker of EMT. In several cell models we interrogated, the molecular environment of PrP^C^ is characterized by its residence within a specialized membrane domain that hosts, in addition to NCAM1, distinct subsets of more than 20 additional proteins. Interestingly, most of these proteins have known roles in the modulation of TGFB1 and integrin sister signalling complexes that are activated during EMT.

The binding of ligands to their cognate receptors often induces the internalization of ligand‐receptor complexes, followed by their recycling or degradation. We reasoned that such a turn of events may lead to a passive co‐internalization of PrP^C^, effectively reducing its levels at the cell surface. Our recent data reveal that PrP^C^ levels are indeed robustly reduced in the presence of non‐toxic nanomolar concentrations of a compound, hereafter referred to as KDC series 100 compound 2 (KDC102), which targets one of the proteins in proximity to PrP^C^. As anticipated, this reduction in PrP^C^ levels depends on the compound‐dependent degradation of the targeted receptor and can be observed in co‐cultures of human neurons and astrocytes. Although mechanistic insights are not essential for translation into the clinic, their existence should prove an asset going forward. Current efforts are directed towards testing KDC102 in proof‐of‐concept prion disease treatment studies in rodents.

## 237. A general mass spectrometry-based method of quantitating the relative amounts of PrP polymorphisms in CWD PrPSc

Christopher J. Silva^a^, Melissa L. Erickson-Beltran^b^, Camilo Duque Velásquez^c^, Judd Aiken^d^ and Debbie McKenzie^e^

^a^Produce Safety & Microbiology Research Unit USDA, WRRC, ARS; ^b^Produce Safety & Microbiology Research Unit, USDA, WRRC, ARS; ^c^University of Alberta, 120 Brain and Aging Research Building, Edmonton, AB T6G 2R3, Canada; ^d^Agricultural Sciences – Centre for Prions and Protein Folding Diseases – University of Alberta; ^e^Univeristy of Alberta

**ABSTRACT**

**Background/Introduction**: Chronic wasting disease (CWD) was first described in a captive cervid herd in 1967, recognized as a prion disease in 1978, and first detected in wild cervids in 1981. CWD is readily transmitted among animals and from a contaminated environment to uninfected animals. By 2019, 26 states in the United States, three Canadian provinces, South Korea, Norway, and Finland had found CWD in captive and/or wild cervid populations. Cervid PrP contains polymorphisms that influence progression of CWD and propagation of CWD strains with different transmission properties [1–3]. It is important to develop a method to quantitate the relative amounts of those polymorphisms present in CWD PrP^Sc^, so that their respective influence on CWD prion propagation can be determined.

**Materials and Methods**: Plasmids containing relevant cervid PrP polymorphisms were prepared. The necessary ^15^N-labelled internal standards were produced by overexpressing the PrP genes in *E. coli* grown in minimal medium with ^15^NH_4_Cl as the sole nitrogen source. Peptides derived from the tryptic or chymotryptic digestion of cervid recombinant PrP and containing the relevant polymorphisms were identified. A multiple reaction monitoring method (MRM) was developed for each of those peptides.

**Results**: Calibration curves relating the signal intensity of the various peptides and their absolute amounts as measure by protein assay were linear and had excellent correlation coefficients. In addition, samples from experimentally infected heterozygous deer (Q_95_**S**_96_/Q_95_**G**_96_ and **H**_95_G_96_ /**Q**_95_G_96_) were analysed and the relative amounts were determined. The optimal digestion for all tryptic peptides was 20 h. For chymotrypsin the optimal digestion time was also 20 h, apart from a single peptide.

**Conclusions**: This approach can be used to quantify the contribution of specific polymorphisms to PrP^CWD^ formation, enhancing our ability to determine the relative susceptibility of various polymorphisms to CWD. It can also be used to detect the distribution of CWD prion strains.

### 

References[1]Johnson CJ, et al. PLoS One
2011;6:e17450.10.1371/journal.pone.0017450PMC306081621445256[2]Duque VelasquezC, et al. VirolJ
2015;89:12,362–12,373.[3]HerbstA, et al.
Emerg Infect Dis. 2017;23:1598–1600.10.3201/eid2309.161474PMC557286728820384

## 238. A general mass spectrometry-based method of quantitating the oxidation of the methionine side chains in classical and atypical scrapie PrPSc

Christopher J. Silva^a^, Melissa L. Erickson-Beltran^b^, Inmaculada Martin-Burriel^c^, Juan José Badiola^d^, Jesus R. Requena^e^ and Rosa Bolea^a,f^

^a^Produce Safety & Microbiology Research Unit, USDA, WRRC, ARS; ^b^Produce Safety & Microbiology Research Unity, USDA, WRRC, ARS; ^c^University of Zaragoza, Zaragoza, Spain; ^d^University of Zaragoza; ^e^University of Santiago de Compostela; ^f^University of Zaragoza – Centre for TSE and emerging animal diseases

**ABSTRACT**

**Background/Introduction**: The structure of PrP^Sc^ is uncertain, but the most compelling data suggests that it is a four-rung β -solenoid. The rungs of the solenoid are β -sheet secondary structures held together by hydrogen bonds. Unstructured loops connect the more rigid β -sheet structures. The side chains of the amino acids in the β -sheets alternately project inside the solenoid or outside of it. This may make the reactivity of adjacent amino side chains dramatically different. The side chain of methionine can be oxidized under physiological conditions to the much more polar sulfoxide. Eukaryotic cells possess enzymes that reduce oxidized methionines, so the methionines of PrP^C^ should be largely unoxidized. Methionines present in PrP^Sc^ may be oxidized by chemical reagents or natural oxidative processes. Quantitating the extent of this oxidation can provide insight into the location of the methionines within the β -solenoid.

**Materials and Methods**: Plasmids containing relevant PrP sequences were prepared.^1 15^N-labelled internal standards were prepared by overexpressing the PrP genes in *E. coli* grown in minimal medium with ^15^NH_4_Cl as the sole nitrogen source. A set of peptides containing the methionines was identified that are derived from the tryptic or chymotryptic digestion of sheep PrP. A multiple reaction monitoring method (MRM) was developed to detect and quantitate each peptide.

**Results**: Calibration curves relating signal intensities of the various peptides and their absolute amounts were linear and had excellent correlation coefficients. The optimal digestion for all tryptic peptides was 20 h. For chymotrypsin the optimal digestion was 20 h, except for one peptide. In peptides containing two methionines, the oxidation state of each could be readily identified by a combination of chromatographic properties and the MRM method.

**Conclusions**: This approach can be used to quantitate the relative amounts of each oxidized methionine in sheep scrapie. By comparing the results obtained from classical and atypical scrapie, we gained insight into the structural differences between the two isoforms.

## 239. Hsp110 modifies prion infection *in vitro* and *in vivo*

Cristóbal Marrero-Winkens^a,b,c^ and Hermann M. Schätzl^a,b,c^

^a^Department of Comparative Biology and Experimental Medicine, Faculty of Veterinary Medicine, AB, Canada; ^b^Calgary Prion Research Unit, AB, Canada; ^c^Hotchkiss Brain Institute, University of Calgary, *AB*, Canada

**CONTACT** Cristóbal Marrero-Winkens cristobal.marrerowin@ucalgary.ca

**KEYWORDS:** Hsp110; chaperones; prion replication; prion fragmentation; disaggregase function

Mammalian heat shock protein 110 (Hsp110) family members cooperate with Hsp70 and Hsp40 to disaggregate misfolded proteins. Efficient *in vitro* disaggregation of α-synuclein and artificially denatured proteins by this tri-chaperone system has recently been reported. In yeast, it is well established that the disaggregase formed by Hsp70 and Hsp104 (which has no mammalian homolog) affects the maintenance of prion phenotypes. We hypothesize that the mammalian tri-chaperone system fulfils a similar function by participating in prion fragmentation and disaggregation. To test this, Hsp110 levels were transiently manipulated in 22L-ScN2a cells. Transient knockdown reduced the levels of PrP^Sc^, while overexpression led to a dose-dependent increase in PrP^Sc^. These effects are reminiscent of Hsp104 modulation in yeast. Next, Hsp110 overexpressing and wild-type mice were inoculated with 22L and Me7 prions. Overexpression of Hsp110 significantly prolonged the survival of Me7- but not 22L-inoculated animals. Sucrose gradient ultracentrifugation of brain homogenates derived from terminally diseased animals also revealed a trend towards larger PrP^Sc^ aggregates in overexpressing mice. Overall, these results confirm that Hsp110 is involved in PrP^Sc^ fragmentation and warrant further study. Finally, we also report on on-going studies, which include prion inoculation of different overexpressing mice as well as the generation of Hsp110 knockout and overexpressing cell lines using the CRISPR-Cas9 system and recombinant lentiviruses

## 240. The NLRP3-Caspase 1 Negatively Regulates Autophagy via TLR4-TRIF in Prion Peptide-Infected Microglia

Mengyu Lai†, Hao Yao†, Syed Zahid Ali Shah, Deming Zhao and Lifeng Yang

National Animal Transmissible Spongiform Encephalopathy Laboratory, College of Veterinary Medicine, China Agricultural University, Beijing, China

**CONTACT** Lifeng Yang yanglf2002@126.com

†Deceased.

**ABSTRACT**

**Introduction**: Prion diseases are neurodegenerative disorders characterized by the accumulation of misfolded prion protein, spongiform changes in the brain, and brain inflammation as a result of the wide-spread activation of microglia. Autophagy is a highly conserved catabolic process for the clearance of cytoplasmic components, including protein aggregates and damaged organelles, this process also eliminates pathological PrP^Sc^ as it accumulates during prion infection [1]. The NALP3 inflammasome is a multiprotein complex that is a component of the innate immune system and is responsible for the release of pro-inflammatory cytokines. Our previous study showed that the neurotoxic prion peptide PrP106-126 induces NALP3 inflammasome activation and subsequent IL-1β release in microglia [2]. Autophagy is involved in the regulation of the immune responses and inflammation in many diseases including neurodegenerative diseases. However, the relationship between autophagy and NALP3 inflammasome in prion diseases has not been investigated.

**Results**: In this study, we demonstrated that the processing and release of mature IL-1β is significantly enhanced by the inhibition of autophagy. Conversely, gene silencing of the NALP3 inflammasome promotes autophagy. Suppression of TRIF or TLR4 by siRNA attenuated PrP106-126-induced autophagy, which is indicating that the TLR4-TRIF signalling pathway is involved in PrP106-26-induced autophagy. Caspase 1 directly cleaved TRIF to diminish TLR-4-TRIF mediated autophagy. Our findings suggest that the inhibition of autophagy by NALP3 inflammasome is probably mediated by activated Caspase-1-induced TRIF cleavage [3]. This is the first study reporting that the NALP3 inflammasome complex negatively regulates autophagy in response to PrP106-126 stimulation in microglia, and partly explains the mechanism of autophagy inhibition by Caspase-1 in PrP106-126-induced BV2 cell activation.

**Conclusions**: Our findings suggest that autophagy up-regulation and inhibition of Caspase-1 may protect against prion-induced neuroinflammation and accelerate misfolded protein degradation and are potential therapeutic approaches for prion diseases.

### 

References[1]DereticV, et al.
Autophagy in infection,inflammation and immunity. Nat Rev Immunol
2013;13:722–737.2406451810.1038/nri3532PMC5340150[2]ShiF, et al.
Inhibition of phagocytosis and lysosomal acidification suppresses neurotoxic prion peptide induced NALP3 inflammasome activation in BV2 microglia. J Neuroimmunol. 2013;260:121–125.2368049010.1016/j.jneuroim.2013.04.016[3]FaureM., et al.
Pathogen-induced autophagy signaling in innate immunity. J Innate Immun. 2013;5:456–470.2365219310.1159/000350918PMC6741472

## 241. Evaluating the applicability of tauK18 as RT-QuIC reaction substrate for the analysis of samples collected from patients with neurodegenerative disorders

Martina Rossi^a^, Chiara Maria Giulia De Luca^a^, Elena De Cecco^a^, Joanna Narkiewicz^a^, Giulia Salzano^a^, Sara Maria Portaleone^b^, Antonio Emanuele Elia^c^, Roberto Eleopra^c^, Giuseppe Legname^a^, Giorgio Giaccone^d^ and Fabio Moda^d^

^a^Laboratory of Prion Biology, Department of Neuroscience, Scuola Internazionale Superiore di Studi Avanzati, Trieste, Italy; ^b^Neuropathology-Neurology 5 Unit, Fondazione IRCCS Istituto Neurologico Carlo Besta, Milan, Italy; ^c^Movement Disorders Department – Neurology I Unit, Fondazione IRCCS Istituto Neurologico Carlo Besta, Milano, Italy; ^d^Otolaryngology Unit, San Paolo Hospital, Department of Health Sciences, University of Milan, Milan, Italy

**CONTACT** Martina Rossi martina.rossi@sissa.it

**ABSTRACT**

**Introduction: **Neurodegenerative diseases are characterized by the accumulation of abnormally folded proteins in different regions of the brain [1,2]. Among them, Alzheimer’s disease (AD), Frontotemporal dementia (FTLD-tau), Corticobasal Degeneration (CBD), and Progressive Supranuclear Palsy (PSP) are associated to different abnormal conformations of the microtubule-associated tau protein. Clinical diagnosis of tauopathies is often challenging due to their overlapping symptoms, especially in the early stages [2]. Compelling evidence suggest that abnormally folded proteins appear in peripheral tissues of demented patients [3–5]. Therefore, we are currently exploiting the Real-Time Quaking Induced Conversion (RT-QuIC) assay to attempt detection of abnormal tau in peripheral tissues of patients with tauopathies.

**Materials and methods: **Recombinant tau fragment comprising the 4-repeats domain (tauK18) was used as substrate for RT-QuIC assay. *In vitro* produced tauK18 aggregates (referred to as artificial seeds) were serially diluted and added to new RT-QuIC reactions to assess its sensitivity. Similarly, brain homogenates (BHs) collected from patients with PSP, FTLD-tau and AD were analysed by means of RT-QuIC. BHs collected from patients with other dementias (including Parkinson’s disease, Dementia with Lewy bodies and Multiple System Atrophy) and subject not affected by neurodegenerative disorders (NDP) were used as controls. Olfactory mucosa (OM) samples collected from patients with a clinical diagnosis of CBD, PSP, and AD are currently under RT-QuIC analysis. OM from PD, MSA, DLB patients, and healthy subjects are included as controls.

**Results:** All dilutions of artificial seed efficiently accelerated the kinetics of tauK18 aggregation, thus revealing the ability of RT-QuIC in detecting up to attograms of tauK18 aggregates. BHs of patients with PSP, FTLD-tau and AD accelerated tauK18 aggregation with greater efficiency than that of DLB, PD, MSA, and NDP. Preliminary data collected from RT-QuIC analysis of OM samples suggest that the aggregation of tauK18 is more efficient when the reaction is supplemented with CBD and PSP samples. Less efficient aggregation was observed after the addition of the other OM samples.

**Conclusions:** The aggregation kinetics of tauK18 used as RT-QuIC reaction substrate can be influenced by other factors other than tau itself, therefore representing an important issue especially from a diagnostic point of view. The presence of other amyloidogenic proteins (e.g. α-synuclein or β-amyloid) can play a pivotal role in promoting tauK18 aggregation, thus generating misleading results. For this reason, we are currently coupling RT-QuIC analysis with structural and biochemical studies to assess whether the final reaction products acquire peculiar features useful for disease discrimination.

**KEYWORDS:** tauK18; RT-QuIC; diagnosis; tauopathies

### 

References[1]SotoC.
Nat Rev Neurosci. 2003;4:49.10.1038/nrn100712511861[2]IrwinDJ.DisordParkinsonism Relat
2015;22 Suppl 1(01):S29–S33.10.1016/j.parkreldis.2015.09.020PMC466261126382841[3]ModaF. et al., N Engl J Med. 7 August
2014;371(6):530–539.10.1056/NEJMoa1404401PMC416274025099577[4]SaijoE. et al., NeuropatholActa
2017
5;133(5):751–765.10.1007/s00401-017-1692-z28293793[5]RedaelliV, BistaffaE. et al., Sci Rep. 7 April
2017;7:46269.10.1038/srep46269PMC538424428387370

## 242. Incubation periods of classical and atypical bovine spongiform encephalopathies (BSE) reflect an inverse relationship between PrP^Sc^-associated neuroinflammation and autophagy

Najiba Mammadova^a^^,^^b^^,^^c^, M. Heather West Greenlee^b^^,^^c^^,^^d^, S. Jo Moore^e^, Donald S. Sakaguchi^a^^,^^c^ and Justin J. Greenlee^e^

^a^Department of Genetics, Development and Cell Biology, Iowa State University, Ames, IA, USA; ^b^Immunobiology Graduate Program, Iowa State University, Ames, IA, USA; ^c^Neuroscience Graduate Program, Iowa State University, Ames, IA, USA; ^d^Department of Biomedical Sciences, Iowa State University College of Veterinary Medicine, Ames, IA, USA; ^e^Virus and Prion Research Unit, National Animal Disease Center, USDA, Agricultural Research Service, Ames, IA,USA

**CONTACT** M. Heather West Greenlee mheather@iastate.edu

**ABSTRACT**

Neurodegenerative protein misfolding disorders are a group of diseases in both humans and animals with a similar mechanism of disease progression. These diseases, that include Alzheimer’s disease, Parkinson’s disease, chronic wasting disease, and bovine spongiform encephalopathy (BSE) result from aberrant folding and accumulation of disease specific proteins. Transmissible spongiform encephalopathies (TSEs) are diseases that result from improper folding and accumulation of the prion protein. Due to their transmissibility, TSEs are an important experimental model to study the basic pathobiology of all protein misfolding disorders. TSE strain variations can influence disease phenotypes such as host susceptibility, biochemical and immunohistochemical profiles, and incubation periods. BSE is a TSE that occurs in cattle that can be subdivided into three different strains: classical BSE, atypical high (H)-type, and atypical low (L)-type BSE. Both H-type and L-type BSEs, have shorter incubation periods after experimental inoculation and, therefore, an accelerated disease progression when compared to classical BSE. Currently, there is a lack of knowledge about the factors that influence accumulation of misfolded proteins and disease progression making this a key challenge for the development of therapies for protein misfolding diseases. In this study, we used the differences between classical (*n* = 12) and atypical BSEs (*n* = 20) as a model to identify the molecular factors associated with disease progression. The NLRP3 inflammasome is a critical component of the innate immune system that leads to release of IL-1β (Interlukin-1β), an important regulator of neuroinflammation in many protein misfolding diseases. Macroautophagy is an intracellular mechanism that plays an essential role in protein clearance and homeostasis. Here we used immunohistochemistry to assess the retina and three brain regions (thalamus, caudate nucleus, and brainstem at the level of the obex) to investigate the relationship between disease incubation period, PrP^Sc^ accumulation, neuroinflammation, and changes in macroautophagy. We demonstrate that atypical BSEs, characterized by shorter incubation periods, present with greater accumulation of PrP^Sc^, glial-cell activation, NLRP3 inflammasome activation, and decreased autophagy. Our work demonstrates a relationship between disease time course, neuroinflammation, and the autophagic stress response, that has not been previously reported. This work may help identify novel therapeutic approaches that can delay or even prevent the progression of protein-misfolding diseases.

## 243. Comparative effect of green tea polyphenols on the amyloid aggregation of human prion protein

Nikita Admane and Abhinav Grover

School of Biotechnology, Jawaharlal Nehru University, New Delhi, India

**CONTACT** Nikita Admane niki.admane@gmail.com

**ABSTRACT**

Misfolding and aggregation of human prion protein (HuPrP) is a hallmark to a variety of neurological disorders, commonly known as the Transmissible Spongiform Encephalopathies (TSEs). Despite several efforts to recognize effective small molecule inhibitors of amyloid disorders, presently no effective therapeutic agent is available for the neurodengerative amyloidosis. Herein, we examined the protective efficacy of two green tea polyphenols, GTP1 and GTP2 against the amyloid conversion of cellular human prion protein to the disease forming scrapie conformation, employing a combination of computational, and experimental approach. Collective results from ThT, TEM, AFM, DLS, and CD experiments show that both the GTPs exhibit an inhibitory effect on the structural transition of HuPrP monomers into beta sheet rich structures, thereby reducing the amyloid fibrillization of HuPrP. Further, MTT assay and confocal microscopy revealed that both the catechins increase the cell viability by reducing toxicity associated with oligomeric species in neuronal cell models. But interestingly, GTP1 has a stronger inhibitory effect on HuPrP amyloidogenesis as compared to GTP2 due to minor differences in the structure of these two polyphenols. Both the compounds stably interact with the hydrophobic residues in the binding pocket of HuPrP which encompasses the β2-α2 loop and the C-terminal region of the α3 helix. Binding mechanisms of GTP1 and GTP2 with HuPrP was analysed by molecular dynamics simulations. The 4Å region of HuPrP binding pocket around these two compounds reveals that GTP1 forms more hydrogen bonds and hydrophobic interactions (as compared to GTP2) with the residues critical in the conversion of cellular to scrapie form of HuPrP, this may be the reason behind making GTP1 a more potent inhibitor. Thus, small molecules (like catechins and polyphenols) that can directly interact with the critical hydrophobic patches of HuPrP can act as inhibitors of prion amyloidogenesis and hold a substantial therapeutic potential. The green tea polyphenols might also be of synergistic benefit in treating the prion diseases.

## 244. The use of recreational hunters in Chronic Wasting Disease (CWD) management: behaviour and incentives

Lusi Xie^a^, Vic Adamowicz^a^ and Patrick Lloyd-Smith^b^

^a^University of Alberta; ^b^University of Saskatchewan

**CONTACT** Lusi Xie lxie@ualberta.ca

**ABSTRACT**

**Background:**While many jurisdictions in North America have implemented some management programmes to address Chronic Wasting Disease (CWD), the disease continues to spread. Although epidemiological models have proposed depopulating infected animals to reduce disease prevalence and spread by increasing hunter harvest (e.g. Potapov et al. 2016) [1], these models do not evaluate the incentives required to increase hunter harvest or the impacts of wildlife disease and management programmes on recreational activity and value. A better understanding of human behavioural responses will help effectively incorporate recreational hunters in wildlife disease control. The objective of this study is to examine the use of incentives to recreational hunters for CWD management by studying hunting trip decisions in response to CWD and its management. We develop an empirical model, with a focus on spatial and temporal substitution patterns of recreational activities, as well as economic benefits of a new recreation season.

**Materials and Methods:**The data used in this study are from an online survey of recreational hunters in Alberta and Canada. Five thousand hunters were sampled and a response rate of approximately 20% resulted in a usable sample of 707. Hunters were asked to recall hunting trips in 2017 and stated intended visitation with proposed extended hunting seasons for CWD management. We develop a discrete-continuous empirical model that captures hunting trip decisions on sites, periods and frequencies. Explanatory variables included in the model are CWD prevalence levels, policy dummy variables, individuals’ socio-demographic variables as well as travel and time costs. With the estimated parameters, spatial and temporal substitution patterns are captured, measures of the potential harvest of affected deer populations, and economic benefits, are calculated.

**Results and Conclusions:**We find a negative but insignificant coefficient of CWD which indicates that individuals do not avoid hunting in CWD-infected areas. Individuals are likely to take around six additional trips on average during the extended seasons – this is more than half of the average trips (around 10) they actually took in 2017. We find that individuals are likely to substitute from hunting in areas with lower CWD risks to hunting in the most CWD-infected areas in both seasons. Individuals are better off from an extended season for CWD management. Our findings indicates that it is possible to direct hunting trips for CWD control by extending hunting seasons in CWD-infected areas without additional monetary or non-monetary incentives.

**KEYWORDS:** Chronic wasting disease; recreation demand

### 

Reference[1]Potapov, et al. PLoS One
2016;11(3):e0151039.

## 245. PrPC knockdown by Liposome-siRNA-peptide complexes (LSPCs) prolongs survival and normal behaviour in prion-infected mice immunotolerant to treatment

Heather Bender^a,b^, Noelle Noyes^a,c^, Jessica L Annis^a^, Amanda Hitpas^a^, Luke Mollnow^a^, Kendra Croak^a^, Sarah Kane^a^, Kaitlyn Wagner^a^, Steven Dow^a,d^ and Mark D. Zabel^a^

^a^Prion Research Center, Department of Microbiology, Immunology and Pathology, College of Veterinary Medicine and Biomedical Sciences, Colorado State University, Fort Collins, CO, USA; ^b^Department of Pharmacology, University of Colorado School of Medicine, Aurora, CO, USA; ^c^Department of Veterinary Population Medicine, College of Veterinary Medicine, University of Minnesota, St. Paul, MN, USA; ^d^Center for Immune and Regenerative Medicine, Department of Clinical Sciences, Colorado State University, Fort Collins, CO, USA

**CONTACT** Mark D. Zabel mzabel@colostate.edu

**ABSTRACT**

Prion diseases are members of neurodegenerative, protein misfolding diseases (NPMDs) that include Alzheimer’s, Parkinson’s and Huntington diseases, Amyotrophic Lateral Sclerosis, tauopathies, traumatic brain injuries, and chronic traumatic encephalopathies. No known therapeutics improve quality of life or survival times of humans afflicted with prion disease. We and others developed a new approach to NPMD therapy based on reducing the amount of the normal, host-encoded protein that acts as a substrate for misfolding into pathologic forms using RNA interference, a pathway that decreases levels of mRNA encoding a particular protein. We developed a non-invasive therapeutic delivery system consisting of siRNA complexed to liposomes and addressed to the central nervous system using a targeting peptide derived from Rabies virus glycoprotein and delivered transvascularly. These liposome-siRNA-peptide complexes (LSPCs) cross the blood-brain barrier and deliver PrP siRNA to neuronal cells to decrease expression of the normal cellular prion protein, PrPC, which acts as a substrate for prion replication. Here we show that LSPCs can extend survival and improve behaviour of prion-infected mice that remain immunotolerant to treatment. LSPC treatment may be a viable therapy for prion and other NPMDs that can improve the quality of life of patients at terminal disease stages.

## 246. Congenital hydrocephalus with drainage problems leading to CJD and Abeta accumulation. A case report

Annemieke Rozemuller, Wim Spliet, Anja Horstman, Will Hermsen, Vandana Gupta and Wim van Hecke

Prionlab. Dept. of Pathology, University Medical Center, Utrecht, The Netherlands

**CONTACT** Annemieke Rozemuller J.M.Rozemuller@umcutrecht.nl

Creutzfeldt-Jakob *amyloid beta *hydrocephalus *drainage.

**ABSTRACT**

**Introduction**: We describe the neuropathology of a 48-year-old man with congenital hydrocephalus and CSF drain from his 18th year who became slow and got rapidly progressive dementia in 2 months, with epilepsy, dysarthria, gait abnormalities and myoclonus. The EEG was typical for CJD. The RT-QuIC test was positive.

**M&M**: The brain autopsy was performed in the prionlab UMCUtrecht. The brain weight was 1410 g; the drain was situated in the bottom of the fourth ventricle. No hydrocephalus was seen but the aquaduct was extremely small. Twenty-five blocks were taken from relevant areas and stained for HE, Kluver-PAS as well immunohistochemically for 3F4. Next to that we stained for Abeta and tau.

**Results**: Routine staining showed fine vacuolar spongiosis of the grey matter and prion staining fitted with a histotype of MM/MV1 according to the histotyping of Parchi et al. (2012). In addition numerous diffuse Abeta plaques were found in the cortex but no classical amyloid plaques or (pre)tangles in the tau staining. Just a few dystrophic neurites.

**Conclusion**: This case report shows that congenital hydrocephalus with drainage problems can lead to protein misfolding with both prion accumulation (Creutzfeldt – Jakob disease) and numerous Abeta plaques on the age of 48 year. This supports earlier theories that drainage is essential for removing misfolded proteins.

## 247. Use of modern population genetic techniques to investigate the effects of the epidemic of kuru, a unique prion disease in the Papua New Guinea highlands

Liam Quinn^a^, Jerome Whitfield^a^, Garrett Hellenthal^b^, Michael Alpers^c,d^, John Collinge^a^, and Simon Mead^a^

^a^MRC Prion Unit at UCL, UCL Institute of Prion Diseases, London, UK; ^b^Department Genetics, Evolution & Environment, University College London; ^c^Centre for International Health, Curtin University, Perth, Australia; ^d^Papua New Guinea Institute of Medical Research, Goroka, Papua New Guinea

**CONTACT** Liam Quinn l.quinn@prion.ucl.ac.uk; Jerome Whitfield j.whitfield@prion.ucl.ac.uk

**ABSTRACT**

**Background**: Kuru was the first observed and to this day remains the largest human prion disease epidemic. It devastatingly affected the Fore linguistic and nearby groups in the Papua New Guinea (PNG) highlands. The condition was transmitted by consumption of prion infected material at mortuary rituals. Over the first 20 documented years of the epidemic ~2,400 people died from kuru in communities with populations of ~40,000 individuals. The Eastern Highlands of Papua New Guinea where the Fore speaking communities reside is marked for its cultural and linguistic diversity. During the kuru epidemic the region was undergoing a marked transition with European contact and colonial administration. These factors present challenges to further understanding the population response to kuru specifically and approaches to find novel variants that affected disease predisposition.

**Materials and Methods**: We analysed a unique genome wide array dataset of 1,513 individuals from the affected communities from 22 linguistic groups and 50 across the Eastern Highlands of PNG. We used population genetic techniques including Chromopainter analysis and fineStructure clustering to understand the population structure of the region, analyse patterns of gene flow and migration, tested for evidence of historical admixture events and evidence of genetic signatures of the impact of kuru on the Fore region including the marked sex bias in cases observed during the epidemic.

**Results**: We find a strong population structure with Principal Component Analysis and fineStructure clustering showing a strong tendency for individuals to cluster with individuals from the same linguistic grouping. Mean F_ST_ values observed in the Eastern Highlands were 0.02, far greater than those observed in the United Kingdom (0.0007) reflecting the degree of distinction between groups. Although we find that linguistic group membership explains many aspects of population structure we also find that topographical features and cultural differences also exert strong forces. Gene flow between linguistic groups is evident with the presence of individuals reflecting historical processes of short and long distance migration.

**Conclusion**: We show that the affected populations have displayed remarkable resilience with no perceptible derivation in population genetic parameters overall despite facing such an existential threat. The marked population structure in the region and processes of gene flow present challenges for ongoing investigations into the genetic response and genetic architecture of kuru disease.

**KEYWORDS:** Kuru; population genetics; natural selection; genetic epidemiology

## 248. How the PrP^C^ C-Terminal, globular domain regulates its toxic N-Terminal Domain

Glenn L. Millhauser^a^, David A. Harris^b^, Graham Roseman^a^, Kevin Schilling^a^, Kate Markham^a^, Alex McDonald^b^ and Bei Wu^b^

^a^Department of Chemistry & Biochemistry, UC Santa Cruz, Santa Cruz, CA, USA; ^b^Department of Biochemistry, Boston University School of Medicine, Boston, MA, USA

**CONTACT** Glenn L. Millhauser glennm@ucsc.edu

**ABSTRACT**

The cellular prion protein (PrP^C^) is composed of two primary segments – a helical C-terminal domain and a flexible N-terminal domain. Research over the last 5 years finds that the N-terminal domain is responsible for the monomeric protein’s neuronal toxicity as revealed by toxic, spontaneous, electrophysiological currents and antibody induced neuronal death in cultured cells and brain slices. This inherent toxicity is arrested, however, by the regulatory C-terminal domain. Our research is focused on revealing the molecular mechanism underlying this critical regulatory function. The physiologic metal ions copper and zinc both bind to the octapeptide repeat segments in the N-terminal domain. Using advanced magnetic resonance approaches, along with electrophysiology measurements, we have identified a previously unseen intramolecular switch in PrP^C^ whereby copper or zinc drive a tertiary contact between the N-terminal domain and a negatively charged, highly conserved surface on the C-terminal domain. Consistent with this model, we find that amino acid alterations at the docking surface that disrupt metal ion binding lead to toxic transmembrane currents. Together, our research provides a new paradigm for understanding PrP^C^ toxicity and how familial mutations cause prion disease.

**Funding**

Supported by NIH grants R01 GM06790 (GM), S10 OD018455 (GM) and R01 NS065244 (DH).

### 

References[1]Spevacek, et al. Structure
2013;21:236–246.[2]Evans, et al. Structure
2016;24:1057–1067.[3]Wu, et al.
eLife. 2017 DOI:10.7554/eLife.23473.001

## 249. Validation studies of bovine heparin manufacturing process spiked with BSE agent

Cyrus Bett, Omozusi Andrews, Teresa Pilant, David M. Asher and Luisa Gregori

FDA/CBER/OBRR/DETTD/LBTSEA, Silver Spring, MD, USA

**CONTACT** Cyrus Bett Cyrus.bett@fda.hhs.gov

**ABSTRACT**

Heparin is an injectable anticoagulant marketed in the US since the 1930’s and used by 12 million Americans every year. Heparin is usually processed from mucosa of porcine or bovine intestines. In 2000, US manufacturers, concerned about possible contamination of bovine tissues with bovine spongiform encephalopathy (BSE) agent, stopped manufacturing bovine heparin. Thus, only porcine heparin is now marketed in the US, and about 75% originates from a single foreign country. Alternative sources of crude heparin would alleviate ongoing concerns about relying on single country and single animal species as a source of all heparin. This would also mitigate the risk of potential future shortages and episodes of adulteration, observed in the past. Bovine heparin is an obvious option, but BSE agent clearance by the bovine heparin manufacturing process should be evaluated. To this end, we applied a four-step bench-scale heparin purification protocol resembling a typical heparin manufacturing process to investigate removal of the spiked BSE agent. We removed aliquots from each step and analysed them for residual abnormal prion protein (PrP^TSE^) using real-time quaking-induced conversion (RT-QuIC) assay, and for infectivity using animal bioassays. The purification process reduced infectivity and PrP^TSE^ by more than 3 logs. These findings show that the heparin manufacturing process has the capacity to remove substantial amounts of the BSE agent and support efforts to reintroduce safe bovine heparin in the USA.

## 250. Interaction with phosphatidic acid leads to prion protein fibrilization

R. F. D. Santos^a,c^, C. Alves^a,b^, L. V. Menezes^b,c^, J. M. A. Brito^a,c^, J. L. Silva^a,c^ and T. C. R. G. Vieira^a,c^

^a^Medical Biochemistry Institute Leopoldo De Meis, Federal University of Rio de Janeiro; ^b^Federal Institute of Rio de Janeiro; ^c^National Institute of Cience and Tecnology of Estructural Biology and Bioimaging, Brazil

**ABSTRACT**

Transmissible Spongiform Encephalopathies (TSEs) are neurodegenerative diseases caused by misfolding of the prion protein (PrP), which assumes a pathogenic conformation and propagates the infection through the conversion of native proteins. Recent models indicate the possible role of biological ligands in this process, which would act as cofactors in the conversion reaction. Lipids present in the mammalian cell membrane, especially in brain cells, have been suggested as endogenous adjuvants promoting prion aggregation. Despite this, little is known about the physical and chemical characteristics of this interaction. This study investigates the interaction and structural changes of recombinant PrP (murine, hamster and rabbit) in the presence of Phosphatidic Acid (PA) lipid vesicles, in an *in vitro* model. Full length or 90–231 fragment of rPrP^C^ were incubated with PA vesicles. The samples were then analysed by spectroscopic techniques to detect changes in aggregation and tertiary structure. Increasing concentrations of PA raised rPrP light scattering, followed by changes in tryptophan fluorescence, indicating the formation of large aggregates. Interaction with PA induced an increase in beta-sheet content, with an intermediate enrichment compared to PrP^Sc^. These aggregates had an amorphous and amyloid fibre structure, shown by thioflavin T binding and microscopy observation. PrP truncated form showed more tendencies to form fibrils. rabPrP:PA aggregation was less robust that the other constructs and pH dependent. The results indicate that PA vesicles interact with rPrP, leading to the formation of amorphous aggregates and amyloid fibrils, close to PrP^Sc^ secondary structure. The absence of the N-terminal is important to follow a fibrillization pathway induced by PA. **rabPrP is able to interact with the biological cofactor tested, but with less effect depending on pH, suggesting that the different binding sites exposed at these pHs can be important.**

**KEYWORDS:** Prion; neurodegenerative diseases; lipids; aggregation; vesicles

Sponsorship: FAPERJ, CNPq, CAPES, IFRJ, INBEB

## 251. The photodynamic inactivation reduces prion infectivity in phthalocyanine treated RML brain homogenate

Marie Kostelanska^a^, Zdenka Backovska Hanusova^a^, Jakub Soukup^a^, Radoslav Matej^b^, Karel Holada^a^

^a^Institute of Immunology and Microbiology, First Faculty of Medicine, Charles University and General University Hospital in Prague, Czech Republic; ^b^Department of Pathology and Molecular Medicine, Thomayer Teaching Hospital, Prague, Czech Republic

**CONTACT** Marie Kostelanska marie.kostelanska@lf1.cuni.cz

**ABSTRACT**

**Introduction**: Prions are highly resistant to conventional sterilization procedures and currently recommended inactivation methods include treatment with highly corrosive chemicals like 2% NaClO or 1M NaOH. Such harsh treatment is incompatible with a number of delicate medical tools. We have previously demonstrated that nontoxic disulfonated hydroxyaluminum phthalocyanine (AlPcOH(SO_3_)_2_) can be used to initiate photodynamic inactivation (PDI) of prions at ambient pressure and temperature.^1^ The aim of this study was to demonstrate the effectiveness of PDI of prions by bioassay in laboratory mouse.

**Methods**: The PDI was performed on 1% RML or uninfectious CD1 brain homogenates (BH) treated with 25 µg ml^−1^ of AlPcOH(SO_3_)_2_ and exposed to red light (18 min, 4 ×2.5W LED). Control RML inoculum was prepared similarly but was kept in the dark. In mouse bioassay, CD1 female mice (*n* = 10) were intracerebrally inoculated with a 25-µl aliquot of AlPcOH(SO_3_)_2_-light treated RML BH or control RML BH containing AlPcOH(SO_3_)_2_ but not irradiated by light. Other controls included mice (*n* = 5) inoculated with untreated, uninfectious CD1 BH or CD1 homogenate containing AlPcOH(SO_3_)_2_, either irradiated or nonirradiated. For comparison groups of mice (*n* = 5) were inoculated with 10-fold dilutions of RML BH (10^−2^ to 10^−6^). The animals were sacrificed after reaching the terminal disease phase. The presence of PrPres in mouse brains was detected by WB and evaluation of PrPres distribution in brains by immunohistochemistry is ongoing.

**Results**: Mice inoculated with PDI-treated RML BH (at a 10^−2^ dilution) survived significantly longer (204 ± 23 days) than mice inoculated with the homogenate containing an identical amount of AlPcOH(SO_3_)_2_, that was not exposed to light (165 ± 11 days). Their survival was also significantly longer than that of mice inoculated with straight 10^−2^, 10^−3^, and 10^−4^ dilutions of the RML BH, who survived 169 ± 11, 165 ± 12 and 176 ±15 days, respectively. The transmission rate was 100% in all groups of mice including serial dilution (10^−2^ to 10^−6^). A comparison of survival time-based on the regression line suggested a decrease in the prion infectivity titer of the RML BH of more than 4 orders of magnitude after PDI treatment.

**Conclusions**: Our results suggest that PDI with AlPcOH(SO_3_)_2_ leads to substantial decrease (~4 log_10_) of prion infectivity in RML BH. The PDI of prions by light induced photocatalytic activity of phthalocyanines represents promising way of prion decontamination.

**KEYWORDS:** Prion; phthalocyanine; photodynamic inactivation; decontamination; mouse bioassay

**Funding**

The project was supported by AZV NV18-04–00179.

### 

Reference[1]Janouskova, et al. J Gen Virol
2012;93(Pt11): 2512–7.10.1099/vir.0.044727-022855785

## 252. RT-QuIC as an antemortem diagnostic tool to detect chronic wasting disease in deer skin

Natália C. Ferreira^a^, Jorge M. Charco^b^, Michael A. Metrick^a^, Christina D. Orru^a^, Andrew G. Hughson^a^, Joaquín Castilla^a^, Michael W. Miller^c^ and Byron Caughey^a^

^a^Laboratory of Persistent Viral Diseases, Rocky Mountain Laboratories, National Institute of Allergy and Infectious Diseases, National Institutes of Health, Hamilton, MT, USA; ^2^ CIC bioGUNE, Derio (Bizkaia), Spain; ^3^Colorado Division of Parks and Wildlife, Wildlife Health Program, Fort Collins, Colorado, USA

**CONTACT** Natália C. Ferreira natalia.docarmoferreiradearaujo@nih.gov

**ABSTRACT**

Chronic wasting disease (CWD) is a fatal prion disease which affects cervids. This disease has an asymptomatic incubation time of 2–4 years, and during this period they can shed prions through saliva, feces, urine and placental tissue, contaminating the environment. Indeed, CWD highly contagious between cervids and shedding from live, infected animals likely contributes to its rapid spread. So far, CWD has been reported at least in 24 states in the US, as well as Canada, South Korea and Norway. To date, there is no evidence of CWD transmission to humans. However, in infected areas, deer population can drop as much as ~25 percent. Currently, there are two tests approved to diagnose CWD: immunohistochemistry and ELISA. However, these tests are applied *postmortem* and tissue types approved are the medial retropharyngeal lymph nodes and a specific region of brainstem (obex). Here we describe our efforts to adapt the real time quaking-induced conversion (RT-QuIC) as a diagnostic tool to detect PrP^CWD^ in deer ear skin. Our initial analysis of a blinded panel of 50 samples yielded 82% sensitivity and 75% specificity. We are working to improve the conditions and performance of this assay, given that it might be useful for *antemortem* CWD diagnostics and surveillance.

## 253. Structural bioinformatics study on mechanisms of PrPc conformational stabilization mediated by the β 2- α 2 loop

Patricia Soto^a^, Frances Morden^b,c^, India Claflin^c^ and Alyssa Bursott^b,c^

^a^Physics department; ^b^Neuroscience program; ^c^Biology department, Creighton University, Omaha, NE, USA

**ABSTRACT**

Understanding the factors that stabilize the globular C-terminal of the cellular form of the prion protein is key to decipher PrP^C^ to PrP^Sc^ conversion. The mechanism of this process is not well understood; however, studies suggest several factors affect the likelihood for prion infection. To understand the conformational stability of PrP^C^, we examined the role of residue mutations, dimerization, and gpi-anchoring using structural bioinformatics techniques. Using structural bioinformatics techniques, we examined the role of point mutations and dimerization in the varying susceptibilities of PrP^C^ structures. Our study on known resistant sheep PrP^C^ structures indicates that key gene polymorphisms alter the network of residue interactions that involve the β 2- α 2 loop. This lowered susceptibility to initial protein misfolding likely occurs by rendering higher structural stability than the susceptible form. Our examination of prion protein dimerization suggests that the hydrophobic interactions at the dimer interface keep the dimer stable in the bulk and on the cell surface. The rotational mobility of dimers with respect to the cell surface is modulated by the gpi-anchoring state of the PrP^C^ protein. With a comprehensive view of our results, we propose that if prion protein fibrillogenesis occurs following the nucleation-polymerization model, then key polymorphisms raise the energetic barrier to prevent templated prion protein conversion and dimerization conceals recognition spots that otherwise would be recognized by PrP^Sc^ to bind and initiate prion protein conversion.

## 254. Humanized recombinant antibody fragments as tools for structural analyses of BSE and other prions

Vineet Rathod^a,b^, Jimmy Lu^a,b^, José M Flores-Fernández^a,b^, Xinli Tang^a,b^, Razieh Kamali-Jamil^a,b^, Leonardo Cortez^a,c^, Assunta Senatore^d^, Simone Hornemann^d^, Valerie Sim^a,c^, Adriano Aguzzi^d^ and Holger Wille^a,b^

^a^Center for Prions and Protein Folding Diseases, University of Alberta, Edmonton, Canada; ^b^Department of Biochemistry, University of Alberta, Edmonton, Canada; ^c^Department of Medicine, University of Alberta, Edmonton, Canada; ^d^Institute of Neuropathology, University of Zürich, Zürich, Switzerland

**ABSTRACT**

Prion diseases are rare, inexorably progressive, and fatal neurodegenerative disorders, with no therapy other than palliation. Prion diseases are unique compared in that they are transmissible, whereby the infectious agent (PrP^Sc^) is propagated through the reproduction of its secondary, tertiary, and quaternary structure [1]. PrP^Sc^ is a conformationally altered isoform of the cellular prion protein, PrP^C^, which is a normal cell surface glycoprotein. Prion diseases affect humans (e.g. Creutzfeldt-Jakob disease: CJD), as well as a variety of animals, including chronic wasting disease (CWD) in deer and elk, scrapie in sheep and goats. Bovine Spongiform Encephalopathy (BSE) is an acquired transmissible prion disease in cattle, colloquially named ‘mad cow’ disease. It was first described in 1986 in the UK and has raised serious concerns about its transmission to humans, where it is known as variant CJD through consumption of contaminated meat [2]. While the structure of recombinant PrP^C^ is extensively studied using various biophysical techniques, no high-resolution structure of PrP^Sc^ has been published, and only its overall architecture as a four-rung ß-solenoid is known [3,4].

Fab fragments (Fabs) are small antibody derivatives that maintain antigen-binding capacity, and can be used to study the structure of their antigens. One of the advantages of Fabs over intact antibodies is their small size, which allows them to penetrate deeper into protein aggregates, such as amyloid fibrils. We are using recombinant Fabs as an alternate approach in deciphering the structure of PrP^Sc^ via immunogold labelling, electron microscopy, and difference mapping. One of our Fab library targets the four main regions of PrP: the charged cluster 1, octapeptide repeats, charged cluster 2, and globular domain. A second set of Fabs was recombinantly engineered from monoclonal antibodies (mAbs) that specifically recognize native PrP^Sc^. These new PrP^Sc^-specific mAbs (IgGs and IgMs) were recently generated in the Wille lab using a rationally designed, structure-based mimic for PrP^Sc^.

The main goal is to use *E. coli* to express fully functional Fabs from both Fab libraries. Functionality assays conducted after the expression and purification of the Fabs indicate that some of them are able to bind native PrP^Sc^ fibrils. Complexes between Fabs and purified BSE prion fibrils are being analysed using immunogold labelling and electron microscopy. Electron micrographs of suitable prion fibrils will be used to generate 3D reconstructions of labelled and unlabelled BSE fibrils and provide insights into the orientation of the respective epitopes in the ß-solenoid architecture of PrP^Sc^.

### 

References[1]WilleH., RequenaJ.
The structure of PrPSc prions. Pathogens. 2018;7(1):20.10.3390/pathogens7010020PMC587474629414853[2]AguzziA., SigurdsonC.J.
Antiprion immunotherapy: to suppress or to stimulate?
Nat Rev Immunol. 2004;4(9):725–736.1534337110.1038/nri1437[3]RequenaJ.R., WilleH.
The structure of the infectious prion protein and its propagation. Prog Mol Biol Transl Sci. 2017;150:341–359.2883866710.1016/bs.pmbts.2017.06.009[4]Vazquez-FernandezE., et al.
The structural architecture of an infectious mammalian prion using electron cryomicroscopy. PLoS Pathog. 2016;12(9):e1005835.2760684010.1371/journal.ppat.1005835PMC5015997

## Targeting proteopathic seeds in Alzheimer’s disease

Mathias Jucker

Department of Cellular Neurology, Hertie Institute for Clinical Brain Research, and German Center for Neurodegenerative Diseases (DZNE), Tübingen, Germany

Abstract not available.
